# Lipid Nanoparticles Deliver the Therapeutic VEGFA mRNA In Vitro and In Vivo and Transform Extracellular Vesicles for Their Functional Extensions

**DOI:** 10.1002/advs.202206187

**Published:** 2023-02-19

**Authors:** Muhammad Nawaz, Sepideh Heydarkhan‐Hagvall, Benyapa Tangruksa, Hernán González‐King Garibotti, Yujia Jing, Marco Maugeri, Franziska Kohl, Leif Hultin, Azadeh Reyahi, Alessandro Camponeschi, Bengt Kull, Jonas Christoffersson, Ola Grimsholm, Karin Jennbacken, Martina Sundqvist, John Wiseman, Abdel Wahad Bidar, Lennart Lindfors, Jane Synnergren, Hadi Valadi

**Affiliations:** ^1^ Department of Rheumatology and Inflammation Research Institute of Medicine Sahlgrenska Academy University of Gothenburg Gothenburg 41346 Sweden; ^2^ BioPharmaceuticals R&D Early Cardiovascular Renal and Metabolism (CVRM) Bioscience Cardiovascular AstraZeneca Gothenburg Mölndal 43183 Sweden; ^3^ Systems Biology Research Center School of Bioscience University of Skövde Skövde SE‐54128 Sweden; ^4^ Advanced Drug Delivery Pharmaceutical Sciences BioPharmaceuticals R&D AstraZeneca Gothenburg Mölndal 43183 Sweden; ^5^ Safety Innovations Clinical Pharmacology and Safety Sciences R&D AstraZeneca Gothenburg Mölndal 43183 Sweden; ^6^ BioPharmaceuticals R&D Discovery Sciences Translational Genomics AstraZeneca Gothenburg Mölndal 43183 Sweden; ^7^ Department of Medical Biochemistry and Biophysics Karolinska Institute Solna Stockholm 17177 Sweden; ^8^ BioPharmaceuticals R&D Clinical Pharmacology and Safety Science Imaging and Data Analytics AstraZeneca Gothenburg Mölndal 43183 Sweden; ^9^ Institute of Pathophysiology and Allergy Research Medical University of Vienna Vienna 1090 Austria; ^10^ Department of Molecular and Clinical Medicine, Institute of Medicine, Sahlgrenska Academy University of Gothenburg Gothenburg 41345 Sweden

**Keywords:** endocytosis, extracellular vesicles, in vivo, lipid nanoparticles, LNP‐mRNA, luciferase mRNA, mRNA copy number, uptake, VEGF‐A mRNA

## Abstract

Lipid nanoparticles (LNPs) are currently used to transport functional mRNAs, such as COVID‐19 mRNA vaccines. The delivery of angiogenic molecules, such as therapeutic *VEGF‐A* mRNA, to ischemic tissues for producing new blood vessels is an emerging strategy for the treatment of cardiovascular diseases. Here, the authors deliver *VEGF‐A* mRNA via LNPs and study stoichiometric quantification of their uptake kinetics and how the transport of exogenous LNP‐mRNAs between cells is functionally extended by cells’ own vehicles called extracellular vesicles (EVs). The results show that cellular uptake of LNPs and their mRNA molecules occurs quickly, and that the translation of exogenously delivered mRNA begins immediately. Following the *VEGF‐A* mRNA delivery to cells via LNPs, a fraction of internalized *VEGF‐A* mRNA is secreted via EVs. The overexpressed *VEGF‐A* mRNA is detected in EVs secreted from three different cell types. Additionally, RNA‐Seq analysis reveals that as cells’ response to LNP‐*VEGF‐A* mRNA treatment, several overexpressed proangiogenic transcripts are packaged into EVs. EVs are further deployed to deliver *VEGF‐A* mRNA in vitro and in vivo. Upon equal amount of *VEGF‐A* mRNA delivery via three EV types or LNPs in vitro, EVs from cardiac progenitor cells are the most efficient in promoting angiogenesis per amount of VEGF‐A protein produced. Intravenous administration of luciferase mRNA shows that EVs could distribute translatable mRNA to different organs with the highest amounts of luciferase detected in the liver. Direct injections of *VEGF‐A* mRNA (via EVs or LNPs) into mice heart result in locally produced VEGF‐A protein without spillover to liver and circulation. In addition, EVs from cardiac progenitor cells cause minimal production of inflammatory cytokines in cardiac tissue compared with all other treatment types. Collectively, the data demonstrate that LNPs transform EVs as functional extensions to distribute therapeutic mRNA between cells, where EVs deliver this mRNA differently than LNPs.

## Graphical abstract

The study shows that a fraction of LNP‐mRNA that is cell‐endocytosed can be sent to other cells via the secretion of extracellular vesicles (EVs). LNPs transform these EVs  as functional extensions to distribute therapeutic mRNA between cells.Importantly, EVs can be isolated such as from cardiac progenitor cells (CPC‐EVs), and thus utilized for mRNA delivery in vivo. Upon mRNA delivery to cardiac tissue, CPC‐EVs cause less expression of inflammatory cytokines, compared to other vehicles used.

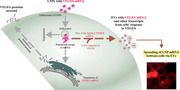

## 1 Introduction

Extracellular vesicles (EVs) are lipid bilayer vesicles that are secreted from almost every cell type and are detected in body fluids and conditioned culture media from living cells. EVs represent a heterogeneous population of vesicles, which may include exosomes (secreted by endosomal/exocytotic pathways), microvesicles (which bud off from plasma membrane), and apoptotic vesicles (generated by cellular disintegration). In 2007, we showed that EVs shuttle endogenous RNA between cells and thus discovered a novel mechanism of exchanging genetic material between cells.^[^ ^1^ ^]^ It has been shown that the RNA‐binding proteins could, in part, contribute to packaging of cytoplasmic RNAs into EVs.^[^ ^2^ ^]^ EVs are reportedly recognized as mediators of intercellular communication,^[^ ^3^, ^4^ ^]^ which can mediate and facilitate local as well as long‐distance functional communication between cells and organs.^[^ ^5^, ^6^ ^]^ The contribution of EVs in cell‐to‐cell communication has been largely recognized due to their inherent properties of transporting endogenous RNA and protein molecules, between cells such as mRNA, microRNA,^[^ ^1^ ^]^ misfolded neurodegenerative proteins,^[^ ^7^ ^]^ and several other biomolecules of exogenous origin. For instance, it has long been thought that viruses hijack the endocytic machinery of host cells^[^ ^8^, ^9^, ^10^, ^11^, ^12^, ^13^ ^]^ and that, after replicating inside the host cell, the viral proteins and RNA fragments are packaged into EVs and egressed through exocytosis (exosomal release pathways), thus spreading to other cells.^[^ ^14^, ^15^, ^16^, ^17^ ^]^ As such, the particles of exogenous origin are packaged into EVs using a natural transport process of cells, i.e., endocytosis/exocytosis.^[^ ^18^ ^]^

It is becoming increasingly evident that EV‐mediated molecular transport plays important roles in several diseases, including cancer,^[^ ^19^, ^20^, ^21^, ^22^, ^23^ ^]^ inflammatory,^[^ ^24^, ^25^ ^]^ neurodegenerative,^[^ ^26^, ^27^, ^28^ ^]^ and cardiovascular^[^ ^29^, ^30^, ^31^, ^32^, ^33^ ^]^ diseases. Lately, it has been recognized that EVs secreted from stem cells actively transport growth factors, paracrine factors, and regulatory RNAs between cells that are involved in angiogenesis and tissue repair.^[^ ^34^, ^35^ ^]^ We and other researchers have shown that EVs can be engineered and loaded with exogenous RNA of interest and applied as vehicles of RNA drug delivery to living cells.^[^ ^18^, ^36^, ^37^, ^38^, ^39^ ^]^

Lipid nanoparticles (LNPs) are clinically approved vehicles of mRNA transport and have recently been utilized as an RNA delivery platform for LNP‐mRNA vaccines against COVID‐19, manufactured by Pfizer‐BioNTech and Moderna,^[^ ^40^, ^41^ ^]^ and their applications are being explored beyond mRNA vaccines.^[^ ^42^, ^43^ ^]^ LNPs have also been applied for the co‐delivery of siRNA and mRNA,^[^ ^44^ ^]^ and for the delivery of therapeutic mRNA in different disease models in vivo,^[^ ^45^, ^46^ ^]^ including clinical trials of immunogenicity for protection against Zika and influenza viruses.^[^ ^47^, ^48^ ^]^ Several studies have demonstrated that LNPs enter cells via endocytosis and accumulate in endolysosomal compartments.^[^ ^49^, ^50^, ^51^, ^52^, ^53^, ^54^ ^]^ Exogenous delivery of angiogenic *VEGF‐A* mRNA to ischemic tissues for the induction of neovascularization is considered a promising strategy for the treatment of cardiovascular diseases. However, the collaborative roles of LNPs and EVs in transport of *VEGF‐A* have not been explored. The *VEGF‐A* mRNA has been shown to improve cardiac function after myocardial infarction in preclinical models.^[^ ^55^, ^56^ ^]^ Recently, naked *VEGF‐A* mRNA in a citrate saline solution (without LNPs as RNA carriers) was applied in clinical trials in patients with type 2 diabetes,^[^ ^57^ ^]^ and patients undergoing coronary artery bypass grafting (CABG).^[^ ^58^, ^59^, ^60^ ^]^ However, currently there is no known effective and safe carrier to deliver therapeutic *VEGF‐A* mRNA to the heart which could potentially stimulate *VEGF‐A*‐dependent blood vessel formation. Therefore, we examined LNP vehicles and EVs for the delivery of functional *VEGF‐A* mRNA to heart‐specific cell line such as cardiac progenitor cells (CPCs), and an angiogenic cell model such as human umbilical vein endothelial cells (HUVECs), and a random epithelial cell model such as human lung epithelial HTB‐177 cells. The delivery of *VEGF‐A* mRNA via vehicles was also compared with naked *VEGF‐A* mRNA in citrate buffer.

Using LNPs we delivered different mRNA molecules to above mentioned cells, including mRNA encoding VEGF‐A protein. We studied cellular uptake kinetics of LNPs and their stochiometric quantification, intracellular metabolism of internalized LNP‐mRNA. Upon delivery of *VEGF‐A* mRNA to cells via LNPs, we discovered that LNP‐*VEGF‐A* mRNA molecules, which are taken up by cells, might have not been fully degraded by recipient cells or not fully translated into protein. But a fraction of intact LNP‐*VEGF‐A* mRNA is sent to other cells via secretion of EVs (which are further taken up by other cells). Transcriptomic analysis of three EV types used in this study demonstrated a robust response to LNP‐*VEGF‐A* treatment, and the several overexpressed proangiogenic transcripts are packaged into EVs and secreted outside of cells. The presence of overexpressed proangiogenic transcripts in EVs might be advantageous over LNPs for heart tissue, as LNPs delivered only one angiogenic mRNA type (*VEGF‐A*), but EVs had acquired angiogenic *VEGF‐A* mRNA and several other angiogenic transcripts. Importantly, when an equal amount of *VEGF‐A* mRNA was transferred to cells via LNPs or EVs, the EVs delivered differently than LNPs which is reflected, at least, from VEGF‐A protein levels, angiogenesis, and inflammatory response.

Our results showed that these co‐opted EVs not only distribute the LNP *VEGF‐A* mRNA to cells but also remain intact and cause the production of VEGF‐A protein both in vitro and in vivo, thus serving as functional extensions of LNP‐mRNA. The cardiac progenitor cells were the most efficient in promoting angiogenesis per amount of VEGF‐A protein produced, in vitro. Importantly, EVs secreted from cardiac progenitor cells caused minimal production of inflammatory cytokines in cardiac tissue compared with all other treatment types used in this study. This suggests that cardiac progenitor EVs might be candidates of safer vehicles for mRNA drug delivery to heart and warrants new investigations to identify EVs that are customized for mRNA delivery to heart or other organs of interest. Since LNP‐based mRNA‐therapeutics have been advanced in human clinical trials, the data from this study also suggests that similar process may occur in humans where part of the LNP‐mRNA distribution between cells or organs might take place via EVs, and this warrants more investigations.

## 2 Results

### 2.1 Kinetics of Cellular Uptake of Lipid Nanoparticles and Their mRNA

**Figure 1:**
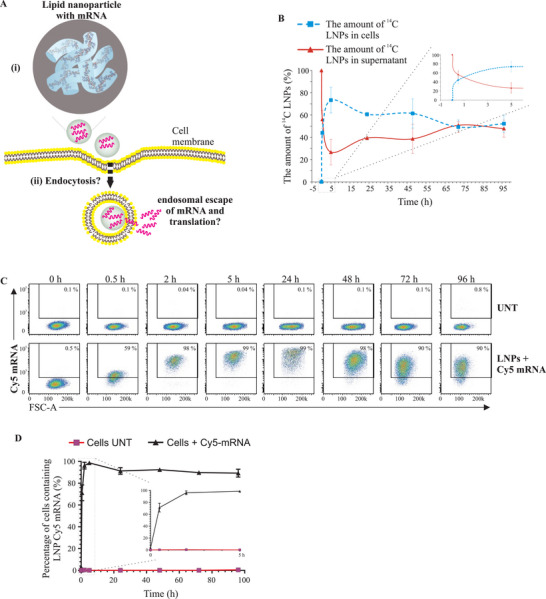
Kinetics of cellular uptake of lipid nanoparticles (LNPs) and LNP‐mRNA. A) Schematic representation of LNP‐mRNA delivery, cellular uptake of LNPs and their mRNA. Part (i) represents an inverse hexagonal phase of MC3‐LNPs loaded with mRNA. Such MC3‐LNP orientation was initially proposed by Arteta et al.^[^ ^61^ ^]^ Part (ii) represents the intracellular fate of LNP‐mRNA investigated in the current study. B) Cellular uptake of LNPs. LNP hexadecyl cholesterol ester was ^14^C‐radiolabeled and delivered to HTB‐177 cells. LNP uptake kinetics were determined by measuring the relative radioactivity of ^14^C‐labeled LNPs at different time points using a microbeta trilux scintillator (*n* = 3). C) Flow cytometry analysis showing the percentage of cells that were positive for fluorescently labeled Cy5 *eGFP* mRNA, quantified at different time points to examine the cellular uptake kinetics of LNPs (*n* = 3). D) Quantitative data showing the percentage of cells containing Cy5‐*eGFP* mRNA at different time points after LNP delivery. A box with zoomed in part represents the data from 0 to 5 h. UNT, untreated.

The LNPs used in this study (DLin‐MC3‐DMA LNPs, referred to as MC3‐LNPs) consisted of four lipid components (ionizable lipid:cholesterol:DSPC:DMPE‐PEG2000) and were either loaded with fluorescently labeled mRNA or translatable *VEGFA* mRNA. **Figure** **1**A‐i shows an inverse hexagonal phase of mRNA loaded MC3‐LNPs used in this study, initially reported by Arteta et al.^[^ ^61^ ^]^ The chemical structure of ionizable MC3‐LNPs and their characterization are presented in Figure S1A–C and Table S1 (Supporting Information). It has been established that LNPs enter cells predominantly via endocytosis and accumulate in the endolysosomal compartments.^[^ ^49^, ^50^, ^51^, ^52^, ^53^, ^54^ ^]^ To examine the cellular uptake of LNPs, they were either radiolabeled (with ^14^C of the hexadecyl cholesterol ester) or encapsulated with fluorescently labeled mRNA (Cy5‐*eGFP* mRNA) or translatable *VEGF‐A* mRNA and were delivered to HTB‐177 cells (Figure 1A‐ii).

The cellular uptake kinetics of LNPs were studied by measuring the relative amount (%) of ^14^C‐labeled LNPs in the extracellular environment, i.e., in the supernatants at different time points. The results show that cellular uptake of LNPs occurred quickly. Within 5 h, approximately 80% of ^14^C‐labeled LNPs had disappeared from the cell culture supernatants, i.e., were consumed by cells (Figure 1B). Between 24 and 96 h, the amount of ^14^C‐labeled LNPs started increasing in the supernatant, indicating that cells might secrete the LNPs into the extracellular environment.

Then, cellular uptake kinetics of mRNA of LNPs were investigated by detecting intracellular LNP‐Cy5‐mRNA at different time points. The uptake of LNP‐Cy5‐mRNA by cells was similar to the uptake of ^14^C‐LNPs in that the LNP‐mRNA was internalized quickly. Flow cytometry analysis of internalized LNP‐Cy5‐mRNA at different time points showed that after 2 h, most of the cells (>90%) were positive for Cy5‐mRNA (Figure 1C,D).

### 2.2 LNPs Successfully Deliver the Translatable VEGF‐A mRNA to Epithelial, Endothelial, and Cardiac Progenitor Cells

*VEGF‐A* mRNA encodes VEGF‐A protein, a secretory growth factor involved in blood vessel formation and repair of heart injuries. Previously, naked *VEGF‐A* mRNA in citrate saline solution (without LNPs as carriers) has been applied in clinical trials in patients with cardiovascular disease,^[^ ^58^, ^60^ ^]^ and type 2 diabetes.^[^ ^57^ ^]^ However, currently, there is no safe carrier that can deliver *VEGF‐A* mRNA to the heart. In the current study, *VEGF‐A* mRNA was delivered using LNPs as carriers in three different cell types: CPCs, HUVECs, and HTB‐177 cells.

**Figure 2:**
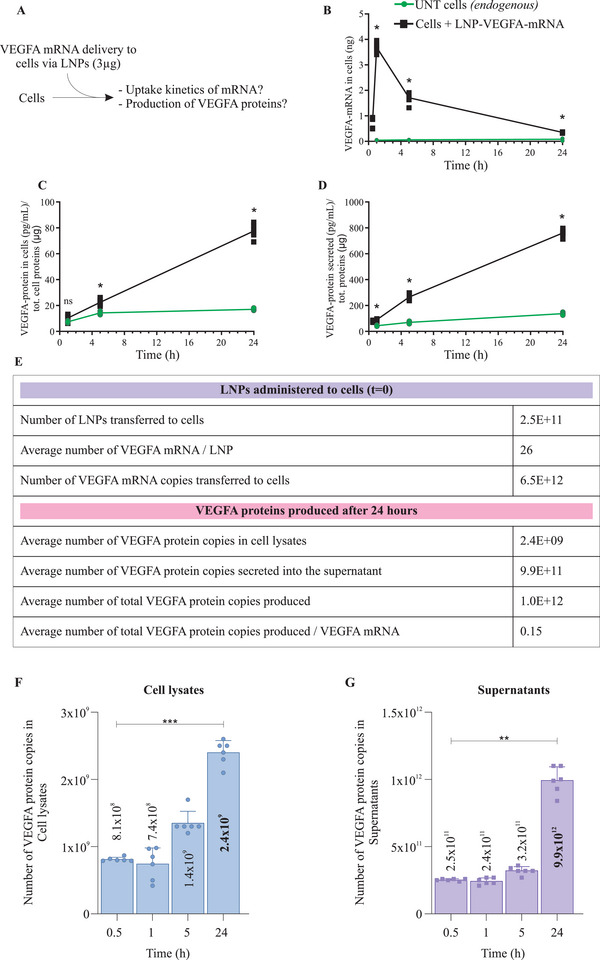
Delivery of *VEGF‐A* mRNA via Lipid Nanoparticles (LNPs) and kinetics of cellular uptake of mRNA. A) A schematic representation of cellular delivery of LNPs containing chemically modified *VEGF‐A* mRNA, and uptake kinetics of LNP‐mRNA. A 3 µg of *VEGF‐A* mRNA was delivered via LNPs. B) Detection and quantification of LNP‐*VEGF‐A* mRNA inside HTB‐177 cells at different time points (*n* = 6). C) Detection and quantification of newly produced VEGF‐A protein in cell lysate, following the delivery of 3 µg *VEGF‐A* mRNA via LNPs (*n* = 6). D) Levels of VEGF‐A protein secreted into the extracellular space (detected in supernatants, *n* = 6). For B–D), the Mann‐Whitney U‐test was applied to compare untreated and treated samples for each time point, separately. Significant differences (*p < 0.05) between LNP‐treated and untreated cells were observed at 1 h, 5 h, and 24 h. ns = no significant differences. E) Stoichiometric quantification of total numbers of LNPs, total *VEGF‐A* mRNA copies administered to cells (time t0), and the number of *VEGF‐A* mRNA copies delivered per LNP (time t0). After 24 h of LNP delivery, the VEGF‐A protein copies produced from delivered LNP‐*VEGF‐A* mRNA were calculated (*n* = 6). F) Copy numbers of VEGF‐A protein quantified in cell lysates and G) in supernatants of HTB‐177 cells treated with LNP‐*VEGF‐A* mRNA (*n* = 6). Statistically significant differences for cells and supernatants were separately evaluated by the Kruskal‐Wallis test followed by Dunn's multiple comparison test to 0.5 h (**p < 0.01, ***p < 0.001; blank = no significant differences). UNT, untreated.

*VEGF‐A* mRNA encapsulated in LNPs was administered to HTB‐177 cells, and the cellular uptake kinetics, and translation of exogenous mRNA into VEGF‐A protein were examined (**Figure** **2**A–D). First, for each time point, qPCR was performed on cellular RNA using a specific probe against *VEGF‐A* mRNA. A significantly elevated amount of *VEGF‐A* mRNA was detected in cells within 1 h after the delivery of LNP‐*VEGF‐A* mRNA, compared to untreated cells (Figure 2B). However, the intracellular levels of *VEGF‐A* mRNA started decreasing after 1 h. Furthermore, examination of VEGF‐A protein in LNP‐treated cells showed that exogenously delivered LNP‐*VEGF‐A* mRNA was translated into VEGF‐A protein and was also secreted into the supernatants of these cells. The levels of VEGF‐A protein produced from exogenous *VEGF‐A* mRNA were significantly higher than endogenous VEGF‐A protein produced by untreated cells (Figure 2C,D). The quick disappearance of ^14^C‐LNPs from the cultured media, the highest levels of LNP‐*VEGF‐A* mRNA in cells at early time points, and the relative time‐dependent increase in VEGF‐A protein levels were in line with that the cells take up LNPs quickly and the translation of LNP‐delivered mRNA starts immediately ( Figure S2A, Supporting Information).

#### 2.2.1 Stoichiometric Analysis of Uptaken LNP‐VEGF‐A mRNA and Protein in Recipient Cells

A stoichiometric quantification was performed for total LNPs administered (t0), total *VEGF‐A* mRNA copies delivered by total LNPs (t0), number of *VEGF‐A* mRNA copies delivered per LNP (t0), and VEGF‐A protein copies produced after 24 h. The VEGF‐A protein copies in cells, secreted in supernatants and protein produced per LNP‐*VEGF‐A* mRNA were quantified and presented in (Figure 2E). Further stoichiometric quantification showed that a maximum number of VEGF‐A protein copies were produced at 24 h and the secreted protein copies were higher in supernatants than in their cell lysates (Figure 2F,G). From 6.5 × 10^12^ *VEGF‐A* mRNA copies delivered to cells, on average 1×10^12^ VEGF‐A protein copies were detected at 24 h (sum of lysate and supernatants). Apparently, one copy of *VEGF‐A* mRNA could produce 0.15 copy of VEGF‐A protein, indicating that not all the mRNA delivered via LNPs is translated into protein. However, it should be noted that this number does not represent the remaining *VEGF‐A mRNA*, which had not escaped endosomes for translation into protein at that time point, and the mRNA which had degraded by lysosomal degradation, or the amount already egressed.

#### 2.2.2 LNPS Can Deliver Translatable VEGF‐A mRNA to Cardiac Progenitor Cells and Human Umbilical Vein Endothelial Cells

**Figure 3:**
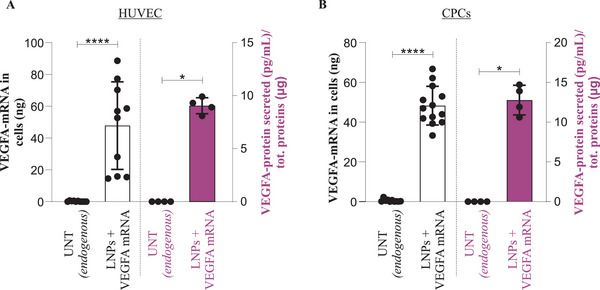
Delivery of translatable *VEGF‐A* mRNA to human umbilical vein endothelial cells and cardiac progenitor cells via lipid nanoparticles (LNPs). A 3 µg of *VEGF‐A* mRNA was delivered to human umbilical vein endothelial cells (HUVECs) and cardiac progenitor cells (CPCs) via LNPs. The levels of *VEGF‐A* mRNA and protein were quantified 24 h post LNP administration. A) Detection of LNP‐*VEGF‐A* mRNA in HUVECs (left side, Y‐axis) and its translation into VEGF‐A protein (right side, Y‐axis). The Mann‐Whitney U‐test was applied to compare the LNP treated and untreated samples. *VEGF‐A* mRNA (*n* = 10, for untreated, and treated), ***p < 0.001. VEGF‐A protein (*n* = 4, for untreated, and treated), *p < 0.05. B) Detection of LNP‐*VEGF‐A* mRNA in CPCs (left side, Y‐axis), and its translation into VEGF‐A protein (right side, Y‐axis). The Mann‐Whitney U‐test was applied to compare the LNP‐treated and untreated samples. *VEGF‐A* mRNA (*n* = 10 for untreated, *n* = 13 for treated), ****p < 0.0001. VEGF‐A protein (*n* = 4, for untreated, and treated), *p < 0.05.

We further examined whether LNP‐*VEGF‐A* mRNA could also be internalized by other cell types, particularly those that participate in heart function and angiogenesis. For instance, CPCs and HUVECs were treated with LNP‐*VEGF‐A* mRNA. The significantly elevated levels of *VEGF‐A* mRNA were detected in both cell types after treatment of LNPs, compared to untreated cells (left side, Y‐axis **Figure** **3**A,B). The internalized LNP‐*VEGF‐A* mRNA was also translated into VEGF‐A protein and secreted into the extracellular environment/supernatants of both cell types, compared to untreated cells (right side, Y‐axis Figure 3A,B).

#### 2.2.3 LNP‐mRNA Delivery and Processing Can be Cell Type Dependent

Differences in the intracellular *VEGF‐A* mRNA levels after the delivery of equal amounts (3 µg) of LNP‐*VEGF‐A* mRNA to three different cell types showed that the uptake of LNPs and *VEGF‐A* mRNA translation appears to be cell type dependent. The cellular retention of *VEGF‐A* mRNA was higher in HUVECs and CPCs than in HTB‐177 cells at the studied time point (24 h) (Figure 3A,B ≈40 ng, compared with Figure 2B ≈1 ng). HTB‐177 cells apparently processed LNP‐*VEGF‐A* mRNA efficiently, i.e., mRNA translation (VEGF‐A protein) was higher in HTB‐177 cells than in HUVECs and CPCs (Figure 2C,D ≈800 pg mL^‐1^, compared with Figure 3A,B < 25 pg mL^‐1^).

### 2.3 mRNA of Internalized LNPs Is Secreted into EVs

**Figure 4:**
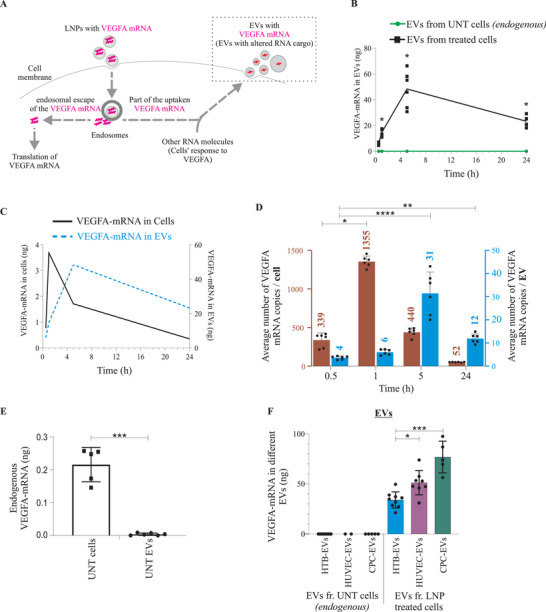
*VEGF‐A* mRNA of lipid nanoparticles (LNPs) is secreted into extracellular vesicles (EVs). A) Schematic illustration for the investigation of the intracellular fate of LNP‐*VEGF‐A* mRNA and secretion of *VEGF‐A* mRNA into EVs. B) Detection and quantification of *VEGF‐A* mRNA in EVs secreted from LNP‐treated HTB‐177 cells (exogenous), and untreated cells (endogenous) (*n* = 6). C) Overlapping and time‐lapsed analysis of the intracellular detection of LNP‐*VEGF‐A* mRNA, with relation to its incorporation into EVs (*n* = 6). D) Number of *VEGF‐A* mRNA copies quantified in EVs and their parental cells at different time points (*n* = 6). Using LNPs, cells were treated with 3 µg (6.5 × 10^12^ copies) of *VEGF‐A* mRNA which was loaded in 2.5 × 10^11^ LNPs (i.e., 26 copies per LNP). On average 1355 *VEGF‐A* mRNA copies per cell (after 1 h, the peak), and 31 *VEGF‐A* mRNA copies/EV (after 5 h, the peak) were detected. Out of 6.5 × 10^12^ copies of *VEGF‐A* mRNA administered via LNPs to cells, only 2186 and 53 *VEGF‐A* mRNA copies per cell and EV, respectively, were detected. Statistically significant differences over time for cells and EVs were evaluated separately by the Kruskal‐Wallis test followed by Dunn's multiple comparison test to 0.5 h (*p < 0.05, **p < 0.01, ****p < 0.0001; ns = no significant differences). E) Levels of endogenous *VEGF‐A* mRNA in untreated cells and their secreted EVs (*n* = 5 cells, *n* = 8 EVs). Statistically significant differences between cells and EVs were evaluated by the Mann‐Whitney U‐test (***p < 0.001). F) Comparison of LNP‐*VEGF‐A* mRNA in EVs secreted from HTB cells (*n* = 10 for UNT (untreated), *n* = 9 for treated), HUVECs (*n* = 2 for UNT, *n* = 8 for treated), and CPCs (*n* = 4 for UNT, *n* = 5 for treated). Statistically significant differences in treated cells were evaluated by the Kruskal‐Wallis test followed by Dunn's multiple comparison test (*p < 0.05; ***p < 0.001; ns = no significant differences). Dots in the plots represent the distribution of individual samples. UNT EVs, EVs from untreated cells.

Previous studies have shown that LNPs are taken up by cells via endocytosis, and a limited amount of LNP‐RNA is detected in the cytosol due to endocytic recycling and inadequate endosomal escape.^[^ ^49^, ^50^, ^51^ ^]^ Thus, we investigated whether a part of endocytosed LNP‐mRNA was secreted into EVs—a cell's natural secretory process (**Figure** **4**A). HTB‐177 cells were treated with *VEGF‐A* mRNA loaded LNPs, and EVs were isolated from LNP treated cells. *VEGF‐A* mRNA in EVs secreted from treated and untreated cells was examined by qPCR. Significantly higher amounts of *VEGF‐A* mRNA were detected in EVs of cells, which had taken up LNPs (Figure 4B). Notably, while LNP‐*VEGF‐A* mRNA was detected in cells within 1 h, the *VEGF‐A* mRNA in EVs was detected only a few hours later, indicating that LNP‐mRNA is first internalized, processed inside cells and then secreted (Figure 4C).

Additionally, stoichiometric analysis was performed to calculate the numbers of *VEGF‐A* mRNA copies detected in EVs and their parental cells. The maximum number of mRNA copies (1355 *VEGF‐A* mRNA copies/cell) was detected 1 h after the delivery of LNP‐*VEGF‐A* mRNA to cells. However, at the same time point, there were only 6 *VEGF‐A* mRNA copies/EV. The maximum number of *VEGF‐A* mRNA copies detected in EVs was only after 5 h (31 *VEGF‐A* mRNA copies/EV) (Figure 4D). Total number of cells at each time point and their secreted EVs at corresponding time point are shown in Figure S2B (Supporting Information). The detailed numbers of EVs from individual samples for each time point are presented in Figure S3 (Supporting Information).

Cells secrete EVs continuously, whereby EVs not only are secreted in large numbers but also are accumulated in the supernatants. Thus, collective total *VEGF‐A* mRNA in total accumulated EVs is apparently high. However, the numbers of *VEGF‐A* mRNA copies per single EV shows that, compared with copies per single EV, there were 225‐ and 14‐fold more *VEGF‐A* mRNA copies per cell at 1 h and 5 h, respectively (Figure 4D). This showed that an individual EV contains far less mRNA copies than an individual cell, and the single cell can secret the same mRNA in several different EVs.

A total of 6.5 × 10^12^ copies of *VEGF‐A* mRNA were administered to cells via LNPs, whereas the total number of *VEGF‐A* mRNA copies detected in EVs was far less than the number of delivered mRNA copies. From the 6.5 × 10^12^ copies of LNP delivered *VEGF‐A* mRNA, only 53 and 2186 *VEGF‐A* mRNA copies were detected per EV and per cell, respectively, in total (sum of copies from all time points) (Figure 4D). This shows that only a tiny fraction of endocytosed LNP‐mRNA is secreted into EVs. Cells that were not treated with LNP‐*VEGF‐A* mRNA expressed a negligible amount of endogenous *VEGF‐A* mRNA, and likewise EVs secreted from untreated cells did not contain endogenous *VEGF‐A* mRNA (Figure 4E).

EVs were also isolated from other cell types (HUVECs and CPCs), which were treated with LNP‐*VEGF‐A* mRNA, and the presence of *VEGF‐A* mRNA in EVs was examined by qPCR. *VEGF‐A* mRNA was detected in EVs secreted after LNP treatment (Figure 4F). Upon the delivery of an equal amount of LNP‐*VEGF‐A* mRNA (3 µg) to three different cell types, the levels of *VEGF‐A* mRNA in EVs indicated that the packaging of LNP‐mRNA into EVs is cell type dependent. The *VEGF‐A* mRNA levels in EVs secreted from HTB‐177 cells were significantly lower than those in EVs secreted from HUVECs and CPCs (Figure 4F). These findings reveal cell‐type‐dependent differences in mRNA uptake and incorporation into EVs despite an equal amount of LNP‐mRNA was delivered.

### 2.4 Characterization of EVs Secreted from LNP‐Treated Cells

#### 2.4.1 Detection of LNP‐mRNA in CD63‐Positive EVs

**Figure 5:**
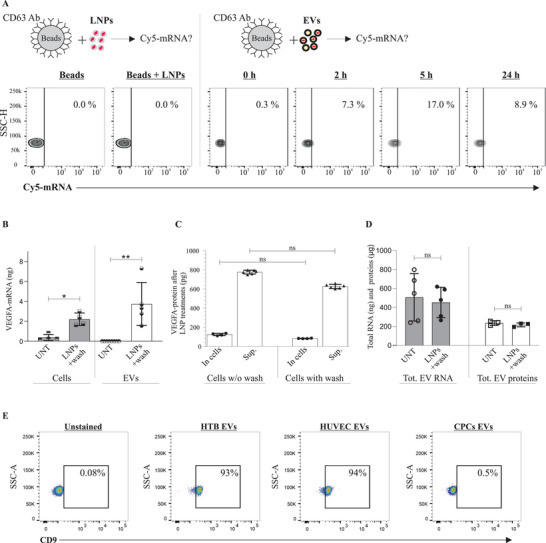
Characterization of extracellular vesicles (EVs) and validation of LNP‐mRNA Incorporation into EVs. A) Flow cytometry analysis showing the percentage of purified CD63^+^ EVs containing Cy5‐*eGFP* mRNA isolated from LNP‐treated cells, at different time points (*n* = 2). One representative measurement is shown from each time point. LNP‐Cy5‐*eGFP* mRNA + beads were used instead of EVs+beads, to evaluate whether a contamination had occurred. For the negative control, only beads (without EVs or LNPs) were used. B) Detection of LNP‐*VEGF‐A* mRNA in cells and their secreted EVs, after washing out the LNPs (*n* = 6 for LNP‐treated and UNT (untreated) cells, *n* = 5 for treated EVs and *n* = 8 for UNT EVs). Statistically significant differences were evaluated by the Mann‐Whitney U‐test (*p < 0.05; **p < 0.01). C) Detection of VEGF‐A protein in cell lysates and supernatants, after washing out LNPs. The Kruskal‐Wallis test followed by Dunn's multiple comparison test was used to compare washed versus unwashed supernatants and washed versus unwashed cells (ns = no significant differences). D) The amounts of total EV‐RNA and total EV‐proteins between washed and unwashed cells from LNP‐treated or untreated groups (*n* = 6). Statistically significant differences were evaluated by the Mann‐Whitney U‐test (ns = no significant differences). E) Detection of CD63, and CD9‐positive HTB‐, HUVEC‐, and CPC‐EVs. Pre‐enriched EVs were captured by anti‐CD63‐antibody‐conjugated beads, and then these CD63^+^ EVs were captured/isolated with PE‐CD9 antibody and acquired on a BD FACSLyric system (BD Biosciences). Results show that EVs that were CD63‐positive were also CD9‐positive. Data were analyzed using FlowJo software (TreeStar Inc.). As a negative control, CD63‐antibody‐conjugated beads alone (without EVs) were incubated with an equivalent volume of PBS. The experiment was performed in biological duplicates (*n* = 2). One representative measurement from each EV type is shown.

Lysosome‐associated membrane protein 3 (LAMP‐3), commonly known as CD63, is a marker of EVs. We examined whether EVs isolated from LNP‐mRNA‐treated cells are CD63‐positive (CD63^+^) and whether they also contain LNP‐mRNA after they are secreted from LNP‐treated cells. EVs from LNP‐mRNA‐treated cells were captured by magnetic dynabeads conjugated with anti‐CD63 antibody and examined by flow cytometry. The CD63^+^ EVs secreted from cells treated with LNP‐Cy5‐mRNA contained Cy5‐mRNA (**Figure** **5**A). To examine the nonspecific binding of LNP‐Cy5‐mRNA with beads, the LNPs were processed with CD63‐antibody‐conjugated beads. The Cy5‐mRNA was not detected in LNP samples and negative controls (beads only).

#### 2.4.2 Detection of LNP‐mRNA in EVs after Washing Out LNPs

As our results showed that within the first 2 h of LNP administration, >90% of cells were positive for LNP‐mRNA and the peak of translatable *VEGF‐A* mRNA in cells was observed within 1 h of LNP administration. Any LNPs that had not been taken up by cells and EVs secreted during this period (within 1 h) were removed; cells were washed with PBS and supplemented with fresh media. In the fresh media of washed cells, no LNP‐mRNA remained outside of cells to interact with EVs. The results showed that there was a significant amount of *VEGF‐A* mRNA in washed cells and newly secreted EVs (which, indeed, were secreted after washing the cells) (Figure 5B). This confirms that the majority of LNP‐*VEGF‐A* mRNA was already taken up by cells (before washing), and part of this mRNA was secreted into newly produced EVs from cells that had taken up LNP‐mRNA before washing. Additionally, the amount of VEGF‐A protein did not significantly differ between washed and unwashed cells with or without LNP treatment (Figure 5C). This also confirms that the majority of LNP‐*VEGF‐A* mRNA was already taken up by cells before washing, otherwise the levels of VEGF‐A protein would differ significantly between washed and unwashed groups. Additionally, no quantifiable differences in total EV‐RNA and total EV‐proteins were observed between the washed and unwashed cells with or without LNP treatment (Figure 5D).

#### 2.4.3 Examination of EV Morphology

For morphological analysis, the isolated EVs were fixed and examined with a transmission electron microscope. EVs isolated from all three cell lines (HTB‐EVs, HUVEC‐EVs, and CPC‐EVs) were round, and their outer rims appeared dense, representing the outer membrane of EVs. The cropped areas are shown at the top right of each panel, where black arrows represent the cropped area (Figure S4, Supporting Information).

#### 2.4.4 Detection of EV Markers in EVs from Three Cell Types

Following the characterization and detection of LNP‐mRNA in CD63^+^ EVs, another EV‐specific marker, such as CD9, was investigated in HTB‐, HUVEC‐, and CPC‐EVs. To identify whether CD63^+^ EVs were also positive for CD9, EVs that were selectively captured by the anti‐CD63 antibody (i.e., CD63^+^ EVs) were further immunostained with a mouse anti‐human PE‐CD9 antibody and analyzed by flow cytometry. The results showed that EVs isolated from HUVECs, and HTB‐177 cells were positive for both CD63 and CD9 (captured with anti‐CD63 and identified with PE‐anti‐CD9) (Figure 5E). However, the EVs isolated from CPCs were only positive for CD63 and might lack or express a very low levels of CD9 (only 0.5% of CD63^+^ EVs contained CD9).

#### 2.4.5 Determination of Size and Concentration of EVs

The sizes and concentrations of EVs isolated 0.5, 1, 5, and 24 h after LNP treatment were determined by Nanoparticle Tracking Analysis. At the studied time points, the mean (± SEM) diameters of secreted EVs were distributed between 92 and 126 nm, and the mean (± SEM) concentrations ranged from 28.30 × 10^8^ (±4.07 × 10^7^) to 5.45 × 10^9^ (±7.73 × 10^7^) particles mL^‐1^. The datasets from each time point are presented in Figure S3 (Supporting Information). Additionally, the sizes of EVs isolated by size exclusion chromatography (SEC‐EVs) were determined by Zetasizer Nano. The size distribution by intensity is shown in Figure S5A (Supporting Information).

#### 2.4.6 Examination of VEGF‐A mRNA and Protein in EV Fractions Isolated by Size Exclusion Chromatography

A control experiment was performed using size exclusion chromatography (SEC) to examine whether *VEGF‐A* mRNA is detected in EV fractions, but not in free form in non‐EV fractions. EVs were purified by SEC columns, and *VEGF‐A* mRNA in the EV fractions (F1–4) was quantified. The VEGF‐A protein was also examined in these fractions. The qPCR data showed that the purified EVs contained *VEGF‐A* mRNA in fraction 1–4 (which represent EVs). The purified EV fractions were enriched in *VEGF‐A* mRNA, but not enriched in VEGF‐A protein. only a small amount of VEGF‐A protein was detected (Figure S5B, Supporting Information).

### 2.5 EVs Secreted from LNP‐mRNA Treated Cells Further Transport the mRNA between Cells

**Figure 6:**
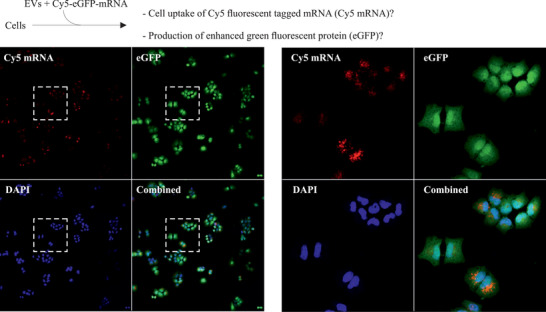
Extracellular vesicles (EVs) can extend the LNP‐mRNA delivery between cells. EVs secreted from LNP‐Cy5‐*eGFP* mRNA treated cells contained Cy5‐*eGFP* mRNA. These EVs containing translatable Cy5‐*eGFP* mRNA were delivered to recipient HTB‐177 cells. The cellular uptake of EV‐Cy5‐*eGFP* mRNA (red) and its translation into eGFP (green) after EV delivery were detected by confocal microscopy (*n* = 3). One representative image is shown. The right‐side panels represent the cropped areas.

We further investigated i) whether EVs could deliver this Cy5‐*eGFP* mRNA to other cells and ii) whether the *eGFP* mRNA was translated into eGFP. EVs containing translatable Cy5‐*eGFP* mRNA were delivered to HTB‐177 cells, in which the cellular uptake of EV Cy5‐*eGFP* mRNA and the production of eGFP was examined. Confocal microscopy analysis revealed that EV‐*eGFP* mRNA was taken up by cells and translated into eGFP (**Figure** **6**). Cells that did not receive EVs were used as controls, and no Cy5‐*eGFP* mRNA or eGFP was detected in untreated cells (Figure S6, Supporting Information).

### 2.6 EVs Can Deliver Functional Exogenous VEGF‐A mRNA to Target Cells

**Figure 7:**
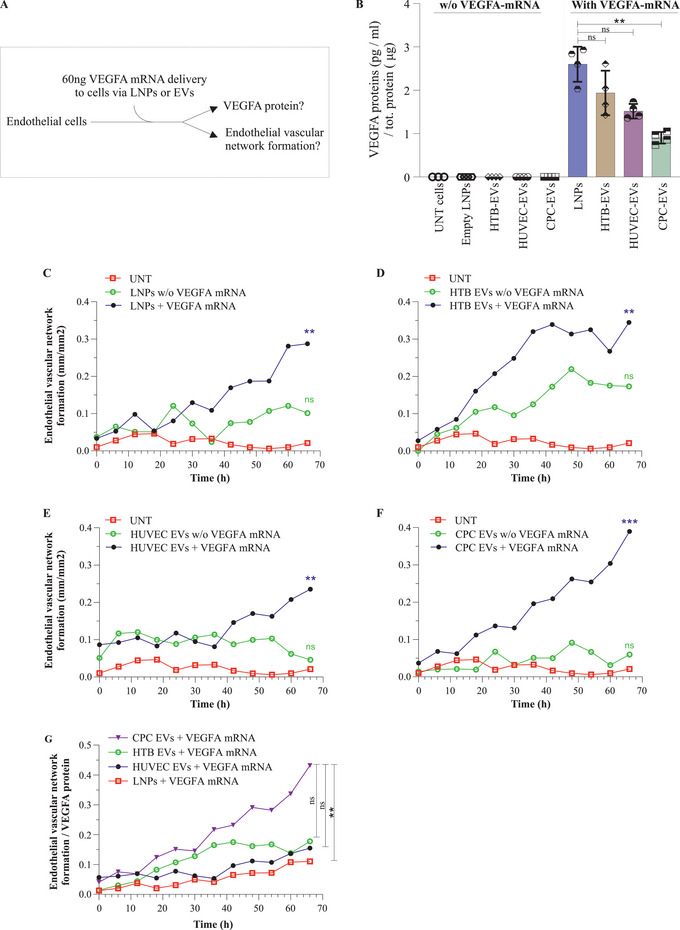
Extracellular vesicles (EVs) can functionally deliver. The *VEGF‐A* mRNA of LNPs. EVs secreted from LNP‐*VEGF‐A* mRNA treated cells were isolated, which contained *VEGF‐A* mRNA and were further delivered to recipient cells examine the functionality. A) *VEGF‐A* mRNA (60 ng) was delivered to HUVECs via EVs or lipid nanoparticles (LNPs), and the production of VEGF‐A protein and vascular networks were quantified. B) Quantification of VEGF‐A protein produced after delivery of *VEGF‐A* mRNA via EVs or LNPs (*n* = 4). Statistically significant differences were evaluated by the Friedman test followed by the Dunn's multiple comparison test to compare LNPs + *VEGF‐A* mRNA with EVs + *VEGF‐A* (**p < 0.01; ns = no significant differences). C–F) Endothelial vascular network formation after delivering equal quantity (60 ng) of *VEGF‐A* mRNA via LNPs or three different EV types. Data at each point represents the mean of seven replicates (*n* = 7 treated, *n* = 3 untreated). For C–F), the Kruskal‐Wallis test followed by the Dunn's multiple comparison test was applied to compare the samples of *VEGF‐A* mRNA, with samples of without *VEGF‐A* mRNA and untreated, at 66 h. (**p < 0.01; ns = no significant differences). G) The efficiency of functional *VEGF‐A* mRNA delivery by measuring endothelial network formation per VEGF‐A protein produced upon delivery of equal amounts (50 ng) of *VEGF‐A* mRNA to cells via LNPs or three different EVs. Data at each point represents the mean of seven replicates (*n* = 7 treated, *n* = 3 untreated). The Kruskal‐Wallis test followed by the Dunn's multiple comparison test was applied to compare CPC + *VEGF‐A* mRNA, with LNP‐, HTB‐, HUCEC‐, (+ *VEGF‐A* mRNA) at 66 h (**p < 0.01; ns = no significant differences). UNT, untreated; w/o, without.

We next investigated whether EVs can transport functional *VEGF‐A* mRNA to cells and whether their delivery differs from that of LNPs. First, qPCR was performed on EV‐*VEGF‐A* mRNA, and an equal quantity (60 ng) of *VEGF‐A* mRNA was delivered to HUVECs via three different EV types or LNPs; the readouts were compared in vitro (**Figure** **7**A). In spite of the equal amount of *VEGF‐A* mRNA delivered (60 ng), the EVs and LNPs differed in their delivery in terms of VEGF‐A protein levels produced (Figure 7B). However, endogenously expressed VEGF‐A protein was detected in negligible amounts in untreated cells, and the cells treated with empty LNPs or EVs without *VEGF‐A* mRNA.

Next, we investigated whether the newly produced VEGF‐A protein (EV‐mediated delivery of *VEGF‐A* mRNA) was functional and could initiate endothelial cell proliferation and angiogenesis. EVs containing *VEGF‐A* mRNA were delivered to endothelial cells. The cell number and size of nuclei were increased approximately twofold after EV delivery of *VEGF‐A* mRNA to HUVECs, compared with untreated cells or cells treated with EVs secreted from untreated cells (i.e., EVs which did not carry *VEGF‐A* mRNA) (Figure S7A–C, Supporting Information). In addition, EV‐mediated delivery of *VEGF‐A* mRNA in vitro, induced network formation compared with untreated cells (Figure S7D, Supporting Information).

Furthermore, the delivery of *VEGF‐A* mRNA via three different EV types induced and prolonged network formation analyzed up to 66 h (Figure 7C–F). Interestingly, delivery of *VEGF‐A* mRNA via CPC‐EVs and LNPs produced the lowest and highest levels of VEGF‐A protein, respectively. LNPs produced approximately threefold more VEGF‐A protein, compared with CPCs (Figure 7B). However, when the efficiency of functional mRNA delivery was quantified by measuring the tube formation per VEGF‐A protein produced, the length of tube formed was the highest with CPC‐EVs and the lowest with LNPs (Figure 7G). This shows that despite equal amounts of *VEGF‐A* mRNA delivered to cells via three different EVs or LNPs, the CPC‐EVs were the most efficient in promoting angiogenesis per amount of VEGF‐A protein produced (Figure 7G).

The effects of endogenous *VEGF‐A* mRNA delivered via untreated EVs to HUVECs were also investigated. EVs isolated from untreated cells (endogenous *VEGF‐A* mRNA) and from LNP‐treated cells (exogenous *VEGF‐A* mRNA) were delivered to HUVECs, and the levels of VEGF‐A protein were quantified in the recipient cells. Delivery of untreated EVs (endogenous *VEGF‐A* mRNA) exerted no effects on VEGF‐A protein levels in recipient cells ( Figure S8, Supporting Information). However, the delivery of EVs which contain exogenous *VEGF‐A* mRNA, produced significantly higher amount of VEGF‐A protein (≈300‐fold). This confirms that VEGF‐A protein in recipient cells is produced from exogenously delivery *VEGF‐A* mRNA, and endogenous *VEGF‐A* mRNA has no effects on data interpretation.

### 2.7 Transcriptional Analysis of Altered EVs Secreted from LNP Treated Cells

Since in vitro delivery of equal amounts of *VEGF‐A* mRNA via three different EV types demonstrated different outcomes, we characterized the total mRNA content of EVs, which were used for in vitro delivery. The transcriptome of EVs was characterized by RNA‐sequencing. Comparative analysis of the gene expression of EVs isolated from LNP‐treated cells (HTB‐177 cells, HUVECs, and CPCs) and untreated controls revealed a robust transcriptional response against LNP treatment in all three cell lines.

**Figure 8:**
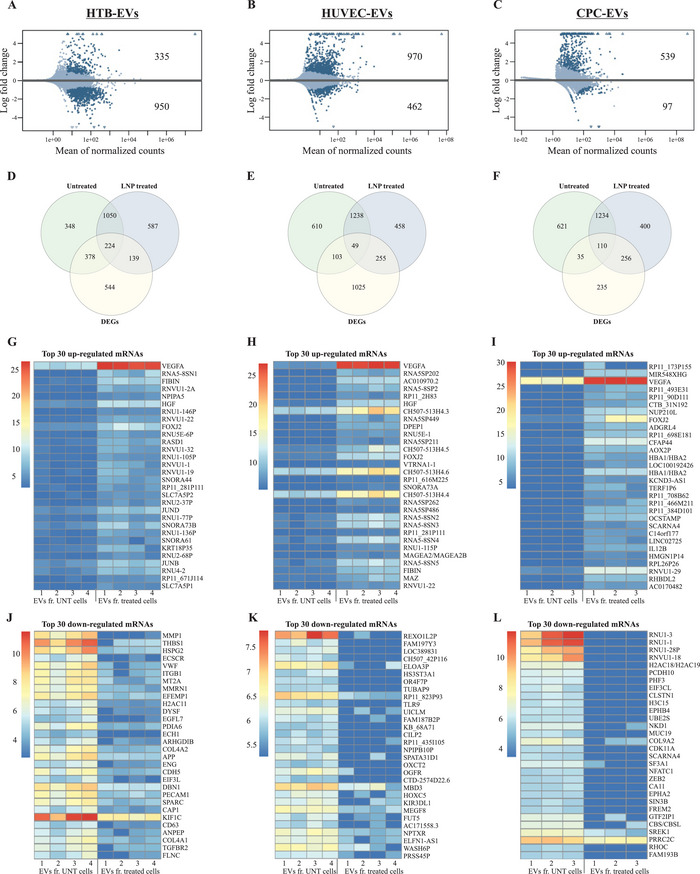
Endogenous genes are deregulated after lipid nanoparticle (LNP) treatment and are secreted into extracellular vesicles (EVs). The top panel shows MA plots of DEGs in EVs from A) HTB cells (*n* = 4), B) HUVECs (*n* = 4), and C) CPCs (*n* = 4) after LNP treatment. Dark blue dots represent significant DEGs (*p* < 0.05). The Venn diagrams in the middle panel show the intersection of the 2000 most highly expressed genes (using mean of replicates) in untreated and LNP‐treated samples, and the total number of identified DEGs in D) HTB‐, E) HUVEC‐, and F) CPC‐EVs. The heatmaps in the bottom panel represent normalized expression values (rows as genes and columns as samples) of the 30 most G–I) upregulated and J–L) downregulated genes, selected based on fold change (logFC). Shrunken logFC values are used for the visualizations in MA plots and heatmaps, and standard logFC values are used for the Venn diagrams.

Hierarchical clustering showed a distinct grouping of samples treated with LNP and untreated controls. Significant differentially expressed genes (DEGs) in EVs from each cell type (HTB‐EVs, HUVEC‐EVs, and CPC‐EVs) after LNP treatment were visualized in an MA plot (**Figure** **8**A–C and Tables S1–S3, Supporting Information). Using combined criteria of false discovery rate < 0.05 and absolute log_2_ fold change > 1, we identified 1285 DEGs in HTB‐EVs (Figure 8A, and Table S1, Supporting Information), 1432 DEGs in HUVEC‐EVs (Figure 8B and Table S2, Supporting Information), and 636 DEGs in CPC‐EVs (Figure 8C and Table S3, Supporting Information). After LNP treatment, 335, 970, and 539 genes were upregulated in HTB‐EVs, HUVEC‐EVs, and CPC‐EVs, respectively. Conversely, 950, 462, and 97 genes were downregulated in HTB‐EVs, HUVEC‐EVs, and CPC‐EVs, respectively.

The quality and expression assessment of RNA‐Seq data was evaluated. The clustered heat maps represent the sample correlation based on the normalized expression of the top 500 genes with the highest variance from HTB‐EVs, HUVEC‐EVs, and CPC‐EVs (Figure S9A, Supporting Information). Additionally, the mean normalized expression of each gene in the LNP‐treated condition (X‐axis) against the untreated condition (Y‐axis) from HTB‐EVs, HUVEC‐EVs, and CPC‐EVs was also analyzed and presented by dot plots (Figure S9B, Supporting Information). The results showed negative or very weak gene expression correlations in these EVs derived from cells before and after LNP treatment (i.e., mRNA transcript expression in these EVs changed after LNP treatment (Tables S1–S3, Supporting Information).

The total number of differentially regulated and overlapping genes among HTB‐EVs, HUVEC‐EVs, and CPC‐EVs from LNP‐treated or untreated cells were presented as Venn diagrams in Figure 8D–F. Only small fractions of the DEGs were among the top 2000 highest expressed genes in EVs from the untreated and LNP‐treated cells. In both LNP‐treated and untreated groups, the intersecting numbers of DEGs with the most highly expressed genes were 224 in HTB‐EVs, 49 in HUVEC‐EVs, and 110 in CPC‐EVs. The Venn diagrams also illustrate that > 50% of the top 2000 most highly expressed genes in both untreated and LNP‐treated cells were not significantly affected by LNP treatment, including 1050 from HTB‐EVs, 1238 from HUVEC‐EVs, and 1234 from CPC‐EVs. Moreover, 37–72% of DEGs (544 from HTB‐EVs, 1025 from HUVEC‐EVs, and 235 from CPC‐EVs) were expressed at a low‐to‐medium level and did not overlap with the 2000 highest expressed genes in the two groups investigated.

Additionally, the top 30 up‐ and downregulated genes in HTB‐EVs, HUVEC‐EVs, and CPC‐EVs after LNP treatment (compared with untreated EVs) were visualized and presented as heatmaps (Figure 8G–L). Complete information on these genes is presented in Table S4 (Supporting Information) (upregulated genes) and Table S5 (Supporting Information) (downregulated genes).

### 2.8 Comparison of DEGs in Modified EVs after LNP Treatment

**Figure 9:**
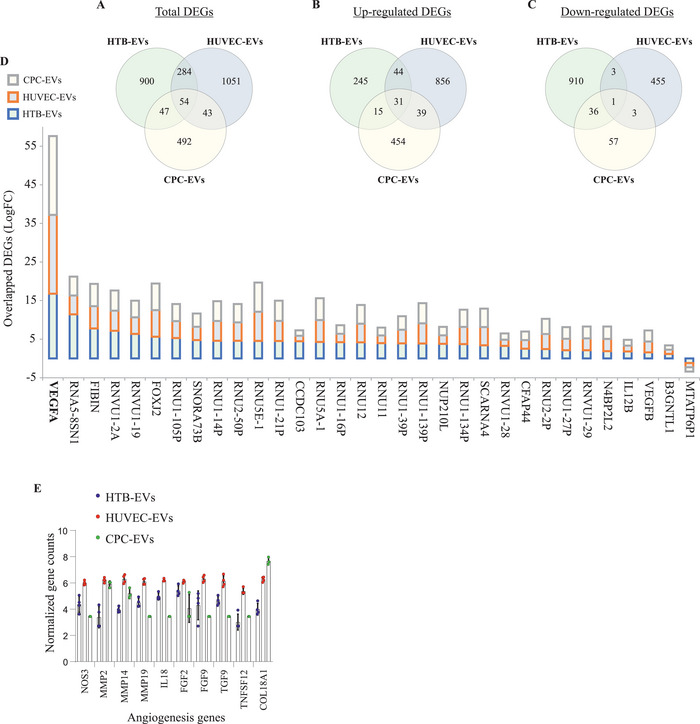
Proangiogenic genes are secreted into extracellular vesicles (EVs) After lipid nanoparticle‐*VEGF‐A* (LNP‐*VEGF‐A*) treatment to cells. The Venn diagrams illustrate the overlapping DEGs (*p* < 0.05 and absolute log_2_ fold change > 1) in EVs from HTB cells (*n* = 4), HUVECs (*n* = 4), and CPCs (*n* = 4) after LNP treatment. A) The total overlap of DEGs in all three EV types. The overlaps of B) upregulated, and C) downregulated DEGs in all three EV types. D) A stacked bar plot showing log_2_ fold change of all the 32 DEGs from panels B and C, which demonstrate overlapping transcriptional patterns in the same direction (either up‐ or down‐regulated) in all EV types after LNP treatment. E) Top 10 angiogenesis genes identified in EVs of LNP‐treated cells. *BiomaRt* R package was applied, and 262 unique Ensembl gene IDs in EV‐mRNAs associated with angiogenesis (GO:0001525) were identified (a full list is provided in Tables S7–S9, Supporting Information); the top 10 angiogenic genes were selected (*n* = 4).

After analyzing the EV gene expression between LNP‐treated versus untreated cells, the differences in EV gene expression were investigated among LNP treated cells only. The Venn diagrams show the overlapping DEGs between EVs of three LNP‐treated cell lines (**Figure** **9**A–C). A comparative analysis of DEGs across the three cell lines showed that 54 genes ( Table S6, Supporting Information) were differentially expressed in EVs from all three LNP‐treated cell lines (Figure 9A). Of these 54 DEGs, 32 genes were regulated in the same direction in all three cell types. Of these 32 genes, 31 genes were upregulated in HTB‐EVs, HUVEC‐EVs, and CPC‐EVs (Figure 9B), and 1 gene was downregulated in all three cell types (Figure 9C). The remaining 22 genes (of the 54 DEGs) were deregulated, but not in the same direction, in all cell lines. The stacked bar plot illustrates the logFC of the 32 overlapping DEGs (31 upregulated and 1 downregulated) (Figure 9D). Importantly, consistent responses were observed after LNP treatment in all cell lines, indicating the effect of LNP treatment on gene regulation. In all LNP‐treated cell lines, the *VEGF‐A, VEGF‐B, IL12B*, *FIBIN*, and *FOXJ2* were some of the common DEGs that were upregulated, whereas *MTATP6P1* was downregulated.

Additionally, we performed canonical pathways on EV‐transcripts expressed after LNP‐*VEGF‐A* mRNA delivery and analyzed the pathways in which these genes are involved, especially related to cardiac functions. The top 5 canonical pathways identified were not associated with cardiac physiology, except for mTOR signaling and calcium signaling. Then a manual search among all the canonical pathways that the program Ingenuity had identified, showed that the transcripts of CPC‐EVs are totally involved in five canonical pathways associated with cardiac functions (Table S10, Supporting Information). HTB‐EVs and HUVEC‐EVs were involved in four pathways associated with cardiac activity. The cardiac associated pathways that were common to all the three EVs were: cardiac *β*‐adrenergic signaling, cardiac hypertrophy signaling, and role of NFAT in cardiac hypertrophy. Proteins involved in each pathway are indicated in Table S10 (Supporting Information).

### 2.9 EVs Secreted from LNP‐VEGF‐A Treated Cells Contain Proangiogenic Genes

Based on the observation that the production of VEGF‐A protein via CPC‐EVs was approximately threefold less than that of LNPs but vessel formation was highest by CPC‐EVs and lowest by LNPs, per VEGF‐A protein produced (Figure 7B, compared with 7G), the RNA‐Seq data was further investigated to sort out angiogenic genes detected in EVs. A total of 262 unique Ensembl gene IDs associated with angiogenesis (GO:0001525) were identified using *biomaRt* R package. The expression data showed that EVs isolated from LNP‐treated cells carried several genes that are involved in angiogenesis. Out of these 262 genes, 259 angiogenic genes were expressed in HTB‐EVs and HUVEC‐EVs, whereas 255 angiogenic genes were expressed in CPC‐EVs (Tables S7–S9, Supporting Information). Importantly, EVs from LNP treated cells also contained several other upregulated angiogenic mRNA transcripts (in addition to *VEGF‐A* mRNA). The expression of the most important angiogenic genes that were common to all three EV types is presented in Figure 9E.

### 2.10 Intravenous Delivery of Luciferase Encoding mRNA via EVs or LNPs

EVs or LNPs containing 1 µg of luciferase mRNA (FLuc‐mRNA) were administered intravenously to female C57bl/Ncr. After 6 h of EV or LNP administration, luciferin (≈5 mL kg^‐1^ RediJect D‐Luciferin) was administrated intravenously. The mice were terminated 20 min after the luciferin administration and the organs were dissected and scanned with a IVIS Spectrum within less than 5 min after termination. The total radiance was quantified and used as marker for translatable luciferase mRNA. Results showed that systemic administration of EVs could deliver the translatable mRNA in different organs with highest levels in the liver. Compared to untreated, significant amounts of radiance by LNP‐ and EV‐FLuc mRNA delivery were also detected in lung, kidney, and spleen. However, in heart, and pancreas, EV delivery was insignificantly higher than untreated ( Figure S10, Supporting Information).

### 2.11 Intramyocardial Delivery of VEGF‐A mRNA via EVs or LNPs or Naked VEGF‐A

**Figure 10:**
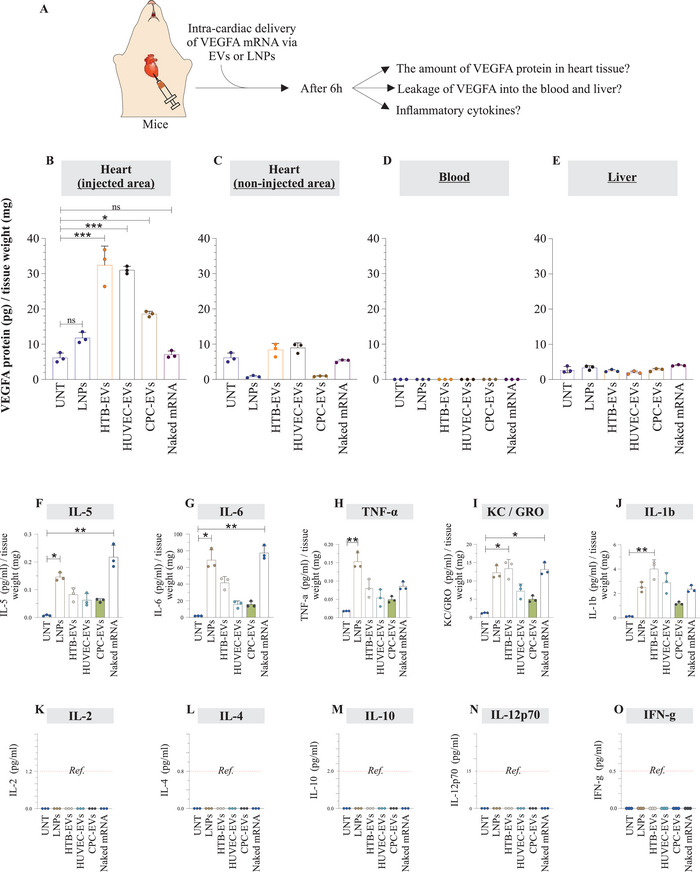
Extracellular vesicles (EVs), and lipid nanoparticles (LNPs) can deliver translatable *VEGF‐A* mRNA to heart via intramyocardial injections. A) An equal amount of EV‐*VEGF‐A* mRNA, LNP‐*VEGF‐A* mRNA, or naked *VEGF‐A* mRNA (50 ng) was directly injected into the myocardium of the left ventricle in mice. After 6 h, samples from the injected and non‐injected areas of the heart, blood, and liver were collected and analyzed for VEGF‐A protein levels. B–E) Quantification of VEGF‐A protein in the injected and non‐injected areas of the heart, blood, and liver (*n* = 3). The amount of VEGF‐A protein was normalized to the tissue weight. Statistically significant differences between UNT (untreated) and *VEGF‐A* mRNA treated mice (*n* = 3 each) were examined by ordinary one‐way ANOVA test. (*p < 0.05, **p < 0.01, ***p < 0.001). F–O) Inflammatory cytokine analysis of heart tissue after injections with extracellular vesicles (EVs) or lipid nanoparticles (LNPs). Tissue proteins were extracted from the injected areas of the heart (after 6 h of EV‐*VEGF‐A* mRNA, LNP‐*VEGF‐A* mRNA, or naked *VEGF‐A* mRNA injection), and a multiplex cytokine analysis (comprising a panel of 10 cytokines) was performed (*n* = 3). Statistically significant differences were evaluated by the Kruskal‐Wallis test followed by the Dunn's multiple comparison test to compare *VEGF‐A* mRNA treated mice with UNT (untreated) mice. Only the groups which showed statistical differences are labeled (*p < 0.05, **p < 0.01). Cytokine levels were normalized to the tissue weight used for protein extraction. Ref, reference value.

Since the overexpression of VEGF‐A protein in organs other than heart was not desired, EV‐, LNP‐, or naked *VEGF‐A* mRNA in citrate solution was directly injected into the myocardium of mice. In recent clinical trials on patients undergoing coronary artery bypass grafting, naked *VEGF‐A* mRNA in citrate saline solution (without LNPs as RNA carriers) was administered via direct intracardiac injections.^[^ ^58^, ^59^ ^]^ In the current study, EVs or LNPs were used as vehicles for *VEGF‐A* mRNA delivery. The injected and noninjected areas of the heart, blood, and liver of the mice were collected 6 h postinjection and analyzed for VEGF‐A protein and cytokine levels (**Figure** **10**A). Despite the delivery of equal amounts of *VEGF‐A* mRNA, the levels of produced VEGF‐A protein differed between delivery via EV‐, LNP‐, and naked mRNA. The highest amounts of VEGF‐A protein were detected in the injected area. Specifically, *VEGF‐A* mRNA delivery via HTB‐EV, HUVEC‐EV, and CPC‐EV showed significantly higher levels of VEGF‐A protein production compared to LNP treatment or naked *VEGF‐A* mRNA (Figure 10B). The levels of VEGF‐A protein in non‐injected areas indicated that the amount of *VEGF‐A* mRNA distributed to non‐injected areas of the heart was insignificant, compared with their counterparts in the injected areas (Figure 10C). Additionally, the leakage of VEGF‐A protein was analyzed. The produced VEGF‐A protein had not spilled over to liver, as shown by its levels in the blood and liver (Figure 10D,E).

### 2.12 EVs of Cardiac Origin When Injected to Heart, Caused Minimal Local Inflammation in the Heart

To examine local inflammation after direct injections of EV‐, LNP‐, or naked *VEGF‐A* mRNA, the cytokine analysis was performed on tissue proteins extracted from the injected areas of the heart. The expression levels of inflammatory cytokines differed between EVs of different cell sources, LNPs, and naked mRNA (Figure 10F–O). Importantly, EVs from cardiac progenitor cells caused minimal production of inflammatory cytokines (IL5, IL6, TNF‐*α*, KC/GRO, and IL1b) in cardiac tissue compared with all other treatment types used in this study. The levels of some cytokines were below the reference values, regardless of the delivery of EVs, LNPs, or naked mRNA.

## 3 Discussion

LNPs are clinically approved RNA transport vehicles and are currently used for the transport of COVID‐19 mRNA vaccines manufactured by Pfizer‐BioNTech and Moderna.^[^ ^40^, ^41^ ^]^ In the current study, we used one of the clinically approved LNPs (MC3‐LNPs) to deliver different mRNA molecules, including mRNA encoding VEGF‐A protein, in vitro and in vivo.

It should be noted that only mRNA molecules that escape the endosomes and translocated to the rough endoplasmic reticulum (coated with ribosomes) in the cytosol are accessible for mRNA translation. Our results show that cellular uptake of LNPs and their mRNA molecules occurs quickly, and the translation of LNP‐delivered mRNA begins immediately after cellular uptake of LNPs, indicating that endosomal escape of their mRNA starts in early hours.

The stoichiometric analysis of the kinetics of *VEGF‐A* mRNA uptake shows that the maximum amount of *VEGF‐A* mRNA in LNP‐treated cells was at 1 h (the peak with 1355 copies per cell). At the same time point (1 h), only 6 *VEGF‐A* mRNA copies per EV were detected. However, the maximum amount of *VEGF‐A* mRNA copies detected in EVs was at 5 h (the peak), which was 31 copies per EV. The peak of *VEGF‐A* mRNA in EVs observed later than cells suggests that first LNP‐*VEGF‐A* mRNA is internalized by cells and then it is secreted via EVs few hours later.

The amount of *VEGF‐A* mRNA in cells and total EVs detected at their corresponding peaks (1 h, and 5 h respectively), apparently gives a notion that the amount was higher in EVs compared to cells. However, the numbers of *VEGF‐A* mRNA/single EV at the same time points revealed that, compared to EVs, the cells contained 225‐ and 14‐fold more *VEGF‐A mRNA* copies per cell at 1 and 5 h, respectively. Stochiometric calculations showed that an individual EV contains far less mRNA copies than an individual cell, and that the single cell can secret the same mRNA in several different EVs.

The results from the current study and other studies have shown that internalization of LNPs occurs within a few hours after administration.^[^ ^49^, ^51^ ^]^ Thus, 1 h after LNP‐*VEGF‐A* mRNA, treatment to cells, the culture media was removed, cells were washed, and then fresh media (without LNPs) was added. This removed any intact LNPs from the extracellular environment which were not taken up by cells within 1 h and EVs secreted within this time. After 24 h incubation, the newly secreted EVs were isolated from these cells that had taken up LNPs before the wash. The results showed that *VEGF‐A* mRNA was detected in EVs. Indeed, these EVs were secreted after the removal of LNPs from the extracellular environment, which suggests that they received the *VEGF‐A* mRNA from their parental cells, which had taken up LNP‐mRNA before the wash. This also supports the results of distinct peaks of LNP‐*VEGF‐A* mRNA in cells and their secreted EVs, i.e., first LNP‐mRNA was detected in cells, with a peak at 1 h, after which EVs containing *VEGF‐A* mRNA were secreted, with a peak at 5 h. Hence, these data suggest that a fraction of LNP‐mRNA, which is taken up by cells, is egressed via secretion of EVs.

**Figure 11:**
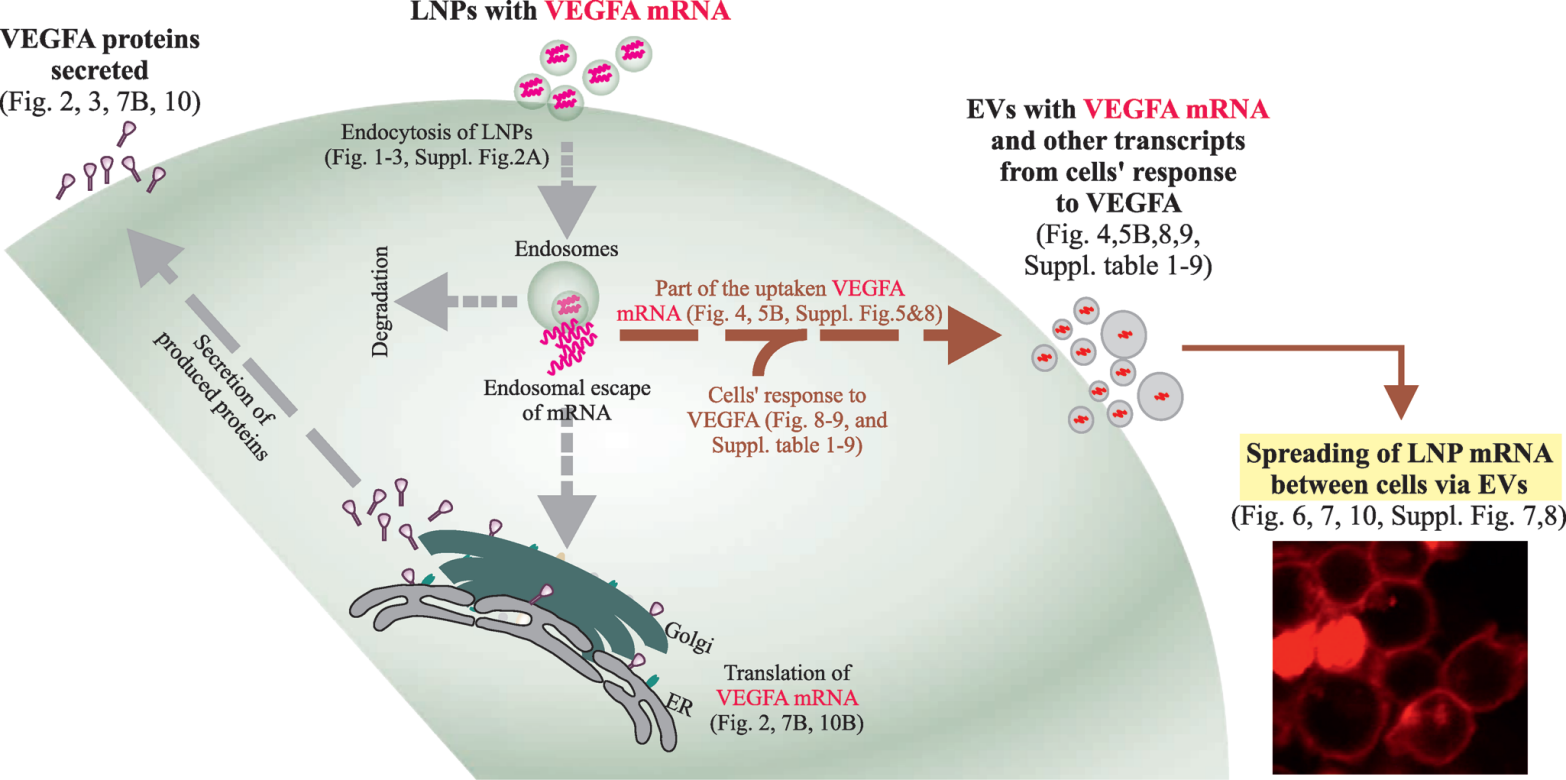
Extracellular vesicles (EVs) spread the mRNA of internalized lipid nanoparticles (LNPs) between cells. Illustration of experimental data showing the intracellular fate of internalized LNP‐*VEGF‐A* mRNA and the role of EVs in delivering LNP‐mRNA between cells, as well as deregulated/adopted mRNA transcripts of cells expressed in response to LNP‐mRNA. Following the uptake of LNP‐mRNA by cells, LNP‐*VEGF‐A* mRNA was translated into protein, part of which remained inside cells and the majority was secreted into the extracellular space (VEGF‐A is a secretory protein). Additionally, a small fraction of the internalized LNP‐*VEGF‐A* mRNA was secreted into EVs and transported to other cells. In response to LNP*‐VEGF‐A* treatment, the endogenous mRNAs of cells were also altered, with several overexpressed proangiogenic genes, which were detected in EVs. These EVs are capable of transporting the overexpressed angiogenic *VEGF‐A* mRNA and other altered mRNA transcripts between cells, and represent functional extensions of LNPs, where certain EVs might be better adopted, in response to LNPs.

As EV secretion from cells and the transport of RNA molecule between cells via EVs is a natural secretory process,^[^ ^1^ ^]^ we investigated whether a fraction of LNP‐mRNA is also transported between cells via EVs. This would mean that not all cells in a population receive the administered mRNA via LNPs, but mRNA may also be distributed to numerous cells via EVs pertaining to natural secretory processes. As CPC‐, HUVEC‐, and HTB‐EVs from LNP‐treated cells contained endocytosed LNP‐*VEGF‐A* mRNA, we investigated whether these EVs were capable of transporting *VEGF‐A* mRNA to other cells and cause the production of VEGF‐A protein in recipient cells in vitro and in vivo. The three EV types successfully delivered *VEGF‐A* mRNA to endothelial cells and showed elevated levels of newly produced VEGF‐A protein. Our results confirm that these EVs can transfer exogenous mRNA across the cell membrane into the cytosol of recipient cells and keep it protected for translation into protein. This also suggests that a fraction of LNP‐mRNA is spread or distributed between cells via EVs (i.e., LNPs alone, do not distribute their mRNA to all cells in a population, but a number of cells could receive this mRNA via EVs). **Figure** **11** illustrates the intracellular fate of internalized LNP‐*VEGF‐A* mRNA and the role of EVs in the delivery of LNP‐mRNA. The part of the internalized LNP‐*VEGF‐A* mRNA is translated into VEGF‐A protein and secreted into the extracellular space. The part of the internalized LNP‐*VEGF‐A* mRNA is packaged into EVs and then secreted. In response to LNP‐*VEGF‐A* treatment, the endogenous mRNAs are altered and packaged into EVs (as observed by deregulated EV transcriptomics), including proangiogenic genes. These altered mRNAs in EVs remain protected and are transported to other cells in which they are functional. Several of these genes (including *VEGF‐A* mRNA) are angiogenic which are incorporated into EVs and distributed to other cells. To our knowledge, there is no known method to control the LNP‐mRNA spreading between cells. However, inhibiting the EV release by blocking EV biogenesis pathways could be a possible way to control the nonspecific spread.

After the delivery of equal quantity of *VEGF‐A* mRNA (60 ng) to endothelial cells via LNPs or EVs, the LNPs can deliver *VEGF‐A* mRNA most efficiently, resulting in the highest levels of VEGF‐A protein production in vitro. The CPC‐EVs were the least effective, resulting in the lowest production of VEGF‐A protein (≈threefold less than that of LNPs). However, surprisingly, CPC‐EVs were the most efficient in promoting angiogenesis (vascular network formation) per amount of VEGF‐A protein produced (Figure 7G, compared with 7B).

The EV transcriptome was further characterized to understand which other mRNA transcripts these EVs contain that may be favorable for recipient cells to induce angiogenesis. Transcriptomic analysis of EVs secreted from LNP‐*VEGF‐A* mRNA cells treated revealed that cells exhibit robust a response to *VEGF‐A* mRNA delivery and overexpresses angiogenic genes, which are secreted via EVs regardless of cell type (HTB‐, HUVEC‐, and CPC‐EVs). Previously, it was proposed that artificial exosome‐based (bioinspired) LNPs can deliver miRNAs that are responsible for EV‐induced angiogenesis.^[^ ^62^ ^]^ Here, we propose a possible reason: LNP‐*VEGF‐A* mRNA treatment makes CPC‐EVs more effective in facilitating angiogenesis in endothelial cells. Unlike LNPs (which contained only one angiogenic mRNA, i.e., *VEGF‐A* mRNA), CPC‐EVs also contained additional mRNAs involved in angiogenic activities (e.g., *NOS3, FGF2, FGF9, TGF9*, and *TNFSF12*), and mRNAs encoding enzymes that modulate matrix during vessel formation (e.g., *MMP‐2* and *MMP‐14)*. Previously, it has been proposed that endogenous cardiac stem cell–secretome or biomimetic cardiac stem cell secretome can facilitate cardiac repair.^[^ ^63^, ^64^ ^]^ Cardiac stem cell‐derived EVs transformed by LNP‐*VEGF‐A* treatment become loaded with angiogenic molecules, which could be therapeutically beneficial for cardiac repair. To this end, more investigations are warranted.

EV‐, LNP‐, or naked *VEGF‐A* mRNA was directly injected into the myocardium of mice. This administration led to local production of VEGF‐A protein with the highest levels detected in the injected area, compared with non‐injected areas (Figure 10B,C). The results from the current study were of particular importance that after localized administration the produced VEGF‐A protein did not leak out (as shown by its absence in blood and liver). Compared to endogenous VEGF‐A protein in untreated mice, no increase was observed in the liver of treated mice, indicating that VEGF‐A was not leaked. This form of administration is of particular interest as the expression of VEGF‐A protein is not desired in other organs due to its angiogenic/tumorigenic characteristics.

Importantly, the examination of the local inflammation after direct injections of EV‐, LNP‐, or naked *VEGF‐A* mRNA, showed that EVs from cardiac progenitor cells caused minimal production of inflammatory cytokines (expression of IL5, IL6, TNF‐*α*, KC/GRO, and IL1b) in cardiac tissue, compared to all other treatment types. These results show that CPC‐EVs are more immunologically adapted for mRNA transport to the heart. In our opinion, mRNA‐based therapies are partly about how well the RNA vehicle communicates with the tissue, e.g., whether a vehicle is adopted or immunogenic to the tissue/recipient. The comparison of four vehicles (LNPs and the three EV types) locally administered to heart tissue shows that EVs from cardiac progenitor cells induce less expression of inflammatory cytokines compared to the other three vehicles, which indicate CPC‐EVs communicate better.

Data from this study also demonstrate the differences between chemically synthesized and biological mRNA transport vehicles, i.e., LNPs and EVs, respectively. Indeed, a biological RNA carrier is a complex vehicle that may contain other RNAs and proteins that might be beneficial to recipient cells to achieve the desired function. This is reflective from the delivery of *VEGF‐A* mRNA to cells using LNPs versus three different EV types as mRNA transport vehicles.

Currently, other than localized naked mRNA administration, there has been not proposed an effective and safe vehicle to deliver *VEGF‐A* mRNA to heart cells. Chien and co‐workers have proposed the need of packaging systems including EVs to investigate *VEGF‐A* mRNA.^[^ ^65^ ^]^ We propose that EVs may have an advantage to act as RNA transport vehicles, as EVs naturally transport bioactive RNA transcripts between cells, e.g., mRNA transcripts involved in angiogenesis, as observed with CPC‐EVs. Importantly, CPC‐EVs were also effective in causing *VEGF‐A*‐dependent angiogenesis per amount of VEGF‐A protein produced, in vitro. This warrants new investigations on whether EVs from homologous organs can be used for tissue‐customized mRNA delivery. It might be possible to identify EVs that are cell/tissue‐customized for delivery to specific cells and organs.

## 4 Experimental Section

### Construction of VEGF‐A mRNA Sequence and Clean Capping

The CDS sequence of *VEGF‐A* 165 (isoform 11) containing an open reading frame with start and stop codon (576 nucleotides including atg start and tga stop codons, encoding for 191 amino acids of VEGF‐A protein) was selected. The sequence code was provided to TriLink Biotechnologies (CA, USA) for *VEGF‐A* mRNA construct with clean cap modifications. A CleanCap (AG, polyadenylated) method was applied which is a cotranscriptional capping method with a fully processed mature mRNA and was optimized for mammalian systems. After clean cap modifications with polyadenylation, the resulting mRNA length was 852 nucleotides. The prepared *VEGF‐A* mRNA was dissolved in 1 × 10^‐3^ m sodium citrate buffer (pH, 6.4), and stored at ‐80 °C.

The sense strand of *VEGF‐A* mRNA used in this study is presented below.

AUGAA CUUUC UGCUG UCUUG GGUGC AUUGG AGCCU UGCCU UGCUG CUCUA CCUCC ACCAU GCCAA GUGGU CCCAG GCUGC ACCCA UGGCA GAAGG AGGAG GGCAG AAUCA UCACG AAGUG GUGAA GUUCA UGGAU GUCUA UCAGC GCAGC UACUG CCAUC CAAUC GAGAC CCUGG UGGAC AUCUU CCAGG AGUAC CCUGA UGAGA UCGAG UACAU CUUCA AGCCA UCCUG UGUGC CCCUG AUGCG AUGCG GGGGC UGCUG CAAUG ACGAG GGCCU GGAGU GUGUG CCCAC UGAGG AGUCC AACAU CACCA UGCAG AUUAU GCGGA UCAAA CCUCA CCAAG GCCAG CACAU AGGAG AGAUG AGCUU CCUAC AGCAC AACAA AUGUG AAUGC AGACC AAAGA AAGAU AGAGC AAGAC AAGAA AAUCC CUGUG GGCCU UGCUC AGAGC GGAGA AAGCA UUUGU UUGUA CAAGA UCCGC AGACG UGUAA AUGUU CCUGC AAAAA CACAG ACUCG CGUUG CAAGG CGAGG CAGCU UGAGU UAAAC GAACG UACUU GCAGA UGUGA CAAGC CGAGG CGGUG A.

(1) $$A=157\left(27\%\right),\;U=118\left(20\%\right),\;C=143\left(25\%\right),\;G=158\left(27\%\right)$$

AUG: start codon, UGA: stop codon

### Formulations of Lipid Nanoparticles (LNPs) and mRNA Loading

DLin‐MC3‐DMA LNPs containing modified *VEGF‐A* mRNA (852 nucleotides, 5meC, Ψ TriLink Biotechnologies, USA) were prepared by precipitating the mRNA with four different lipid components. These components consist of an ionizable lipid, DLin‐MC3‐DMA (MC3), two helper lipids (DSPC and Cholesterol) and a PEGylated lipid (DMPE‐PEG2000). A solution of *VEGF‐A* mRNA in water was prepared by mixing mRNA dissolved in MilliQ‐water, 100 × 10^‐3^ m citrate buffer pH = 3 and MilliQ‐water to give a solution of 50 × 10^‐3^ m citrate. Lipid solutions in ethanol (99.5%) were prepared with a composition of four lipid components [MC3:Cholesterol:DSPC:DMPE‐PEG2000] = 50:38.5:10:1.5 mol% and a total lipid content of 12.5 × 10^‐3^ m. The mRNA and lipid solutions were mixed in a NanoAssemblr (Precision NanoSystems, Inc., BC, Canada) microfluidic mixing system at a volume mixing ratio of 3:1 and a constant total flow rate of 12 mL min^‐1^. At the time of mixing, the ratio between the nitrogen atoms on the ionizable lipid and phosphor atoms on the mRNA chain was 3.08:1.

For all formulations of mRNA loaded MC3‐LNPs used in the current study, a 10:1 (w/w) total lipid:mRNA was applied, which gives 1/(10+1)*100 = 9.1% (w/w) mRNA. In other words, 10:1 (w/w) total lipid/mRNA was applied.

In some preparations of LNPs, CleanCap Cy5‐*eGFP* mRNA (996 nucleotides, 5 meC, Ψ) and CleanCap *eGFP* mRNA (Trilink Biotechnology) were mixed with 1:1 ratio and encapsulated instead of *VEGF‐A* mRNA. The initial 0.25 mL and the last 0.05 mL of the LNP solution prepared were discarded while the rest of the volume was collected as the sample fraction. The sample fraction was transferred immediately to a Slide‐a‐lyzer G2 dialysis cassette (10 000 MWCO, Thermo Fischer Scientific Inc.) and dialyzed overnight at 4 °C against PBS (pH 7.4) to remove residue ethanol (25 v%). The volume of the PBS buffer was 600× the sample fraction volume. The dialyzed sample was collected and filtrated through a 0.22 µm sterile filter (Gillex, Merck) prior any characterization.

### Formulation of Radiolabeled LNPs

In order to track the LNP uptake, the LNPs were radioactive labeled. Briefly, the stocks of MC3, DSPC, cholesterol, and DMPE‐PEG2000 lipids were dissolved in ethanol and mixed in a mol% ratio of 50:10:38.5:1.5 to obtain a lipid concentration of 12.5 × 10^‐3^ m (1.80 mg mL^‐1^). For isotope‐labeled LNPs, additional ^14^C‐labeled hexadecyl cholesterol ester (^14^C‐Chol) stocks (2.81 mg mL^‐1^, 14 800 000 Bq mL^‐1^) was added on top of the lipid stock solution in a relative mol% ratio of 3.55:10 for 14C‐Chol:Cholesterol.

### Characterization of Formulated LNPs

The intensity‐averaged particle size (Z‐average, d_Z_) was measured using ZetaSizer (Malvern Instruments Inc.). The measurement solution was made by diluting 20 µL of the sample fraction using 980 µL PBS (pH 7.4). The mRNA concentration and encapsulation efficiency (EE) of the final product were measured by Quant‐it Ribogreen Assay Kit (Thermo Fischer Scientific).

### Cell Cultures

The human epithelial HTB‐177 (NCI‐H460) cell line purchased from ATCC was cultured according to the manufacturer's protocol. The HTB‐177 cells were cultured in RPMI‐1640 growth medium containing sodium bicarbonate, without sodium pyruvate and HEPES (Sigma Aldrich), which was supplemented with 10% EV‐depleted fetal bovine serum (FBS) (Sigma Aldrich), 1% of L‐glutamine and 1% penicillin–streptomycin (Thermo Fisher Scientific), at 37 °C and 5% CO_2_. The medium was replaced with a fresh medium after 48 h, followed by adding LNP‐*VEGF‐A* mRNA to the cells in culture for an experimental period of 24 h. The heat‐inactivated FBS (56 °C, 1 h) was EV‐depleted by ultracentrifugation at 120 000 × *g* for 2 h, at 4 °C on an Optima L‐100 XP ultracentrifuge with 70Ti rotor (Beckman Coulter). EV‐depleted supernatant was filtered using 0.2 µm filters before further use in RPMI‐1640 growth medium.

Additionally, human umbilical vein endothelial cells (HUVECs) (Lonza, Switzerland) were plated and expanded in culture medium according to the manufacturer's instructions (CC‐5035 EGM‐PLUS BulletKit Medium; CC‐5036 EGM‐PLUS Basal Media + CC‐4542 EGM‐PLUS SingleQuots Kit, Lonza, Switzerland). Briefly, cells were cultured in T‐75 cm^2^ culture flasks with EGM‐Plus medium and incubated at 37 °C in 5% CO_2_ and 95% saturated atmospheric humidity. The culture medium was replaced with fresh media every 2 d until the cells attained around 80% confluency, and then the cells were expanded. At 80% confluency, the cells were rinsed with Ca^++^/Mg^++^ free Dulbecco's phosphate buffered saline. TrypLE Express Enzyme (1X), without phenol red, was added to detach the cell layer from the flask. The enzyme activity was stopped by adding the complete culture medium to the flask. The cells were aspirated by gently pipetting and transferred to a tube and centrifuged at 1000 rpm for 5 min. The cell (HUVECs) pellet was resuspended with fresh culture medium and dispensed into a T‐75 cm^2^ culture flask at a density of 2 × 10^6^ cells per flask and incubated at 37 °C and 5% CO_2_ for 24 h followed by the addition of LNP‐*VEGF‐A* mRNA to each flask for an experimental period of additional 24 h. At the endpoint, the conditioned medium was collected for EV isolation. Also, the cells were detached from the culture flask using TrypLE, as described before. The cells were counted and checked for their viability before centrifugation. The cell pellets were used for further analysis. All the steps were carried out under aseptic conditions.

iCell cardiac progenitor cells (R1093, Fujifilm Cellular Dynamics, Madison, WI, USA) were thawed, centrifuged (180 ×*g* for 5 min), resuspended in the maintenance medium composed of William's E Medium, Cocktail B (Thermo Fisher Scientific) and seeded in fibronectin‐coated (1 mg mL^‐1^ fibronectin solution diluted in sterile D‐PBS to a final concentration of 5 µg mL^‐1^ immediately before use, Sigma‐Aldrich, St. Louis, MO, USA) six‐well plates at a density of 500 000 cells per well. Cells were then incubated at 37 °C in the ambient atmosphere supplemented with 5% CO_2_ and 95% relative humidity. The medium was replaced with a fresh maintenance medium after 24 h, followed by adding LNP‐*VEGF‐A* mRNA to each well for an experimental period of an additional 24 h. At the endpoint, the conditioned medium was collected for EV isolation. Also, the cells were detached from the culture plates using TrypLE, as described before. The cells were counted and checked for their viability before centrifugation. The cell pellets were used for further analysis. All the steps were carried out under aseptic conditions.

### Uptake of ^4^C‐Labeled Hexadecyl Cholesterol Ester LNPs by Cells

The cellular uptake kinetics of radio‐labeled LNPs was performed at the following time points: 0.5, 2, 5, 24, 48, 72, and 96 h. Untreated cells (0 h) were used as controls. The time‐dependent disappearance (consumption) of LNPs from cultured media was measured by quantifying the relative radioactivity using Microbeta Trilux 1450 scintillator instrument (PerkinElmer, Inc., Massachusetts, USA). The HTB‐177 cells were plated in 24‐well multiwell plates with a density of 4 × 10^5^ cells per well, each growing in 4 mL of culturing medium in the presence of 1% of human serum (Sigma Aldrich). For each time point, an individual plate containing radiolabeled LNPs‐treated cells and untreated cells were used (3 replicates per condition). For a control sample, the PBS (without LNPs) was added to the untreated cells. After each treatment (and all time points), the supernatants were collected and immediately used for radioactivity assay. A 25 µL of supernatant (per sample/replicate) was carefully transferred to a 96‐well multiplate format filter, which was air‐dried in a chemical hood for 3 h. Then, the filter was transferred on a hard preheated (90 °C) plastic film. The filter was then covered with a vax film. The vax was melted after a few seconds and the vax‐covered filter was removed from the heat plate. The filter was stored inside a plastic bag which was applied on the top of a 96‐well format cassette fitting inside the specific scintillator drawer. Next, the radioactivity of each sample/well in the filter was measured as count per minute (CPM) for ^14^C‐LNPs in 96‐well format cassette. Medium samples without ^14^C‐LNPs were used as Blank. The average of two readings was normalized to the blank for each test sample. The radioactivity of each individual sample medium was calculated, based on two reference samples: just medium (0%) and medium with LNPs added at time 0 (100%). Based on the % radioactivity in cultured medium, the % of LNP uptake was indirectly calculated (difference between 100% radioactivity, i.e., freshly added LNPs and the medium). Average values were plotted in a line chart using the average of three replicates.

### Delivery of Cy5‐eGFP mRNA to Cells via LNPs and mRNA Uptake Analysis

HTB‐177 cells were thawed at 37 °C and cultured in RPMI‐1640 medium (Sigma Aldrich) supplemented with 1% penicillin–streptomycin, 1% L‐Glutamine, and 10% EV‐depleted FBS. Medium was replaced with fresh medium every 2 d. The cells were expanded twice followed by seeding in 24‐well plates at a density of 80 000 cells per well. 24 h post seeding, the media was replaced with fresh media and incubated for an additional 24 h. Then the next day, 7.5 µg of Cy5‐*eGFP* mRNA were administrated via LNPs along with 1% of human serum. Cells were harvested at the following time points: 0.5, 2, 5, 24, 48, 72, and 96 h. Untreated cells (0 h) were used as control.

The samples collected at each treatment (time point) were analyzed by flow cytometry (BD FACSLyric, BD Biosciences) to detect and quantify the uptake of LNP‐Cy5‐*eGFP* mRNA by recipient cells. The results were analyzed using FlowJo software (Treestar Inc.).

### Delivery of LNP‐VEGF‐A mRNA to Human Epithelial, Endothelial, and Cardiac Progenitor Cells

The HTB‐177 and HUVECs were seeded at a density of 1 × 10^6^ cells, and 2 × 10^6^ cells /T75 flask, respectively in 15 mL of growth media. The CPCs were seeded at a density of 500  000 cells (1 × 10^5^) per well in six‐well multiwell plate in 3 mL culture media.

The culture medium was replaced with fresh media after 24 h (for HTB‐177), and 48 h (HUVEC and CPCs) of the incubation period. Then after 24 h adaptation with fresh media, the cells were treated with 30 µL of MC3‐LNPs containing 3 µg of *VEGF‐A* mRNA per replicate. The supernatants of cultured cells and the cell lysates were harvested 24 h post LNP‐mRNA administration. Untreated cells were used as controls.

### Isolation of Extracellular Vesicles (EVs) from LNP‐mRNA Treated Cells

EVs from conditioned media (15 mL from T75 flasks, and from six well plates: 5 wells × 3 mL = 15 mL) of LNP‐mRNA‐treated cells and negative controls were isolated, according to previously described method.^[^ ^18^ ^]^ Briefly, to remove cell debris, the collected conditioned medium was centrifuged at 3000 × *g* for 15 min at 4 °C on a 4K15 centrifuge (Sigma Aldrich). The supernatant was transferred to 12 mL Quick‐Seal tubes (Beckman Coulter) and ultracentrifuged at 60 000 × *g* for 35 min at 4 °C, followed by filtration through 0.2 µm filters under sterile conditions. Finally, the filtered supernatant was ultracentrifuged using Optima L‐100 XP ultracentrifuge with Ti70.1 rotor (Beckman Coulter) at 120 000× *g* for 70 min at 4 °C to pellet the EVs. The EV pellets were resuspended in 100–150 µL of pre‐filtered PBS (0.2 µm).

As control experiment, EVs from HTB‐177 cells were isolated by size exclusion chromatography (SEC) using qEV‐70/10 mL columns (Izon Science Ltd, New Zealand) according to the manufacturer's guidelines. After discarding the 20 mL void volume, the 11 fractions were collected each with 5 mL. Since manufacture's protocol guides that the first 4 fractions contain EVs, and later fractions contain free proteins, therefore the first 4 fractions were pooled and concentrated using 30 KDa amicon ultra/15 mL centrifuge filters (cat: # UFC903024, Sigma Aldrich, now Merck) and used for the analysis of *VEGF‐A* mRNA (qPCR) and VEGF‐A protein (ELISA).

### Quantification of LNP Particles, EV Particles, and Their VEGF‐A mRNA Copy Numbers

The LNP particle numbers (number of LNPs per volume) were calculated using a particle refractive index of 1.45, and according to the methods as described previously.^[^ ^61^ ^]^ The characteristics of LNPs from which particle number was calculated are given in **Table** **1**.

**Table 1:** Characteristics of LNPs used in the current study

LNP	mRNA	Encapsulation [%]	Size dZ [nm]	PDI	Concentration	Volume
MC3‐LNPs	*VEGF‐A*	99	85	0.024	0.1 mg mL^‐1^	1 mL

Additionally, the number of *VEGF‐A* mRNA copies (moles) detected in cells or EVs was calculated according to the following formula.

(2) $$n\;\left(\mathrm{mol}\right)\;=\frac{\mathrm{mass}\;\left(g\right)}{M.W\;\left(\frac{g}{\mathrm{mol}}\right)}$$

Following formula was used for the molecular weight (M.W) conversions of *VEGF‐A* mRNA;

(3) $$\begin{array}{ccc} M.W.\;\mathrm{of}\;\mathrm{VEGF}-\mathrm{AmRNA} & = & \left(\mathrm{An}\times 329.2\right)+\left(\mathrm{Un}\times 306.2\right)\\ & & +\left(\mathrm{Cn}\times 305.2\right)+\left(\mathrm{Gn}\times 345.2\right)+159^{a} \end{array}$$

An, Un, Cn, and Gn are the total number of each respective nucleotide within the whole *VEGF‐A* mRNA. The 159ª represents an addition of “159” to the M.W takes into account the M.W. of a 5′ triphosphate.

### Quantification of VEGF‐A Protein Copy Numbers

The copy numbers of VEGF‐A protein were calculated based on (*n*) moles (kDa), according to following formula.

(4) $$n\;\left(\mathrm{mol}\right)\;=\frac{\mathrm{mass}\;\left(g\right)}{M.W\;\left(\frac{g}{\mathrm{mol}}\right)}$$

The calculated molecular weight of VEGF‐A protein with 191 amino acids residue was 22.31364 kDa (encoded from *VEGF‐A* mRNA sequence used in this study). The sequence of encoded protein is provided below.

MNFLLSWVHWSLALLLYLHHAKWSQAAPMAEGGGQNHHEVVKFMDVYQRSYCHPIETLVDIFQEYPDEIEYIFKPSCVPLMRCGGCCNDEGLECVPTEESNITMQIMRIKPHQGQHIGEMSFLQHNKCECRPKKDRARQENPCGPCSERRKHLFVQDPQTCKCSCKNTDSRCKARQLELNERTCRCDKPRR.

### Characterization of EVs and Detection of Exogenous mRNA in EVs: Determination of Size and Concentration (Particle Number) of EVs

The size and concentration of EVs (from HTB‐177 cells) isolated at 0.5 h, 1 h, 5 h, and 24 h were determined by Nanoparticle Tracking Analysis (NTA LM14c, Malvern Panalytical, UK) equipped with a sCMOS camera type. The EV pellets were initially dissolved in 1000 µL of PBS, which were further diluted five times with PBS to reduce the number of particles in the field of view below 100 particles per frame. Two independent measurements (biological replicates) from each time point were performed in scatter mode. Measurement readings for each EV sample were taken in three captures for 90 s for the total of 2248 frames, at adjusted camera level of 16 and detection threshold of 5. Blur and Max Jump Distance were set to auto. The readings, acquisition and data analysis were examined using the NanoSight Fluorescent NTA LM14c software version 3.2 (Malvern Panalytical, UK).

Additionally, the size distribution of EVs isolated by size exclusion chromatography was determined by Zetasizer Nano ZS (Malvern Panalytical, UK) according to the manufacturer's instructions.

### Transmission Electron Microscopy (TEM) Analysis of EVs

The isolated EVs were fixed in 2% paraformaldehyde – 0.1 m phosphate buffered saline for 30 min. Glow discharge technique (30 s, 7,2 V, using a Bal‐Tec MED 020 Coating System) was applied over carbon‐coated copper grids, and immediately, the grids were placed on top of sample drops for 15 min. Then, the grids with adherent EVs were washed in a 0.1 m PBS drop. An additional fixation in 1% glutaraldehyde was performed for 5 min. After washing properly in distilled water, the grids were contrasted with 1% uranyl acetate and embedded in methylcellulose. Excess fluid was removed and allowed to dry before examination with a transmission electron microscope FEI Tecnai G2 Spirit (ThermoFisher Scientific, Oregon, USA). All images were acquired using Radius software (Version 2.1) with a Xarosa digital camera (EMSIS GmbH, Münster, Germany).

### Detection of Cy5‐eGFP mRNA in CD63^+^ EVs by Antibody Conjugated Bead Assays

To incorporate Cy5‐*eGFP* mRNA in EVs, the HTB‐177 cells (1 × 10^6^ cells/T75 flask) were cultured in 15 mL RMPI‐1640 media. The conditioned media was replaced with fresh media after 24 h, and cells were incubated for a period of 24 h. Then next day, 100 µL of LNPs containing 100 µg Cy5 *eGFP* mRNA were added to HTB cells and incubated at 37 °C for 24 h. EVs were isolated/pre‐enriched by ultracentrifuge method as described above. CD63^+^ EVs were captured by magnetic dynabeads conjugated anti‐CD63 antibody (Immunoaffinity assay: Exosome‐Human CD63 isolation/detection reagent for cell culture medium, Thermo Fisher Scientific, cat.#: 10606D). In the binding reaction, 30 µL of CD63‐antibody conjugated beads were incubated with 60 µg of EVs. CD63^+^ EVs captured/immobilized by CD63‐antibody were acquired on a BD FACSLyric system (BD Biosciences) to detect Cy5‐*eGFP* mRNA. The data were analyzed using FlowJo software (TreeStar Inc.). The EVs from untreated cells were used as controls. For negative controls, 30 µL of CD63‐antibody conjugated beads alone (without EVs or LNPs) were incubated with an equivalent volume of PBS. Additionally, LNPs were also used as control to examine the contamination factor of LNPs.

### Detection of CD63 and CD9 EV Markers within Same Fraction of EVs

EVs were isolated by ultracentrifugation method as described above. After pre‐enrichment, the CD63 and CD9 positive EVs were isolated/fetched by an immunoaffinity‐based method. CD63 isolation/detection reagent for cell culture medium (Thermo Fisher Scientific, cat.#: 10606D) was used to immobilize the CD63^+^ EVs to magnetic dynabeads conjugated with anti‐CD63 antibody. It is recommended to titrate EV samples based on either pre‐enriched method, i.e., ultracentrifugation or commercially available total exosome isolation reagent. In the current study, EV samples were titrated based on pre‐enriched method (ultracentrifugation). In the binding reaction, 30 µL of CD63‐antibody conjugated beads were incubated with 60 µg of EVs (beads + EVs; total volume 120 µL). As a negative control, 30 µL of CD63–antibody conjugated beads alone, were incubated with an equivalent volume of PBS (no EVs). The EVs were immobilized on anti‐CD63 beads and incubated overnight (day 1). Then the next day (day 2) unbound beads or EVs were washed 4 times with BSA–PBS isolation buffer (0.25% BSA dissolved in PBS). The washing steps were performed using magnetic separators (EasySep, StemCell technologies), according to the manufacturer's protocol provided with the kit (cat.#: 10606D). After the final wash, the immobilized CD63^+^ EVs were suspended in 120 µL of BSA‐PBS isolation buffer and then further stained with a mouse anti‐human PE‐CD9 antibody (BD Pharmingen, cat.#: 555372). A 20 µL of PE‐CD9 antibody was added to CD63^+^ EV solution and incubated in a sample shaker for 1 h, at room temperature (in the dark). To remove unbound CD9‐antibody, the sample was washed 4 times with BSA‐PBS isolation buffer using magnetic separators, and finally suspended in 200 µL of isolation buffer. The immobilized CD63^+^ EVs were acquired on a BD FACSLyric system (BD Biosciences) to detect CD9^+^ EVs. The data were analyzed using FlowJo software (TreeStar Inc.). The experiment was performed in biological duplicates.

### Examination of VEGF‐A Protein in EVs of LNP Treated Cells

The presence of VEGF‐A protein was examined in the EVs isolated from LNP‐treated and untreated cells using Human *VEGF‐A* sandwich ELISA Kit (cat.#: RAB0507, Sigma Aldrich, now Merck) according to manufacturer's instructions. 100 µL of EV solution or serially diluted VEGF‐A protein standards were added per well. VEGF‐A protein concentration (pg mL^‐1^) was recorded on ELISA reader instrument (Spectra max, 340 PC, molecular devices), where the VEGF‐A protein level was measured relative to *VEGF‐A* standard curve. The levels of VEGF‐A protein in EVs were normalized to total EV‐proteins (µg).

### qPCR and Detection of LNP‐Derived VEGF‐A mRNA in Cells and EVs

The total RNA from LNP‐mRNA treated HTB‐177, HUVECs, and CPCs and from their secreted EVs was isolated using miRNeasy Mini Kit (Qiagen, cat. #: 217004) according to the manufacturers’ guidelines. Total RNA was quantified by Qubit 2.0 fluorometer (Thermo Fisher Scientific) and NanoDrop 1000 (Thermo Fisher Scientific). The RNA quality was assessed by a 230/260 ratio recorded on NanoDrop. RNA samples from untreated cells and their EVs, and from empty LNP (without *VEGF‐A* mRNA) treated cells and their EVs were used as control.

LNP‐*VEGF‐A* mRNA in the cells and in their secreted EVs was detected and quantified by real time qPCR. 30–50 ng of total cellular total RNA were reverse transcribed into cDNA using high‐capacity cDNA kit with RNase inhibitor (Thermo Fisher Scientific: 4374966). 45 ng of cDNA was used for *VEGF‐A* mRNA quantification using hydrolysis probes (TaqMan probe assay, Thermo Fisher Scientific, assay ID: Hs00900055_m1) on ViiA 7 instrument according to the manufacturer's instructions. To generate the standard curve for absolute quantification, the *VEGF‐A* mRNA standards were prepared using pure *VEGF‐A* mRNA (TriLink Biotechnologies, USA). 2 µg of pure *VEGF‐A* mRNA was reverse transcribed into cDNA, and *VEGF‐A* cDNA was serially diluted ten‐fold (highest point: 100 ng, and lowest point: 0.0001 ng) to generate a standard curve. The assay was performed in technical triplicates. For the absolute quantification of *VEGF‐A* mRNA, the cellular and EV cDNA was interpolated against the *VEGF‐A* standard curve with minimal *R* ^2^ > 0.975. GAPDH gene (Thermo Fisher Scientific assay ID: Hs02758991_g1) was used as internal control.

### Kinetics of LNP‐VEGF‐A mRNA in Recipient Cells and EVs

To examine the kinetics of LNP‐mRNA in cells, the HTB‐177 cells were cultured, treated, and analyzed for different time points. Cells were treated with 30 µL of LNP‐*VEGF‐A* mRNA (containing 3 µg of *VEGF‐A* mRNA). The supernatants and cells were harvested after 0 h (untreated), 0.5 h, 1 h, 5 h, and 24 h. EVs were isolated from each time point. The total RNA from cells and EVs was isolated separately. To observe the peak of *VEGF‐A* against different time points, the *VEGF‐A* mRNA was quantified both in the cells and EVs using qPCR as mentioned above.

### Detection of VEGF‐A mRNA in Cells and EVs after Washing of LNP‐VEGF‐A mRNA

To confirm that *VEGF‐A* mRNA from LNPs is endocytosed and is detected inside cells and is not quantified as a contaminant from supernatants, the LNPs were removed after 1 h of incubation with HTB‐177 cells. Briefly, the cultured media containing remaining LNPs (which had not taken up by cells), was removed and the cells (flasks) were washed with PBS twice and the fresh media (without LNPs) was added to the cells and incubated for 24 h. The cells and cultured supernatants were collected, and EVs were isolated. *VEGF‐A* mRNA was analyzed in the cells as well as in their secreted EVs by qPCR as mentioned above. Untreated cells were used as control.

### Detection of VEGF‐A Protein in Cells Treated with LNP‐VEGF‐A mRNA

It was further investigated whether the LNP‐delivered *VEGF‐A* mRNA is translated into VEGF‐A protein inside cells, and then the protein is secreted outside cells. 24 h post LNP‐*VEGF‐A* mRNA treatment, cells and the conditioned media were collected and centrifuged at 3000 × *g* for 10 min at 4 °C on a 4K15 centrifuge (Sigma Aldrich) to remove cell debris and transferred to new tubes and saved for ELISA. Cell lysates were generated using 500 µL of M‐PER Mammalian Protein Extraction (lysis) Reagent (Thermo Fisher Scientific cat.#: 78503) containing 1% halt protease inhibitor cocktail, EDTA free (Thermo Fisher Scientific, cat.#: 87785). The cells with lysis reagent were gently agitated on a 3D Bio‐rocker for 10 min at 4 °C and centrifuged at 12 000 × *g* for 5 min to pellet the cell debris. The upper phase (containing cellular proteins) was transferred to a new tube, and the pellet was discarded. Total proteins from the conditioned media and the cell lysates were quantified by Qubit 2.0 fluorometer (Thermo Fisher Scientific). Untreated cells, and the conditioned media from the untreated cells were used as controls.

VEGF‐A protein was quantified in both conditioned media and cell lysates using Human *VEGF‐A* sandwich ELISA Kit (cat.#: RAB0507, Sigma Aldrich, now Merck) according to manufacturer's instructions. 100 µL of each conditioned media, cell lysate protein solution or serially diluted VEGF‐A protein standards was added per well. VEGF‐A protein concentration (pg mL^‐1^) was recorded on ELISA reader instrument (Spectra max, 340 PC, molecular devices). The levels of VEGF‐A protein in the conditioned media and the cells were normalized to total proteins of supernatants and cell lysates, respectively.

### Confocal Microscopy Analysis of EV‐Mediated Delivery of Translatable eGFP mRNA to Human Epithelial Cells

Further, it was investigated whether EVs can deliver (transport) the LNP‐mRNA to other cells and whether the delivered mRNA is translated into a corresponding protein in recipient cells. First, Cy5‐*eGFP* mRNA was incorporated into EVs. Briefly, the LNPs containing Cy5‐*eGFP* mRNA (100 µg) were delivered to HTB‐177 cells (1 × 10^6^/T75 flask) as described above. After 24 h of treatment, EVs were isolated from cultured media of LNP treated cells.

100 µg of EVs containing Cy5‐*eGFP* mRNA were delivered to HTB‐177 cells, in vitro. The cellular uptake of EVs containing Cy5‐*eGFP* mRNA was examined by detecting Cy5‐*eGFP* mRNA using confocal microscopy, whereas the translation of *eGFP* mRNA into protein was examined by detecting enhanced green fluorescent (eGFP) protein. Briefly, 24 h after EV delivery, the cells were washed in PBS and fixed using 3.7% formaldehyde in PBS followed by a 0.5% Triton X‐100 permeabilization step. Fixed cells were incubated 5 min in a 300 × 10^‐9^ m DAPI stain solution for nuclear staining. Images were acquired on a Zeiss LSM 700 confocal microscope and analyzed by ZEN software. Untreated cells were used as control.

### EV‐Mediated Delivery of Translatable VEGF‐A mRNA to Human Epithelial Cells In Vitro

After detecting a translatable LNP‐mRNA delivery to cells via EVs, it was investigated whether EVs could also deliver functional mRNA to cells. HTB‐177, HUVECs, and CPCs were cultured as described above, and *VEGF‐A* mRNA was incorporated into EVs. Briefly, after 24 h adaptation period, cells were treated with 50 µL of LNPs containing 3 µg *VEGF‐A* mRNA. Then 24 h posttreatment, EVs were isolated from culture media by ultracentrifugation method and *VEGF‐A* in EVs was quantified by qPCR as described above. The EVs containing 60 ng of *VEGF‐A* mRNA (per replicate) were delivered to HUVECs. The production of VEGF‐A protein from exogenously delivered *VEGF‐A* mRNA was investigated in the conditioned medium and cell lysates using Human *VEGF‐A* sandwich ELISA Kit. Untreated cells were used as controls.

### Angiogenesis Assay

After confirming the EV delivery of *VEGF‐A* mRNA in vitro and detecting VEGF‐A protein, it was further examined whether EVs containing *VEGF‐A* mRNA had any functional effects in vitro. EV‐*VEGF‐A* mRNA was delivered into an angiogenesis coculture assay in vitro (V2a, Caltag Medsystems Ltd, Buckingham, UK, discontinued assay) according to the manufacturer's instructions, to investigate the functional effect of EV‐*VEGF‐A* mRNA on tube formation. Briefly, the co‐culture cells (HUVEC/Human dermal Fibroblasts) were rapidly thawed, centrifuged, and plated in 96‐well plates at 37 °C, 5% CO_2_ in a humidified atmosphere for 24 h. The isolated EVs containing 60 ng of exogenous *VEGF‐A* mRNA were added to each well and the plate was placed in an IncuCyte live‐content imaging system (Satorius GmbH, Germany), and images were automatically acquired in both phase and fluorescence every 6 to 12 h for 4 to 10 d at 4× (single image).

Tube formation over the 4‐10‐day assay was quantified using the Essen BioScience Angiogenesis Analysis Module. The fluorescent images were analyzed to generate a segmentation mask closely resembling the in vitro network. The mask was then refined to specifically identify tube‐forming events, and the kinetic response was plotted using the IncuCyte and GraphPad Prism software. At the end point, the cells were fixed and counterstained using DAPI (4′,6‐diamidine‐2′‐phenylindole dihydrochloride, Merck) for further automated High Content microscopy (ImageXpress).

### Intravenous Delivery of Luciferase Encoding mRNA via EVs or LNPs

Animal work was performed in accordance with the National Institute of Health (NIH) guidelines for use of experimental animals and the study protocol was approved by the Animal Ethics Committee at Gothenburg University (Gothenburg Ethical Review Board number EA 2194‐2019). Male C57BL/6Ncrl at 8–10 weeks of age and weight of ≈25 g were purchased from Charles River and housed on a 12 h light/12 h dark cycle, ambient temperature at 21–22 °C and 50% humidity. The EVs or LNPs carrying 1 µg Luciferase mRNA (FLuc‐mRNA, Trilink Biotechnologies) were injected to female C57bl/Ncr mice intravenously with a single dose. The animals were divided into 3 groups for and received the following treatment at 5 mL kg^‐1^: LNP‐FLuc‐mRNA, EV‐FLuc‐mRNA, or untreated (PBS). After 6 h of EV or LNP administration, luciferin (≈5 mL kg^‐1^ RediJect D‐Luciferin, Perkinelmer) was administrated intravenously. The mice were terminated 20 min after the luciferin administration and the organs were dissected and scanned with a IVIS Spectrum (PerkinElmer) within less than 5 minutes after termination. The total radiance was quantified and used as marker for translatable luciferase mRNA.

### Intramyocardial Injections of EV‐VEGF‐A mRNA, LNP‐VEGF‐A mRNA, and Naked VEGF‐A mRNA

After confirming the functionality of EV‐*VEGF‐A* mRNA in vitro, whether these EVs can deliver functional *VEGF‐A* mRNA in vivo and produce a VEGF‐A protein were examined.

Animal work was performed in accordance with the National Institute of Health (NIH) guidelines for use of experimental animals and the study protocol was approved by the Animal Ethics Committee at Gothenburg University (Gothenburg Ethical Review Board number Ea 001173‐2017). Male C57BL/6Ncrl mice at 10–12 weeks of age and weight of ≈25 g were purchased from Charles River and housed on a 12 h light/12 h dark cycle, ambient temperature at 21–22 °C and 50% humidity. On the day of the injections, mice were anesthetized with 2–3% Isoflurane mixed with oxygen, intubated, and connected to a ventilator. The mice were ventilated with air ≈800 mL min^‐1^ and oxygen ≈100 mL min^‐1^ (≈230 strokes min^‐1^ (MiniVent Ventilator for Mice (Model 845), Harvard Apparatus, Holliston, MA)). Core temperature was continuously monitored and maintained at 35–36.5 °C by a heating operating table and heating lamp controlled by rectal thermometer. Electrodes were inserted under the skin to register the heart rate and electrical activity (PharmLab, Paris, France). The mice were subjected to a left thoracotomy at the fourth intercostal space ≈2 to 3 mm to the left of the sternum. A rib spreader was used to keep the incision open. The pericardium was opened, the heart was held using an USP 8‐0 suture (Braun, Kronberg im Taunus, Germany) and 40 µL of EVs or LNPs (corresponding to 50 ng *VEGF‐A* mRNA) or PBS (no mRNA) treatments were injected in one single site in the myocardium of the left ventricle using an insulin syringe (Becton, Dickinson and Company, Franklin lakes, NJ). The chest was then closed, and the mice were monitored during continued maintenance of body temperature and ventilation until it regained consciousness and could be disconnected. The mice were sacrificed 6 h postinjection, and the heart, liver, and blood were collected. For the heart, the area of injection in the left ventricle was dissected and separated from the rest of the heart and snap‐frozen. The remaining parts of the heart (remote left ventricle, right ventricle, and atria) were snap‐frozen in a second tube and referred to as remote non‐injected area for further analysis.

The animals were divided into six groups for HTB‐EVs, HUVEC‐EVs, CPC‐EVs or LNPs loaded with *VEGF‐A* mRNA or naked *VEGF‐A mRNA* in citrate buffer and were injected in separate groups. Untreated naïve mice were used as controls. VEGF‐A protein was detected and quantified by ELISA which was performed separately on the injected area of the left ventricle and rest of the heart as well as the liver, and blood.

### Quantification of Human VEGF‐A Protein in Mouse Heart, Liver, and Plasma

Total protein from injected and noninjected heart areas as well as from liver was extracted using T‐PER Tissue Protein Extraction Reagent (Thermo Fisher Scientific Cat. 78510) in the presence of 1% halt protease inhibitor cocktail, EDTA free (Thermo Fisher Scientific, cat.#: 87785), following the manufacturer's instructions. Briefly, 20–70 mg of tissue was lysed in 250 µL of lysis reagent supplemented with proteases inhibitors (Thermo Fisher Scientific) in the Tissue LyserII (Qiagen) for 5 min at the maximum speed (30 Hz). The tissue lysates were centrifuged at 10 000 × *g* for 15 min at 4 °C to deplete tissue debris. The upper phase was transferred to a new tube and the pellet was discarded. The blood was centrifuged at 2000× g, for 5 min, 4 °C to collect the plasma. Total proteins from tissues, and plasma were quantified by Qubit 2.0 fluorometer (Thermo Fisher Scientific). Human VEGF‐A sandwich ELISA (Cat. #: RAB0507, Sigma Aldrich) was performed using 100 µL of total proteins from each tissue and plasma sample, according to the manufacturer's instructions. The amount of VEGF‐A protein (pg mL^‐1^) in each organ was normalized to the relative organ weight (g).

### Multiplex Cytokine Analysis in the Mice Heart Tissue

The plate, samples, and controls were prepared and used to detect the cytokine expression according to the manufacturer's protocol (V‐PLEX Plus Mouse Cytokine 19‐Plex, Meso Scale Discovery, Rockville, MD, USA). Briefly, the samples were prediluted 2×, and the standards were serially diluted to generate the standard curve. The Meso Scale Discovery (MSD) plate was washed three times with 200 µL wash buffer. The samples, standard curves, and QC's (50 µL each) were transferred to MSD plate and incubated for 2 h at room temperature (with gentle shaking). After the incubation period, the washing step was repeated. The detection antibody (25 µL) was added to plate and incubated for 2 h at room temperature (with gentle shaking). The washing step was repeated and 150 µL reading buffer was added and the values were recorded in MSD Sector Imager S600. The data calculations were conducted for all 10 spots. Assays were set up and run in a robotic assay system from Beckman Coulter including pipetting robot, shake incubator, washer, and MSD reader.

### Transcriptomic Analysis of EVs Secreted from LNP‐VEGF‐A mRNA Treated Cells

EVs were isolated from LNP‐*VEGF‐A* mRNA treated cells (HTB‐177, HUVECs, and CPCs) used in the current study. Total RNA was isolated from the EVs as mentioned above, and the RNA quality was assessed by Bioanalyzer 2100 instrument according to the manufacturer's instructions (Agilent, Santa Clara CA). The purity of the RNA quality was also assessed by a 230/260 ratio recorded on NanoDrop 1000 (Thermo Fisher Scientific).

### RNA‐Seq Analysis

Library construction was performed using Takara SMARTer Stranded Total RNA‐Seq Kit v2 – Pico Input Mammalian kit, which is specifically designed for very low input total RNA samples. Clustering was done by “cBot” and samples were sequenced on NovaSeq6000 (NovaSeq Control Software 1.7.5/RTA v3.4.4) a 151 nt (Read1)‐10nt(Index1)‐10nt(Index2)‐151nt(Read2) setup using “NovaSeqXp” workflow in “S4” mode flowcell. The Bcl to FastQ conversion was performed using bcl2fastq_v2.20.0.422 from the CASAVA software suite. The quality scale used was Sanger / phred33 / Illumina 1.8+. Processing of FASTQ files was carried out by the SciLifeLab National Genomics Infrastructure at the Uppsala Multidisciplinary Center for Advanced Computational Science, Sweden. The sequenced reads were quality controlled with the FastQC software and preprocessed with Trim Galore. The processed reads were then aligned to the reference genome of Homo sapiens (build GRCh38) with the STAR aligner. Read counts for genes were generated using the feature Counts library and normalized TPM values calculated with StringTie, and raw gene read counts were generated by Salmon. Technical documentation on the RNA‐seq pipeline can be accessed here: https://github.com/nf‐core/rnaseq. Raw and processed data are available for download at Gene Expression Omnibus (https://www.ncbi.nlm.nih.gov/geo/) accession number: GSE198486.

### Differential Expression Analysis

The raw gene count data generated from the Salmon tool including 60669 transcripts from 8 HTB samples, 8 HUVEC samples, and 6 CPC samples, were imported into R for bioinformatic analysis, and statistical testing for differential expression was carried out using the *DESeq2* R‐package.^[^ ^66^ ^]^ Filtering and normalization of the raw counts were performed for EVs of each cell line separately within the *DESeq()* function in the DESeq2 package. The Wald test was used for the identification of differentially expressed genes (DEGs). *P*‐values were adjusted for multiple testing using the Benjamin Hoch method and a false discovery rate (FDR) rate of ≤0.05 was considered statistically significant. A result table with log_2_ fold changes, *p*‐values, and adjusted *p*‐values was generated and used for creating graphs or heatmaps.

### Explorative Data Analysis

The gene expression dataset was further explored to investigate the transcriptional effect of the LNP treatment. The genes with no counts were filtered and the data were normalized for each cell line using the *varianceStabilizingTransformation()* function in the DESeq2 package. PCA plots were generated from top 1000 most variable genes using *plotPCA()* function and MA plots were generated using *plotMA()* function with a shrinkage estimator from *ashr* package^[^ ^67^ ^]^ to shrink logFC. The output from the logFC shrinkage was used for visualization and ranking of DEGs in MA plots and DEG‐expression heatmap. The top 500 most variable genes were selected, and samples were clustered and visualized using heatmaps to assess the reproducibility and quality of the experiment. Heatmaps were also used for visualization of the DEGs. The *pheatmap* R‐package was used to create the heatmaps with spearman rank correlation as distance measure.

### Venn Diagram

The top 2000 most highly expressed genes in the untreated control group and the LNP treated group, respectively, were compared using the *VennDiagram* R‐package^[^ ^68^ ^]^ and intersected with the list of DEGs from these two experimental groups to investigate the fraction of DEGs among the 2000 highest expressed genes.

### Expression Analysis of Angiogenesis Genes in Different EV Types

A total of 262 unique Ensembl gene IDs associated with angiogenesis (GO:0001525) were identified using *biomaRt* R package. Out of these genes, compared to the expression in the RNA‐seq dataset, 259 genes were expressed in HTB and HUVEC, while 255 genes were expressed in CPC. The expression of the genes associated with angiogenesis was visualized with heatmaps and bar plots.

### Statistical Analysis

The statistical analysis was performed by GraphPad Prism v.9 (GraphPad Software). The in vitro data were analyzed by the Mann‐Whitney U‐test or Kruskal‐Wallis test followed by the Dunn's multiple comparison test. The Friedman test followed by the Dunn's multiple comparison test was applied to compare LNPs with EVs delivery in vitro. For the in vivo data, the statistically significant differences were evaluated by the Kruskal‐Wallis test followed by the Dunn's multiple comparison test to compare *VEGF‐A* mRNA treated samples with untreated samples. Each statistical parameter applied for different datasets is mentioned in the individual figure legends. Additionally, the statistical analysis for differentially expressed genes was carried out using the *DESeq2* R‐package.^[^ ^66^ ^]^ Filtering and normalization of the raw counts were performed for each cell line separately within the DESeq() function in the DESeq2 package. The Wald test was used for the identification of differentially expressed genes. *P*‐values were adjusted for multiple testing using the Benjamin Hoch method. The differentially expressed genes with false discovery rate (FDR) rate of ≤0.05 and absolute log2 FC > 1 were considered statistically significant. A result table with log_2_ fold changes, *p*‐values and FDR‐adjusted *p*‐values was generated and used for creating graphs. The statistical tool used for each figure is described in the figure legends along with *p*‐values, where applicable.

## Conflict of Interest

The authors declare no conflict of interest.

## Author Contributions

Conceptualization: M.N. and H.V.; Methodology: M.N., S.H.‐H., B.T., H.G.‐K.G., Y.J., M.M., F.K., L.H., A.R., A.C., B.K., J.C., K.J., O.G., J.W., A.W.B., L.L., H.V.; Investigation: M.N., L.L., J.S., H.V.; Data analysis: M.N., B.T., M.S., L.L., J.S., H.V.; Validation: M.N. and H.V.; Visualization: M.N. and H.V.; Funding acquisition: H.V. and J.S.; Project administration: H.V.; Supervision: H.V.; Writing—original draft: M.N. and H.V. wrote the first draft with the input from all the coauthors. Writing—review & editing: M.N., S.H.‐H., L.L., J.S., and H.V. with the input from all the coauthors.

## Supporting information

Supporting Information

## Data Availability

The data that support the findings of this study are openly available in Qry at https://www.ncbi.nlm.nih.gov/geo, reference number 198486.
