# Supplementary material for: Lipid Nanoparticles Deliver the Therapeutic VEGFA mRNA In Vitro and In Vivo and Transform Extracellular Vesicles for Their Functional Extensions
Source: Adv Sci (Weinh). 2023 Feb 19;10(12):2206187. doi: 10.1002/advs.202206187 (PMC10131815; doi:10.1002/advs.202206187)
Supplement: Supplementary file 1 — Supporting Information [file ADVS-10-2206187-s001.pdf]

ADVANCED SCIENCE
Open Access

Supporting Information

for *Adv. Sci.*, DOI 10.1002/advs.202206187

Lipid Nanoparticles Deliver the Therapeutic VEGFA mRNA In Vitro and In Vivo and Transform Extracellular Vesicles for Their Functional Extensions

*Muhammad Nawaz, Sepideh Heydarkhan-Hagvall, Benyapa Tangruksa, Hernán González-King Garibotti, Yujia Jing, Marco Maugeri, Franziska Kohl, Leif Hultin, Azadeh Reyahi, Alessandro Camponeschi, Bengt Kull, Jonas Christoffersson, Ola Grimsholm, Karin Jennbacken, Martina Sundqvist, John Wiseman, Abdel Wahad Bidar, Lennart Lindfors, Jane Synnergren and Hadi Valadi**

Supporting information

Lipid Nanoparticles Deliver the Therapeutic VEGFA mRNA *in Vitro* and *in Vivo* and Transform Extracellular Vesicles for Their Functional Extensions

Muhammad Nawaz [1], Sepideh Heydarkhan-Hagvall [2,3], Benyapa Tangruksa [1,3], Hernán González-King Garibotti [2], Yujia Jing [4], Marco Maugeri [1,5], Franziska Kohl [6,7], Leif Hultin [8], Azadeh Reyahi [1], Alessandro Camponeschi [1], Bengt Kull [2], Jonas Christoffersson [2,3], Ola Grimsholm [1,9], Karin Jennbacken [2], Martina Sundqvist [1], John Wiseman [6], Abdel Wahad Bidar [6], Lennart Lindfors [4], Jane Synnergren [3,10], Hadi Valadi [1]*

Affiliations

[1] Department of Rheumatology and Inflammation Research, Institute of Medicine, Sahlgrenska Academy, University of Gothenburg, Gothenburg, 41346, Sweden

[2] BioPharmaceuticals R&D, Early Cardiovascular, Renal and Metabolism (CVRM), Bioscience Cardiovascular, AstraZeneca, 431 83, Gothenburg, Mölndal, Sweden

[3] Systems Biology Research Center, School of Bioscience, University of Skövde, SE-541 28 Skövde, Sweden

[4] Advanced Drug Delivery, Pharmaceutical Sciences, BioPharmaceuticals R&D, AstraZeneca, Gothenburg, 431 83 Mölndal, Sweden

[5] Safety Innovations, Clinical Pharmacology and Safety Sciences, R&D, AstraZeneca, Gothenburg, 431 83 Mölndal, Sweden

[6] BioPharmaceuticals R&D, Discovery Sciences, Translational Genomics, AstraZeneca, 431 83, Gothenburg, Mölndal, Sweden

[7] Department of Medical Biochemistry and Biophysics, Karolinska Institute, Solna, Stockholm, 171 77, Sweden

[8] BioPharmaceuticals R&D, Clinical Pharmacology and Safety Science, Imaging and Data Analytics, AstraZeneca, 431 83, Gothenburg, Mölndal, Sweden

[9] Institute of Pathophysiology and Allergy Research, Medical University of Vienna, 1090 Vienna, Austria

[10] Department of Molecular and Clinical Medicine, Institute of Medicine, Sahlgrenska Academy, University of Gothenburg, Gothenburg, 41345, Sweden

*Correspondence: Hadi Valadi hadi.valadi@gu.se

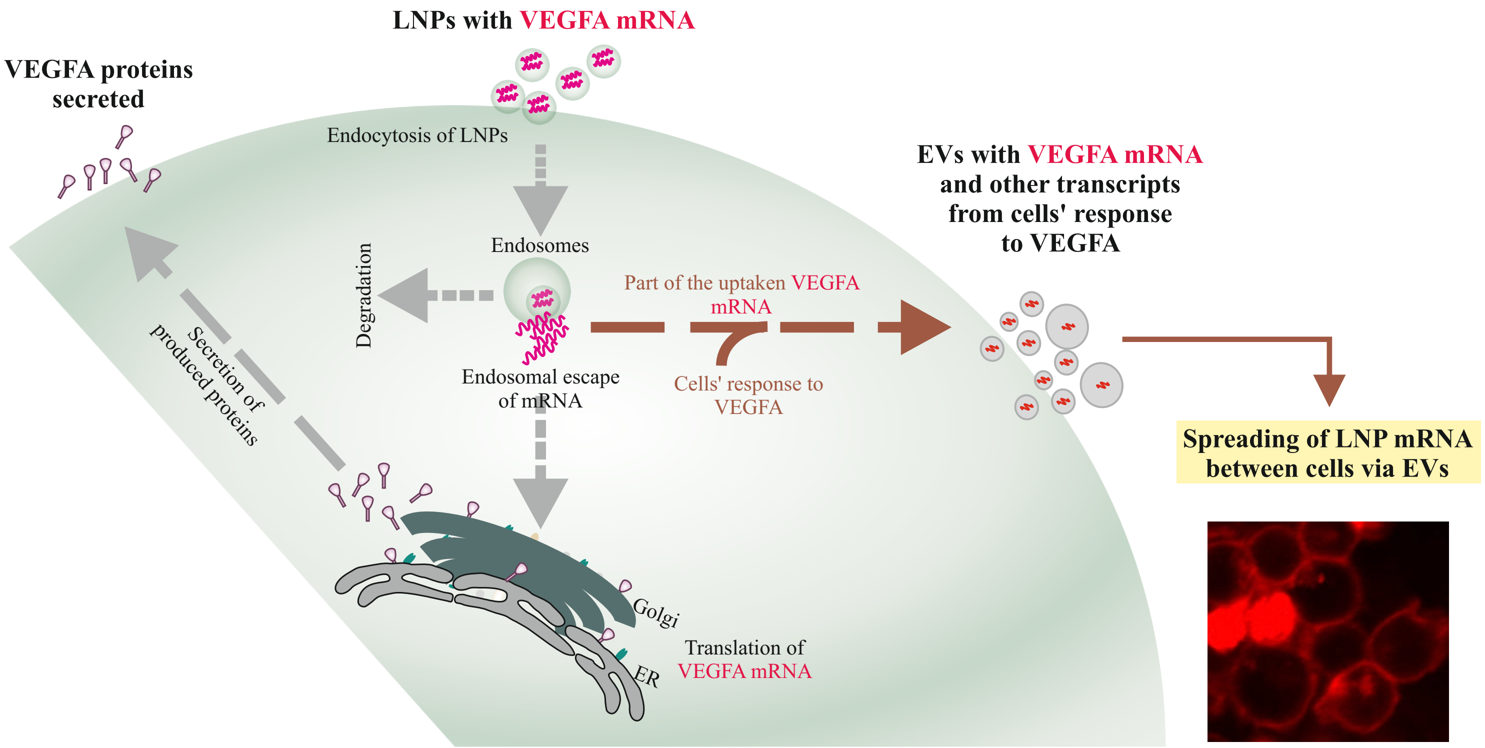
LNPs with VEGFA mRNA
VEGFA proteins secreted
Endocytosis of LNPs
Endosomes
Degradation
Secretion of produced proteins
Endosomal escape of mRNA
Part of the uptaken VEGFA mRNA
Cells' response to VEGFA
EVs with VEGFA mRNA and other transcripts from cells' response to VEGFA
Spreading of LNP mRNA between cells via EVs
Golgi
ER
Translation of VEGFA mRNA

ToC image

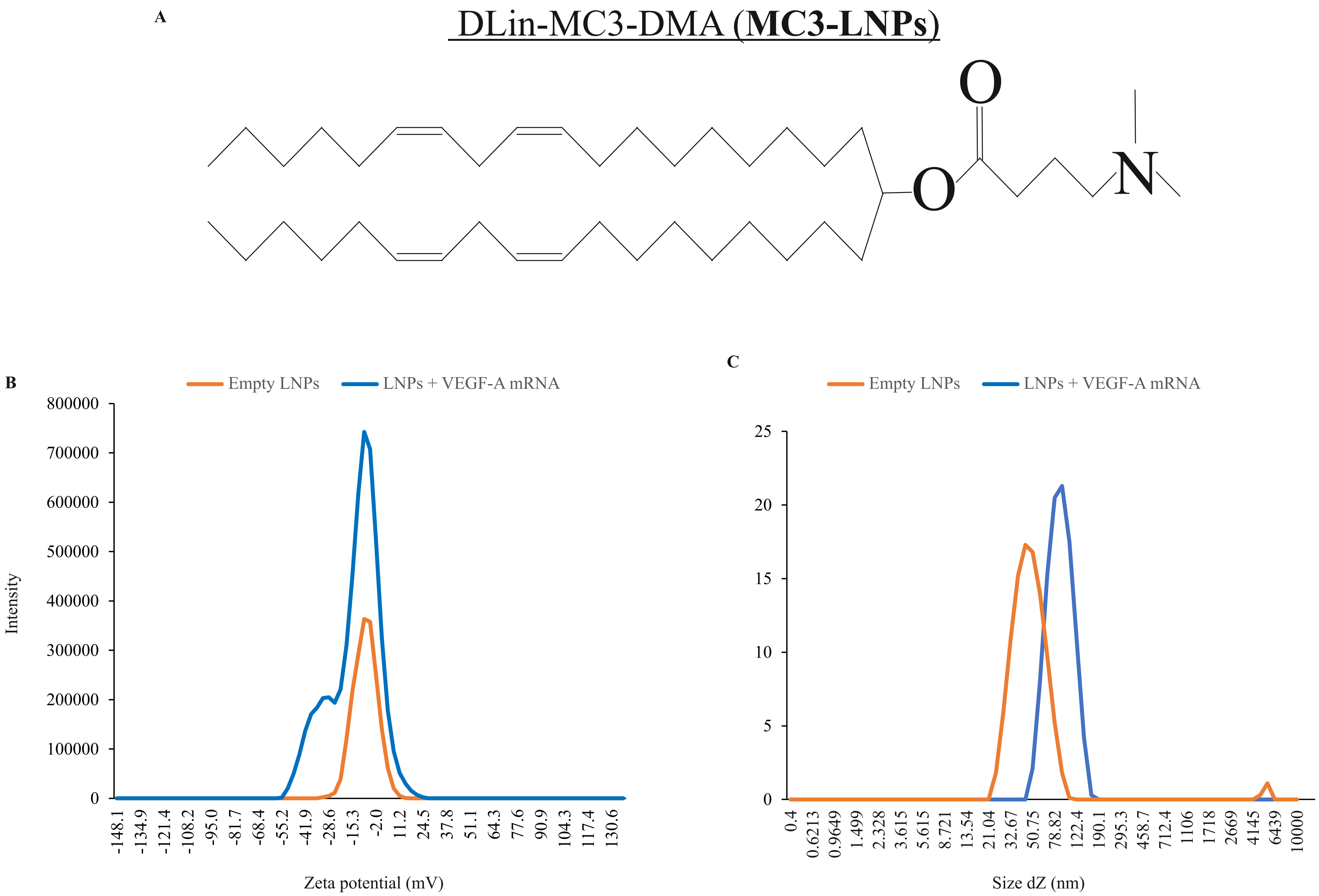
A
DLin-MC3-DMA (**MC3-LNPs**)
O
O
N
B
Empty LNPs
LNPs + VEGF-A mRNA
800000
700000
600000
500000
400000
300000
200000
100000
0
Intensity
-148.1 -134.9 -121.4 -108.2 -95.0 -81.7 -68.4 -55.2 -41.9 -28.6 -15.3 -2.0 11.2 24.5 37.8 51.1 64.3 77.6 90.9 104.3 117.4 130.6
Zeta potential (mV)
C
Empty LNPs
LNPs + VEGF-A mRNA
25
20
15
10
5
0
0.4 0.6213 0.9649 1.499 2.328 3.615 5.615 8.721 13.54 21.04 32.67 50.75 78.82 122.4 190.1 295.3 458.7 712.4 1106 1718 2669 4145 6439 10000
Size dZ (nm)

Suppl. Fig. 1

Supplementary Fig. 1: (A) The chemical structure of Dlin-MC3-DMA lipid nanoparticles (MC-LNPs) used in the current study. **(B)** Zeta potential distribution of MC3-LNPs (containing Dlin-MC3-DMA/Chol/DSPC/DMPE-PEG) with and without *VEGF-A* mRNA measured in 5 mM phosphate, pH 7.4. The zeta potential value for the *VEGF-A* mRNA loaded LNPs, is -18.0 mV. The zeta potential for the LNPs without *VEGF-A* mRNA (empty LNPs), is -7.3 mV. Each value is averaged from 5 replicated measurements. Since the PEG lipid used in LNPs carries a net negative charge, therefore zeta potential values are in negative. **(C)** Size distribution of MC3-LNPs LNPs with and without *VEGF-A* mRNA used in the current study. Empty LNPs: LNPs without mRNA.

A

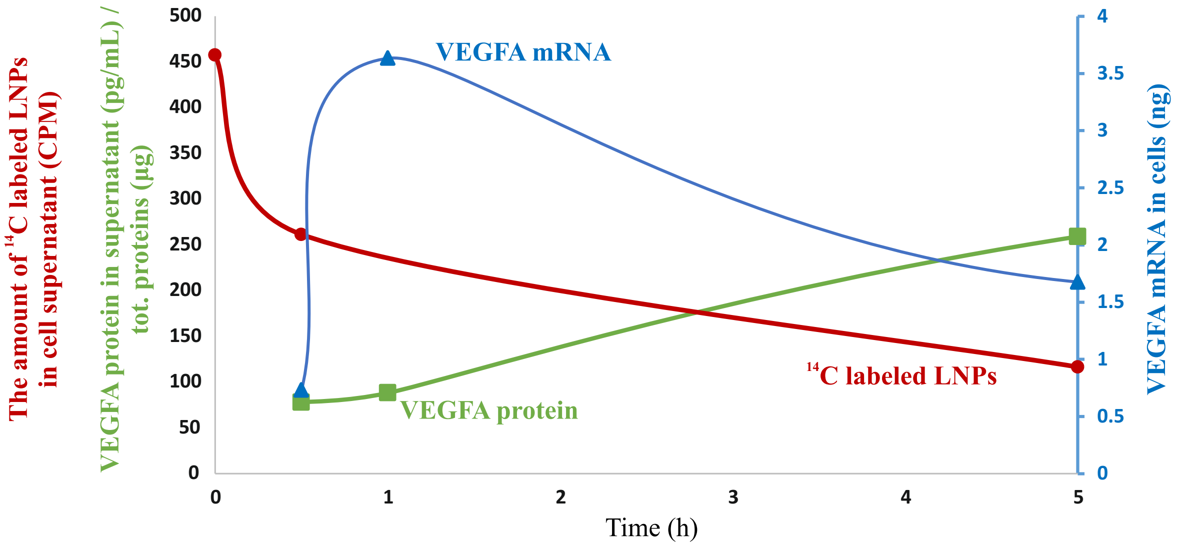

B

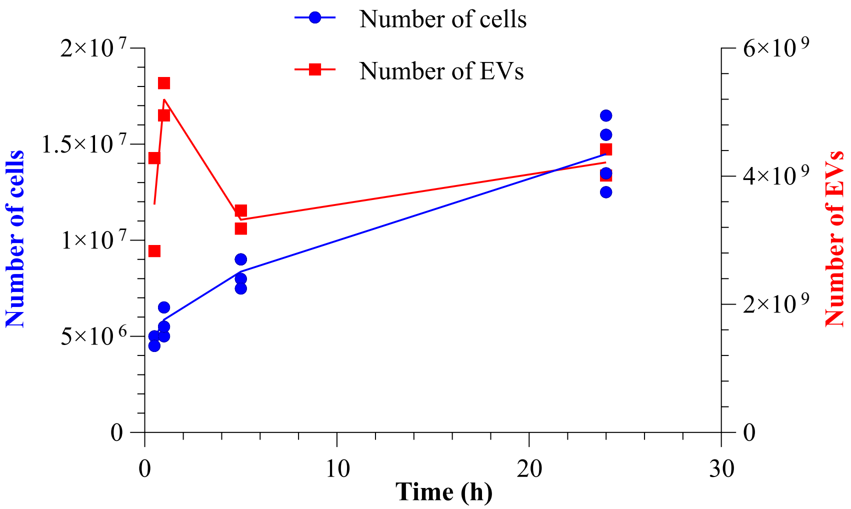

C

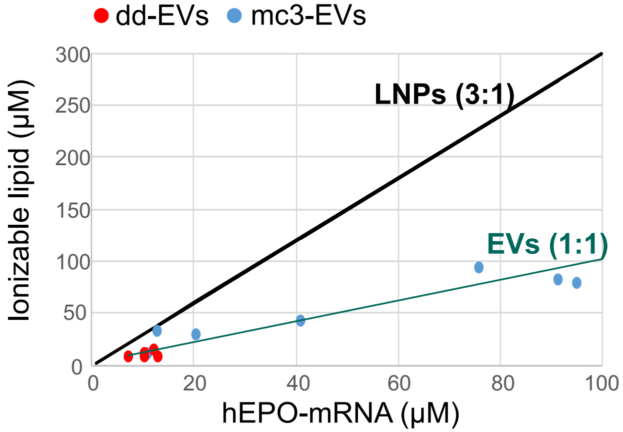

Suppl. Fig. 2

Supplementary Fig. 2: (A) The relationships between lipid nanoparticle (LNP) uptake by cells, LNP-mRNA detection in cells, and time-dependent translation of VEGF-A mRNA into VEGF-A protein. The Y-axis on the left side represents uptake of 14C-labeled LNPs and VEGF-A protein levels in supernatants. The Y-axis on the right side represents VEGF-A mRNA in cells. CPM, count per minute. **(B)** Number of cells and EVs corresponding to time points at which VEGF-A mRNA copy numbers were quantified. **(C)** Stoichiometric content (molar ratio) of ionizable lipids per mRNA nucleotides present in LNPs and EVs. The EVs were isolated from two different LNPs, i.e. "mc3-EVs" that were isolated from cells treated with LNPs formulated with DLin-MC3-DMA ionizable lipids, and "dd-EVs" that were isolated from cells treated with LNPs formulated with DLin-DMA ionizable lipids. Red circles (dd-EV) and blue circles (mc3-EV). Ionizable lipids and hEPO-mRNA of mc3-EVs ($n = 7$) each. Ionizable lipids and hEPO-mRNA of dd-EVs ($n = 6$) each. Both LNPs contain modified hEPO mRNA (858 nucleotides) (5meC, Ψ) (Trilink). dd-EVs: the EVs secreted from cells treated with DLin-DMA ionizable lipids. mc3-EVs: the EVs secreted from cells treated with DLin-MC3-DMA ionizable lipids. Suppl. Fig. 2J originally published by Maugeri M. et al. [18].

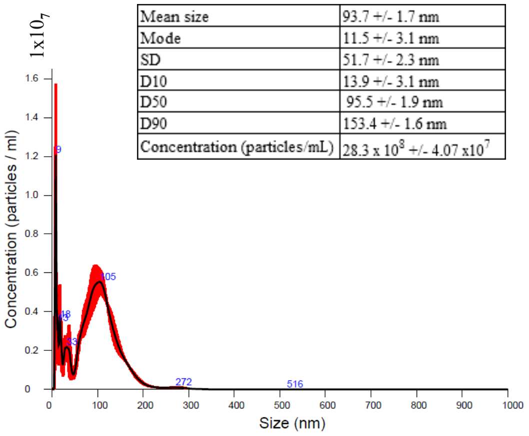

Mean size	93.7 +/- 1.7 nm
Mode	11.5 +/- 3.1 nm
SD	51.7 +/- 2.3 nm
D10	13.9 +/- 3.1 nm
D50	95.5 +/- 1.9 nm
D90	153.4 +/- 1.6 nm
Concentration (particles/mL)	28.3×10^8 +/- 4.07×10^7

0.5h, replicate 1

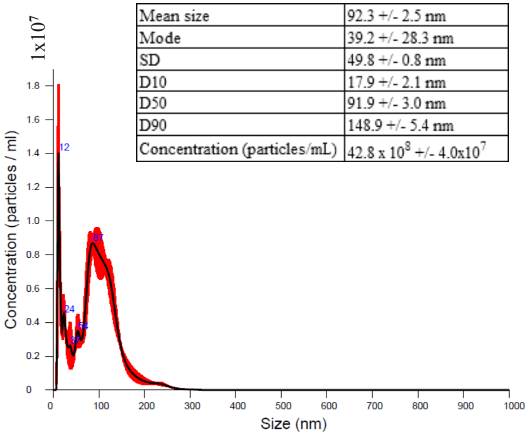

Mean size	92.3 +/- 2.5 nm
Mode	39.2 +/- 28.3 nm
SD	49.8 +/- 0.8 nm
D10	17.9 +/- 2.1 nm
D50	91.9 +/- 3.0 nm
D90	148.9 +/- 5.4 nm
Concentration (particles/mL)	42.8×10^8 +/- 4.0×10^7

0.5h, replicate 2

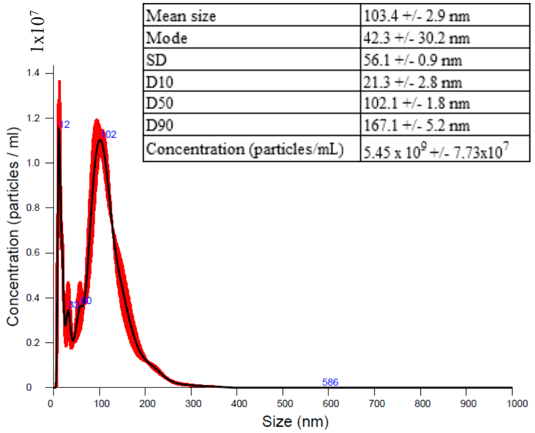

Mean size	103.4 +/- 2.9 nm
Mode	42.3 +/- 30.2 nm
SD	56.1 +/- 0.9 nm
D10	21.3 +/- 2.8 nm
D50	102.1 +/- 1.8 nm
D90	167.1 +/- 5.2 nm
Concentration (particles/mL)	5.45×10^9 +/- 7.73×10^7

1h, replicate 1

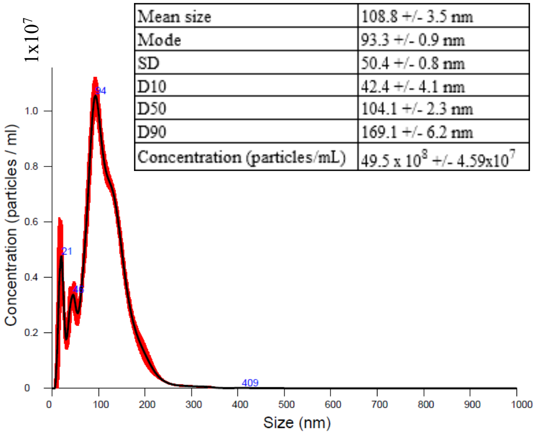

Mean size	108.8 +/- 3.5 nm
Mode	93.3 +/- 0.9 nm
SD	50.4 +/- 0.8 nm
D10	42.4 +/- 4.1 nm
D50	104.1 +/- 2.3 nm
D90	169.1 +/- 6.2 nm
Concentration (particles/mL)	49.5×10^8 +/- 4.59×10^7

1h, replicate 2

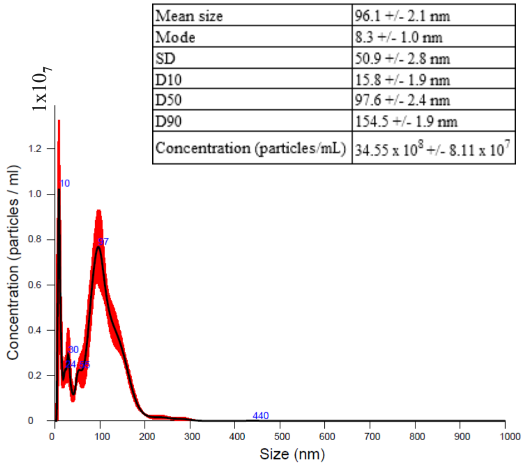

Mean size	96.1 +/- 2.1 nm
Mode	8.3 +/- 1.0 nm
SD	50.9 +/- 2.8 nm
D10	15.8 +/- 1.9 nm
D50	97.6 +/- 2.4 nm
D90	154.5 +/- 1.9 nm
Concentration (particles/mL)	34.55×10^8 +/- 8.11×10^7

5h, replicate 1

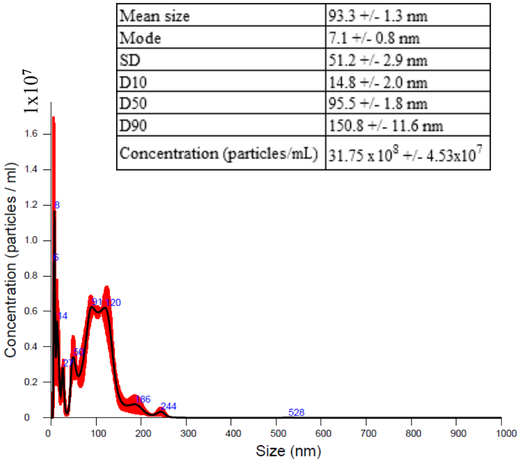

Mean size	93.3 +/- 1.3 nm
Mode	7.1 +/- 0.8 nm
SD	51.2 +/- 2.9 nm
D10	14.8 +/- 2.0 nm
D50	95.5 +/- 1.8 nm
D90	150.8 +/- 11.6 nm
Concentration (particles/mL)	31.75×10^8 +/- 4.53×10^7

5h, replicate 2

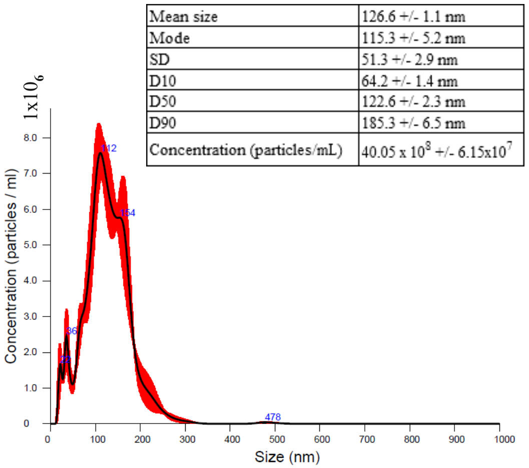

Mean size	126.6 +/- 1.1 nm
Mode	115.3 +/- 5.2 nm
SD	51.3 +/- 2.9 nm
D10	64.2 +/- 1.4 nm
D50	122.6 +/- 2.3 nm
D90	185.3 +/- 6.5 nm
Concentration (particles/mL)	40.05×10^8 +/- 6.15×10^7

24h, replicate 1

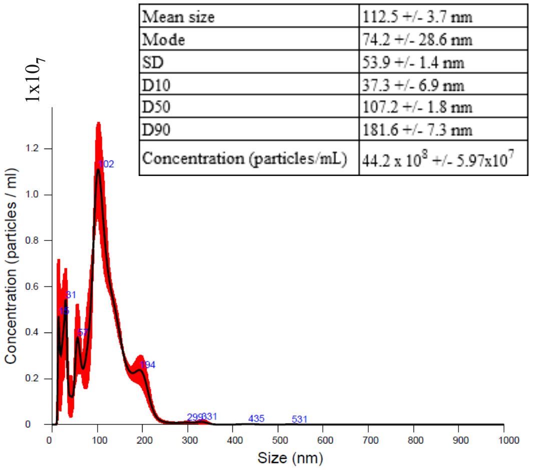

Mean size	112.5 +/- 3.7 nm
Mode	74.2 +/- 28.6 nm
SD	53.9 +/- 1.4 nm
D10	37.3 +/- 6.9 nm
D50	107.2 +/- 1.8 nm
D90	181.6 +/- 7.3 nm
Concentration (particles/mL)	44.2×10^8 +/- 5.97×10^7

24h, replicate 2

Supplementary Fig. 3: The size (nm) and concentration of extracellular vesicles (EVs, particle/mL) determined by Nanoparticle Tracking Analysis (NTA) at different time points (0.5 h, 1 h, 5 h, and 24 h). The particle concentration in the graphs represent the values recorded after EV dilution. However, their adjusted concentration is given in the tables.

A
B
C

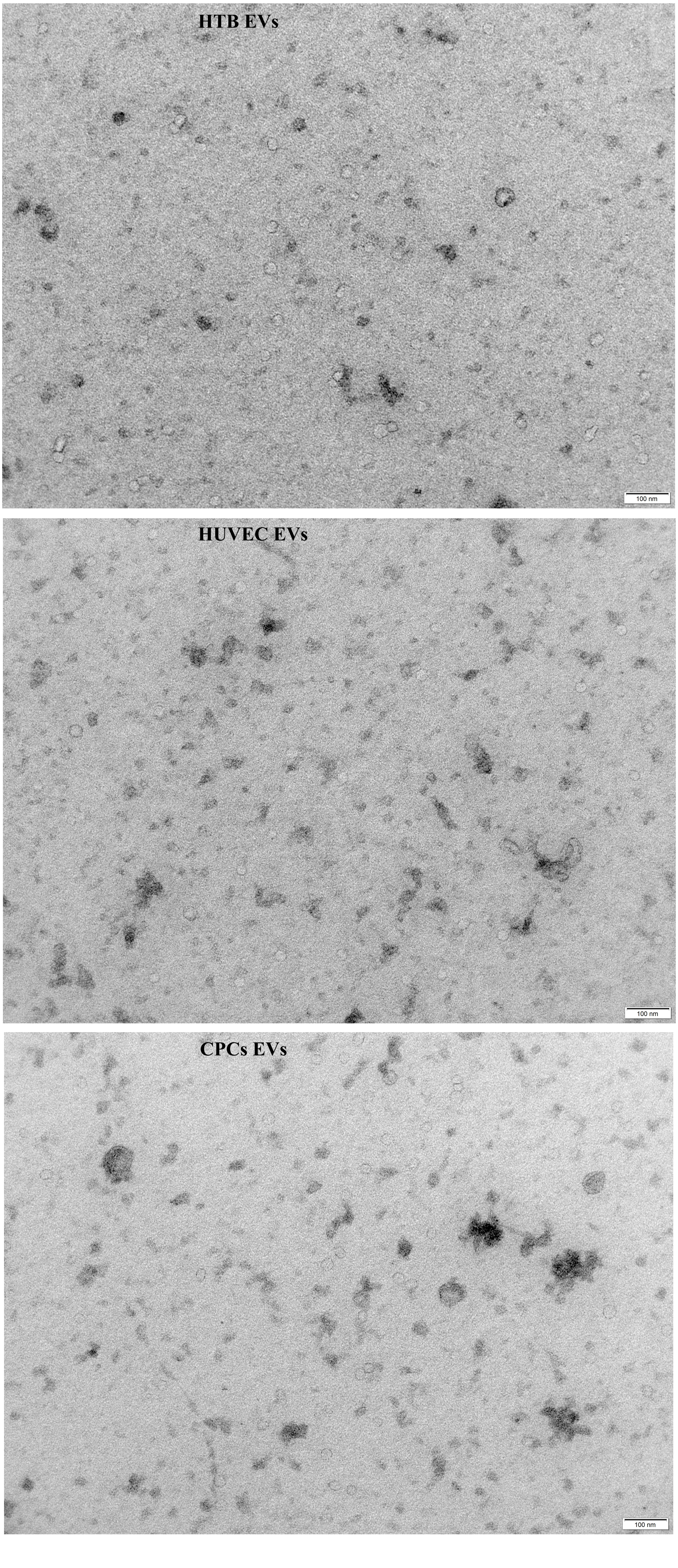

Suppl. Fig. 4

Supplementary Fig. 4: Transmission electron microscopy of **(A)** HTB-EVs, **(B)** HUVEC-EVs, and **(C)** CPC-EVs (n = 3). Scalebar: 100 nm.

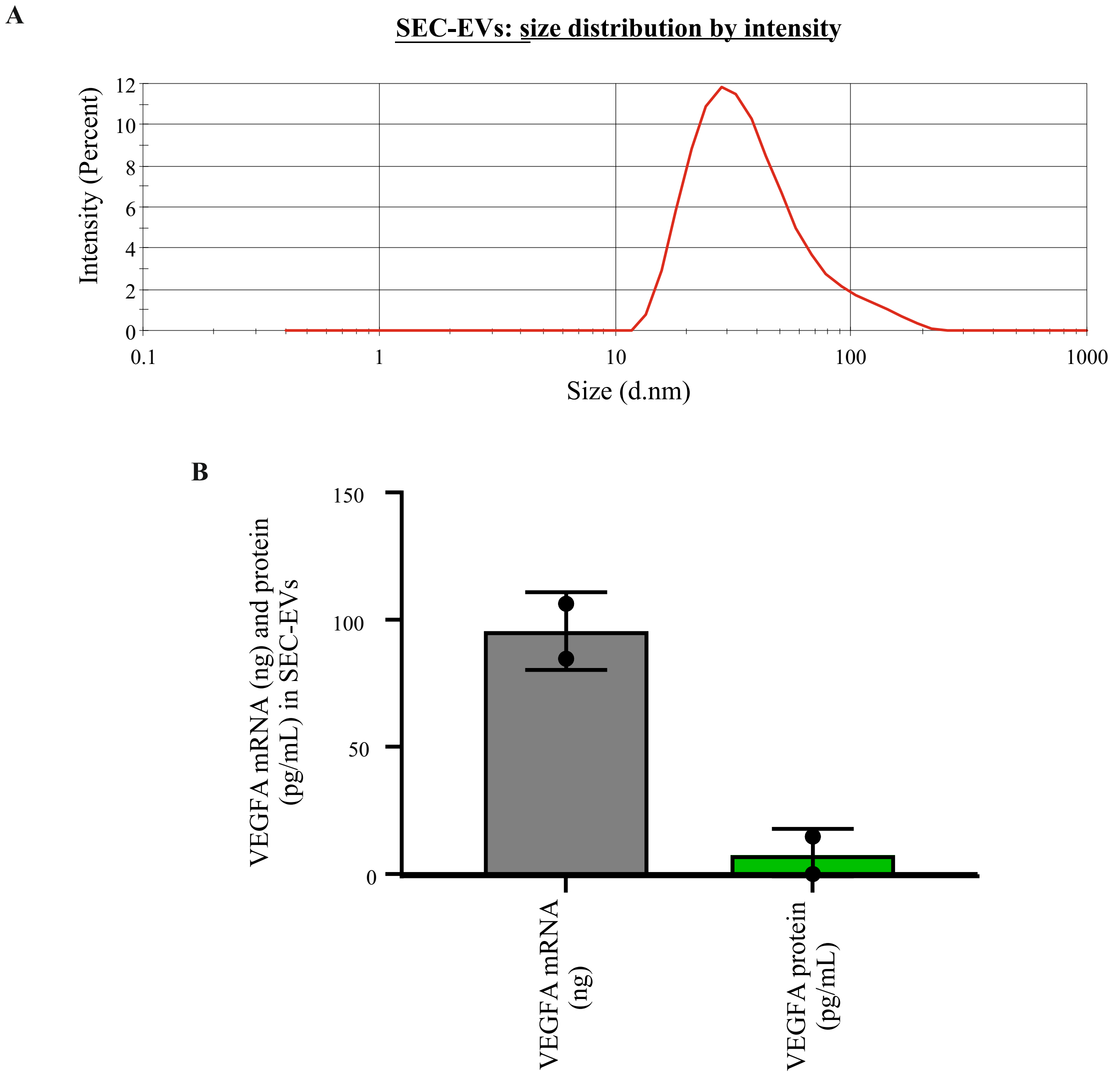

Supplementary Fig. 5: (A) Zetasizer-based size distribution of extracellular vesicles (EVs) isolated by size exclusion chromatography (SEC). **(B)** Detection of *VEGF-A* mRNA (by qPCR) and VEGF-A protein (by ELISA) in purified SEC-EV fractions.

UNT cells (cells w/o Cy5-eGFP-mRNA)

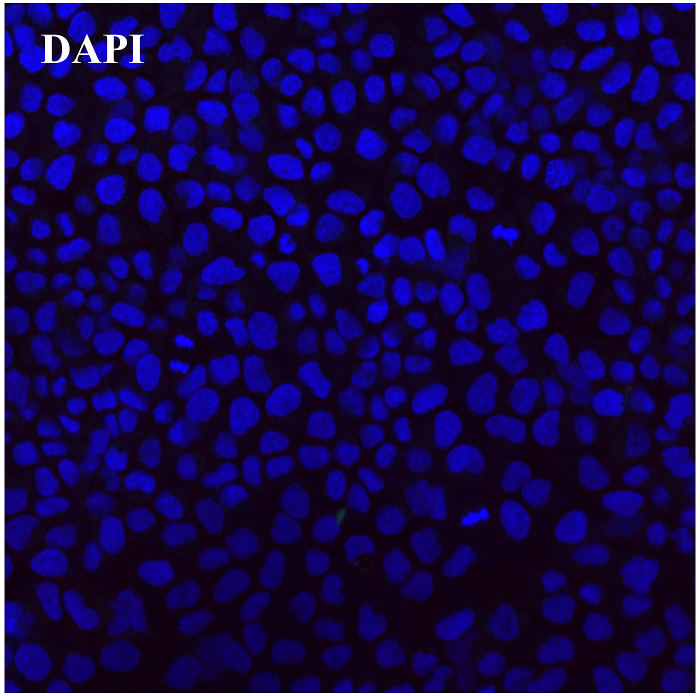

Supplementary Fig. 6: Examination of Cy5-*eGFP* mRNA and eGFP Protein in Untreated Cells

Control (untreated) HTB-177 cells (which did not receive extracellular vesicles (EVs) with Cy5-*eGFP* mRNA) were examined by confocal microscopy for the presence or absence of Cy5-*eGFP* or eGFP protein. The blue shades represent nuclei (DAPI stained), whereas no Cy5-*eGFP* mRNA or eGFP were detected. One representative image is shown. UNT, untreated; w/o, without.

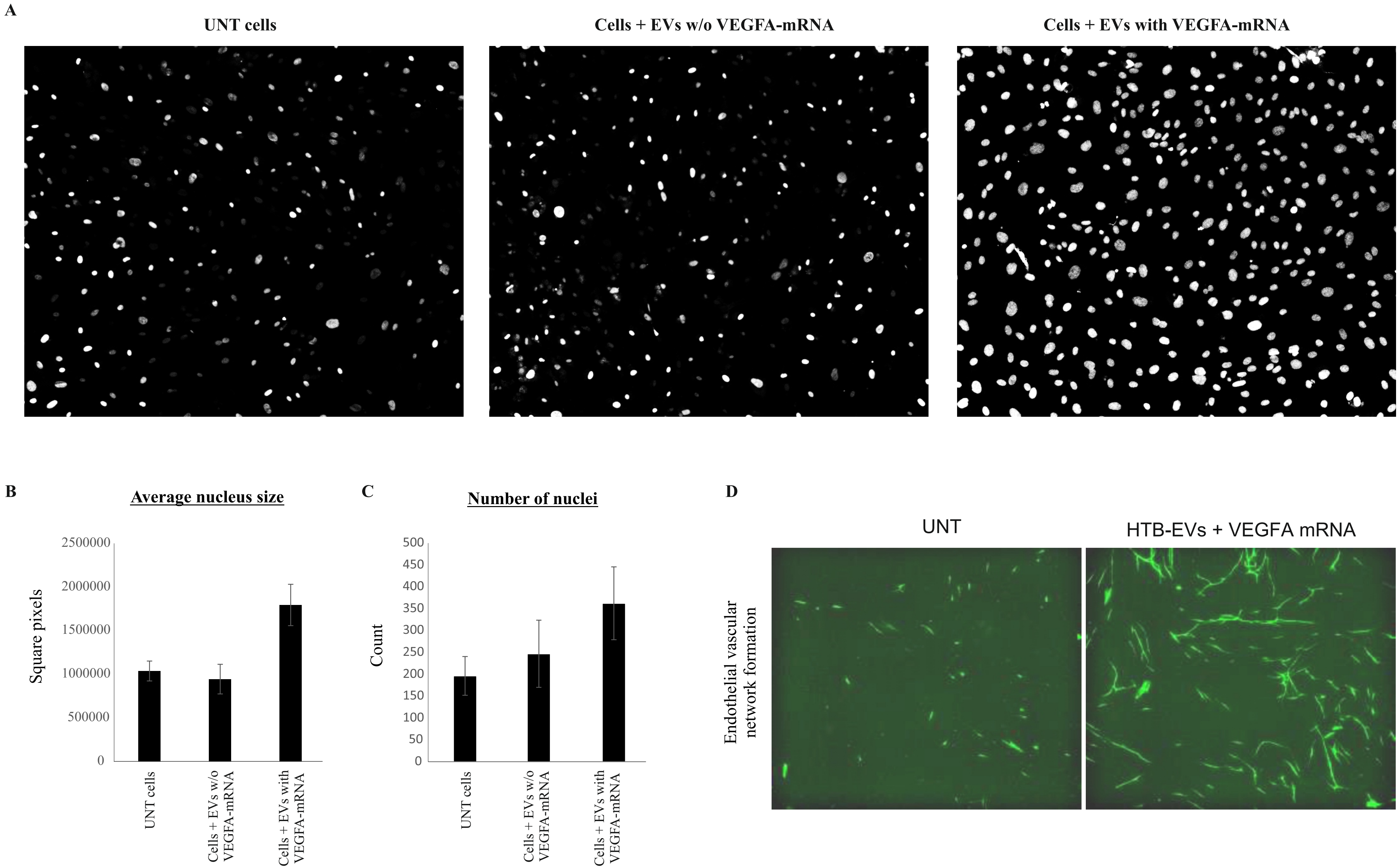
A
UNT cells
Cells + EVs w/o VEGFA-mRNA
Cells + EVs with VEGFA-mRNA
B
Average nucleus size
Square pixels
2500000
2000000
1500000
1000000
500000
0
UNT cells
Cells + EVs w/o VEGFA-mRNA
Cells + EVs with VEGFA-mRNA
C
Number of nuclei
Count
500
450
400
350
300
250
200
150
100
50
0
UNT cells
Cells + EVs w/o VEGFA-mRNA
Cells + EVs with VEGFA-mRNA
D
UNT
HTB-EVs + VEGFA mRNA
Endothelial vascular network formation

Suppl. Fig. 7

Supplementary Fig. 7: Proliferation of Endothelial Cells After *VEGF-A* mRNA Delivery via Extracellular Vesicles (EVs)

EVs carrying *VEGF-A* mRNA were delivered to HUVECs, and cell proliferation was assessed by measuring the number and size of nuclei. **(A)** Microscopic examination showing the morphological differences in cell nuclei numbers after delivering EVs containing *VEGF-A* mRNA, compared to EVs without *VEGF-A* mRNA or untreated cells (i.e., cells that did not receive any EVs). **(B)** Mean size of nuclei after delivery of *VEGF-A* mRNA via EVs, compared to controls (untreated cells or cells treated with EVs carrying no *VEGF-A* mRNA). **(C)** Quantification of the number of nuclei after delivery of *VEGF-A* mRNA via EVs, compared to controls. **(D)** Network (tube) formation in HUVECs after delivery of EVs containing *VEGF-A* mRNA. Untreated cells, and EVs without *VEGF-A* mRNA were used as controls. UNT, untreated; w/o, without.

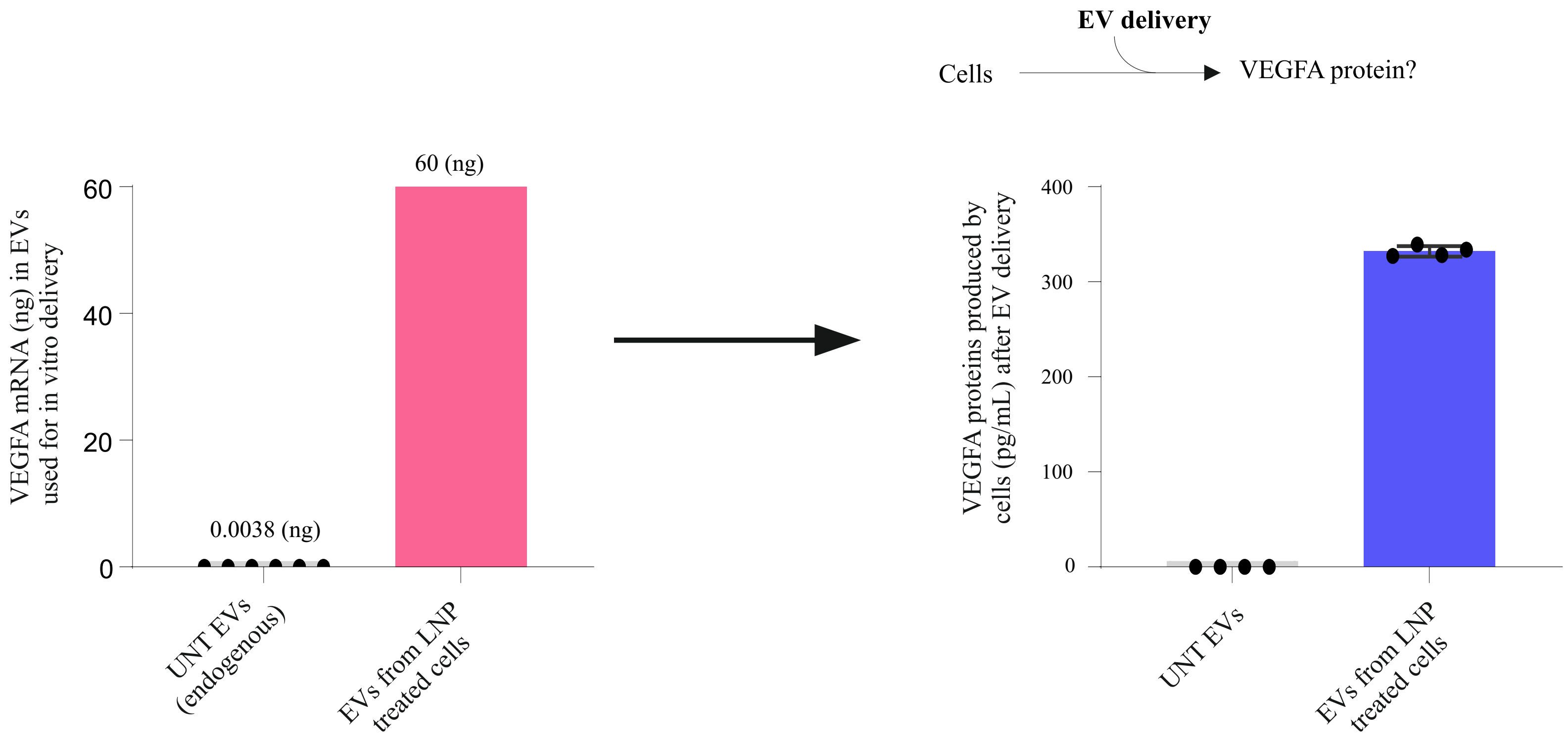

EV delivery
Cells
VEGFA protein?
60 (ng)
0.0038 (ng)
VEGFA mRNA (ng) in EVs used for in vitro delivery
60
40
20
0
UNT EVs (endogenous)
EVs from LNP treated cells
VEGFA proteins produced by cells (pg/mL) after EV delivery
400
300
200
100
0
UNT EVs
EVs from LNP treated cells

Suppl. Fig. 8

Supplementary Fig. 8: The Effect of Endogenous *VEGF-A* mRNA on Data Interpretation

A control experiment was performed to evaluate the interference of endogenous *VEGF-A* mRNA on data interpretation. *VEGF-A* mRNA was quantified in HTB-EVs secreted from untreated HTB-177 cells to estimate their endogenous amounts in EVs. The untreated EVs (EVs secreted from untreated cells) were delivered to HUVECs, and VEGF-A protein levels were quantified in recipient cells. EVs had only a minute amount of endogenous *VEGF-A* mRNA; likewise, the amount of newly produced VEGF-A protein in recipient cells was negligible. In contrast, the EV delivery of exogenous *VEGF-A* mRNA produced significantly higher amounts of VEGF-A protein. This data shows that the VEGF-A protein detected in recipient cells is produced from exogenously delivered *VEGF-A* mRNA.

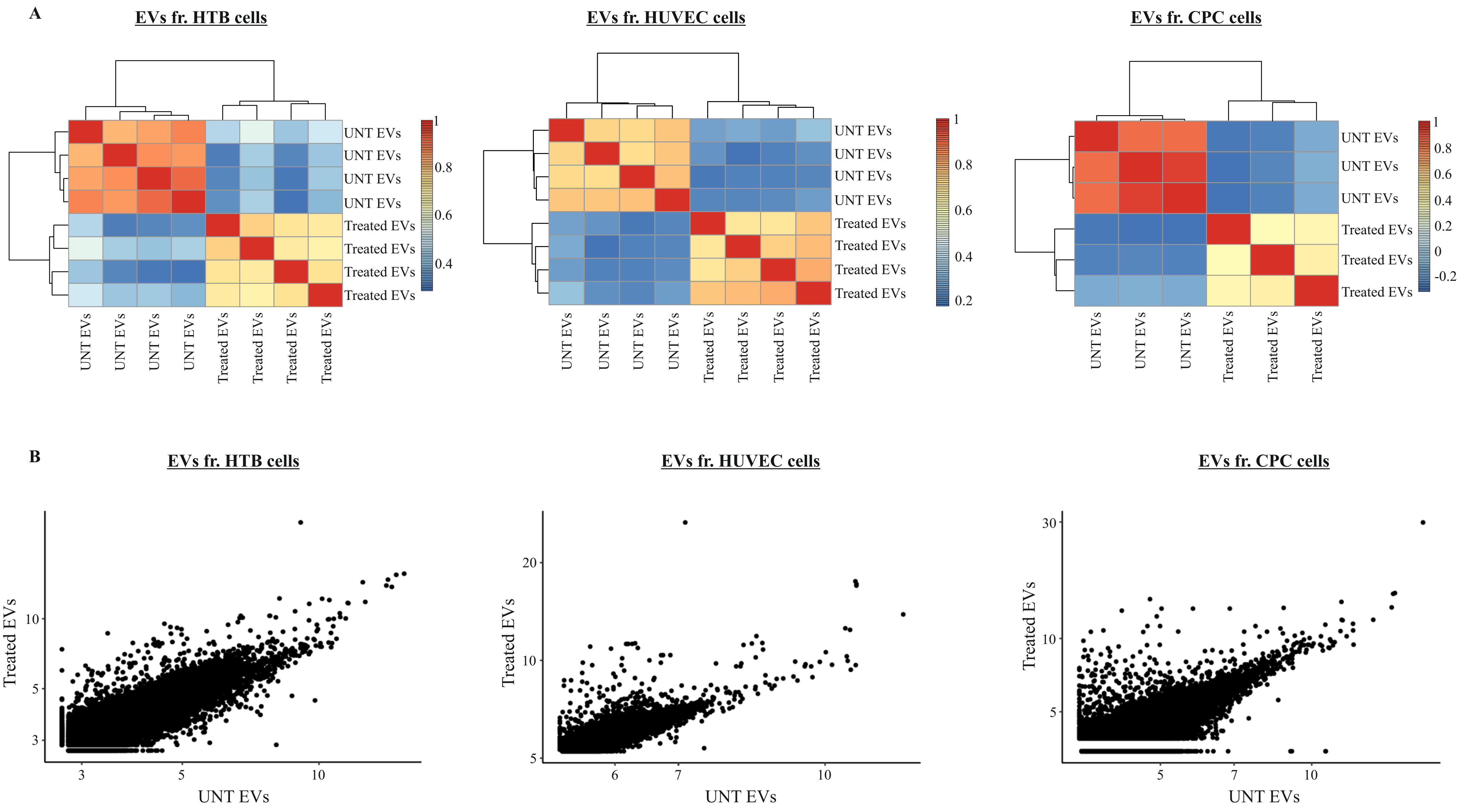
A
EVs fr. HTB cells
EVs fr. HUVEC cells
EVs fr. CPC cells
UNT EVs
Treated EVs
B
EVs fr. HTB cells
EVs fr. HUVEC cells
EVs fr. CPC cells
Treated EVs
UNT EVs

Suppl. Fig. 9

Supplementary Fig. 9: The Quality and Expression Assessment of RNA-Seq Data

(A) The clustered heat maps represent the sample correlation based on the normalized expression of the top 500 genes with the highest variance from LNP-treated and untreated HTB-, HUVEC-, and CPC-EVs. **(B)** Dot plots representing the mean normalized gene expression under the LNP-treated condition (Y-axis) against the untreated condition (X-axis) from HTB-, HUVEC-, and CPC-EVs. UNT, untreated; w/o, without.

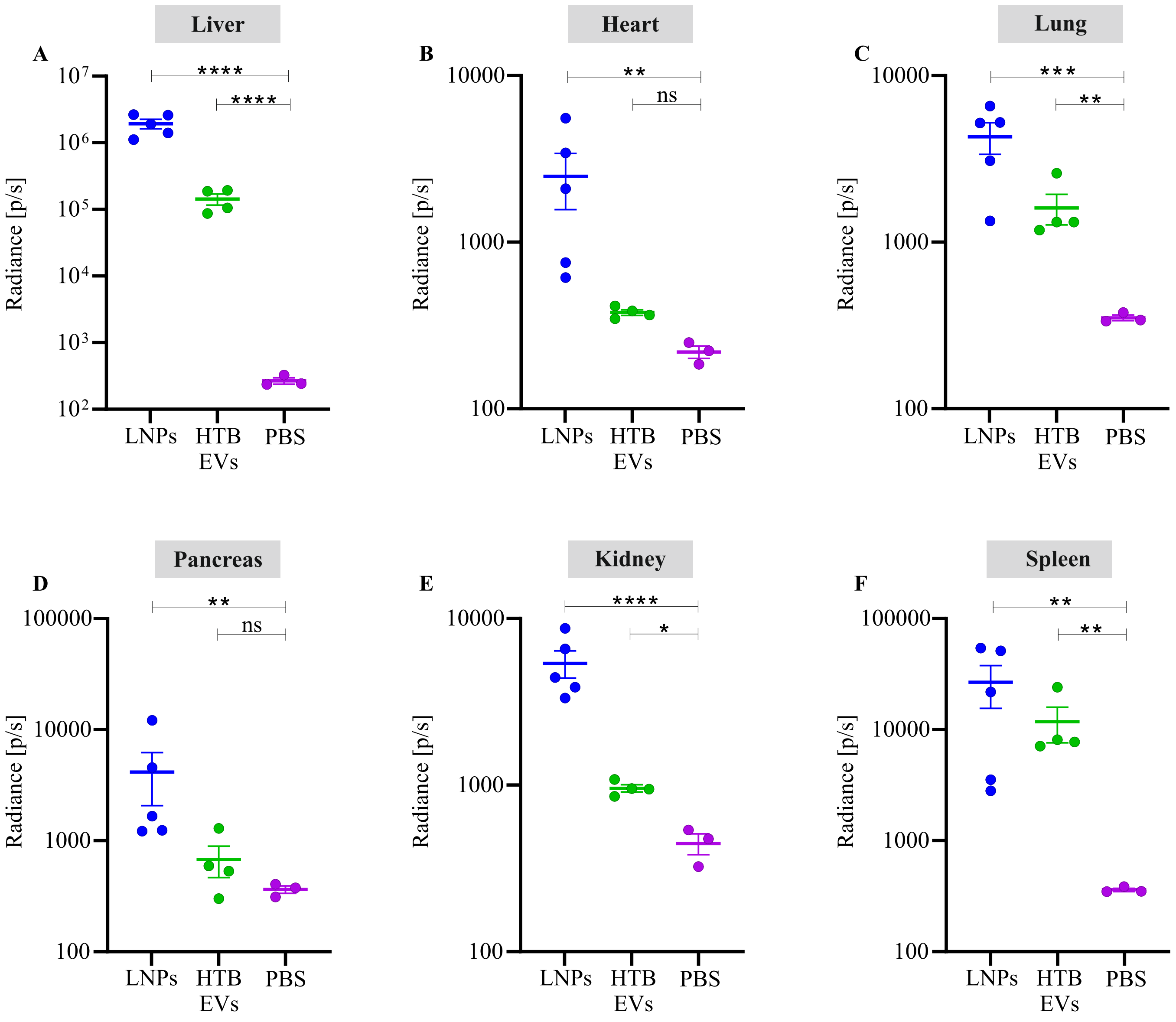
Liver
A
Radiance [p/s]
LNPs
HTB EVs
PBS
****
****
Heart
B
**
ns
Lung
C
***
**
Pancreas
D
**
ns
Kidney
E
****
*
Spleen
F
**
**

Suppl. Fig. 10

Supplementary Fig. 10: Intravenous Delivery of Luciferase mRNA via EVs or LNPs

EVs or LNPs containing 1 μg of luciferase mRNA (FLuc-mRNA) were administered intravenously to female C57bl/Ncr. After 6 hours of EV or LNP administration, luciferin (~5 mL/kg) was administrated intravenously. The mice were terminated 20 minutes after the luciferin administration and the organs were dissected and scanned with a IVIS Spectrum within less than 5 minutes after termination. The total radiance was quantified and used as marker for translatable luciferase mRNA. The amount of luciferase (activity) in **(A)** liver, **(B)** heart, **(C)** lung, **(D)** pancreas, **(E)** kidney, and **(F)** spleen. One-way ANOVA test was applied to compare LNP treated, or HTB-EV treated mice with untreated (PBS). n= 5. (*$p < 0.05$, **$p < 0.01$, ***$p < 0.001$, ****$p < 0.0001$).

Suppl. Table 1: Deregulated genes in extracellular vesicles secreted from HTB-177 cells after LNP treatment

Ensembl ID	Symbol	Entrez Gene Name	Location	Type(s)	Drug(s)	logFC	FDR
ENSG00000275700.4	AATF	apoptosis antagonizing transcription factor	Nucleus	transcription regulator		-1,136	2.91×10^-02
ENSG00000140798.15	ABCC12	ATP binding cassette subfamily C member 12	Cytoplasm	transporter		1,591	3.69×10^-02
ENSG00000143994.13	ABHD1	abhydrolase domain containing 1	Other	other		1,159	4.25×10^-02
ENSG00000175164.13	ABO	ABO, alpha 1-3-N-acetylgalactosaminyltransferase and alpha 1-3-galactosyltransferase	Plasma Membrane	enzyme		1,393	2.24×10^-04
ENSG00000120437.8	ACAT2	acetyl-CoA acetyltransferase 2	Cytoplasm	enzyme		-2,285	4.61×10^-03
ENSG00000131473.16	ACLY	ATP citrate lyase	Cytoplasm	enzyme	bempedoic acid/ezetimibe, bempedoic acid	-2,372	1.20×10^-02
ENSG00000122729.18	ACO1	aconitase 1	Cytoplasm	enzyme		-1,755	1.01×10^-02
ENSG00000075624.13	ACTB	actin beta	Cytoplasm	other		-1,897	7.58×10^-03
ENSG00000184009.9	ACTG1	actin gamma 1	Cytoplasm	other		-1,635	2.07×10^-02
ENSG00000136518.16	ACTL6A	actin like 6A	Nucleus	other		-2,428	4.74×10^-03
ENSG00000138071.13	ACTR2	actin related protein 2	Plasma Membrane	other		-1,795	2.44×10^-03
ENSG00000139567.12	ACVRL1	activin A receptor like type 1	Plasma Membrane	kinase	PF-3446962, panulisib, BMP type I receptor	-1,527	2.10×10^-02
ENSG00000156218.12	ADAMTSL3	ADAMTS like 3	Other	other		1,353	3.73×10^-03
ENSG00000105963.13	ADAP1	ArfGAP with dual PH domains 1	Nucleus	other		1,599	1.45×10^-02
ENSG00000185736.15	ADARB2	adenosine deaminase RNA specific B2 (inactive)	Nucleus	enzyme		1,198	1.85×10^-02
ENSG00000087274.16	ADD1	adducin 1	Cytoplasm	other		-1,221	7.38×10^-03
ENSG00000162618.12	ADGRL4	adhesion G protein-coupled receptor L4	Plasma Membrane	G-protein coupled receptor		-1,633	4.78×10^-02
ENSG00000156110.13	ADK	adenosine kinase	Nucleus	kinase	adenosine kinase inhibitor, pegintron/ribaviri	-2,981	4.23×10^-02
ENSG00000239900.11	ADSL	adenylosuccinate lyase	Cytoplasm	enzyme		-1,215	4.85×10^-02
ENSG00000157510.13	AFAP1L1	actin filament associated protein 1 like 1	Other	other		-2,315	1.21×10^-02
ENSG00000141385.9	AFG3L2	AFG3 like matrix AAA peptidase subunit 2	Cytoplasm	peptidase		-1,675	2.26×10^-02
ENSG00000124942.13	AHNAK	AHNAK nucleoprotein	Nucleus	other		-2,708	6.70×10^-07
ENSG00000100591.7	AHSA1	activator of HSP90 ATPase activity 1	Cytoplasm	other		-1,928	5.00×10^-03
ENSG00000131016.16	AKAP12	A-kinase anchoring protein 12	Cytoplasm	transporter		-2,276	9.66×10^-06
ENSG00000198074.9	AKR1B10	aldo-keto reductase family 1 member B10	Cytoplasm	enzyme		-1,486	2.45×10^-02
ENSG00000149925.16	ALDOA	aldolase, fructose-bisphosphate A	Cytoplasm	enzyme		-1,545	1.31×10^-02
ENSG00000166025.17	AMOTL1	angiomotin like 1	Plasma Membrane	other		-1,292	1.20×10^-02
ENSG00000091879.13	ANGPT2	angiopoietin 2	Extracellular Space	growth factor	AMG 780, CVX-060, nesvacumab, CVX-241	-1,666	9.13×10^-03
ENSG00000131503.20	ANKHD1/ANKHD1-EIF4EBP3	ankyrin repeat and KH domain containing 1	Cytoplasm	other		-1,358	3.63×10^-03
ENSG00000148677.6	ANKRD1	ankyrin repeat domain 1	Cytoplasm	transcription regulator		-1,291	3.00×10^-02
ENSG00000224309.7	ANKRD30BP2	ankyrin repeat domain 30B pseudogene 2	Other	other		1,904	4.04×10^-02
ENSG00000230006.7	ANKRD36BP2	ankyrin repeat domain 36B pseudogene 2	Other	other		1,187	1.06×10^-02
ENSG00000011426.10	ANLN	anillin actin binding protein	Cytoplasm	other		-2,828	4.95×10^-06
ENSG00000140350.15	ANP32A	acidic nuclear phosphoprotein 32 family member A	Cytoplasm	other		-2,138	2.23×10^-05
ENSG00000136938.8	ANP32B	acidic nuclear phosphoprotein 32 family member B	Nucleus	other		-2,134	1.86×10^-03
ENSG00000259790.1	ANP32BP1	acidic nuclear phosphoprotein 32 family member B pseudogene 1	Other	other		1,258	2.18×10^-02
ENSG00000143401.14	ANP32E	acidic nuclear phosphoprotein 32 family member E	Nucleus	other		-2,126	7.17×10^-04
ENSG00000166825.13	ANPEP	alanyl aminopeptidase, membrane	Plasma Membrane	peptidase	CNGRC peptide-TNF alpha conjugate	-3,331	3.77×10^-06
ENSG00000103254.9	ANTKMT	adenine nucleotide translocase lysine methyltransferase	Cytoplasm	enzyme		3,404	4.96×10^-02
ENSG00000182718.16	ANXA2	annexin A2	Plasma Membrane	other		-2,281	3.13×10^-05
ENSG00000164111.14	ANXA5	annexin A5	Plasma Membrane	transporter		-3,073	6.46×10^-06
ENSG00000197043.13	ANXA6	annexin A6	Plasma Membrane	ion channel		-2,196	7.46×10^-04
ENSG00000138279.15	ANXA7	annexin A7	Plasma Membrane	ion channel		-1,879	4.30×10^-02
ENSG00000100280.16	AP1B1	adaptor related protein complex 1 subunit beta 1	Cytoplasm	other		-1,79	9.22×10^-03
ENSG00000183020.13	AP2A2	adaptor related protein complex 2 subunit alpha 2	Cytoplasm	transporter		-1,519	1.63×10^-02
ENSG00000161203.13	AP2M1	adaptor related protein complex 2 subunit mu 1	Cytoplasm	other		-2,642	3.27×10^-05
ENSG00000077420.15	APBB1IP	amyloid beta precursor protein binding family B member 1 interacting protein	Cytoplasm	other		3,685	5.79×10^-04
ENSG00000100823.11	APEX1	apurinic/apyrimidinic endodeoxyribonuclease 1	Nucleus	enzyme	E 3330, lucanthone	-1,382	2.67×10^-02
ENSG00000166181.12	API5	apoptosis inhibitor 5	Cytoplasm	other		-1,797	2.36×10^-02
ENSG00000084234.16	APLP2	amyloid beta precursor like protein 2	Cytoplasm	other		-2,976	3.64×10^-09
ENSG00000142192.20	APP	amyloid beta precursor protein	Plasma Membrane	other	bapineuzumab, florbetapir F18, aducanumal	-3,547	1.21×10^-10
ENSG00000198576.3	ARC	activity regulated cytoskeleton associated protein	Cytoplasm	other		3,209	5.07×10^-09
ENSG00000095139.13	ARCN1	archain 1	Cytoplasm	other		-2,009	7.74×10^-04
ENSG00000143761.13	ARF1	ADP ribosylation factor 1	Cytoplasm	enzyme		-2,131	3.90×10^-02
ENSG00000198826.10	ARHGAP11A	Rho GTPase activating protein 11A	Cytoplasm	other		-1,865	2.34×10^-04
ENSG00000146376.10	ARHGAP18	Rho GTPase activating protein 18	Cytoplasm	other		-2,114	1.75×10^-02
ENSG00000160007.17	ARHGAP35	Rho GTPase activating protein 35	Nucleus	transcription regulator		-1,51	3.54×10^-03
ENSG00000111348.8	ARHGDIB	Rho GDP dissociation inhibitor beta	Cytoplasm	enzyme		-4,107	1.26×10^-05
ENSG00000102606.17	ARHGEF7	Rho guanine nucleotide exchange factor 7	Cytoplasm	other		-1,281	4.90×10^-02
ENSG00000117713.17	ARID1A	AT-rich interaction domain 1A	Nucleus	transcription regulator		-1,397	6.14×10^-03
ENSG00000141748.12	ARL5C	ADP ribosylation factor like GTPase 5C	Other	other		2,604	1.67×10^-03
ENSG00000241685.8	ARPC1A	actin related protein 2/3 complex subunit 1A	Extracellular Space	other		-2,999	7.30×10^-04
ENSG00000130429.12	ARPC1B	actin related protein 2/3 complex subunit 1B	Cytoplasm	other		-1,785	1.60×10^-02
ENSG00000163466.15	ARPC2	actin related protein 2/3 complex subunit 2	Cytoplasm	other		-1,273	1.31×10^-02
ENSG00000111229.15	ARPC3	actin related protein 2/3 complex subunit 3	Cytoplasm	other		-2,736	1.26×10^-05
ENSG00000153317.14	ASAP1	ArfGAP with SH3 domain, ankyrin repeat and PH domain 1	Plasma Membrane	other		-1,812	3.37×10^-03
ENSG00000239388.8	ASB14	ankyrin repeat and SOCS box containing 14	Cytoplasm	other		1,381	4.41×10^-02
ENSG00000112249.13	ASCC3	activating signal cointegrator 1 complex subunit 3	Cytoplasm	enzyme		-1,713	2.42×10^-02
ENSG00000141505.11	ASGR1	asialoglycoprotein receptor 1	Plasma Membrane	transmembrane receptor		1,506	3.89×10^-02
ENSG00000156802.12	ATAD2	ATPase family AAA domain containing 2	Nucleus	enzyme		-1,516	2.20×10^-02
ENSG00000111676.14	ATN1	atrophin 1	Nucleus	transcription regulator		-1,247	4.20×10^-02
ENSG00000163399.15	ATP1A1	ATPase Na+/K+ transporting subunit alpha 1	Plasma Membrane	transporter	digoxin, acetyldigitoxin derivative, trichlorom	-2,003	1.86×10^-03
ENSG00000143153.12	ATP1B1	ATPase Na+/K+ transporting subunit beta 1	Plasma Membrane	transporter		1,124	3.58×10^-02
ENSG00000152234.15	ATP5F1A	ATP synthase F1 subunit alpha	Cytoplasm	transporter		-1,55	8.96×10^-03
ENSG00000110955.8	ATP5F1B	ATP synthase F1 subunit beta	Cytoplasm	transporter		-2,121	3.49×10^-02
ENSG00000165629.19	ATP5F1C	ATP synthase F1 subunit gamma	Cytoplasm	transporter		-2,488	2.06×10^-02
ENSG00000167283.7	ATP5MG	ATP synthase membrane subunit g	Cytoplasm	enzyme		-1,777	1.30×10^-03
ENSG00000156411.9	ATP5MJ	ATP synthase membrane subunit j	Cytoplasm	other		-1,378	3.66×10^-02
ENSG00000116459.10	ATP5PB	ATP synthase peripheral stalk-membrane subunit b	Cytoplasm	transporter		-2,612	2.97×10^-03
ENSG00000241837.6	ATP5PO	ATP synthase peripheral stalk subunit OSCP	Cytoplasm	transporter		-1,319	4.60×10^-02
ENSG00000131100.12	ATP6V1E1	ATPase H+ transporting V1 subunit E1	Cytoplasm	transporter		-3,394	1.01×10^-02
ENSG00000128524.4	ATP6V1F	ATPase H+ transporting V1 subunit F	Cytoplasm	enzyme		-1,643	4.49×10^-02
ENSG00000136888.6	ATP6V1G1	ATPase H+ transporting V1 subunit G1	Cytoplasm	transporter		-1,826	4.65×10^-02
ENSG00000085224.20	ATRX	ATRX chromatin remodeler	Nucleus	transcription regulator		-1,496	3.20×10^-03
ENSG00000146776.14	ATXN7L1	ataxin 7 like 1	Other	other		1,018	2.31×10^-02
ENSG00000167601.11	AXL	AXL receptor tyrosine kinase	Plasma Membrane	kinase	CH5451098, gilteritinib, bosutinib/rituximab,	-2,826	1.36×10^-03
ENSG00000175711.8	B3GNTL1	UDP-GlcNAc:betaGal beta-1,3-N-acetylglucosaminyltransferase like 1	Other	enzyme		1,187	8.97×10^-03
ENSG00000151929.9	BAG3	BAG cochaperone 3	Cytoplasm	other		-1,597	8.51×10^-03
ENSG00000009954.10	BAZ1B	bromodomain adjacent to zinc finger domain 1B	Nucleus	transcription regulator		-1,606	1.52×10^-03
ENSG00000185825.15	BCAP31	B cell receptor associated protein 31	Cytoplasm	transporter		-2,307	1.10×10^-02
ENSG00000050820.16	BCAR1	BCAR1 scaffold protein, Cas family member	Plasma Membrane	enzyme		-1,753	8.37×10^-03
ENSG00000127152.17	BCL11B	BAF chromatin remodeling complex subunit BCL11B	Nucleus	transcription regulator		1,217	2.51×10^-02
ENSG00000129473.9	BCL2L2	BCL2 like 2	Cytoplasm	other	pelcitoclax, navitoclax, ABT-737	-1,323	1.20×10^-02
ENSG00000236824.1	BCYRN1	brain cytoplasmic RNA 1	Other	other		-1,818	3.77×10^-03
ENSG00000145734.18	BDP1	B double prime 1, subunit of RNA polymerase III transcription initiation factor IIIB	Nucleus	transcription regulator		-1,551	1.22×10^-03
ENSG00000166681.13	BEX3	brain expressed X-linked 3	Cytoplasm	other		-2,126	3.11×10^-04
ENSG00000182492.15	BGN	biglycan	Extracellular Space	other		-4,302	4.36×10^-03
ENSG00000089685.14	BIRC5	baculoviral IAP repeat containing 5	Cytoplasm	other	gataparsen, EZN 3042	-1,623	8.37×10^-03
ENSG00000165733.7	BMS1	BMS1 ribosome biogenesis factor	Nucleus	other		-1,263	1.66×10^-02
ENSG00000104765.14	BNIP3L	BCL2 interacting protein 3 like	Cytoplasm	other		-1,95	3.40×10^-02
ENSG00000145919.10	BOD1	biorientation of chromosomes in cell division 1	Nucleus	other		-3,79	4.35×10^-03
ENSG00000038219.12	BOD1L1	biorientation of chromosomes in cell division 1 like 1	Nucleus	other		-1,131	1.46×10^-02
ENSG00000169925.16	BRD3	bromodomain containing 3	Nucleus	kinase	BI 894999	-1,636	1.86×10^-02
ENSG00000112983.17	BRD8	bromodomain containing 8	Nucleus	transcription regulator		-1,205	4.54×10^-02
ENSG00000145741.15	BTF3	basic transcription factor 3	Nucleus	transcription regulator		-1,318	2.82×10^-02
ENSG00000082153.17	BZW1	basic leucine zipper and W2 domains 1	Cytoplasm	translation regulator		-1,523	3.56×10^-03
ENSG00000110696.9	C11orf58	chromosome 11 open reading frame 58	Other	other		-1,184	2.53×10^-02
ENSG00000180425.10	C11orf71	chromosome 11 open reading frame 71	Nucleus	other		1,563	2.50×10^-02
ENSG00000278615.4	C11orf98	chromosome 11 open reading frame 98	Other	other		-2,164	2.17×10^-02
ENSG00000235162.8	C12orf75	chromosome 12 open reading frame 75	Other	other		-2,26	2.01×10^-02
ENSG00000184208.10	C22orf46	CTA-216E10.6	Other	other		-1,882	1.09×10^-04
ENSG00000157782.9	CABP1	calcium binding protein 1	Plasma Membrane	other		1,154	7.76×10^-03
ENSG00000116161.17	CACYBP	calcyclin binding protein	Nucleus	other		-1,58	3.22×10^-03
ENSG00000164304.15	CAGE1	cancer antigen 1	Other	other		2,382	3.89×10^-03
ENSG00000143933.16	CALM1 (includes others)	calmodulin 1	Cytoplasm	other		-1,293	6.20×10^-03
ENSG00000160014.16	CALM1 (includes others)	calmodulin 1	Cytoplasm	other		-3,032	7.06×10^-07
ENSG00000198668.10	CALM1 (includes others)	calmodulin 1	Cytoplasm	other		-1,33	1.07×10^-02
ENSG00000179218.13	CALR	calreticulin	Cytoplasm	transcription regulator		-2,379	2.06×10^-05
ENSG00000128595.16	CALU	calumenin	Cytoplasm	other		-1,844	1.55×10^-04
ENSG00000171302.16	CANT1	calcium activated nucleotidase 1	Extracellular Space	enzyme		-1,495	2.71×10^-02
ENSG00000127022.14	CANX	calnexin	Cytoplasm	other		-2,058	1.10×10^-04
ENSG00000131236.16	CAP1	cyclase associated actin cytoskeleton regulatory protein 1	Plasma Membrane	other		-3,706	1.11×10^-05
ENSG00000135387.19	CAPRIN1	cell cycle associated protein 1	Plasma Membrane	other		-1,605	4.90×10^-03
ENSG00000105483.16	CARD8	caspase recruitment domain family member 8	Nucleus	other		-1,165	2.36×10^-02
ENSG00000255420.1	CASC23	cancer susceptibility 23	Other	other		2,87	3.89×10^-03
ENSG00000164305.17	CASP3	caspase 3	Cytoplasm	peptidase	caspase 3 inhibitor	-2,086	1.72×10^-02
ENSG00000177469.12	CAVIN1	caveolae associated protein 1	Nucleus	transcription regulator		-2,497	1.24×10^-05
ENSG00000168497.4	CAVIN2	caveolae associated protein 2	Plasma Membrane	other		-1,475	1.57×10^-02
ENSG00000122565.18	CBX3	chromobox 3	Nucleus	transcription regulator		-1,345	6.75×10^-03
ENSG00000183741.11	CBX6	chromobox 6	Nucleus	other		-2,013	1.17×10^-02
ENSG00000060339.13	CCAR1	cell division cycle and apoptosis regulator 1	Nucleus	transcription regulator		-1,293	4.20×10^-02
ENSG00000167131.16	CCDC103	coiled-coil domain containing 103	Cytoplasm	other		4,462	1.37×10^-04
ENSG00000154874.14	CCDC144B	coiled-coil domain containing 144B (pseudogene)	Other	other		-3,149	8.59×10^-04
ENSG00000135205.14	CCDC146	coiled-coil domain containing 146	Cytoplasm	other		2,127	2.90×10^-02
ENSG00000142871.15	CCN1	cellular communication network factor 1	Extracellular Space	other		-1,57	1.17×10^-02
ENSG00000118523.5	CCN2	cellular communication network factor 2	Extracellular Space	growth factor	FG-3019	-2,361	4.04×10^-05
ENSG00000157456.7	CCNB2	cyclin B2	Cytoplasm	other		-2,25	1.20×10^-02
ENSG00000166226.12	CCT2	chaperonin containing TCP1 subunit 2	Cytoplasm	kinase		-2,338	1.43×10^-03
ENSG00000163468.14	CCT3	chaperonin containing TCP1 subunit 3	Cytoplasm	other		-1,871	1.53×10^-04
ENSG00000115484.14	CCT4	chaperonin containing TCP1 subunit 4	Cytoplasm	other		-2,631	1.77×10^-05
ENSG00000150753.11	CCT5	chaperonin containing TCP1 subunit 5	Cytoplasm	other		-2,201	7.23×10^-05
ENSG00000146731.10	CCT6A	chaperonin containing TCP1 subunit 6A	Cytoplasm	other		-3,212	1.61×10^-05
ENSG00000135624.15	CCT7	chaperonin containing TCP1 subunit 7	Cytoplasm	other		-3,211	1.06×10^-03
ENSG00000156535.13	CD109	CD109 molecule	Plasma Membrane	other		-1,557	5.12×10^-03
ENSG00000085063.14	CD59	CD59 molecule (CD59 blood group)	Plasma Membrane	other		-2,104	4.57×10^-05
ENSG00000135404.11	CD63	CD63 molecule	Plasma Membrane	other		-3,968	1.32×10^-04
ENSG00000129226.13	CD68	CD68 molecule	Plasma Membrane	other		1,952	5.65×10^-03
ENSG00000010278.11	CD9	CD9 molecule	Plasma Membrane	other		-2,878	4.06×10^-04
ENSG00000125810.9	CD93	CD93 molecule	Plasma Membrane	other		-3,014	2.30×10^-08
ENSG00000117399.13	CDC20	cell division cycle 20	Nucleus	other		-2,053	3.47×10^-02
ENSG00000164287.12	CDC20B	cell division cycle 20B	Other	other		1,605	1.48×10^-02
ENSG00000070831.15	CDC42	cell division cycle 42	Cytoplasm	enzyme		-1,824	1.26×10^-02
ENSG00000096401.7	CDC5L	cell division cycle 5 like	Nucleus	transcription regulator		-1,523	4.33×10^-02
ENSG00000184661.13	CDCA2	cell division cycle associated 2	Nucleus	other		-1,905	1.40×10^-03
ENSG00000179776.17	CDH5	cadherin 5	Plasma Membrane	other		-3,513	1.69×10^-09
ENSG00000059758.7	CDK17	cyclin dependent kinase 17	Cytoplasm	kinase	SM1-71	-1,769	1.15×10^-02

ENSG00000153046.17	CDYL	chromodomain Y like	Nucleus	transcription regulator		-1,527	4.31×10^-02
ENSG00000196166.3	CEACAM7	CEA cell adhesion molecule 7	Plasma Membrane	other		1,793	4.31×10^-02
ENSG00000245848.2	CEBPA	CCAAT enhancer binding protein alpha	Nucleus	transcription regulator		2,543	4.48×10^-04
ENSG00000172216.5	CEBPB	CCAAT enhancer binding protein beta	Nucleus	transcription regulator		4,113	4.02×10^-04
ENSG00000221869.4	CEBPD	CCAAT enhancer binding protein delta	Nucleus	transcription regulator		4,191	1.20×10^-02
ENSG00000125817.7	CENPB	centromere protein B	Nucleus	other		-1,681	7.96×10^-03
ENSG00000138778.11	CENPE	centromere protein E	Nucleus	other	GSK923295	-2,312	6.61×10^-05
ENSG00000117724.12	CENPF	centromere protein F	Nucleus	other		-2,452	3.39×10^-05
ENSG00000100422.13	CERK	ceramide kinase	Plasma Membrane	kinase		-2,294	8.07×10^-03
ENSG00000188452.13	CERKL	ceramide kinase like	Nucleus	other		1,568	3.88×10^-02
ENSG00000197748.12	CFAP43	cilia and flagella associated protein 43	Cytoplasm	other		1,715	2.07×10^-02
ENSG00000206530.8	CFAP44	cilia and flagella associated protein 44	Extracellular Space	peptidase		2,532	1.99×10^-11
ENSG00000172757.12	CFL1	cofilin 1	Nucleus	other		-2,659	1.23×10^-05
ENSG00000167670.15	CHAF1A	chromatin assembly factor 1 subunit A	Nucleus	other		-2,49	7.67×10^-03
ENSG00000272888.5	CHASERR	CHD2 adjacent suppressive regulatory RNA	Other	other		1,177	4.76×10^-02
ENSG00000106153.12	CHCHD2	coiled-coil-helix-coiled-coil-helix domain containing 2	Cytoplasm	other		-3,814	2.09×10^-03
ENSG00000111642.14	CHD4	chromodomain helicase DNA binding protein 4	Nucleus	enzyme		-2,42	9.76×10^-03
ENSG00000100888.12	CHD8	chromodomain helicase DNA binding protein 8	Nucleus	enzyme		-1,59	4.19×10^-03
ENSG00000101421.3	CHMP4B	charged multivesicular body protein 4B	Cytoplasm	other		-2,001	2.55×10^-03
ENSG00000122966.13	CIT	citron rho-interacting serine/threonine kinase	Cytoplasm	kinase		-2,983	3.42×10^-06
ENSG00000175216.14	CKAP5	cytoskeleton associated protein 5	Nucleus	transcription regulator		-2,364	9.87×10^-06
ENSG00000166165.12	CKB	creatine kinase B	Cytoplasm	kinase		1,783	9.64×10^-04
ENSG00000013297.10	CLDN11	claudin 11	Plasma Membrane	other		-1,825	4.00×10^-02
ENSG00000176435.6	CLEC14A	C-type lectin domain containing 14A	Cytoplasm	other		-3,668	1.28×10^-04
ENSG00000213719.8	CLIC1	chloride intracellular channel 1	Nucleus	ion channel		-2,55	1.96×10^-04
ENSG00000169504.14	CLIC4	chloride intracellular channel 4	Plasma Membrane	ion channel		-1,945	9.99×10^-04
ENSG00000074201.8	CLNS1A	chloride nucleotide-sensitive channel 1A	Plasma Membrane	ion channel		-1,506	1.93×10^-02
ENSG00000092853.13	CLSPN	claspin	Nucleus	other		-1,808	1.57×10^-03
ENSG00000171603.16	CLSTN1	calsyntenin 1	Plasma Membrane	other		-2,345	1.31×10^-03
ENSG00000175416.12	CLTB	clathrin light chain B	Plasma Membrane	other		-1,501	3.58×10^-02
ENSG00000120885.19	CLU	clusterin	Cytoplasm	other	AB-16B5, clusterin antisense oligonucleotide	-1,517	2.01×10^-02
ENSG00000168405.14	CMAHP	cytidine monophospho-N-acetylneuraminic acid hydroxylase, pseudogene	Cytoplasm	other		2,13	3.80×10^-02
ENSG00000184220.10	CMSS1	cms1 ribosomal small subunit homolog	Other	other		-2,258	1.04×10^-02
ENSG00000169714.16	CNBP	CCHC-type zinc finger nucleic acid binding protein	Nucleus	transcription regulator		-1,431	4.00×10^-03
ENSG00000133313.14	CNDP2	carnosine dipeptidase 2	Cytoplasm	peptidase		-1,32	1.68×10^-02
ENSG00000205423.11	CNEP1R1	CTD nuclear envelope phosphatase 1 regulatory subunit 1	Nucleus	other		1,67	4.60×10^-02
ENSG00000064666.14	CNN2	calponin 2	Cytoplasm	other		-2,065	7.03×10^-03
ENSG00000117519.15	CNN3	calponin 3	Cytoplasm	other		-2,43	5.85×10^-04
ENSG00000125107.16	CNOT1	CCR4-NOT transcription complex subunit 1	Cytoplasm	other		-1,195	4.60×10^-02
ENSG00000182871.14	COL18A1	collagen type XVIII alpha 1 chain	Extracellular Space	other	collagenase	-2,555	2.48×10^-02
ENSG00000187498.14	COL4A1	collagen type IV alpha 1 chain	Extracellular Space	other	collagenase	-3,191	2.52×10^-07
ENSG00000134871.17	COL4A2	collagen type IV alpha 2 chain	Extracellular Space	other	collagenase	-3,622	3.32×10^-09
ENSG00000163359.15	COL6A3	collagen type VI alpha 3 chain	Extracellular Space	other	collagenase	1,104	5.96×10^-03
ENSG00000184432.9	COPB2	COPI coat complex subunit beta 2	Cytoplasm	transporter		-1,497	1.19×10^-02
ENSG00000138663.8	COPS4	COP9 signalosome subunit 4	Cytoplasm	peptidase		-1,682	4.21×10^-02
ENSG00000168090.9	COPS6	COP9 signalosome subunit 6	Nucleus	other		-1,747	1.08×10^-03
ENSG00000110880.10	CORO1C	coronin 1C	Cytoplasm	other		-1,732	7.59×10^-04
ENSG00000103187.7	COTL1	coactosin like F-actin binding protein 1	Cytoplasm	other		-1,616	3.49×10^-03
ENSG00000131143.8	COX4I1	cytochrome c oxidase subunit 4I1	Cytoplasm	enzyme		-1,652	1.25×10^-03
ENSG00000178741.11	COX5A	cytochrome c oxidase subunit 5A	Cytoplasm	enzyme		-2,206	1.20×10^-02
ENSG00000160471.12	COX6B2	cytochrome c oxidase subunit 6B2	Cytoplasm	enzyme		2,495	7.27×10^-03
ENSG00000164919.10	COX6C	cytochrome c oxidase subunit 6C	Cytoplasm	enzyme		-2,672	9.84×10^-06
ENSG00000127184.10	COX7C	cytochrome c oxidase subunit 7C	Cytoplasm	enzyme		-2,195	2.61×10^-06
ENSG00000110090.12	CPT1A	carnitine palmitoyltransferase 1A	Cytoplasm	enzyme	perhexiline	-1,53	4.94×10^-02
ENSG00000150938.9	CRIM1	cysteine rich transmembrane BMP regulator 1	Extracellular Space	kinase		-2,439	9.87×10^-06
ENSG00000009307.15	CSDE1	cold shock domain containing E1	Cytoplasm	enzyme		-1,93	2.34×10^-03
ENSG00000124207.16	CSE1L	chromosome segregation 1 like	Nucleus	transporter		-1,522	1.81×10^-02
ENSG00000113712.16	CSNK1A1	casein kinase 1 alpha 1	Cytoplasm	kinase	SM1-71	-1,654	1.32×10^-03
ENSG00000204435.13	CSNK2B	casein kinase 2 beta	Cytoplasm	kinase		-2,93	5.99×10^-03
ENSG00000160213.5	CSTB	cystatin B	Cytoplasm	peptidase		-1,534	4.08×10^-02
ENSG00000260708.1	CTA_29F111		Other	other		2,535	8.01×10^-05
ENSG00000102974.14	CTCF	CCCTC-binding factor	Nucleus	transcription regulator		-1,868	8.13×10^-03
ENSG00000259433.2	CTD_2651B204		Other	other		1,544	3.27×10^-02
ENSG00000269825.1	CTD_3099C69		Other	other		-2,069	2.71×10^-02
ENSG00000175215.9	CTDSP2	CTD small phosphatase 2	Nucleus	phosphatase		-1,584	1.21×10^-02
ENSG00000044115.20	CTNNA1	catenin alpha 1	Plasma Membrane	other		-1,435	9.45×10^-03
ENSG00000119326.14	CTNNAL1	catenin alpha like 1	Plasma Membrane	other		-1,778	4.67×10^-02
ENSG00000198561.12	CTNND1	catenin delta 1	Nucleus	other		-1,24	1.33×10^-02
ENSG00000164733.20	CTSB	cathepsin B	Cytoplasm	peptidase		-2,575	6.05×10^-06
ENSG00000101160.13	CTSZ	cathepsin Z	Cytoplasm	peptidase		-2,083	1.79×10^-02
ENSG00000055130.15	CUL1	cullin 1	Nucleus	enzyme		-1,962	2.11×10^-02
ENSG00000139842.14	CUL4A	cullin 4A	Nucleus	other		-1,342	2.71×10^-02
ENSG00000081041.8	CXCL2	C-X-C motif chemokine ligand 2	Extracellular Space	cytokine		2,311	5.41×10^-06
ENSG00000163734.4	CXCL3	C-X-C motif chemokine ligand 3	Extracellular Space	cytokine		2,564	6.76×10^-07
ENSG00000169429.10	CXCL8	C-X-C motif chemokine ligand 8	Extracellular Space	cytokine	BMS-986253	2,558	3.71×10^-09
ENSG00000167740.9	CYB5D2	cytochrome b5 domain containing 2	Extracellular Space	other		1,507	4.60×10^-02
ENSG00000100243.20	CYB5R3	cytochrome b5 reductase 3	Cytoplasm	enzyme		-2,581	2.56×10^-04
ENSG00000273749.4	CYFIP1	cytoplasmic FMR1 interacting protein 1	Cytoplasm	translation regulator		-1,108	4.27×10^-02
ENSG00000129562.10	DAD1	defender against cell death 1	Cytoplasm	other		-3,8	1.85×10^-03
ENSG00000155368.16	DBI	diazepam binding inhibitor, acyl-CoA binding protein	Cytoplasm	other		-2,881	4.56×10^-06
ENSG00000113758.13	DBN1	drebrin 1	Cytoplasm	other		-3,348	3.38×10^-11
ENSG00000166341.7	DCHS1	dachsous cadherin-related 1	Plasma Membrane	other		-1,726	2.11×10^-02
ENSG00000129187.14	DCTD	dCMP deaminase	Cytoplasm	enzyme	tetrahydrouridine	-2,661	5.37×10^-03
ENSG00000204843.12	DCTN1	dynactin subunit 1	Cytoplasm	other		-1,403	5.12×10^-03
ENSG00000167986.13	DDB1	damage specific DNA binding protein 1	Nucleus	other		-1,02	3.19×10^-02
ENSG00000175197.10	DDIT3	DNA damage inducible transcript 3	Nucleus	transcription regulator		1,918	6.08×10^-05
ENSG00000244038.9	DDOST	dolichyl-diphosphooligosaccharide--protein glycosyltransferase non-catalytic subunit	Cytoplasm	enzyme		-2,251	3.27×10^-02
ENSG00000079785.14	DDX1	DEAD-box helicase 1	Nucleus	enzyme		-2,127	2.63×10^-03
ENSG00000100201.18	DDX17	DEAD-box helicase 17	Nucleus	enzyme		-2,031	8.25×10^-04
ENSG00000215301.9	DDX3X	DEAD-box helicase 3 X-linked	Cytoplasm	enzyme		-1,299	3.63×10^-02
ENSG00000108654.11	DDX5	DEAD-box helicase 5	Nucleus	enzyme		-1,44	9.76×10^-03
ENSG00000165507.8	DEPP1	DEPP1 autophagy regulator	Cytoplasm	other		-3,209	7.92×10^-04
ENSG00000077044.9	DGKD	diacylglycerol kinase delta	Cytoplasm	kinase		1,127	2.93×10^-02
ENSG00000133943.20	DGLUCY	D-glutamate cyclase	Cytoplasm	enzyme		1,325	1.41×10^-02
ENSG00000116133.11	DHCR24	24-dehydrocholesterol reductase	Cytoplasm	enzyme		-2,06	1.62×10^-02
ENSG00000109606.12	DHX15	DEAH-box helicase 15	Nucleus	enzyme		-1,263	4.56×10^-02
ENSG00000204560.9	DHX16	DEAH-box helicase 16	Nucleus	enzyme		-1,861	4.80×10^-02
ENSG00000140829.11	DHX38	DEAH-box helicase 38	Nucleus	enzyme		-1,495	3.44×10^-02
ENSG00000135829.16	DHX9	DExH-box helicase 9	Nucleus	enzyme		-1,395	1.20×10^-02
ENSG00000131504.15	DIAPH1	diaphanous related formin 1	Plasma Membrane	other		-1,584	1.40×10^-03
ENSG00000180525.11	DIP2C-AS1	DIP2C antisense RNA 1	Other	other		1,111	1.83×10^-02
ENSG00000147113.16	DIPK2B	divergent protein kinase domain 2B	Other	other		-1,512	1.39×10^-02
ENSG00000130826.15	DKC1	dyskerin pseudouridine synthase 1	Nucleus	enzyme		-1,476	1.57×10^-02
ENSG00000126787.12	DLGAP5	DLG associated protein 5	Nucleus	other		-1,97	4.07×10^-02
ENSG00000183914.14	DNAH2	dynein axonemal heavy chain 2	Cytoplasm	other		1,06	2.05×10^-02
ENSG00000086061.15	DNAJA1	DnaJ heat shock protein family (Hsp40) member A1	Nucleus	other		-2,499	9.87×10^-06
ENSG00000132002.7	DNAJB1	DnaJ heat shock protein family (Hsp40) member B1	Nucleus	transcription regulator		1,936	1.40×10^-05
ENSG00000102580.14	DNAJC3	DnaJ heat shock protein family (Hsp40) member C3	Cytoplasm	other		-2,028	2.63×10^-02
ENSG00000247400.3	DNAJC3-DT	DNAJC3 divergent transcript	Other	other		1,674	2.94×10^-02
ENSG00000101152.10	DNAJC5	DnaJ heat shock protein family (Hsp40) member C5	Plasma Membrane	other		-1,419	4.85×10^-02
ENSG00000126698.10	DNAJC8	DnaJ heat shock protein family (Hsp40) member C8	Nucleus	other		-1,898	4.15×10^-02
ENSG00000107554.15	DNMBP	dynamin binding protein	Cytoplasm	other		-1,383	6.38×10^-03
ENSG00000227695.5	DNMBP-AS1	DNMBP antisense RNA 1	Other	other		1,772	3.90×10^-02
ENSG00000150760.12	DOCK1	dedicator of cytokinesis 1	Cytoplasm	other		-1,61	1.29×10^-02
ENSG00000128512.19	DOCK4	dedicator of cytokinesis 4	Plasma Membrane	other		-1,87	7.78×10^-05
ENSG00000088387.17	DOCK9	dedicator of cytokinesis 9	Cytoplasm	other		-1,432	8.11×10^-03
ENSG00000092964.16	DPYSL2	dihydropyrimidinase like 2	Cytoplasm	enzyme		-1,917	2.55×10^-04
ENSG00000113657.12	DPYSL3	dihydropyrimidinase like 3	Cytoplasm	enzyme		-1,431	3.56×10^-03
ENSG00000151914.17	DST	dystonin	Plasma Membrane	other		-1,59	2.15×10^-03
ENSG00000125868.15	DSTN	destrin, actin depolymerizing factor	Cytoplasm	other		-1,878	3.24×10^-04
ENSG00000125821.11	DTD1	D-aminoacyl-tRNA deacylase 1	Cytoplasm	enzyme		-1,536	4.33×10^-02
ENSG00000120129.5	DUSP1	dual specificity phosphatase 1	Nucleus	phosphatase		1,374	1.92×10^-02
ENSG00000244306.9	DUXAP10	double homeobox A pseudogene 10	Other	other		1,163	1.74×10^-02
ENSG00000197102.10	DYNC1H1	dynein cytoplasmic 1 heavy chain 1	Cytoplasm	peptidase		-1,355	4.91×10^-02
ENSG00000077380.15	DYNC1I2	dynein cytoplasmic 1 intermediate chain 2	Cytoplasm	other		-1,489	2.00×10^-02
ENSG00000126870.15	DYNC2I1	dynein 2 intermediate chain 1	Extracellular Space	other		-2,317	5.91×10^-05
ENSG00000135636.13	DYSF	dysferlin	Plasma Membrane	other		-5,089	2.50×10^-04
ENSG00000205250.8	E2F4	E2F transcription factor 4	Nucleus	transcription regulator		-1,839	1.45×10^-02
ENSG00000117298.14	ECE1	endothelin converting enzyme 1	Plasma Membrane	peptidase		-2,784	2.81×10^-06
ENSG00000104823.8	ECH1	enoyl-CoA hydratase 1	Cytoplasm	enzyme		-5,193	3.77×10^-04
ENSG00000198721.12	ECI2	enoyl-CoA delta isomerase 2	Cytoplasm	enzyme		-2,522	4.36×10^-03
ENSG00000136813.14	ECPAS	Ecm29 proteasome adaptor and scaffold	Cytoplasm	other		-1,316	1.86×10^-02
ENSG00000249751.3	ECSCR	endothelial cell surface expressed chemotaxis and apoptosis regulator	Other	other		-5,685	3.13×10^-06
ENSG00000114346.13	ECT2	epithelial cell transforming 2	Cytoplasm	other		-1,939	9.83×10^-03
ENSG00000102189.16	EEA1	early endosome antigen 1	Cytoplasm	other		-1,245	1.33×10^-02
ENSG00000156508.17	EEF1A1	eukaryotic translation elongation factor 1 alpha 1	Cytoplasm	translation regulator		-2,46	5.62×10^-05
ENSG00000196205.8	EEF1A1P5	eukaryotic translation elongation factor 1 alpha 1 pseudogene 5	Extracellular Space	other		-1,981	2.56×10^-03
ENSG00000114942.13	EEF1B2	eukaryotic translation elongation factor 1 beta 2	Cytoplasm	translation regulator		-1,804	1.62×10^-03
ENSG00000254772.9	EEF1G	eukaryotic translation elongation factor 1 gamma	Cytoplasm	translation regulator		-2,318	4.89×10^-05
ENSG00000115380.18	EFEMP1	EGF containing fibulin extracellular matrix protein 1	Extracellular Space	enzyme		-3,905	1.22×10^-12
ENSG00000172889.15	EGFL7	EGF like domain multiple 7	Extracellular Space	other	parsatuzumab	-5,172	3.04×10^-04
ENSG00000129521.13	EGLN3	egl-9 family hypoxia inducible factor 3	Cytoplasm	enzyme	molidustat	1,143	1.86×10^-02
ENSG00000120738.7	EGR1	early growth response 1	Nucleus	transcription regulator		2,601	2.83×10^-13
ENSG00000024422.11	EHD2	EH domain containing 2	Nucleus	other		-2,807	3.70×10^-04
ENSG00000103966.9	EHD4	EH domain containing 4	Plasma Membrane	enzyme		-2,046	2.71×10^-04
ENSG00000125977.6	EIF2S2	eukaryotic translation initiation factor 2 subunit beta	Cytoplasm	translation regulator		-1,421	6.90×10^-03
ENSG00000130741.10	EIF2S3	eukaryotic translation initiation factor 2 subunit gamma	Cytoplasm	translation regulator		-1,879	1.86×10^-03
ENSG00000107581.12	EIF3A	eukaryotic translation initiation factor 3 subunit A	Cytoplasm	translation regulator		-1,576	1.62×10^-02
ENSG00000184110.14	EIF3C	eukaryotic translation initiation factor 3 subunit C	Cytoplasm	translation regulator		-1,521	9.82×10^-03
ENSG00000100353.17	EIF3D	eukaryotic translation initiation factor 3 subunit D	Cytoplasm	translation regulator		-2,171	2.97×10^-04
ENSG00000104408.9	EIF3E	eukaryotic translation initiation factor 3 subunit E	Cytoplasm	translation regulator		-1,852	3.31×10^-03
ENSG00000147677.10	EIF3H	eukaryotic translation initiation factor 3 subunit H	Cytoplasm	enzyme		-1,242	2.31×10^-02
ENSG00000100129.17	EIF3L	eukaryotic translation initiation factor 3 subunit L	Cytoplasm	translation regulator		-4,373	2.28×10^-04
ENSG00000161960.14	EIF4A1	eukaryotic translation initiation factor 4A1	Cytoplasm	translation regulator		-1,368	3.91×10^-02
ENSG00000063046.17	EIF4B	eukaryotic translation initiation factor 4B	Cytoplasm	translation regulator		-1,289	2.05×10^-02
ENSG00000114867.19	EIF4G1	eukaryotic translation initiation factor 4 gamma 1	Cytoplasm	translation regulator		-2,288	2.48×10^-05
ENSG00000110321.15	EIF4G2	eukaryotic translation initiation factor 4 gamma 2	Cytoplasm	translation regulator		-1,506	7.03×10^-03
ENSG00000075151.19	EIF4G3	eukaryotic translation initiation factor 4 gamma 3	Cytoplasm	translation regulator		-2,101	8.75×10^-04
ENSG00000106682.14	EIF4H	eukaryotic translation initiation factor 4H	Cytoplasm	translation regulator		-1,519	9.86×10^-03
ENSG00000132507.17	EIF5A	eukaryotic translation initiation factor 5A	Cytoplasm	translation regulator	SNS01-T nanoparticles	-1,459	2.35×10^-02
ENSG00000158417.10	EIF5B	eukaryotic translation initiation factor 5B	Cytoplasm	translation regulator		-1,665	1.12×10^-03

ENSG00000120690.13	ELF1	E74 like ETS transcription factor 1	Nucleus	transcription regulator		-1,805	3.32×10^-02
ENSG00000111145.7	ELK3	ETS transcription factor ELK3	Nucleus	transcription regulator		-2,337	1.34×10^-04
ENSG00000134531.9	EMP1	epithelial membrane protein 1	Plasma Membrane	other		-1,084	3.95×10^-02
ENSG00000106991.13	ENG	endoglin	Plasma Membrane	transmembrane receptor	carotuximab	-3,921	7.69×10^-06
ENSG00000074800.13	ENO1	enolase 1	Cytoplasm	enzyme		-2,208	1.28×10^-04
ENSG00000145293.14	ENOPH1	enolase-phosphatase 1	Cytoplasm	enzyme		-2,55	2.05×10^-02
ENSG00000079819.16	EPB41L2	erythrocyte membrane protein band 4.1 like 2	Plasma Membrane	other		-1,08	1.86×10^-02
ENSG00000229153.5	EPHA1-AS1	EPHA1 antisense RNA 1	Other	other		1,103	2.86×10^-02
ENSG00000133216.16	EPHB2	EPH receptor B2	Plasma Membrane	kinase	ALW-II-49-7, ALW-II-38-3, SM1-71	-1,464	5.29×10^-03
ENSG00000196411.9	EPHB4	EPH receptor B4	Plasma Membrane	kinase	tesevatinib, NVP-BHG712, JI 101, AZ12672	-1,587	4.51×10^-02
ENSG00000136628.17	EPRS1	glutamyl-prolyl-tRNA synthetase 1	Cytoplasm	enzyme		-1,611	1.14×10^-02
ENSG00000105722.9	ERF	ETS2 repressor factor	Nucleus	transcription regulator		-3,715	1.07×10^-02
ENSG00000157554.18	ERG	ETS transcription factor ERG	Nucleus	transcription regulator		-1,265	4.50×10^-02
ENSG00000100632.10	ERH	ERH mRNA splicing and mitosis factor	Nucleus	other		-1,984	1.03×10^-02
ENSG00000165837.11	ERICH6B	glutamate rich 6B	Other	other		1,572	3.05×10^-03
ENSG00000149564.11	ESAM	endothelial cell adhesion molecule	Plasma Membrane	other		-2,407	3.69×10^-02
ENSG00000089048.14	ESF1	ESF1 nucleolar pre-rRNA processing protein homolog	Nucleus	other		-2,224	3.65×10^-05
ENSG00000164283.12	ESM1	endothelial cell specific molecule 1	Extracellular Space	growth factor		-2,681	7.57×10^-04
ENSG00000139641.12	ESYT1	extended synaptotagmin 1	Cytoplasm	other		-2,204	2.20×10^-02
ENSG00000185862.6	EVI2B	ecotropic viral integration site 2B	Plasma Membrane	other		1,794	4.17×10^-03
ENSG00000182944.17	EWSR1	EWS RNA binding protein 1	Nucleus	other		-1,255	2.06×10^-02
ENSG00000181104.6	F2R	coagulation factor II thrombin receptor	Plasma Membrane	G-protein coupled receptor	chrysalin, vorapaxar, PAR1 inhibitor, argatro	-1,373	1.35×10^-02
ENSG00000164687.10	FABP5	fatty acid binding protein 5	Cytoplasm	transporter		-1,148	4.33×10^-02
ENSG00000134824.13	FADS2	fatty acid desaturase 2	Plasma Membrane	enzyme		-1,75	4.74×10^-03
ENSG00000065809.13	FAM107B	family with sequence similarity 107 member B	Other	other		-1,226	4.55×10^-02
ENSG00000138640.14	FAM13A	family with sequence similarity 13 member A	Cytoplasm	other		1,173	1.76×10^-03
ENSG00000161682.14	FAM171A2	family with sequence similarity 171 member A2	Other	other		3,971	6.16×10^-04
ENSG00000005238.19	FAM214B	family with sequence similarity 214 member B	Nucleus	other		1,318	9.34×10^-03
ENSG00000139438.5	FAM222A	family with sequence similarity 222 member A	Other	other		1,469	4.78×10^-02
ENSG00000185112.5	FAM43A	family with sequence similarity 43 member A	Other	other		-2,191	7.36×10^-03
ENSG00000196550.10	FAM72A	family with sequence similarity 72 member A	Cytoplasm	other		1,25	3.49×10^-03
ENSG00000188610.12	FAM72B	family with sequence similarity 72 member B	Cytoplasm	other		2,126	2.64×10^-05
ENSG00000263513.5	FAM72C/FAM72D	family with sequence similarity 72 member D	Nucleus	other		1,339	8.23×10^-03
ENSG00000125998.7	FAM83C	family with sequence similarity 83 member C	Other	other		1,32	4.75×10^-02
ENSG00000149806.10	FAU	FAU ubiquitin like and ribosomal protein S30 fusion	Cytoplasm	other		-2,039	2.60×10^-04
ENSG00000105202.7	FBL	fibrillarin	Nucleus	enzyme		-1,706	1.31×10^-02
ENSG00000162458.12	FBLIM1	filamin binding LIM protein 1	Plasma Membrane	other		-2,237	4.75×10^-02
ENSG00000138829.10	FBN2	fibrillin 2	Extracellular Space	other		-2,341	4.08×10^-05
ENSG00000118564.14	FBXL5	F-box and leucine rich repeat protein 5	Cytoplasm	enzyme		-2,889	4.53×10^-03
ENSG00000138081.19	FBXO11	F-box protein 11	Cytoplasm	enzyme		-1,98	4.66×10^-03
ENSG00000178974.9	FBXO34	F-box protein 34	Other	other		-1,259	2.79×10^-02
ENSG00000079459.12	FDFT1	farnesyl-diphosphate farnesyltransferase 1	Cytoplasm	enzyme	lapaquistat, zoledronic acid	-1,586	1.33×10^-02
ENSG00000160752.14	FDPS	farnesyl diphosphate synthase	Cytoplasm	enzyme	risedronic acid, zoledronic acid, minodronate	-1,227	1.93×10^-02
ENSG00000161513.11	FDXR	ferredoxin reductase	Cytoplasm	enzyme		-1,335	1.58×10^-02
ENSG00000249715.9	FER1L5	fer-1 like family member 5	Other	other		2,601	2.87×10^-08
ENSG00000176971.3	FIBIN	fin bud initiation factor homolog	Cytoplasm	other		7,792	6.56×10^-44
ENSG00000088832.14	FKBP1A	FKBP prolyl isomerase 1A	Cytoplasm	enzyme	methotrexate/sirolimus/tacrolimus, prednisor	-2,385	1.05×10^-04
ENSG00000100442.10	FKBP3	FKBP prolyl isomerase 3	Nucleus	enzyme		-2,246	3.19×10^-02
ENSG00000004478.7	FKBP4	FKBP prolyl isomerase 4	Nucleus	enzyme		-1,645	1.40×10^-02
ENSG00000105701.15	FKBP8	FKBP prolyl isomerase 8	Cytoplasm	other		-1,614	3.49×10^-02
ENSG00000143631.10	FLG	filaggrin	Cytoplasm	other		-1,24	1.31×10^-02
ENSG00000151702.16	FLI1	Fli-1 proto-oncogene, ETS transcription factor	Nucleus	transcription regulator		-1,147	3.62×10^-02
ENSG00000177731.15	FLII	FLII actin remodeling protein	Nucleus	other		-1,787	7.30×10^-03
ENSG00000136068.14	FLNB	filamin B	Cytoplasm	other		-2,834	2.61×10^-06
ENSG00000128591.15	FLNC	filamin C	Cytoplasm	other		-4,113	5.58×10^-04
ENSG00000131781.12	FMO5	flavin containing dimethylaniline monoxygenase 5	Cytoplasm	enzyme		1,183	3.48×10^-02
ENSG00000115414.18	FN1	fibronectin 1	Extracellular Space	enzyme	ocriplasmin, bifikafusp alfa, L19TNFalpha, L	-2,371	3.34×10^-05
ENSG00000170345.9	FOS	Fos proto-oncogene, AP-1 transcription factor subunit	Nucleus	transcription regulator		2,807	1.69×10^-09
ENSG00000054598.6	FOXC1	forkhead box C1	Nucleus	transcription regulator		1,73	3.50×10^-06
ENSG00000237424.1	FOXD2-AS1	FOXD2 adjacent opposite strand RNA 1	Other	other		1,783	4.47×10^-02
ENSG00000186766.7	FOXI2	forkhead box I2	Nucleus	transcription regulator		1,402	1.45×10^-02
ENSG00000065970.8	FOXJ2	forkhead box J2	Nucleus	transcription regulator		5,64	2.08×10^-23
ENSG00000111206.12	FOXM1	forkhead box M1	Nucleus	transcription regulator		-2,061	4.27×10^-03
ENSG00000205097.6	FRG2/FRG2B	FSHD region gene 2 family member B	Other	other		1,801	2.10×10^-02
ENSG00000134363.11	FST	follistatin	Extracellular Space	other		1,739	1.45×10^-03
ENSG00000163430.9	FSTL1	follistatin like 1	Extracellular Space	other		-1,152	4.35×10^-02
ENSG00000167996.15	FTH1	ferritin heavy chain 1	Cytoplasm	enzyme		-1,927	3.08×10^-03
ENSG00000114416.17	FXR1	FMR1 autosomal homolog 1	Cytoplasm	other		-1,354	3.79×10^-02
ENSG00000163820.14	FYCO1	FYVE and coiled-coil domain autophagy adaptor 1	Cytoplasm	other		-1,145	3.18×10^-02
ENSG00000138757.14	G3BP2	G3BP stress granule assembly factor 2	Cytoplasm	enzyme		-2,935	4.94×10^-06
ENSG00000136928.4	GABBR2	gamma-aminobutyric acid type B receptor subunit 2	Plasma Membrane	G-protein coupled receptor	baclofen, arbaclofen, amitriptyline/baclofen/k	-1,528	2.72×10^-02
ENSG00000099860.8	GADD45B	growth arrest and DNA damage inducible beta	Cytoplasm	other		1,317	3.31×10^-02
ENSG00000141429.13	GALNT1	polypeptide N-acetylgalactosaminyltransferase 1	Cytoplasm	enzyme		-2,615	3.24×10^-04
ENSG00000143641.9	GALNT2	polypeptide N-acetylgalactosaminyltransferase 2	Cytoplasm	enzyme		-1,126	4.07×10^-02
ENSG00000257594.3	GALNT4	polypeptide N-acetylgalactosaminyltransferase 4	Cytoplasm	enzyme		1,263	4.20×10^-02
ENSG00000089597.16	GANAB	glucosidase II alpha subunit	Cytoplasm	enzyme	miglitol	-2,246	2.55×10^-03
ENSG00000111640.14	GAPDH	glyceraldehyde-3-phosphate dehydrogenase	Cytoplasm	enzyme		-1,609	1.82×10^-02
ENSG00000106105.13	GARS1	glycyl-tRNA synthetase 1	Cytoplasm	enzyme		-1,508	9.26×10^-03
ENSG00000114480.12	GBE1	1,4-alpha-glucan branching enzyme 1	Cytoplasm	enzyme		-2,109	5.00×10^-03
ENSG00000131979.18	GCH1	GTP cyclohydrolase 1	Cytoplasm	enzyme		2,918	1.87×10^-12
ENSG00000135414.9	GDF11	growth differentiation factor 11	Extracellular Space	growth factor	luspatercept	-1,089	2.92×10^-02
ENSG00000057608.16	GDI2	GDP dissociation inhibitor 2	Cytoplasm	other		-1,712	3.94×10^-03
ENSG00000131459.12	GFPT2	glutamine-fructose-6-phosphate transaminase 2	Cytoplasm	enzyme		2,791	2.93×10^-09
ENSG00000165678.19	GHITM	growth hormone inducible transmembrane protein	Cytoplasm	other		-1,846	1.47×10^-02
ENSG00000204120.14	GIGYF2	GRB10 interacting GYF protein 2	Cytoplasm	other		-1,293	2.35×10^-02
ENSG00000133574.9	GIMAP4	GTPase, IMAP family member 4	Nucleus	other		-2,177	2.28×10^-05
ENSG00000171115.3	GIMAP8	GTPase, IMAP family member 8	Other	other		-1,374	1.68×10^-02
ENSG00000152661.7	GJA1	gap junction protein alpha 1	Plasma Membrane	transporter		-1,423	4.35×10^-02
ENSG00000090863.11	GLG1	golgi glycoprotein 1	Cytoplasm	other		-1,166	3.62×10^-02
ENSG00000114353.16	GNAI2	G protein subunit alpha i2	Plasma Membrane	enzyme		-2,204	1.65×10^-04
ENSG00000078369.17	GNB1	G protein subunit beta 1	Plasma Membrane	enzyme		-1,664	7.73×10^-04
ENSG00000127920.5	GNG11	G protein subunit gamma 11	Plasma Membrane	enzyme		-2,526	2.41×10^-05
ENSG00000134697.12	GNL2	G protein nucleolar 2	Nucleus	enzyme		-1,285	4.50×10^-02
ENSG00000111670.14	GNPTAB	N-acetylglucosamine-1-phosphate transferase subunits alpha and beta	Cytoplasm	enzyme		1,165	1.74×10^-02
ENSG00000136935.13	GOLGA1	golgin A1	Cytoplasm	other		-1,314	4.30×10^-02
ENSG00000144674.16	GOLGA4	golgin A4	Cytoplasm	other		-1,125	2.55×10^-02
ENSG00000277322.1	GOLGA6L6 (includes others)	golgin A6 family like 6	Other	other		1,022	2.35×10^-02
ENSG00000278662.4	GOLGA6L9 (includes others)	golgin A6 family like 9	Other	other		1,206	3.04×10^-03
ENSG00000115806.12	GORASP2	golgi reassembly stacking protein 2	Cytoplasm	other		-1,519	3.61×10^-03
ENSG00000125166.12	GOT2	glutamic-oxaloacetic transaminase 2	Cytoplasm	enzyme		-1,598	2.35×10^-02
ENSG00000160818.16	GPATCH4	G-patch domain containing 4	Other	other		-1,377	9.72×10^-03
ENSG00000167588.12	GPD1	glycerol-3-phosphate dehydrogenase 1	Cytoplasm	enzyme		1,774	6.89×10^-03
ENSG00000105220.14	GPI	glucose-6-phosphate isomerase	Extracellular Space	enzyme		-1,129	3.61×10^-02
ENSG00000166123.13	GPT2	glutamic--pyruvic transaminase 2	Cytoplasm	enzyme		1,254	4.90×10^-03
ENSG00000167468.16	GPX4	glutathione peroxidase 4	Cytoplasm	enzyme		-3,053	1.26×10^-07
ENSG00000169181.12	GSG1L	GSG1 like	Plasma Membrane	other		1,03	2.80×10^-02
ENSG00000104687.12	GSR	glutathione-disulfide reductase	Cytoplasm	enzyme	carmustine/prednisone, carmustine	-1,882	2.23×10^-02
ENSG00000197265.8	GTF2E2	general transcription factor IIE subunit 2	Nucleus	transcription regulator		-2,531	1.22×10^-02
ENSG00000226259.10	GTF2H2B	general transcription factor IIH subunit 2B (pseudogene)	Other	other		-3,319	4.60×10^-02
ENSG00000270196.1	GTF3C6P3	GTF3C6 pseudogene 3	Other	other		1,971	3.88×10^-02
ENSG00000178605.12	GTPBP6	GTP binding protein 6 (putative)	Other	other		1,099	1.62×10^-02
ENSG00000189060.5	H1-0	H1.0 linker histone	Nucleus	other		1,38	3.47×10^-02
ENSG00000184897.5	H1-10	H1.10 linker histone	Nucleus	other		2,884	2.72×10^-03
ENSG00000196787.3	H2AC11	H2A clustered histone 11	Nucleus	other		-4,182	3.18×10^-06
ENSG00000276903.1	H2AC16	H2A clustered histone 16	Nucleus	other		-1,76	3.46×10^-02
ENSG00000184260.5	H2AC20	H2A clustered histone 20	Nucleus	other		1,499	1.08×10^-02
ENSG00000188486.3	H2AX	H2A.X variant histone	Nucleus	transcription regulator		1,249	3.37×10^-02
ENSG00000261716.1	H2BC20P	H2B clustered histone 20, pseudogene	Other	other		1,123	1.82×10^-02
ENSG00000184678.9	H2BC21	H2B clustered histone 21	Nucleus	other		1,202	9.82×10^-03
ENSG00000196890.4	H2BU1	H2B.U histone 1	Nucleus	other		1,26	2.55×10^-02
ENSG00000183598.3	H3C13	H3 clustered histone 13	Nucleus	other		2,166	3.23×10^-05
ENSG00000273983.1	H3C8	H3 clustered histone 8	Nucleus	other		-2,965	1.37×10^-04
ENSG00000270882.2	H4C14	H4 clustered histone 14	Nucleus	other		3,127	2.35×10^-02
ENSG00000276180.1	H4C9	H4 clustered histone 9	Nucleus	other		-2,136	4.96×10^-02
ENSG00000084754.10	HADHA	hydroxyacyl-CoA dehydrogenase trifunctional multienzyme complex subunit alpha	Cytoplasm	enzyme		-1,844	5.42×10^-03
ENSG00000137814.9	HAUS2	HAUS augmin like complex subunit 2	Cytoplasm	other		-2,411	3.21×10^-02
ENSG00000249115.8	HAUS5	HAUS augmin like complex subunit 5	Cytoplasm	other		1,479	7.32×10^-03
ENSG00000188536.12	HBA1/HBA2	hemoglobin subunit alpha 2	Extracellular Space	transporter	iron dextran, mefloquine, donepezil/mefloqui	3,239	4.46×10^-07
ENSG00000068024.16	HDAC4	histone deacetylase 4	Nucleus	transcription regulator	OKI-179, tributyrin, belinostat, pyroxamide	1,218	1.22×10^-02
ENSG00000115677.16	HDLBP	high density lipoprotein binding protein	Nucleus	transporter		-1,674	2.48×10^-03
ENSG00000092148.12	HECTD1	HECT domain E3 ubiquitin protein ligase 1	Other	enzyme		-1,643	6.65×10^-04
ENSG00000276550.4	HERC2P2	hect domain and RLD 2 pseudogene 2	Other	other		-1,71	4.81×10^-02
ENSG00000186834.3	HEXIM1	HEXIM P-TEFb complex subunit 1	Nucleus	transcription regulator		1,721	5.03×10^-05
ENSG00000019991.15	HGF	hepatocyte growth factor	Extracellular Space	growth factor	rilotumumab, SCH 900105, bevacizumab/rilo	5,851	1.34×10^-46
ENSG00000164161.9	HHIP	hedgehog interacting protein	Plasma Membrane	other		-2,253	7.34×10^-06
ENSG00000100644.16	HIF1A	hypoxia inducible factor 1 subunit alpha	Nucleus	transcription regulator	PX 478, EZN 2968	-1,193	3.80×10^-02
ENSG00000149929.15	HIRIP3	HIRA interacting protein 3	Nucleus	other		-1,299	8.11×10^-03
ENSG00000123485.11	HJURP	Holliday junction recognition protein	Nucleus	other		-1,321	1.30×10^-02
ENSG00000156515.21	HK1	hexokinase 1	Cytoplasm	kinase		-1,546	9.95×10^-03
ENSG00000204525.14	HLA-C	major histocompatibility complex, class I, C	Plasma Membrane	other		-2,068	2.80×10^-02
ENSG00000204592.8	HLA-E	major histocompatibility complex, class I, E	Plasma Membrane	transmembrane receptor		-1,491	4.78×10^-02
ENSG00000181126.13	HLA-V	major histocompatibility complex, class I, V (pseudogene)	Other	other		2,152	2.69×10^-09
ENSG00000137309.19	HMGA1	high mobility group AT-hook 1	Nucleus	transcription regulator		-2,141	1.68×10^-03
ENSG00000189403.14	HMGB1	high mobility group box 1	Nucleus	transcription regulator		-1,807	1.82×10^-04
ENSG00000230547.1	HMGB1P11	high mobility group box 1 pseudogene 11	Other	other		1,887	8.57×10^-03
ENSG00000259781.1	HMGB1P6	high mobility group box 1 pseudogene 6	Other	other		-2,498	4.27×10^-07
ENSG00000135486.17	HNRNPA1	heterogeneous nuclear ribonucleoprotein A1	Nucleus	other		-2,489	1.23×10^-05
ENSG00000224578.4	HNRNPA1P48	heterogeneous nuclear ribonucleoprotein A1 pseudogene 48	Other	other		-1,289	4.52×10^-02
ENSG00000122566.20	HNRNPA2B1	heterogeneous nuclear ribonucleoprotein A2/B1	Nucleus	other		-1,838	1.45×10^-03
ENSG00000170144.18	HNRNPA3	heterogeneous nuclear ribonucleoprotein A3	Nucleus	other		-2,222	1.06×10^-04
ENSG00000226790.3	HNRNPA3P1	heterogeneous nuclear ribonucleoprotein A3 pseudogene 1	Nucleus	other		1,599	1.15×10^-02
ENSG00000260689.2	HNRNPA3P11	heterogeneous nuclear ribonucleoprotein A3 pseudogene 11	Other	other		1,651	2.71×10^-02
ENSG00000092199.17	HNRNPC	heterogeneous nuclear ribonucleoprotein C	Nucleus	other		-1,262	2.52×10^-02
ENSG00000138668.18	HNRNPD	heterogeneous nuclear ribonucleoprotein D	Nucleus	transcription regulator		-1,39	8.37×10^-03
ENSG00000169045.17	HNRNPH1	heterogeneous nuclear ribonucleoprotein H1	Nucleus	other		-1,114	2.37×10^-02
ENSG00000096746.17	HNRNPH3	heterogeneous nuclear ribonucleoprotein H3	Nucleus	other		-1,851	3.22×10^-04
ENSG00000165119.18	HNRNPK	heterogeneous nuclear ribonucleoprotein K	Nucleus	other		-2,301	2.80×10^-05
ENSG00000104824.16	HNRNPL	heterogeneous nuclear ribonucleoprotein L	Nucleus	other		-1,024	4.22×10^-02
ENSG00000099783.11	HNRNPM	heterogeneous nuclear ribonucleoprotein M	Nucleus	other		-2,56	2.92×10^-06
ENSG00000125944.18	HNRNPR	heterogeneous nuclear ribonucleoprotein R	Nucleus	other		-1,599	6.37×10^-03
ENSG00000105323.16	HNRNPUL1	heterogeneous nuclear ribonucleoprotein U like 1	Nucleus	other		-1,204	4.20×10^-02

ENSG00000105997.22	HOXA3	homeobox A3	Nucleus	transcription regulator		-1,946	9.96×10^-03
ENSG00000165704.14	HPRT1	hypoxanthine phosphoribosyltransferase 1	Cytoplasm	enzyme	6-mercaptopurine, thioguanine, azathioprine	-2,557	1.87×10^-02
ENSG00000113905.4	HRG	histidine rich glycoprotein	Extracellular Space	other		1,364	4.30×10^-02
ENSG00000108786.10	HSD17B1	hydroxysteroid 17-beta dehydrogenase 1	Cytoplasm	enzyme		1,304	3.47×10^-02
ENSG00000080824.18	HSP90AA1	heat shock protein 90 alpha family class A member 1	Cytoplasm	enzyme	alvespimycin, retaspimycin, luminespib, TAS	-2,049	7.85×10^-04
ENSG00000096384.19	HSP90AB1	heat shock protein 90 alpha family class B member 1	Cytoplasm	enzyme	alvespimycin, retaspimycin, TAS-116, cispla	-2,053	1.66×10^-03
ENSG00000166598.12	HSP90B1	heat shock protein 90 beta family member 1	Cytoplasm	other	alvespimycin, retaspimycin, cisplatin	-2,209	4.68×10^-05
ENSG00000204388.6	HSPA1A/HSPA1B	heat shock protein family A (Hsp70) member 1A	Cytoplasm	enzyme		2,264	6.94×10^-04
ENSG00000204389.9	HSPA1A/HSPA1B	heat shock protein family A (Hsp70) member 1A	Cytoplasm	enzyme		2,118	3.61×10^-04
ENSG00000126803.9	HSPA2	heat shock protein family A (Hsp70) member 2	Cytoplasm	other		1,537	1.14×10^-04
ENSG00000170606.13	HSPA4	heat shock protein family A (Hsp70) member 4	Cytoplasm	other		-1,191	4.39×10^-02
ENSG00000109971.13	HSPA8	heat shock protein family A (Hsp70) member 8	Cytoplasm	enzyme		-1,648	6.14×10^-03
ENSG00000113013.12	HSPA9	heat shock protein family A (Hsp70) member 9	Cytoplasm	other		-1,467	9.73×10^-03
ENSG00000106211.8	HSPB1	heat shock protein family B (small) member 1	Cytoplasm	other		-2,673	2.88×10^-06
ENSG00000144381.16	HSPD1	heat shock protein family D (Hsp60) member 1	Cytoplasm	enzyme		-2,114	7.93×10^-04
ENSG00000115541.10	HSPE1	heat shock protein family E (Hsp10) member 1	Cytoplasm	enzyme		-1,602	8.10×10^-03
ENSG00000142798.16	HSPG2	heparan sulfate proteoglycan 2	Extracellular Space	enzyme		-5,115	6.02×10^-17
ENSG00000086758.15	HUWE1	HECT, UBA and WWE domain containing E3 ubiquitin protein ligase 1	Nucleus	transcription regulator		-1,732	2.05×10^-03
ENSG00000149428.18	HYOU1	hypoxia up-regulated 1	Cytoplasm	other		-2,191	2.26×10^-04
ENSG00000067704.9	IARS2	isoleucyl-tRNA synthetase 2, mitochondrial	Cytoplasm	enzyme		-2,015	2.90×10^-03
ENSG00000108622.10	ICAM2	intercellular adhesion molecule 2	Plasma Membrane	other		-2,682	9.34×10^-03
ENSG00000125968.8	ID1	inhibitor of DNA binding 1, HLH protein	Nucleus	transcription regulator		1,64	2.77×10^-03
ENSG00000115738.9	ID2	inhibitor of DNA binding 2	Nucleus	transcription regulator		1,969	3.92×10^-04
ENSG00000160888.6	IER2	immediate early response 2	Cytoplasm	transcription regulator		3,286	2.83×10^-13
ENSG00000137331.11	IER3	immediate early response 3	Cytoplasm	other		1,624	4.64×10^-04
ENSG00000162783.10	IER5	immediate early response 5	Nucleus	other		1,939	1.35×10^-08
ENSG00000188483.7	IER5L	immediate early response 5 like	Other	other		1,385	1.39×10^-02
ENSG00000159217.9	IGF2BP1	insulin like growth factor 2 mRNA binding protein 1	Cytoplasm	translation regulator		-1,22	2.98×10^-02
ENSG00000073792.15	IGF2BP2	insulin like growth factor 2 mRNA binding protein 2	Cytoplasm	translation regulator		-2,461	1.16×10^-03
ENSG00000115457.9	IGFBP2	insulin like growth factor binding protein 2	Extracellular Space	other		-1,39	3.21×10^-02
ENSG00000141753.6	IGFBP4	insulin like growth factor binding protein 4	Extracellular Space	other		-2,685	2.35×10^-02
ENSG00000163453.11	IGFBP7	insulin like growth factor binding protein 7	Extracellular Space	transporter		-3,182	2.13×10^-02
ENSG00000113141.15	IK	IK cytokine	Extracellular Space	cytokine		-2,22	1.58×10^-04
ENSG00000113302.4	IL12B	interleukin 12B	Extracellular Space	cytokine	ustekinumab, methotrexate/ustekinumab	1,817	2.57×10^-03
ENSG00000115607.9	IL18RAP	interleukin 18 receptor accessory protein	Plasma Membrane	transmembrane receptor		2,264	4.69×10^-03
ENSG00000115602.16	IL1RL1	interleukin 1 receptor like 1	Plasma Membrane	transmembrane receptor		-1,579	6.56×10^-04
ENSG00000136244.11	IL6	interleukin 6	Extracellular Space	cytokine	anti-IL-6 monoclonal antibody, tocilizumab, s	1,774	5.62×10^-05
ENSG00000134352.19	IL6ST	interleukin 6 cytokine family signal transducer	Plasma Membrane	transmembrane receptor		-1,326	4.34×10^-03
ENSG00000106348.16	IMPDH1	inosine monophosphate dehydrogenase 1	Cytoplasm	enzyme	thioguanine, VX-944, pegintron/ribavirin, PE	-2,293	2.51×10^-02
ENSG00000149503.12	INCENP	inner centromere protein	Nucleus	other		-2,274	5.91×10^-05
ENSG00000153487.12	ING1	inhibitor of growth family member 1	Nucleus	transcription regulator		1,336	4.00×10^-03
ENSG00000164941.13	INTS8	integrator complex subunit 8	Nucleus	other		-1,887	4.89×10^-02
ENSG00000065150.18	IPO5	importin 5	Nucleus	transporter		-1,503	4.65×10^-03
ENSG00000205339.9	IPO7	importin 7	Nucleus	transporter		-1,638	2.38×10^-03
ENSG00000140575.12	IQGAP1	IQ motif containing GTPase activating protein 1	Cytoplasm	other		-2,776	1.09×10^-07
ENSG00000183856.10	IQGAP3	IQ motif containing GTPase activating protein 3	Plasma Membrane	other		-1,428	4.00×10^-02
ENSG00000184216.11	IRAK1	interleukin 1 receptor associated kinase 1	Plasma Membrane	kinase	1-(3-hydroxypropyl)-2-[(3-nitrobenzoyl)amin	-1,7	1.68×10^-02
ENSG00000119669.4	IRF2BPL	interferon regulatory factor 2 binding protein like	Nucleus	enzyme		1,813	2.05×10^-03
ENSG00000185950.8	IRS2	insulin receptor substrate 2	Cytoplasm	enzyme	NT219	1,388	1.28×10^-03
ENSG00000170561.12	IRX2	iroquois homeobox 2	Nucleus	transcription regulator		2,247	1.43×10^-05
ENSG00000177508.11	IRX3	iroquois homeobox 3	Nucleus	transcription regulator		1,564	3.43×10^-02
ENSG00000161638.10	ITGA5	integrin subunit alpha 5	Plasma Membrane	transmembrane receptor		-2,639	1.97×10^-06
ENSG00000091409.14	ITGA6	integrin subunit alpha 6	Plasma Membrane	transmembrane receptor		-2,237	2.08×10^-04
ENSG00000150093.18	ITGB1	integrin subunit beta 1	Plasma Membrane	transmembrane receptor	OS2966	-4,397	1.65×10^-12
ENSG00000123104.11	ITPR2	inositol 1,4,5-trisphosphate receptor type 2	Cytoplasm	ion channel		-1,256	4.76×10^-02
ENSG00000163166.13	IWS1	interacts with SUPT6H, CTD assembly factor 1	Nucleus	enzyme		-1,88	9.76×10^-05
ENSG00000162434.11	JAK1	Janus kinase 1	Cytoplasm	kinase	SHR0302, solcitinib, tofacitinib, ruxolitinib, m	-2,914	3.95×10^-06
ENSG00000177606.6	JUN	Jun proto-oncogene, AP-1 transcription factor subunit	Nucleus	transcription regulator		2,251	9.87×10^-06
ENSG00000171223.5	JUNB	JunB proto-oncogene, AP-1 transcription factor subunit	Nucleus	transcription regulator		3,89	1.09×10^-16
ENSG00000130522.5	JUND	JunD proto-oncogene, AP-1 transcription factor subunit	Nucleus	transcription regulator		4,121	1.13×10^-19
ENSG00000173801.16	JUP	junction plakoglobin	Plasma Membrane	other		-2,13	2.57×10^-03
ENSG00000065427.14	KARS1	lysyl-tRNA synthetase 1	Cytoplasm	enzyme		-1,818	2.07×10^-02
ENSG00000178695.5	KCTD12	potassium channel tetramerization domain containing 12	Plasma Membrane	ion channel		-1,8	1.28×10^-03
ENSG00000112078.13	KCTD20	potassium channel tetramerization domain containing 20	Cytoplasm	other		-1,314	1.93×10^-02
ENSG00000105438.8	KDELR1	KDEL endoplasmic reticulum protein retention receptor 1	Cytoplasm	transporter		-2,529	9.98×10^-03
ENSG00000004487.15	KDM1A	lysine demethylase 1A	Nucleus	enzyme	TAS1440, GSK-2879552, RO7051790, IMG	-1,762	6.01×10^-03
ENSG00000128052.8	KDR	kinase insert domain receptor	Plasma Membrane	kinase	CHIR-265, midostaurin, PDGF receptor tyro	-2,745	1.31×10^-02
ENSG00000121774.17	KHDRBS1	KH RNA binding domain containing, signal transduction associated 1	Nucleus	transcription regulator	CWP232291	-2,008	1.64×10^-03
ENSG00000110427.14	KIAA1549L	KIAA1549 like	Cytoplasm	other		-1,019	2.55×10^-02
ENSG00000197077.12	KIAA1671	KIAA1671	Other	other		-1,186	1.22×10^-02
ENSG00000222035.3	KIAA2012-AS1	KIAA2012 antisense RNA 1	Other	other		1,381	3.43×10^-02
ENSG00000138160.5	KIF11	kinesin family member 11	Nucleus	other	ispinesib, AZD4877, MK-0731, filanesib, SB-	-1,701	3.62×10^-02
ENSG00000129250.11	KIF1C	kinesin family member 1C	Cytoplasm	other		-3,396	9.33×10^-08
ENSG00000112984.11	KIF20A	kinesin family member 20A	Cytoplasm	other		-1,985	2.51×10^-02
ENSG00000139116.17	KIF21A	kinesin family member 21A	Cytoplasm	other		-1,294	1.25×10^-02
ENSG00000101350.7	KIF3B	kinesin family member 3B	Cytoplasm	transporter		-1,293	5.77×10^-03
ENSG00000170759.10	KIF5B	kinesin family member 5B	Cytoplasm	other		-2,084	4.74×10^-05
ENSG00000127528.5	KLF2	Kruppel like factor 2	Nucleus	transcription regulator		2,779	1.06×10^-04
ENSG00000136826.14	KLF4	Kruppel like factor 4	Nucleus	transcription regulator		1,151	2.34×10^-02
ENSG00000128944.13	KNSTRN	kinetochore localized astrin (SPAG5) binding protein	Cytoplasm	other		-1,491	1.91×10^-02
ENSG00000182481.8	KPNA2	karyopherin subunit alpha 2	Nucleus	other		-2,184	6.60×10^-04
ENSG00000102753.9	KPNA3	karyopherin subunit alpha 3	Nucleus	transporter		-1,799	1.36×10^-02
ENSG00000025800.13	KPNA6	karyopherin subunit alpha 6	Nucleus	other		-1,225	2.21×10^-02
ENSG00000108424.9	KPNB1	karyopherin subunit beta 1	Nucleus	other		-1,687	1.12×10^-03
ENSG00000186395.6	KRT10	keratin 10	Cytoplasm	other		-2,381	2.05×10^-02
ENSG00000215606.4	KRT18P35	keratin 18 pseudogene 35	Other	other		5,019	4.95×10^-05
ENSG00000171403.9	KRT9	keratin 9	Cytoplasm	other		1,134	3.12×10^-02
ENSG00000105700.10	KXD1	KxDL motif containing 1	Cytoplasm	other		-2,171	3.49×10^-03
ENSG00000198945.7	L3MBTL3	L3MBTL histone methyl-lysine binding protein 3	Nucleus	other		-2,302	3.68×10^-03
ENSG00000112769.18	LAMA4	laminin subunit alpha 4	Extracellular Space	enzyme		-2,73	6.81×10^-07
ENSG00000091136.13	LAMB1	laminin subunit beta 1	Extracellular Space	other		-2,571	1.41×10^-06
ENSG00000172037.13	LAMB2	laminin subunit beta 2	Extracellular Space	enzyme		-1,659	2.94×10^-02
ENSG00000135862.5	LAMC1	laminin subunit gamma 1	Extracellular Space	other		-1,551	1.68×10^-03
ENSG00000155506.16	LARP1	La ribonucleoprotein 1, translational regulator	Cytoplasm	translation regulator		-1,227	2.65×10^-02
ENSG00000001497.16	LAS1L	LAS1 like ribosome biogenesis factor	Nucleus	other		-1,159	2.42×10^-02
ENSG00000150457.8	LATS2	large tumor suppressor kinase 2	Nucleus	kinase		-1,351	3.80×10^-02
ENSG00000134333.13	LDHA	lactate dehydrogenase A	Cytoplasm	enzyme		-2,488	1.89×10^-06
ENSG00000111716.12	LDHB	lactate dehydrogenase B	Cytoplasm	enzyme		-2,088	1.86×10^-04
ENSG00000130164.11	LDLR	low density lipoprotein receptor	Plasma Membrane	transporter	iodine I 131 iodocholesterol	-1,318	2.92×10^-02
ENSG00000100097.11	LGALS1	galectin 1	Extracellular Space	other	OTX008	-3,495	3.99×10^-05
ENSG00000131981.15	LGALS3	galectin 3	Extracellular Space	other	GCS-100, GR-MD-02	-1,343	2.73×10^-02
ENSG00000244968.6	LIFR-AS1	LIFR antisense RNA 1	Other	other		1,6	4.19×10^-03
ENSG00000064042.17	LIMCH1	LIM and calponin homology domains 1	Cytoplasm	other		-1,499	9.13×10^-03
ENSG00000265933.5	LINC00668	long intergenic non-protein coding RNA 668	Other	other		1,128	3.65×10^-02
ENSG00000203721.5	LINC00862	long intergenic non-protein coding RNA 862	Other	other		1,73	1.00×10^-02
ENSG00000246898.1	LINC00920	long intergenic non-protein coding RNA 920	Other	other		1,913	2.09×10^-02
ENSG00000235314.1	LINC00957	long intergenic non-protein coding RNA 957	Other	other		2,067	3.07×10^-02
ENSG00000244041.7	LINC01011	long intergenic non-protein coding RNA 1011	Other	other		2,483	4.84×10^-05
ENSG00000233304.5	LINC01346	long intergenic non-protein coding RNA 1346	Other	other		1,744	1.48×10^-02
ENSG00000237094.11	LINC01347 (includes others)	long intergenic non-protein coding RNA 1347	Other	other		1,441	4.07×10^-03
ENSG00000253686.1	LINC01484	long intergenic non-protein coding RNA 1484	Other	other		1,847	1.45×10^-05
ENSG00000280350.1	LINC01726	long intergenic non-protein coding RNA 1726	Other	other		2,607	2.24×10^-03
ENSG00000220161.4	LINC02076	long intergenic non-protein coding RNA 2076	Other	other		1,394	2.05×10^-02
ENSG00000261122.6	LINC02167	long intergenic non-protein coding RNA 2167	Other	other		2,147	3.39×10^-02
ENSG00000260792.1	LINC02280	long intergenic non-protein coding RNA 2280	Other	other		1,416	7.32×10^-04
ENSG00000074695.5	LMAN1	lectin, mannose binding 1	Cytoplasm	other		-1,514	2.60×10^-02
ENSG00000169223.14	LMAN2	lectin, mannose binding 2	Cytoplasm	transporter		-1,702	4.95×10^-02
ENSG00000113368.11	LMNB1	lamin B1	Nucleus	other		-1,384	2.21×10^-02
ENSG00000206535.7	LNP1	leukemia NUP98 fusion partner 1	Other	other		2,117	1.88×10^-04
ENSG00000227717.4	LOC100132202/LOC645202	golgin A6 family-like	Other	other		1,098	1.18×10^-02
ENSG00000230387.2	LOC100505664	uncharacterized LOC100505664	Other	other		2,155	6.66×10^-08
ENSG00000241860.6	LOC100996442		Other	other		1,146	1.69×10^-02
ENSG00000227733.8	LOC101927468	uncharacterized LOC101927468	Other	other		1,21	2.57×10^-03
ENSG00000232732.9	LOC101927687	uncharacterized LOC101927687	Other	other		1,411	2.15×10^-02
ENSG00000261136.1	LOC105370941	uncharacterized LOC105370941	Other	other		1,233	4.07×10^-02
ENSG00000231918.1	LOC730100	uncharacterized LOC730100	Other	other		1,143	1.59×10^-02
ENSG00000134013.15	LOXL2	lysyl oxidase like 2	Extracellular Space	enzyme	PAT-1251, simtuzumab	-2,538	7.17×10^-04
ENSG00000077454.15	LRCH4	leucine rich repeats and calponin homology domain containing 4	Cytoplasm	transcription regulator		1,623	7.42×10^-05
ENSG00000138095.18	LRPPRC	leucine rich pentatricopeptide repeat containing	Cytoplasm	other		-1,181	4.20×10^-02
ENSG00000136802.11	LRRC8A	leucine rich repeat containing 8 VRAC subunit A	Plasma Membrane	ion channel		-1,615	3.20×10^-03
ENSG00000161654.9	LSM12	LSM12 homolog	Other	other		-1,672	2.89×10^-02
ENSG00000119681.11	LTBP2	latent transforming growth factor beta binding protein 2	Extracellular Space	other		-1,409	6.90×10^-03
ENSG00000135521.8	LTV1	LTV1 ribosome biogenesis factor	Other	other		-2,329	2.34×10^-03
ENSG00000146963.17	LUC7L2	LUC7 like 2, pre-mRNA splicing factor	Nucleus	other		-1,608	3.05×10^-03
ENSG00000169641.13	LUZP1	leucine zipper protein 1	Nucleus	other		-1,299	2.09×10^-03
ENSG00000127603.23	MACF1	microtubule actin crosslinking factor 1	Cytoplasm	other		-2,071	2.72×10^-05
ENSG00000110514.18	MADD	MAP kinase activating death domain	Cytoplasm	other		-2,357	1.46×10^-02
ENSG00000182759.3	MAFA	MAF bZIP transcription factor A	Nucleus	transcription regulator		2,164	1.61×10^-02
ENSG00000204103.3	MAFB	MAF bZIP transcription factor B	Nucleus	transcription regulator		1,701	4.99×10^-04
ENSG00000198517.9	MAFK	MAF bZIP transcription factor K	Nucleus	transcription regulator		-2,002	1.68×10^-03
ENSG00000179222.17	MAGED1	MAGE family member D1	Plasma Membrane	transcription regulator		-1,822	7.53×10^-03
ENSG00000198162.12	MAN1A2	mannosidase alpha class 1A member 2	Cytoplasm	enzyme		-1,657	2.55×10^-04
ENSG00000131711.14	MAP1B	microtubule associated protein 1B	Cytoplasm	other		-1,468	1.86×10^-03
ENSG00000047849.21	MAP4	microtubule associated protein 4	Cytoplasm	other		-1,178	1.42×10^-02
ENSG00000071054.15	MAP4K4	mitogen-activated protein kinase kinase kinase kinase 4	Cytoplasm	kinase	PF-06260933, GNE-495, NCB-0846	-2,023	5.68×10^-06
ENSG00000184368.15	MAP7D2	MAP7 domain containing 2	Other	other		2,496	1.37×10^-03
ENSG00000101367.8	MAPRE1	microtubule associated protein RP/EB family member 1	Cytoplasm	other		-1,665	2.48×10^-03
ENSG00000166974.12	MAPRE2	microtubule associated protein RP/EB family member 2	Cytoplasm	other		-1,825	2.58×10^-03
ENSG00000277443.1	MARCKS	myristoylated alanine rich protein kinase C substrate	Plasma Membrane	other	BIO-11006	-1,875	1.68×10^-03
ENSG00000175130.6	MARCKSL1	MARCKS like 1	Cytoplasm	other		-2,567	4.97×10^-03
ENSG00000125952.18	MAX	MYC associated factor X	Nucleus	transcription regulator		-1,469	1.39×10^-02
ENSG00000103495.13	MAZ	MYC associated zinc finger protein	Nucleus	transcription regulator		3,736	4.67×10^-02
ENSG00000134046.11	MBD2	methyl-CpG binding domain protein 2	Nucleus	transcription regulator		-1,228	6.16×10^-03
ENSG00000152601.17	MBNL1	muscleblind like splicing regulator 1	Nucleus	other		-1,905	1.28×10^-03
ENSG00000140943.16	MBTPS1	membrane bound transcription factor peptidase, site 1	Cytoplasm	peptidase	sonepcizumab	-1,273	3.80×10^-02
ENSG00000076706.14	MCAM	melanoma cell adhesion molecule	Plasma Membrane	other		-3,029	4.04×10^-07
ENSG00000076003.4	MCM6	minichromosome maintenance complex component 6	Nucleus	enzyme		-1,653	4.94×10^-02
ENSG00000137337.14	MDC1	mediator of DNA damage checkpoint 1	Nucleus	other		-1,205	9.99×10^-03
ENSG00000146701.11	MDH2	malate dehydrogenase 2	Cytoplasm	enzyme		-1,687	1.30×10^-02
ENSG00000124733.3	MEA1	male-enhanced antigen 1	Cytoplasm	other		-2,668	3.57×10^-02

ENSG00000165304.7	MELK	maternal embryonic leucine zipper kinase	Cytoplasm	kinase	OTS167	-3,547	2.09×10^-02
ENSG00000140259.6	MFAP1	microfibril associated protein 1	Extracellular Space	other		-1,205	3.48×10^-02
ENSG00000117122.13	MFAP2	microfibril associated protein 2	Extracellular Space	other		-2,565	1.88×10^-02
ENSG00000197614.10	MFAP5	microfibril associated protein 5	Extracellular Space	other		1,929	2.59×10^-02
ENSG00000116688.16	MFN2	mitofusin 2	Cytoplasm	enzyme		-1,63	2.89×10^-02
ENSG00000152127.8	MGAT5	alpha-1,6-mannosylglycoprotein 6-beta-N-acetylglucosaminyltransferase	Cytoplasm	enzyme		-1,544	3.59×10^-03
ENSG00000111341.9	MGP	matrix Gla protein	Extracellular Space	other		-2,742	8.65×10^-04
ENSG00000101752.11	MIB1	MIB E3 ubiquitin protein ligase 1	Cytoplasm	enzyme		-1,344	6.83×10^-03
ENSG00000133816.13	MICAL2	microtubule associated monooxygenase, calponin and LIM domain containing 2	Cytoplasm	enzyme		-1,243	1.30×10^-02
ENSG00000100139.13	MICALL1	MICAL like 1	Cytoplasm	other		-2,191	3.47×10^-02
ENSG00000240972.1	MIF	macrophage migration inhibitory factor	Extracellular Space	cytokine	imalumab	-1,964	2.06×10^-03
ENSG00000215417.10	MIR17HG	miR-17-92a-1 cluster host gene	Other	other		2,033	7.92×10^-03
ENSG00000148773.12	MKI67	marker of proliferation Ki-67	Nucleus	other		-1,12	1.30×10^-02
ENSG00000196611.4	MMP1	matrix metallopeptidase 1	Extracellular Space	peptidase	rebimastat, marimastat	-9,035	5.05×10^-13
ENSG00000157227.12	MMP14	matrix metallopeptidase 14	Extracellular Space	peptidase	rebimastat, marimastat, prinomastat, BT171	-2,688	1.40×10^-04
ENSG00000087245.12	MMP2	matrix metallopeptidase 2	Extracellular Space	peptidase	prinomastat, rebimastat, marimastat, MMP2	-3,445	3.46×10^-03
ENSG00000138722.9	MMRN1	multimerin 1	Extracellular Space	other		-4,028	1.20×10^-11
ENSG00000173269.13	MMRN2	multimerin 2	Extracellular Space	other		-1,577	1.12×10^-02
ENSG00000185787.14	MORF4L1	mortality factor 4 like 1	Nucleus	other		-1,453	7.11×10^-03
ENSG00000179010.14	MRFAP1	Morf4 family associated protein 1	Nucleus	other		-1,41	1.93×10^-02
ENSG00000137547.8	MRPL15	mitochondrial ribosomal protein L15	Cytoplasm	other		-2,664	9.26×10^-03
ENSG00000147065.16	MSN	moesin	Plasma Membrane	other		-2,897	5.33×10^-08
ENSG00000198804.2	MT-CO1	cytochrome c oxidase subunit I	Cytoplasm	enzyme	naproxen/sumatriptan, esomeprazole/naprox	-1,573	2.55×10^-02
ENSG00000198938.2	MT-CO3	cytochrome c oxidase subunit III	Cytoplasm	enzyme		-1,743	3.02×10^-03
ENSG00000125148.6	MT2A	metallothionein 2A	Cytoplasm	other		-4,35	1.88×10^-12
ENSG00000248527.1	MTATP6P1	MT-ATP6 pseudogene 1	Other	other		-1,2	2.36×10^-02
ENSG00000236590.1	MTATP6P4	MT-ATP6 pseudogene 4	Other	other		1,308	1.20×10^-02
ENSG00000137409.18	MTCH1	mitochondrial carrier 1	Cytoplasm	other		-1,952	4.16×10^-02
ENSG00000233550.1	MTCYBP8		Other	other		2,557	1.93×10^-02
ENSG00000120254.15	MTHFD1L	methylenetetrahydrofolate dehydrogenase (NADP+ dependent) 1 like	Cytoplasm	enzyme		-2,608	3.08×10^-02
ENSG00000263914.1	MTND2P13	MT-ND2 pseudogene 13	Other	other		1,751	3.45×10^-02
ENSG00000225630.1	MTND2P28	MT-ND2 pseudogene 28	Other	other		2,075	3.57×10^-02
ENSG00000247627.2	MTND4P12	MT-ND4 pseudogene 12	Other	other		1,578	4.14×10^-03
ENSG00000225796.2	MTND4P23	MT-ND4 pseudogene 23	Other	other		1,934	5.00×10^-03
ENSG00000230548.1	MTND4P27	MT-ND4 pseudogene 27	Other	other		1,689	4.16×10^-02
ENSG00000270906.1	MTND4P35		Other	other		1,52	4.75×10^-02
ENSG00000270230.1	MTND6P22		Other	other		2,524	1.43×10^-03
ENSG00000249119.1	MTND6P4		Other	other		1,926	2.31×10^-02
ENSG00000129422.13	MTUS1	microtubule associated scaffold protein 1	Extracellular Space	other		-1,249	5.18×10^-03
ENSG00000184956.15	MUC6	mucin 6, oligomeric mucus/gel-forming	Extracellular Space	other		1,247	1.32×10^-03
ENSG00000013364.18	MVP	major vault protein	Nucleus	other		-2,823	4.74×10^-03
ENSG00000185697.16	MYBL1	MYB proto-oncogene like 1	Nucleus	transcription regulator		3,487	7.84×10^-11
ENSG00000136997.14	MYC	MYC proto-oncogene, bHLH transcription factor	Nucleus	transcription regulator	MYC-targeting siRNA DCR-MYC, AVI-4126	1,105	8.23×10^-03
ENSG00000005810.17	MYCBP2	MYC binding protein 2	Nucleus	enzyme		-1,45	4.24×10^-03
ENSG00000133026.12	MYH10	myosin heavy chain 10	Cytoplasm	enzyme		-2,643	1.10×10^-05
ENSG00000100345.20	MYH9	myosin heavy chain 9	Cytoplasm	enzyme		-1,466	4.14×10^-02
ENSG00000101608.12	MYL12A	myosin light chain 12A	Cytoplasm	other		-2,751	4.07×10^-06
ENSG00000118680.12	MYL12B	myosin light chain 12B	Cytoplasm	other		-3,092	1.69×10^-03
ENSG00000092841.18	MYL6	myosin light chain 6	Cytoplasm	enzyme		-2,111	3.04×10^-04
ENSG00000145555.14	MYO10	myosin X	Cytoplasm	enzyme		-1,238	2.11×10^-02
ENSG00000231898.8	MYO3B-AS1	MYO3B antisense RNA 1	Other	other		1,587	1.26×10^-02
ENSG00000138119.16	MYOF	myoferlin	Nucleus	other		-2,471	3.00×10^-05
ENSG00000244754.8	N4BP2L2	NEDD4 binding protein 2 like 2	Nucleus	transcription regulator		1,895	3.25×10^-05
ENSG00000196531.10	NACA	nascent polypeptide associated complex subunit alpha	Cytoplasm	transcription regulator		-1,39	1.39×10^-02
ENSG00000187109.13	NAP1L1	nucleosome assembly protein 1 like 1	Nucleus	other		-1,501	6.73×10^-03
ENSG00000205531.12	NAP1L4	nucleosome assembly protein 1 like 4	Cytoplasm	other		-2,38	7.42×10^-05
ENSG00000132780.16	NASP	nuclear autoantigenic sperm protein	Nucleus	other		-1,406	7.46×10^-03
ENSG00000010292.12	NCAPD2	non-SMC condensin I complex subunit D2	Nucleus	other		-1,446	2.01×10^-02
ENSG00000109805.9	NCAPG	non-SMC condensin I complex subunit G	Nucleus	other		-1,848	1.07×10^-02
ENSG00000213212.3	NCLP1	nucleolin pseudogene 1	Other	other		1,881	3.84×10^-02
ENSG00000184983.9	NDUFA6	NADH:ubiquinone oxidoreductase subunit A6	Cytoplasm	enzyme		-2	5.06×10^-03
ENSG00000004779.9	NDUFAB1	NADH:ubiquinone oxidoreductase subunit AB1	Cytoplasm	enzyme		-2,716	5.26×10^-03
ENSG00000224877.3	NDUFAF8	NADH:ubiquinone oxidoreductase complex assembly factor 8	Cytoplasm	other		-2,469	6.75×10^-03
ENSG00000140990.14	NDUFB10	NADH:ubiquinone oxidoreductase subunit B10	Cytoplasm	enzyme		-1,681	1.98×10^-02
ENSG00000259374.2	NDUFB4P11		Other	other		1,272	4.75×10^-02
ENSG00000164258.11	NDUFS4	NADH:ubiquinone oxidoreductase subunit S4	Cytoplasm	enzyme		-2,195	4.39×10^-02
ENSG00000168653.10	NDUFS5	NADH:ubiquinone oxidoreductase subunit S5	Cytoplasm	enzyme		-2,976	4.96×10^-05
ENSG00000245532.5	NEAT1	nuclear paraspeckle assembly transcript 1	Other	other		-1,598	1.20×10^-02
ENSG00000132688.10	NES	nestin	Extracellular Space	other		-2,16	7.23×10^-05
ENSG00000173848.18	NET1	neuroepithelial cell transforming 1	Nucleus	other		-2,754	1.97×10^-06
ENSG00000186575.17	NF2	neurofibromin 2	Plasma Membrane	other		-1,344	4.14×10^-02
ENSG00000263412.1	NFE2L1-DT		Other	other		1,866	5.20×10^-03
ENSG00000100906.10	NFKBIA	NFKB inhibitor alpha	Cytoplasm	transcription regulator		3,617	6.61×10^-12
ENSG00000145912.8	NHP2	NHP2 ribonucleoprotein	Nucleus	other		-1,664	1.97×10^-02
ENSG00000155438.11	NIFK	nucleolar protein interacting with the FHA domain of MKI67	Nucleus	other		-1,449	2.11×10^-02
ENSG00000100503.23	NIN	ninein	Cytoplasm	other		-1,376	3.05×10^-03
ENSG00000239672.7	NME1	NME/NM23 nucleoside diphosphate kinase 1	Cytoplasm	kinase		-1,246	2.51×10^-02
ENSG00000188976.10	NOC2L	NOC2 like nucleolar associated transcriptional repressor	Nucleus	transcription regulator		-1,802	1.39×10^-03
ENSG00000103512.14	NOMO1 (includes others)	NODAL modulator 1	Plasma Membrane	other		-1,931	3.10×10^-03
ENSG00000182117.5	NOP10	NOP10 ribonucleoprotein	Nucleus	other		-3,089	3.63×10^-02
ENSG00000087269.15	NOP14	NOP14 nucleolar protein	Nucleus	other		-1,431	2.05×10^-02
ENSG00000148400.9	NOTCH1	notch receptor 1	Plasma Membrane	transcription regulator	OMP-52M51	-2,415	5.14×10^-05
ENSG00000214967.5	NPIPA5 (includes others)	nuclear pore complex interacting protein family member A5	Nucleus	other		7,047	3.70×10^-07
ENSG00000185864.16	NPIPB5 (includes others)	nuclear pore complex interacting protein family member B5	Nucleus	other		1,245	3.02×10^-03
ENSG00000181163.13	NPM1	nucleophosmin 1	Nucleus	transcription regulator		-2,166	2.49×10^-04
ENSG00000223864.1	NPM1P19	nucleophosmin 1 pseudogene 19	Other	other		1,78	4.16×10^-02
ENSG00000107833.10	NPM3	nucleophosmin/nucleoplasmin 3	Nucleus	other		-2,458	4.20×10^-02
ENSG00000181019.12	NQO1	NAD(P)H quinone dehydrogenase 1	Cytoplasm	enzyme		-1,321	3.90×10^-02
ENSG00000175745.11	NR2F1	nuclear receptor subfamily 2 group F member 1	Nucleus	ligand-dependent nuclear receptor		1,148	8.37×10^-03
ENSG00000123358.19	NR4A1	nuclear receptor subfamily 4 group A member 1	Nucleus	ligand-dependent nuclear receptor		1,368	4.92×10^-03
ENSG00000198435.3	NRARP	NOTCH regulated ankyrin repeat protein	Nucleus	transcription regulator		3,757	8.17×10^-14
ENSG00000078618.19	NRDC	nardilysin convertase	Cytoplasm	peptidase		-2,004	2.67×10^-04
ENSG00000099250.17	NRP1	neuropilin 1	Plasma Membrane	transmembrane receptor	vesencumab	-1,904	2.84×10^-05
ENSG00000164346.9	NSA2	NSA2 ribosome biogenesis factor	Nucleus	other		-1,839	6.23×10^-03
ENSG00000178694.9	NSUN3	NOP2/Sun RNA methyltransferase 3	Cytoplasm	enzyme		1,563	2.52×10^-02
ENSG00000141698.16	NT5C3B	5'-nucleotidase, cytosolic IIIB	Cytoplasm	phosphatase		-2,056	4.78×10^-02
ENSG00000135318.11	NT5E	5'-nucleotidase ecto	Plasma Membrane	phosphatase	Sym024, MEDI9447, CPI-006, NZV930, BM	-2,008	5.65×10^-03
ENSG00000163545.8	NUAK2	NUAK family kinase 2	Nucleus	kinase		1,68	5.00×10^-03
ENSG00000090273.13	NUDC	nuclear distribution C, dynein complex regulator	Cytoplasm	other		-2,093	2.67×10^-04
ENSG00000198585.11	NUDT16	nudix hydrolase 16	Nucleus	enzyme		-2,216	9.69×10^-06
ENSG00000272325.1	NUDT3	nudix hydrolase 3	Cytoplasm	phosphatase		-1,446	1.48×10^-02
ENSG00000108256.8	NUFIP2	nuclear FMR1 interacting protein 2	Cytoplasm	other		1,076	1.41×10^-02
ENSG00000155561.14	NUP205	nucleoporin 205	Nucleus	other		-1,545	2.33×10^-02
ENSG00000143552.9	NUP210L	nucleoporin 210 like	Other	other		3,803	4.63×10^-07
ENSG00000162231.13	NXF1	nuclear RNA export factor 1	Nucleus	other		-1,407	4.96×10^-02
ENSG00000065154.11	OAT	ornithine aminotransferase	Cytoplasm	enzyme		-1,807	4.49×10^-02
ENSG00000115758.12	ODC1	ornithine decarboxylase 1	Cytoplasm	enzyme	tazarotene, eflornithine	-1,743	2.57×10^-03
ENSG00000217315.1	OR2W2P	olfactory receptor family 2 subfamily W member 2 pseudogene	Other	other		3,593	3.59×10^-07
ENSG00000176253.2	OR4K13	olfactory receptor family 4 subfamily K member 13	Plasma Membrane	other		2,435	3.60×10^-02
ENSG00000135506.15	OS9	OS9 endoplasmic reticulum lectin	Nucleus	other		-1,194	3.93×10^-02
ENSG00000228474.5	OST4	oligosaccharyltransferase complex subunit 4, non-catalytic	Cytoplasm	other		-2,943	1.12×10^-03
ENSG00000068308.13	OTUD5	OTU deubiquitinase 5	Cytoplasm	enzyme		-2,486	7.84×10^-03
ENSG00000185624.14	P4HB	prolyl 4-hydroxylase subunit beta	Cytoplasm	enzyme		-1,608	4.66×10^-03
ENSG00000170515.13	PA2G4	proliferation-associated 2G4	Nucleus	transcription regulator		-1,402	1.12×10^-02
ENSG00000070756.13	PABPC1	poly(A) binding protein cytoplasmic 1	Cytoplasm	translation regulator		-2,059	3.87×10^-04
ENSG00000090621.13	PABPC4	poly(A) binding protein cytoplasmic 4	Cytoplasm	translation regulator		-1,314	3.55×10^-02
ENSG00000175115.11	PACS1	phosphofurin acidic cluster sorting protein 1	Cytoplasm	other		-1,241	3.67×10^-02
ENSG00000006712.14	PAF1	PAF1 homolog, Paf1/RNA polymerase II complex component	Nucleus	other		-2,125	4.74×10^-03
ENSG00000168092.13	PAFAH1B2	platelet activating factor acetylhydrolase 1b catalytic subunit 2	Cytoplasm	enzyme		-1,536	6.28×10^-03
ENSG00000116288.12	PARK7	Parkinsonism associated deglycase	Nucleus	enzyme		-2,373	2.62×10^-02
ENSG00000143799.12	PARP1	poly(ADP-ribose) polymerase 1	Nucleus	enzyme	talazoparib, SC10914, olaparib, ABT-767, ru	-1,433	9.98×10^-03
ENSG00000102699.5	PARP4	poly(ADP-ribose) polymerase family member 4	Cytoplasm	enzyme		-2,312	5.79×10^-04
ENSG00000152931.7	PART1	prostate androgen-regulated transcript 1	Other	other		1,087	4.90×10^-03
ENSG00000188677.14	PARVB	parvin beta	Cytoplasm	other		1,528	1.06×10^-02
ENSG00000231806.2	PCAT7	prostate cancer associated transcript 7	Other	other		1,688	4.20×10^-02
ENSG00000166228.8	PCBD1	pterin-4 alpha-carbinolamine dehydratase 1	Nucleus	transcription regulator		-3,462	2.35×10^-02
ENSG00000169564.6	PCBP1	poly(rC) binding protein 1	Nucleus	translation regulator		-1,862	1.40×10^-03
ENSG00000197111.15	PCBP2	poly(rC) binding protein 2	Nucleus	other		-1,598	3.89×10^-03
ENSG00000114054.13	PCCB	propionyl-CoA carboxylase subunit beta	Cytoplasm	enzyme		-1,899	3.24×10^-02
ENSG00000132646.10	PCNA	proliferating cell nuclear antigen	Nucleus	enzyme		-2,141	2.05×10^-03
ENSG00000081154.11	PCNP	PEST proteolytic signal containing nuclear protein	Nucleus	other		-2,084	1.06×10^-02
ENSG00000148843.13	PDCD11	programmed cell death 11	Nucleus	other		-1,318	3.47×10^-02
ENSG00000115539.13	PDCL3	phosducin like 3	Cytoplasm	other		-1,907	1.78×10^-02
ENSG00000156973.13	PDE6D	phosphodiesterase 6D	Cytoplasm	enzyme		1,685	3.73×10^-02
ENSG00000131828.13	PDHA1	pyruvate dehydrogenase E1 subunit alpha 1	Cytoplasm	enzyme		-1,858	8.37×10^-03
ENSG00000168291.12	PDHB	pyruvate dehydrogenase E1 subunit beta	Cytoplasm	enzyme		-2,058	1.39×10^-02
ENSG00000167004.12	PDIA3	protein disulfide isomerase family A member 3	Cytoplasm	peptidase		-2,708	5.01×10^-06
ENSG00000155660.10	PDIA4	protein disulfide isomerase family A member 4	Cytoplasm	enzyme		-2,86	2.52×10^-07
ENSG00000143870.12	PDIA6	protein disulfide isomerase family A member 6	Cytoplasm	enzyme		-3,729	8.36×10^-10
ENSG00000107438.8	PDLIM1	PDZ and LIM domain 1	Cytoplasm	transcription regulator		-2,312	1.98×10^-02
ENSG00000261371.5	PECAM1	platelet and endothelial cell adhesion molecule 1	Plasma Membrane	other		-3,404	1.69×10^-09
ENSG00000141456.14	PELP1	proline, glutamate and leucine rich protein 1	Nucleus	other		-1,406	1.39×10^-02
ENSG00000112378.11	PERP	p53 apoptosis effector related to PMP22	Plasma Membrane	other		-1,46	2.06×10^-02
ENSG00000100029.17	PES1	pescadillo ribosomal biogenesis factor 1	Nucleus	other		-1,228	3.57×10^-02
ENSG00000162928.8	PEX13	peroxisomal biogenesis factor 13	Cytoplasm	transporter		1,142	1.25×10^-02
ENSG00000142657.20	PGD	phosphogluconate dehydrogenase	Cytoplasm	enzyme		-1,676	3.89×10^-03
ENSG00000215021.8	PHB2	prohibitin 2	Cytoplasm	transcription regulator		-2,065	8.65×10^-04
ENSG00000106443.14	PHF14	PHD finger protein 14	Nucleus	other		-1,919	2.29×10^-04
ENSG00000139289.13	PHLDA1	pleckstrin homology like domain family A member 1	Cytoplasm	other		1,348	5.33×10^-03
ENSG00000181649.5	PHLDA2	pleckstrin homology like domain family A member 2	Cytoplasm	other		2,71	8.58×10^-09
ENSG00000073921.17	PICALM	phosphatidylinositol binding clathrin assembly protein	Cytoplasm	other		-2,114	7.30×10^-03
ENSG00000011405.13	PIK3C2A	phosphatidylinositol-4-phosphate 3-kinase catalytic subunit type 2 alpha	Cytoplasm	kinase		-1,31	4.74×10^-02
ENSG00000180957.17	PITPNB	phosphatidylinositol transfer protein beta	Cytoplasm	transporter		-2,282	3.38×10^-03
ENSG00000107959.15	PITRM1	pitrilysin metallopeptidase 1	Cytoplasm	peptidase		-1,581	3.47×10^-02
ENSG00000198961.9	PJA2	praja ring finger ubiquitin ligase 2	Cytoplasm	enzyme		-1,702	3.04×10^-03
ENSG00000067225.17	PKM	pyruvate kinase M1/2	Cytoplasm	kinase	CAP-232	-1,355	3.54×10^-02
ENSG00000123143.12	PKN1	protein kinase N1	Cytoplasm	kinase		-2,104	9.34×10^-03
ENSG00000104368.17	PLAT	plasminogen activator, tissue type	Extracellular Space	peptidase	6-aminocaproic acid	-1,473	3.54×10^-02
ENSG00000151176.7	PLBD2	phospholipase B domain containing 2	Extracellular Space	other		1,322	1.68×10^-03
ENSG00000178209.14	PLEC	plectin	Cytoplasm	other		-1,91	4.69×10^-03
ENSG00000105559.11	PLEKHA4	pleckstrin homology domain containing A4	Cytoplasm	other		1,967	7.31×10^-04

ENSG00000183281.14	PLGLB1/PLGLB2	plasminogen like B2	Extracellular Space	peptidase		1,934	3.00×10^-02
ENSG00000083444.16	PLOD1	procollagen-lysine,2-oxoglutarate 5-dioxygenase 1	Cytoplasm	enzyme		-2,012	1.26×10^-02
ENSG00000152952.11	PLOD2	procollagen-lysine,2-oxoglutarate 5-dioxygenase 2	Cytoplasm	enzyme		-2,086	2.80×10^-03
ENSG00000102024.17	PLS3	plastin 3	Cytoplasm	other		-2,317	4.82×10^-05
ENSG00000004399.12	PLXND1	plexin D1	Plasma Membrane	transmembrane receptor		-2,562	2.52×10^-02
ENSG00000099817.11	POLR2E	RNA polymerase II, I and III subunit E	Nucleus	enzyme		-2,082	4.53×10^-03
ENSG00000105258.8	POLR2I	RNA polymerase II subunit I	Nucleus	transcription regulator		-1,731	2.04×10^-02
ENSG00000113356.10	POLR3G	RNA polymerase III subunit G	Nucleus	enzyme		-1,725	1.63×10^-02
ENSG00000221900.5	POM121L12	POM121 transmembrane nucleoporin like 12	Other	other		3,622	2.88×10^-06
ENSG00000125534.9	PPDPF	pancreatic progenitor cell differentiation and proliferation factor	Other	other		-1,595	1.14×10^-02
ENSG00000110841.13	PPFIBP1	PPFIA binding protein 1	Plasma Membrane	other		-1,397	1.67×10^-03
ENSG00000196262.13	PPIA	peptidylprolyl isomerase A	Cytoplasm	enzyme	cyclosporin A/methotrexate, basiliximab/cycl	-1,973	3.00×10^-04
ENSG00000166794.4	PPIB	peptidylprolyl isomerase B	Cytoplasm	enzyme		-3,151	9.87×10^-07
ENSG00000115241.10	PPM1G	protein phosphatase, Mg2+/Mn2+ dependent 1G	Nucleus	phosphatase		-1,29	2.76×10^-02
ENSG00000172531.14	PPP1CA	protein phosphatase 1 catalytic subunit alpha	Cytoplasm	phosphatase		-2,526	4.34×10^-03
ENSG00000257557.2	PPP1R12A-AS1	PPP1R12A antisense RNA 1	Other	other		1,316	2.21×10^-02
ENSG00000087074.7	PPP1R15A	protein phosphatase 1 regulatory subunit 15A	Cytoplasm	other		1,827	3.82×10^-05
ENSG00000100796.17	PPP4R3A	protein phosphatase 4 regulatory subunit 3A	Plasma Membrane	other		-1,069	4.47×10^-02
ENSG00000011485.14	PPP5C	protein phosphatase 5 catalytic subunit	Nucleus	phosphatase		-1,685	7.92×10^-03
ENSG00000117450.13	PRDX1	peroxiredoxin 1	Cytoplasm	enzyme		-2,105	6.42×10^-04
ENSG00000167815.11	PRDX2	peroxiredoxin 2	Cytoplasm	enzyme		-3,249	2.40×10^-02
ENSG00000123131.12	PRDX4	peroxiredoxin 4	Cytoplasm	enzyme		-2,959	6.57×10^-04
ENSG00000169230.9	PRELID1	PRELI domain containing 1	Cytoplasm	other		-1,808	6.24×10^-03
ENSG00000124126.13	PREX1	phosphatidylinositol-3,4,5-trisphosphate dependent Rac exchange factor 1	Cytoplasm	other		-3,184	1.25×10^-03
ENSG00000046889.18	PREX2	phosphatidylinositol-3,4,5-trisphosphate dependent Rac exchange factor 2	Cytoplasm	other		-2,216	5.72×10^-04
ENSG00000253729.7	PRKDC	protein kinase, DNA-activated, catalytic subunit	Nucleus	kinase	panulisib, CC-115, nedisertib, SM1-71, BR1(	-2,644	2.86×10^-06
ENSG00000185532.14	PRKG1	protein kinase cGMP-dependent 1	Cytoplasm	kinase		1,348	3.98×10^-02
ENSG00000171867.16	PRNP	prion protein	Plasma Membrane	other		-1,674	1.09×10^-02
ENSG00000101161.7	PRPF6	pre-mRNA processing factor 6	Nucleus	transcription regulator		-2,043	5.80×10^-03
ENSG00000174231.16	PRPF8	pre-mRNA processing factor 8	Nucleus	other		-1,213	3.63×10^-02
ENSG00000101911.12	PRPS2	phosphoribosyl pyrophosphate synthetase 2	Cytoplasm	kinase		-1,797	2.75×10^-02
ENSG00000183248.11	PRR36	proline rich 36	Other	other		1,363	4.06×10^-02
ENSG00000150687.11	PRSS23	serine protease 23	Extracellular Space	peptidase		-1,034	2.30×10^-02
ENSG00000122378.13	PRXL2A	peroxiredoxin like 2A	Extracellular Space	other		-1,296	1.36×10^-02
ENSG00000197746.13	PSAP	prosaposin	Extracellular Space	other		-2,579	1.10×10^-05
ENSG00000164985.14	PSIP1	PC4 and SFRS1 interacting protein 1	Nucleus	transcription regulator		-1,732	1.20×10^-03
ENSG00000106588.10	PSMA2	proteasome 20S subunit alpha 2	Cytoplasm	peptidase		-1,283	3.19×10^-02
ENSG00000101182.14	PSMA7	proteasome 20S subunit alpha 7	Cytoplasm	peptidase		-2,631	8.01×10^-05
ENSG00000008018.8	PSMB1	proteasome 20S subunit beta 1	Cytoplasm	peptidase	carfilzomib, bortezomib/cladribine/rituximab,	-1,882	3.21×10^-02
ENSG00000142507.9	PSMB6	proteasome 20S subunit beta 6	Nucleus	peptidase		-2,039	5.66×10^-04
ENSG00000100764.13	PSMC1	proteasome 26S subunit, ATPase 1	Nucleus	peptidase		-1,413	6.48×10^-03
ENSG00000165916.8	PSMC3	proteasome 26S subunit, ATPase 3	Nucleus	enzyme		-1,989	4.24×10^-03
ENSG00000131470.14	PSMC3IP	PSMC3 interacting protein	Nucleus	transcription regulator		-1,602	1.27×10^-02
ENSG00000173692.12	PSMD1	proteasome 26S subunit, non-ATPase 1	Cytoplasm	other	bortezomib/cladribine/rituximab, bortezomib/	-1,9	2.21×10^-03
ENSG00000185627.17	PSMD13	proteasome 26S subunit, non-ATPase 13	Cytoplasm	peptidase		-1,711	1.84×10^-02
ENSG00000103035.10	PSMD7	proteasome 26S subunit, non-ATPase 7	Cytoplasm	other		-2,503	7.97×10^-05
ENSG00000140043.11	PTGR2	prostaglandin reductase 2	Cytoplasm	enzyme	glycyrrhetinic acid	1,157	2.42×10^-02
ENSG00000169398.19	PTK2	protein tyrosine kinase 2	Cytoplasm	kinase	PF-573228, PF-431396, NVP-TAE 226, BI 8	-1,248	4.11×10^-02
ENSG00000187514.14	PTMA	prothymosin alpha	Nucleus	other		-1,735	7.22×10^-03
ENSG00000179295.15	PTPN11	protein tyrosine phosphatase non-receptor type 11	Cytoplasm	phosphatase	BBP-398, ERAS-601, PF-07284892, TNO15	-1,526	1.69×10^-03
ENSG00000225706.1	PTPRD-AS1	PTPRD antisense RNA 1	Other	other		1,89	1.85×10^-03
ENSG00000142949.16	PTPRF	protein tyrosine phosphatase receptor type F	Plasma Membrane	phosphatase		-1,568	1.48×10^-02
ENSG00000183255.11	PTTG1IP	PTTG1 interacting protein	Nucleus	other		-1,457	2.52×10^-02
ENSG00000163661.3	PTX3	pentraxin 3	Extracellular Space	other		-3,376	1.43×10^-03
ENSG00000134644.15	PUM1	pumilio RNA binding family member 1	Cytoplasm	other		-1,641	8.26×10^-03
ENSG00000136045.11	PWP1	PWP1 homolog, endonuclein	Nucleus	other		-1,713	4.39×10^-02
ENSG00000130508.10	PXDN	peroxidasin	Extracellular Space	enzyme		-2,115	1.53×10^-04
ENSG00000145337.4	PYURF	PIGY upstream reading frame	Cytoplasm	other		-2,514	2.92×10^-02
ENSG00000143545.8	RAB13	RAB13, member RAS oncogene family	Plasma Membrane	enzyme		-3,078	3.08×10^-07
ENSG00000104388.14	RAB2A	RAB2A, member RAS oncogene family	Cytoplasm	enzyme		-1,67	2.39×10^-02
ENSG00000152932.7	RAB3C	RAB3C, member RAS oncogene family	Cytoplasm	enzyme		1,093	1.78×10^-02
ENSG00000075785.12	RAB7A	RAB7A, member RAS oncogene family	Cytoplasm	enzyme		-1,529	9.11×10^-03
ENSG00000136238.17	RAC1	Rac family small GTPase 1	Plasma Membrane	enzyme		-1,912	1.32×10^-03
ENSG00000204628.11	RACK1	receptor for activated C kinase 1	Cytoplasm	enzyme		-1,987	3.70×10^-04
ENSG00000164754.12	RAD21	RAD21 cohesin complex component	Nucleus	transcription regulator		-1,452	8.26×10^-03
ENSG00000039560.13	RAI14	retinoic acid induced 14	Nucleus	transcription regulator		-1,942	4.14×10^-05
ENSG00000006451.7	RALA	RAS like proto-oncogene A	Cytoplasm	enzyme		-2,536	9.69×10^-06
ENSG00000132341.11	RAN	RAN, member RAS oncogene family	Nucleus	enzyme		-1,127	3.00×10^-02
ENSG00000099901.16	RANBP1	RAN binding protein 1	Nucleus	other		-1,434	2.23×10^-02
ENSG00000153201.15	RANBP2	RAN binding protein 2	Nucleus	enzyme		-1,032	4.13×10^-02
ENSG00000100401.19	RANGAP1	Ran GTPase activating protein 1	Nucleus	other		-1,592	1.98×10^-02
ENSG00000107263.18	RAPGEF1	Rap guanine nucleotide exchange factor 1	Cytoplasm	other		-1,803	7.88×10^-03
ENSG00000145715.14	RASA1	RAS p21 protein activator 1	Cytoplasm	transporter		-1,703	4.03×10^-02
ENSG00000185989.10	RASA3	RAS p21 protein activator 3	Plasma Membrane	ion channel		-1,998	2.90×10^-02
ENSG00000108551.4	RASD1	ras related dexamethasone induced 1	Cytoplasm	enzyme		5,218	1.08×10^-20
ENSG00000153179.11	RASSF3	Ras association domain family member 3	Other	other		-2,932	3.98×10^-08
ENSG00000122965.10	RBM19	RNA binding motif protein 19	Nucleus	other		1,259	3.96×10^-02
ENSG00000049449.8	RCN1	reticulocalbin 1	Cytoplasm	other		-2,46	7.06×10^-06
ENSG00000137710.14	RDX	radixin	Cytoplasm	other		-1,751	1.54×10^-03
ENSG00000181826.9	RELL1	RELT like 1	Plasma Membrane	other		-3,578	1.86×10^-02
ENSG00000148300.11	REXO4	REX4 homolog, 3'-5' exonuclease	Nucleus	transcription regulator		-1,901	2.52×10^-02
ENSG00000143390.17	RFX5	regulatory factor X5	Nucleus	transcription regulator		1,41	3.50×10^-03
ENSG00000183054.11	RGPD4 (includes others)	RANBP2 like and GRIP domain containing 5	Nucleus	enzyme		-1,483	5.00×10^-03
ENSG00000138835.22	RGS3	regulator of G protein signaling 3	Nucleus	enzyme		-1,432	9.34×10^-03
ENSG00000143248.12	RGS5	regulator of G protein signaling 5	Plasma Membrane	enzyme		-1,261	1.59×10^-02
ENSG00000067560.10	RHOA	ras homolog family member A	Cytoplasm	enzyme		-2,643	4.15×10^-06
ENSG00000143878.9	RHOB	ras homolog family member B	Cytoplasm	enzyme		1,434	9.25×10^-03
ENSG00000126785.12	RHOJ	ras homolog family member J	Cytoplasm	enzyme		-1,181	4.38×10^-02
ENSG00000269900.3	RMRP	RNA component of mitochondrial RNA processing endoribonuclease	Cytoplasm	other		1,937	4.01×10^-02
ENSG00000263740.2	RN7SL4P	RNA, 7SL, cytoplasmic 4, pseudogene	Other	other		2,116	9.86×10^-03
ENSG00000265735.2	RN7SL5P	RNA, 7SL, cytoplasmic 5, pseudogene	Other	other		1,788	1.80×10^-03
ENSG00000239437.3	RN7SL752P		Other	other		3,48	1.28×10^-03
ENSG00000275757.1	RNA5-8SN1	RNA, 5.8S ribosomal N1	Other	other		11,432	2.57×10^-09
ENSG00000163961.4	RNF168	ring finger protein 168	Nucleus	enzyme		-1,212	2.90×10^-02
ENSG00000155827.11	RNF20	ring finger protein 20	Nucleus	enzyme		-1,497	4.69×10^-03
ENSG00000196204.11	RNF216P1	ring finger protein 216 pseudogene 1	Other	other		-1,52	2.07×10^-02
ENSG00000200340.1	RNU1-105P	RNA, U1 small nuclear 105, pseudogene	Other	other		5,285	2.18×10^-07
ENSG00000199805.1	RNU1-134P	RNA, U1 small nuclear 134, pseudogene	Other	other		3,741	1.41×10^-02
ENSG00000206908.1	RNU1-136P	RNA, U1 small nuclear 136, pseudogene	Other	other		4,905	2.51×10^-05
ENSG00000212609.1	RNU1-139P		Other	other		3,891	1.23×10^-02
ENSG00000201910.1	RNU1-140P		Other	other		5,389	2.47×10^-03
ENSG00000200885.1	RNU1-146P	RNA, U1 small nuclear 146, pseudogene	Other	other		6,934	6.50×10^-07
ENSG00000199629.1	RNU1-14P	RNA, U1 small nuclear 14, pseudogene	Other	other		4,597	9.79×10^-05
ENSG00000202347.1	RNU1-16P	RNA, U1 small nuclear 16, pseudogene	Other	other		4,205	1.43×10^-03
ENSG00000200197.1	RNU1-21P	RNA, U1 small nuclear 21, pseudogene	Other	other		4,532	6.50×10^-04
ENSG00000206596.1	RNU1-27P	RNA, U1 small nuclear 27, pseudogene	Other	other		2,125	3.24×10^-02
ENSG00000200807.1	RNU1-32P	RNA, U1 small nuclear 32, pseudogene	Other	other		5	1.22×10^-03
ENSG00000206624.1	RNU1-39P	RNA, U1 small nuclear 39, pseudogene	Other	other		3,925	2.01×10^-02
ENSG00000212170.1	RNU1-77P		Other	other		4,349	6.07×10^-09
ENSG00000207322.1	RNU1-89P	RNA, U1 small nuclear 89, pseudogene	Other	other		3,322	2.76×10^-05
ENSG00000239023.1	RNU1-98P		Other	other		3,871	5.20×10^-03
ENSG00000270103.3	RNU11		Other	other		3,959	3.42×10^-06
ENSG00000276027.1	RNU12	RNA, U12 small nuclear	Nucleus	other		4,192	1.77×10^-04
ENSG00000222328.1	RNU2-2P	RNA, U2 small nuclear 2, pseudogene	Nucleus	other		2,443	1.44×10^-05
ENSG00000222627.1	RNU2-37P	RNA, U2 small nuclear 37, pseudogene	Other	other		4,989	3.77×10^-06
ENSG00000222426.1	RNU2-50P	RNA, U2 small nuclear 50, pseudogene	Other	other		4,594	7.48×10^-04
ENSG00000222810.1	RNU2-68P		Other	other		5,065	7.15×10^-05
ENSG00000200795.1	RNU4-1	RNA, U4 small nuclear 1	Nucleus	other		3,507	8.70×10^-12
ENSG00000202538.1	RNU4-2	RNA, U4 small nuclear 2	Other	other		3,939	2.83×10^-13
ENSG00000199568.1	RNU5A-1	RNA, U5A small nuclear 1	Other	other		4,295	2.74×10^-04
ENSG00000200972.1	RNU5A-8P	RNA, U5A small nuclear 8, pseudogene	Other	other		4,657	5.48×10^-05
ENSG00000200156.1	RNU5B-1	RNA, U5B small nuclear 1	Other	other		3,347	8.30×10^-04
ENSG00000199347.1	RNU5E-1	RNA, U5E small nuclear 1	Other	other		4,553	1.05×10^-02
ENSG00000202444.1	RNU5E-6P	RNA, U5E small nuclear 6, pseudogene	Other	other		5,705	1.60×10^-10
ENSG00000199377.1	RNU5F-1	RNA, U5F small nuclear 1	Other	other		3,571	1.67×10^-03
ENSG00000207340.1	RNVU1-1	RNA, variant U1 small nuclear 1	Other	other		5,971	2.68×10^-05
ENSG00000275538.1	RNVU1-19	RNA, variant U1 small nuclear 19	Other	other		6,358	7.76×10^-05
ENSG00000199879.1	RNVU1-22		Other	other		6,038	1.54×10^-13
ENSG00000277918.1	RNVU1-28		Other	other		3,271	1.66×10^-05
ENSG00000273768.1	RNVU1-29		Other	other		2,116	3.24×10^-02
ENSG00000278099.1	RNVU1-2A	RNA, variant U1 small nuclear 2A	Other	other		7,182	2.23×10^-15
ENSG00000270722.1	RNVU1-31	RNA, variant U1 small nuclear 31	Other	other		3,201	1.14×10^-06
ENSG00000207110.1	RNVU1-32	RNA, variant U1 small nuclear 32	Other	other		5,545	4.08×10^-07
ENSG00000154133.14	ROBO4	roundabout guidance receptor 4	Plasma Membrane	other		-2,052	7.65×10^-04
ENSG00000273175.1	RP11_11N74		Other	other		1,791	4.96×10^-02
ENSG00000264207.1	RP11_196G1823		Other	other		1,307	4.30×10^-02
ENSG00000258010.3	RP11_210M151		Other	other		1,83	6.84×10^-04
ENSG00000279762.3	RP11_227G1512		Other	other		1,135	2.78×10^-02
ENSG00000279286.2	RP11_248B241		Other	other		1,241	1.73×10^-02
ENSG00000249731.1	RP11_259O23		Other	other		1,428	1.08×10^-02
ENSG00000279065.1	RP11_264J410		Other	other		2,376	1.38×10^-03
ENSG00000243635.1	RP11_281P111		Other	other		4,796	1.60×10^-08
ENSG00000279070.2	RP11_287D12		Other	other		1,84	4.89×10^-02
ENSG00000233367.1	RP11_307L34		Other	other		2,683	9.84×10^-03
ENSG00000279569.1	RP11_394B21		Other	other		1,994	7.45×10^-03
ENSG00000265916.1	RP11_434D29		Other	other		2,45	1.50×10^-02
ENSG00000275740.1	RP11_449H33		Other	other		-5,188	1.32×10^-02
ENSG00000276900.1	RP11_467L137		Other	other		1,947	3.91×10^-02
ENSG00000278576.1	RP11_507K26		Other	other		1,243	8.72×10^-03
ENSG00000275216.1	RP11_54H74		Other	other		1,464	1.62×10^-03
ENSG00000257884.2	RP11_597A114		Other	other		-1,241	3.80×10^-02
ENSG00000267546.2	RP11_666A88		Other	other		2,1	1.70×10^-02
ENSG00000259135.1	RP11_671J114		Other	other		4,697	2.36×10^-05
ENSG00000265043.1	RP11_728E143		Other	other		1,248	3.83×10^-02
ENSG00000273702.1	RP11_758H92		Other	other		2,129	9.99×10^-03
ENSG00000275880.1	RP11_90L18		Other	other		2,416	7.47×10^-03
ENSG00000279479.1	RP11_91I82		Other	other		2,035	1.14×10^-02
ENSG00000273367.1	RP5_827C216		Other	other		2,492	8.37×10^-03
ENSG00000279419.1	RP5_907C103		Other	other		1,307	7.21×10^-03
ENSG00000198755.10	RPL10A	ribosomal protein L10a	Nucleus	other		-1,316	4.16×10^-02
ENSG00000142676.12	RPL11	ribosomal protein L11	Cytoplasm	other		-2,286	1.81×10^-05

ENSG00000197958.12	RPL12	ribosomal protein L12	Nucleus	other		-1,415	3.63×10^-02
ENSG00000142541.16	RPL13A	ribosomal protein L13a	Cytoplasm	other		-1,58	2.74×10^-02
ENSG00000188846.13	RPL14	ribosomal protein L14	Cytoplasm	other		-1,924	1.46×10^-04
ENSG00000174748.18	RPL15	ribosomal protein L15	Cytoplasm	other		-1,563	4.19×10^-03
ENSG00000265681.6	RPL17	ribosomal protein L17	Cytoplasm	other		-1,405	1.56×10^-02
ENSG00000063177.12	RPL18	ribosomal protein L18	Cytoplasm	other		-1,572	9.34×10^-03
ENSG00000105640.12	RPL18A	ribosomal protein L18a	Cytoplasm	other		-1,979	4.02×10^-04
ENSG00000108298.9	RPL19	ribosomal protein L19	Cytoplasm	other		-1,886	2.46×10^-03
ENSG00000122026.10	RPL21	ribosomal protein L21	Cytoplasm	other		-2,138	2.12×10^-04
ENSG00000116251.9	RPL22	ribosomal protein L22	Cytoplasm	translation regulator		-2,006	1.40×10^-04
ENSG00000125691.12	RPL23	ribosomal protein L23	Cytoplasm	other		-1,539	4.36×10^-03
ENSG00000198242.13	RPL23A	ribosomal protein L23a	Cytoplasm	other		-2,176	2.50×10^-04
ENSG00000114391.12	RPL24	ribosomal protein L24	Cytoplasm	other		-1,536	3.69×10^-03
ENSG00000161970.12	RPL26	ribosomal protein L26	Cytoplasm	other		-1,803	1.21×10^-03
ENSG00000131469.12	RPL27	ribosomal protein L27	Cytoplasm	other		-2,019	2.50×10^-04
ENSG00000162244.10	RPL29	ribosomal protein L29	Cytoplasm	other		-2,055	1.57×10^-04
ENSG00000100316.15	RPL3	ribosomal protein L3	Nucleus	other	cytarabine/daunorubicin/omacetaxine mepe	-1,677	3.91×10^-03
ENSG00000144713.12	RPL32	ribosomal protein L32	Cytoplasm	other		-1,519	2.21×10^-02
ENSG00000109475.16	RPL34	ribosomal protein L34	Cytoplasm	other		-1,472	7.22×10^-03
ENSG00000182899.14	RPL35A	ribosomal protein L35a	Cytoplasm	other		-1,574	1.17×10^-02
ENSG00000241343.9	RPL36A	ribosomal protein L36a	Cytoplasm	other		-1,808	1.41×10^-03
ENSG00000165502.6	RPL36AL	ribosomal protein L36a like	Cytoplasm	other		-2,373	3.27×10^-05
ENSG00000145592.13	RPL37	ribosomal protein L37	Cytoplasm	other		-1,252	4.94×10^-02
ENSG00000198918.7	RPL39	ribosomal protein L39	Cytoplasm	other		-1,994	1.19×10^-04
ENSG00000174444.14	RPL4	ribosomal protein L4	Cytoplasm	enzyme		-1,705	3.88×10^-03
ENSG00000122406.12	RPL5	ribosomal protein L5	Cytoplasm	other		-2,453	7.34×10^-06
ENSG00000089009.15	RPL6	ribosomal protein L6	Nucleus	other		-1,913	7.65×10^-04
ENSG00000147604.13	RPL7	ribosomal protein L7	Nucleus	transcription regulator		-1,726	2.97×10^-03
ENSG00000148303.16	RPL7A	ribosomal protein L7a	Cytoplasm	other		-1,349	4.96×10^-02
ENSG00000161016.15	RPL8	ribosomal protein L8	Cytoplasm	other		-1,673	4.50×10^-03
ENSG00000163682.15	RPL9	ribosomal protein L9	Nucleus	other		-1,591	2.02×10^-03
ENSG00000089157.15	RPLP0	ribosomal protein lateral stalk subunit P0	Cytoplasm	other		-1,712	6.90×10^-03
ENSG00000137818.11	RPLP1	ribosomal protein lateral stalk subunit P1	Cytoplasm	other		-1,755	2.34×10^-03
ENSG00000177600.8	RPLP2	ribosomal protein lateral stalk subunit P2	Cytoplasm	other		-1,562	1.41×10^-02
ENSG00000163902.11	RPN1	ribophorin I	Cytoplasm	enzyme		-2,559	4.47×10^-05
ENSG00000118705.16	RPN2	ribophorin II	Cytoplasm	enzyme		-3,754	2.63×10^-03
ENSG00000124614.13	RPS10	ribosomal protein S10	Cytoplasm	other		-1,543	1.63×10^-02
ENSG00000142534.6	RPS11	ribosomal protein S11	Cytoplasm	other		-1,49	8.29×10^-03
ENSG00000112306.7	RPS12	ribosomal protein S12	Cytoplasm	other	neomycin	-1,933	5.09×10^-04
ENSG00000134419.15	RPS15A	ribosomal protein S15a	Cytoplasm	other		-1,329	1.06×10^-02
ENSG00000105193.8	RPS16	ribosomal protein S16	Cytoplasm	other		-1,747	4.36×10^-03
ENSG00000182774.10	RPS17	ribosomal protein S17	Cytoplasm	other		-1,311	1.79×10^-02
ENSG00000231500.6	RPS18	ribosomal protein S18	Cytoplasm	other		-2,462	3.99×10^-05
ENSG00000008988.9	RPS20	ribosomal protein S20	Cytoplasm	other		-1,825	7.06×10^-04
ENSG00000186468.12	RPS23	ribosomal protein S23	Cytoplasm	translation regulator		-2,246	5.61×10^-05
ENSG00000138326.18	RPS24	ribosomal protein S24	Cytoplasm	other		-1,511	1.02×10^-02
ENSG00000197728.9	RPS26	ribosomal protein S26	Cytoplasm	other		-1,538	6.83×10^-03
ENSG00000143947.12	RPS27A	ribosomal protein S27a	Cytoplasm	other		-1,973	1.40×10^-04
ENSG00000240342.3	RPS2P5	ribosomal protein S2 pseudogene 5	Other	other		-2,611	4.45×10^-04
ENSG00000149273.14	RPS3	ribosomal protein S3	Cytoplasm	enzyme		-2,125	8.01×10^-05
ENSG00000145425.9	RPS3A	ribosomal protein S3A	Nucleus	other		-2,213	2.35×10^-05
ENSG00000234797.5	RPS3AP6	RPS3A pseudogene 6	Other	other		-3,017	9.84×10^-06
ENSG00000198034.10	RPS4X	ribosomal protein S4 X-linked	Cytoplasm	other		-1,867	7.74×10^-04
ENSG00000083845.8	RPS5	ribosomal protein S5	Cytoplasm	other		-1,43	3.18×10^-02
ENSG00000137154.12	RPS6	ribosomal protein S6	Cytoplasm	other		-2,452	5.21×10^-06
ENSG00000071242.11	RPS6KA2	ribosomal protein S6 kinase A2	Nucleus	kinase	PMD-026, LJH685	-1,592	2.57×10^-02
ENSG00000142937.11	RPS8	ribosomal protein S8	Cytoplasm	other		-1,589	6.00×10^-03
ENSG00000168028.13	RPSA	ribosomal protein SA	Cytoplasm	translation regulator		-2,236	5.88×10^-05
ENSG00000167325.14	RRM1	ribonucleotide reductase catalytic subunit M1	Nucleus	enzyme	L-asparaginase/gemcitabine/oxaliplatin, aler	-2,259	4.19×10^-03
ENSG00000189306.10	RRP7A	ribosomal RNA processing 7 homolog A	Cytoplasm	other		-2,619	4.34×10^-03
ENSG00000169402.15	RSPH10B/RSPH10B2	radial spoke head 10 homolog B	Extracellular Space	other		2,28	1.56×10^-02
ENSG00000197747.8	S100A10	S100 calcium binding protein A10	Cytoplasm	other		-1,907	4.22×10^-04
ENSG00000163191.5	S100A11	S100 calcium binding protein A11	Cytoplasm	other		-1,532	2.10×10^-02
ENSG00000188643.10	S100A16	S100 calcium binding protein A16	Nucleus	other		-2,764	4.36×10^-03
ENSG00000170989.8	S1PR1	sphingosine-1-phosphate receptor 1	Plasma Membrane	G-protein coupled receptor	fingolimod phosphate, siponimod, ponesimo	-2,513	2.85×10^-03
ENSG00000151835.13	SACS	sacsin molecular chaperone	Plasma Membrane	other		-1,158	1.07×10^-02
ENSG00000142230.11	SAE1	SUMO1 activating enzyme subunit 1	Cytoplasm	enzyme		-1,609	3.60×10^-02
ENSG00000160633.12	SAFB	scaffold attachment factor B	Nucleus	other		-1,684	1.37×10^-03
ENSG00000138760.8	SCARB2	scavenger receptor class B member 2	Plasma Membrane	transmembrane receptor		-1,867	7.36×10^-04
ENSG00000252481.1	SCARNA13	small Cajal body-specific RNA 13	Nucleus	other		1,725	6.56×10^-03
ENSG00000280466.1	SCARNA4	small Cajal body-specific RNA 4	Other	other		3,407	5.67×10^-04
ENSG00000099194.5	SCD	stearoyl-CoA desaturase	Cytoplasm	enzyme		-1,813	4.18×10^-04
ENSG00000136193.16	SCRN1	secernin 1	Cytoplasm	other		-1,277	1.63×10^-02
ENSG00000157020.17	SEC13	SEC13 homolog, nuclear pore and COPII coat complex component	Cytoplasm	transporter		-1,461	1.66×10^-02
ENSG00000129657.14	SEC14L1	SEC14 like lipid binding 1	Cytoplasm	transporter		-1,646	3.14×10^-04
ENSG00000148396.18	SEC16A	SEC16 homolog A, endoplasmic reticulum export factor	Cytoplasm	other		-1,503	8.70×10^-03
ENSG00000121542.11	SEC22A	SEC22 homolog A, vesicle trafficking protein	Cytoplasm	transporter		1,849	7.61×10^-03
ENSG00000176986.14	SEC24C	SEC24 homolog C, COPII coat complex component	Cytoplasm	transporter		-2,109	1.83×10^-02
ENSG00000138674.16	SEC31A	SEC31 homolog A, COPII coat complex component	Cytoplasm	other		-2,43	1.61×10^-05
ENSG00000058262.9	SEC61A1	SEC61 translocon subunit alpha 1	Cytoplasm	transporter		-1,922	1.68×10^-03
ENSG00000132432.13	SEC61G	SEC61 translocon subunit gamma	Plasma Membrane	transporter		-2,776	7.05×10^-03
ENSG00000008952.16	SEC62	SEC62 homolog, preprotein translocation factor	Cytoplasm	transporter		-1,013	4.77×10^-02
ENSG00000162430.16	SELENON	selenoprotein N	Cytoplasm	other		-2,484	1.94×10^-03
ENSG00000250722.5	SELENOP	selenoprotein P	Extracellular Space	other		1,324	2.05×10^-02
ENSG00000178980.14	SELENOW	selenoprotein W	Cytoplasm	enzyme		-1,444	3.91×10^-02
ENSG00000170381.12	SEMA3E	semaphorin 3E	Extracellular Space	other		1,066	2.33×10^-02
ENSG00000112701.17	SENP6	SUMO specific peptidase 6	Cytoplasm	peptidase		-1,3	2.99×10^-02
ENSG00000229000.1	SEPTIN7P8	septin 7 pseudogene 8	Other	other		2,444	5.57×10^-03
ENSG00000142864.14	SERBP1	SERPINE1 mRNA binding protein 1	Cytoplasm	other		-1,405	1.41×10^-02
ENSG00000124570.17	SERPINB6	serpin family B member 6	Cytoplasm	other		-1,386	4.30×10^-02
ENSG00000106366.8	SERPINE1	serpin family E member 1	Extracellular Space	other	TM5614, drotrecogin alfa	-3,852	1.24×10^-03
ENSG00000149257.13	SERPINH1	serpin family H member 1	Extracellular Space	other		-1,547	1.27×10^-02
ENSG00000119335.16	SET	SET nuclear proto-oncogene	Nucleus	phosphatase		-1,57	1.27×10^-02
ENSG00000168137.15	SETD5	SET domain containing 5	Nucleus	enzyme		-1,536	4.46×10^-03
ENSG00000099995.18	SF3A1	splicing factor 3a subunit 1	Nucleus	other		-2,184	6.56×10^-04
ENSG00000183431.11	SF3A3	splicing factor 3a subunit 3	Nucleus	other		-1,853	3.72×10^-03
ENSG00000115524.15	SF3B1	splicing factor 3b subunit 1	Nucleus	other	H3B-8800	-1,664	2.08×10^-04
ENSG00000087365.14	SF3B2	splicing factor 3b subunit 2	Nucleus	other		-1,699	1.12×10^-03
ENSG00000189091.12	SF3B3	splicing factor 3b subunit 3	Nucleus	other		-1,539	2.57×10^-03
ENSG00000111252.10	SH2B3	SH2B adaptor protein 3	Plasma Membrane	other		-1,493	3.68×10^-03
ENSG00000095370.19	SH2D3C	SH2 domain containing 3C	Cytoplasm	other		-3,46	7.47×10^-03
ENSG00000125089.16	SH3TC1	SH3 domain and tetratricopeptide repeats 1	Extracellular Space	other		-2,273	2.34×10^-02
ENSG00000158352.15	SHROOM4	shroom family member 4	Plasma Membrane	other		-1,7	2.60×10^-04
ENSG00000138083.4	SIX3	SIX homeobox 3	Nucleus	transcription regulator		1,125	3.55×10^-02
ENSG00000124140.12	SLC12A5	solute carrier family 12 member 5	Plasma Membrane	transporter	bumetanide	1,392	5.96×10^-03
ENSG00000125434.10	SLC25A35	solute carrier family 25 member 35	Cytoplasm	other		1,449	1.41×10^-02
ENSG00000005022.5	SLC25A5	solute carrier family 25 member 5	Cytoplasm	transporter	clodronic acid	-1,771	4.89×10^-03
ENSG00000112759.16	SLC29A1	solute carrier family 29 member 1 (Augustine blood group)	Plasma Membrane	transporter	cannabidiol	-3,115	2.82×10^-02
ENSG00000157637.12	SLC38A10	solute carrier family 38 member 10	Cytoplasm	other		-1,331	4.99×10^-02
ENSG00000147804.9	SLC39A4	solute carrier family 39 member 4	Plasma Membrane	transporter		1,307	2.80×10^-02
ENSG00000011083.8	SLC6A7	solute carrier family 6 member 7	Plasma Membrane	transporter		1,394	4.04×10^-02
ENSG00000260727.1	SLC7A5P1	solute carrier family 7 member 5 pseudogene 1	Other	other		5,38	2.34×10^-04
ENSG00000258186.2	SLC7A5P2	solute carrier family 7 member 5 pseudogene 2	Other	other		4,946	2.61×10^-06
ENSG00000065613.13	SLK	STE20 like kinase	Nucleus	kinase		-1,509	1.20×10^-03
ENSG00000137776.16	SLTM	SAFB like transcription modulator	Nucleus	other		-1,172	1.21×10^-02
ENSG00000080503.19	SMARCA2	SWI/SNF related, matrix associated, actin dependent regulator of chromatin, subfamily a, member 2	Nucleus	transcription regulator		-1,802	7.46×10^-03
ENSG00000153147.5	SMARCA5	SWI/SNF related, matrix associated, actin dependent regulator of chromatin, subfamily a, member 5	Nucleus	transcription regulator		-1,128	3.47×10^-02
ENSG00000099956.17	SMARCB1	SWI/SNF related, matrix associated, actin dependent regulator of chromatin, subfamily b, member 1	Nucleus	transcription regulator		-2,439	7.76×10^-03
ENSG00000173473.10	SMARCC1	SWI/SNF related, matrix associated, actin dependent regulator of chromatin subfamily c member 1	Nucleus	transcription regulator		-1,637	4.60×10^-03
ENSG00000072501.17	SMC1A	structural maintenance of chromosomes 1A	Nucleus	transporter		-1,779	1.36×10^-03
ENSG00000113810.15	SMC4	structural maintenance of chromosomes 4	Nucleus	transporter		-1,237	1.30×10^-02
ENSG00000180747.15	SMG1P3	SMG1 pseudogene 3	Other	other		-1,322	4.93×10^-02
ENSG00000102172.15	SMS	spermine synthase	Cytoplasm	enzyme	(±)-2-hydroxyoleic acid	-2,525	7.01×10^-05
ENSG00000247775.2	SNCA-AS1	SNCA antisense RNA 1	Other	other		4,684	5.66×10^-05
ENSG00000242125.3	SNHG3	small nucleolar RNA host gene 3	Nucleus	other		1,416	1.13×10^-02
ENSG00000245910.8	SNHG6	small nucleolar RNA host gene 6	Other	other		-2,191	2.01×10^-02
ENSG00000212464.1	SNORA12	small nucleolar RNA, H/ACA box 12	Other	other		3,033	7.17×10^-04
ENSG00000280498.1	SNORA16A	small nucleolar RNA, H/ACA box 16A	Other	other		8,005	9.10×10^-03
ENSG00000280496.1	SNORA17B	small nucleolar RNA, H/ACA box 17B	Other	other		2,493	1.88×10^-04
ENSG00000207145.1	SNORA18	small nucleolar RNA, H/ACA box 18	Other	other		2,842	6.11×10^-04
ENSG00000273544.1	SNORA44	small nucleolar RNA, H/ACA box 44	Other	other		6,113	5.62×10^-05
ENSG00000212443.1	SNORA53	small nucleolar RNA, H/ACA box 53	Other	other		1,948	3.58×10^-02
ENSG00000278274.1	SNORA61	small nucleolar RNA, H/ACA box 61	Other	other		4,732	1.23×10^-05
ENSG00000274266.1	SNORA73A	small nucleolar RNA, H/ACA box 73A	Nucleus	other		4,722	3.07×10^-03
ENSG00000200087.1	SNORA73B	small nucleolar RNA, H/ACA box 73B	Other	other		4,768	9.12×10^-06
ENSG00000163788.13	SNRK	SNF related kinase	Cytoplasm	kinase		-1,909	4.01×10^-03
ENSG00000125835.17	SNRPB	small nuclear ribonucleoprotein polypeptides B and B1	Nucleus	other		-2,45	1.45×10^-04
ENSG00000125870.10	SNRPB2	small nuclear ribonucleoprotein polypeptide B2	Nucleus	other		-2,283	3.10×10^-03
ENSG00000124562.9	SNRPC	small nuclear ribonucleoprotein polypeptide C	Nucleus	other		-2,442	8.76×10^-04
ENSG00000167088.10	SNRPD1	small nuclear ribonucleoprotein D1 polypeptide	Nucleus	other		-1,64	1.68×10^-03
ENSG00000100028.11	SNRPD3	small nuclear ribonucleoprotein D3 polypeptide	Nucleus	other		-1,722	2.00×10^-02
ENSG00000139343.10	SNRPF	small nuclear ribonucleoprotein polypeptide F	Nucleus	other		-1,156	2.07×10^-02
ENSG00000143977.13	SNRPG	small nuclear ribonucleoprotein polypeptide G	Nucleus	other		-2,059	1.22×10^-02
ENSG00000128739.20	SNRPN	small nuclear ribonucleoprotein polypeptide N	Nucleus	other		-3,082	3.07×10^-02
ENSG00000100603.13	SNW1	SNW domain containing 1	Nucleus	transcription regulator		-1,667	7.39×10^-03
ENSG00000205302.6	SNX2	sorting nexin 2	Cytoplasm	transporter		-2,078	2.74×10^-02
ENSG00000112335.14	SNX3	sorting nexin 3	Cytoplasm	transporter		-2,565	1.65×10^-03
ENSG00000129515.18	SNX6	sorting nexin 6	Cytoplasm	transporter		-1,337	8.52×10^-03
ENSG00000184557.4	SOCS3	suppressor of cytokine signaling 3	Cytoplasm	phosphatase		1,527	6.38×10^-04
ENSG00000142168.14	SOD1	superoxide dismutase 1	Cytoplasm	enzyme		-2,814	2.63×10^-03
ENSG00000112096.16	SOD2	superoxide dismutase 2	Cytoplasm	enzyme	manganese	1,136	1.10×10^-02
ENSG00000159140.17	SON	SON DNA and RNA binding protein	Nucleus	other		-1,823	2.34×10^-05
ENSG00000125398.5	SOX9	SRY-box transcription factor 9	Nucleus	transcription regulator		2,434	3.29×10^-08
ENSG00000113140.10	SPARC	secreted protein acidic and cysteine rich	Extracellular Space	other		-3,315	5.79×10^-10
ENSG00000197471.11	SPN	sialophorin	Plasma Membrane	transmembrane receptor		1,15	2.93×10^-02
ENSG00000171621.13	SPSB1	splA/ryanodine receptor domain and SOCS box containing 1	Cytoplasm	other		1,496	1.48×10^-02
ENSG00000197694.13	SPTAN1	spectrin alpha, non-erythrocytic 1	Plasma Membrane	other		-1,582	3.68×10^-03
ENSG00000115306.15	SPTBN1	spectrin beta, non-erythrocytic 1	Plasma Membrane	other		-2,847	2.55×10^-06
ENSG00000198911.11	SREBF2	sterol regulatory element binding transcription factor 2	Nucleus	transcription regulator		-1,655	4.54×10^-02
ENSG00000140319.10	SRP14	signal recognition particle 14	Cytoplasm	other		-2,204	2.62×10^-05
ENSG00000143742.12	SRP9	signal recognition particle 9	Cytoplasm	other		-2,252	5.85×10^-04

ENSG00000182934.11	SRPRA	SRP receptor subunit alpha	Cytoplasm	other		-2,098	3.75×10^-03
ENSG00000101955.14	SRPX	sushi repeat containing protein X-linked	Cytoplasm	other		-2,757	4.80×10^-03
ENSG00000114850.6	SSR3	signal sequence receptor subunit 3	Cytoplasm	other		-2,115	1.15×10^-03
ENSG00000235374.2	SSR4P1	signal sequence receptor subunit 4 pseudogene 1	Other	other		2,434	2.74×10^-02
ENSG00000149136.7	SSRP1	structure specific recognition protein 1	Nucleus	transcription regulator		-1,451	1.22×10^-02
ENSG00000100380.12	ST13	ST13 Hsp70 interacting protein	Cytoplasm	other		-1,872	2.70×10^-04
ENSG00000010327.10	STAB1	stabilin 1	Plasma Membrane	transporter	FP-1305	-2,016	1.71×10^-02
ENSG00000101972.18	STAG2	stromal antigen 2	Nucleus	other		-1,646	8.02×10^-03
ENSG00000084090.13	STARD7	StAR related lipid transfer domain containing 7	Cytoplasm	other		-1,187	4.71×10^-02
ENSG00000117632.20	STMN1	stathmin 1	Cytoplasm	other		-1,805	4.08×10^-04
ENSG00000134910.12	STT3A	STT3 oligosaccharyltransferase complex catalytic subunit A	Plasma Membrane	enzyme		-1,344	2.92×10^-02
ENSG00000163527.9	STT3B	STT3 oligosaccharyltransferase complex catalytic subunit B	Cytoplasm	enzyme		-2,662	1.33×10^-02
ENSG00000173597.8	SULT1B1	sulfotransferase family 1B member 1	Cytoplasm	enzyme		-1,363	4.90×10^-02
ENSG00000116030.16	SUMO1	small ubiquitin like modifier 1	Nucleus	enzyme		-1,44	3.17×10^-02
ENSG00000188612.11	SUMO2	small ubiquitin like modifier 2	Nucleus	enzyme		-1,652	4.74×10^-03
ENSG00000092201.9	SUPT16H	SPT16 homolog, facilitates chromatin remodeling subunit	Nucleus	transcription regulator		-1,126	2.11×10^-02
ENSG00000109111.14	SUPT6H	SPT6 homolog, histone chaperone and transcription elongation factor	Nucleus	transcription regulator		-1,741	4.85×10^-04
ENSG00000148248.13	SURF4	surfeit 4	Cytoplasm	other		-2,268	1.62×10^-03
ENSG00000133789.14	SWAP70	switching B cell complex subunit SWAP70	Cytoplasm	other		-2,017	1.53×10^-04
ENSG00000135316.17	SYNCRIP	synaptotagmin binding cytoplasmic RNA interacting protein	Nucleus	other	supinoxin	-1,598	3.41×10^-03
ENSG00000055070.16	SZRD1	SUZ RNA binding domain containing 1	Other	other		-1,538	3.63×10^-02
ENSG00000147526.19	TACC1	transforming acidic coiled-coil containing protein 1	Nucleus	other		-1,746	3.99×10^-05
ENSG00000270647.5	TAF15	TATA-box binding protein associated factor 15	Nucleus	other		-2,395	1.45×10^-05
ENSG00000158710.14	TAGLN2	transgelin 2	Cytoplasm	other		-1,433	1.28×10^-02
ENSG00000177156.10	TALDO1	transaldolase 1	Cytoplasm	enzyme		-1,593	1.49×10^-02
ENSG00000136111.12	TBC1D4	TBC1 domain family member 4	Cytoplasm	other		-1,987	5.12×10^-03
ENSG00000183735.9	TBK1	TANK binding kinase 1	Cytoplasm	kinase	6-aminopyrazolopyrimidine derivative compo	-2,599	2.10×10^-02
ENSG00000154144.12	TBRG1	transforming growth factor beta regulator 1	Nucleus	other		-1,421	3.38×10^-02
ENSG00000133142.17	TCEAL4	transcription elongation factor A like 4	Other	other		-2,109	2.97×10^-04
ENSG00000185222.7	TCEAL9	transcription elongation factor A like 9	Other	other		-1,628	6.36×10^-03
ENSG00000100207.18	TCF20	transcription factor 20	Nucleus	transcription regulator		-1,01	4.62×10^-02
ENSG00000120438.11	TCP1	t-complex 1	Cytoplasm	other		-1,677	1.06×10^-02
ENSG00000120156.20	TEK	TEK receptor tyrosine kinase	Plasma Membrane	kinase	CEP-11981, everolimus/vandetanib, caboza	-3,004	3.36×10^-03
ENSG00000166848.5	TERF2IP	TERF2 interacting protein	Nucleus	other		-1,549	3.68×10^-02
ENSG00000003436.14	TFPI	tissue factor pathway inhibitor	Extracellular Space	other	dalteparin, BAY1093884	-1,179	6.27×10^-03
ENSG00000105329.9	TGFB1	transforming growth factor beta 1	Extracellular Space	growth factor	YL-13027, dalantercept, SRK-181, NIS793,	-1,483	4.03×10^-02
ENSG00000163513.17	TGFBR2	transforming growth factor beta receptor 2	Plasma Membrane	kinase	IMC-TR1, SM1-71	-3,16	1.51×10^-07
ENSG00000198959.11	TGM2	transglutaminase 2	Cytoplasm	enzyme		-2,849	1.83×10^-07
ENSG00000137801.10	THBS1	thrombospondin 1	Extracellular Space	other		-5,966	2.61×10^-20
ENSG00000054118.13	THRAP3	thyroid hormone receptor associated protein 3	Nucleus	transcription regulator		-1,869	9.34×10^-04
ENSG00000066056.13	TIE1	tyrosine kinase with immunoglobulin like and EGF like domains 1	Plasma Membrane	kinase	WZ-4-145	-1,945	9.01×10^-03
ENSG00000134809.8	TIMM10	translocase of inner mitochondrial membrane 10	Cytoplasm	transporter		-3,114	7.81×10^-04
ENSG00000142910.15	TINAGL1	tubulointerstitial nephritis antigen like 1	Extracellular Space	transporter		-1,685	3.57×10^-02
ENSG00000104067.16	TJP1	tight junction protein 1	Plasma Membrane	other		-1,084	3.63×10^-02
ENSG00000137076.18	TLN1	talin 1	Plasma Membrane	other		-2,806	1.79×10^-06
ENSG00000107807.12	TLX1	T cell leukemia homeobox 1	Nucleus	transcription regulator		1,554	4.75×10^-02
ENSG00000170348.8	TMED10	transmembrane p24 trafficking protein 10	Cytoplasm	transporter		-1,533	2.14×10^-03
ENSG00000086598.10	TMED2	transmembrane p24 trafficking protein 2	Cytoplasm	transporter		-1,355	1.32×10^-02
ENSG00000152558.14	TMEM123	transmembrane protein 123	Plasma Membrane	other		-1,329	3.15×10^-02
ENSG00000164124.10	TMEM144	transmembrane protein 144	Other	other		1,524	8.06×10^-04
ENSG00000198792.12	TMEM184B	transmembrane protein 184B	Other	other		-2,937	8.68×10^-05
ENSG00000112697.15	TMEM30A	transmembrane protein 30A	Cytoplasm	transporter		-2,121	7.80×10^-04
ENSG00000257167.2	TMPO-AS1	TMPO antisense RNA 1	Other	other		1,626	3.65×10^-03
ENSG00000205542.10	TMSB10/TMSB4X	thymosin beta 4 X-linked	Cytoplasm	other		-2,261	2.86×10^-06
ENSG00000185215.8	TNFAIP2	TNF alpha induced protein 2	Extracellular Space	other		1,451	7.76×10^-03
ENSG00000118503.14	TNFAIP3	TNF alpha induced protein 3	Nucleus	enzyme		1,415	1.36×10^-02
ENSG00000149115.13	TNKS1BP1	tankyrase 1 binding protein 1	Nucleus	other		-1,578	3.11×10^-03
ENSG00000130204.12	TOMM40	translocase of outer mitochondrial membrane 40	Cytoplasm	ion channel		-1,883	4.47×10^-02
ENSG00000196683.10	TOMM7	translocase of outer mitochondrial membrane 7	Cytoplasm	transporter		-2,107	8.97×10^-03
ENSG00000198900.5	TOP1	DNA topoisomerase I	Nucleus	enzyme	bevacizumab/irinotecan/oxaliplatin, SN-38, b	-1,276	2.91×10^-02
ENSG00000131747.14	TOP2A	DNA topoisomerase II alpha	Nucleus	enzyme	bortezomib/doxorubicin, daunorubicin/tretino	-1,332	1.97×10^-02
ENSG00000077097.13	TOP2B	DNA topoisomerase II beta	Nucleus	enzyme	daunorubicin/tretinoin, chlorambucil/epirubic	-2,489	2.73×10^-04
ENSG00000101150.17	TPD52L2	TPD52 like 2	Cytoplasm	other		-1,393	3.90×10^-02
ENSG00000111669.14	TPI1	triosephosphate isomerase 1	Cytoplasm	enzyme		-1,836	1.86×10^-03
ENSG00000143549.19	TPM3	tropomyosin 3	Cytoplasm	other	AZD-7451	-1,328	1.31×10^-02
ENSG00000167460.14	TPM4	tropomyosin 4	Cytoplasm	other		-1,766	2.97×10^-03
ENSG00000047410.13	TPR	translocated promoter region, nuclear basket protein	Nucleus	other		-1,357	6.45×10^-03
ENSG00000188001.9	TPRG1	tumor protein p63 regulated 1	Cytoplasm	other		1,342	2.80×10^-03
ENSG00000133112.16	TPT1	tumor protein, translationally-controlled 1	Cytoplasm	other		-1,853	5.89×10^-04
ENSG00000115993.11	TRAK2	trafficking kinesin protein 2	Plasma Membrane	transporter		-2,661	5.42×10^-06
ENSG00000067167.7	TRAM1	translocation associated membrane protein 1	Cytoplasm	other		-2,142	2.18×10^-02
ENSG00000168538.15	TRAPPC11	trafficking protein particle complex subunit 11	Cytoplasm	other		-2,705	3.30×10^-02
ENSG00000181029.8	TRAPPC5	trafficking protein particle complex subunit 5	Cytoplasm	other		-1,923	1.49×10^-02
ENSG00000123144.10	TRIR	telomerase RNA component interacting RNase	Nucleus	enzyme		-2,235	4.11×10^-02
ENSG00000167721.10	TSR1	TSR1 ribosome maturation factor	Nucleus	other		-1,305	2.81×10^-02
ENSG00000113312.10	TTC1	tetratricopeptide repeat domain 1	Cytoplasm	other		-1,696	1.17×10^-02
ENSG00000167552.13	TUBA1A	tubulin alpha 1a	Cytoplasm	other	docetaxel/gemcitabine/vincristine, colchicine	-1,39	6.90×10^-03
ENSG00000123416.15	TUBA1B	tubulin alpha 1b	Cytoplasm	other		-2,019	1.15×10^-03
ENSG00000167553.14	TUBA1C	tubulin alpha 1c	Cytoplasm	other	docetaxel/gemcitabine/vincristine, colchicine	-1,909	2.97×10^-04
ENSG00000196230.12	TUBB	tubulin beta class I	Cytoplasm	other	ixabepilone/rifampin, allopurinol/colchicine, c	-3,037	8.12×10^-08
ENSG00000131462.7	TUBG1	tubulin gamma 1	Cytoplasm	other	docetaxel/gemcitabine/vincristine, colchicine	-2,069	1.30×10^-02
ENSG00000178952.8	TUFM	Tu translation elongation factor, mitochondrial	Cytoplasm	translation regulator		-1,809	5.05×10^-03
ENSG00000136810.12	TXN	thioredoxin	Cytoplasm	enzyme	PX-12	-1,382	3.63×10^-02
ENSG00000239264.8	TXNDC5	thioredoxin domain containing 5	Cytoplasm	enzyme		-3,932	1.33×10^-02
ENSG00000198431.15	TXNRD1	thioredoxin reductase 1	Cytoplasm	enzyme	arsenic trioxide/idarubicin/tretinoin, arsenic t	-1,315	2.75×10^-02
ENSG00000176890.15	TYMS	thymidylate synthetase	Nucleus	enzyme	pemetrexed, capecitabine/temozolomide, cy	-1,929	1.12×10^-02
ENSG00000130985.16	UBA1	ubiquitin like modifier activating enzyme 1	Cytoplasm	enzyme	MLN7243	-2,334	8.69×10^-04
ENSG00000221983.7	UBA52	ubiquitin A-52 residue ribosomal protein fusion product 1	Cytoplasm	enzyme		-1,357	2.11×10^-02
ENSG00000150991.14	UBC	ubiquitin C	Cytoplasm	enzyme		1,93	7.87×10^-04
ENSG00000109332.19	UBE2D3	ubiquitin conjugating enzyme E2 D3	Cytoplasm	enzyme		-1,011	2.82×10^-02
ENSG00000198833.6	UBE2J1	ubiquitin conjugating enzyme E2 J1	Cytoplasm	enzyme		-1,638	1.79×10^-02
ENSG00000185651.14	UBE2L3	ubiquitin conjugating enzyme E2 L3	Nucleus	enzyme		-1,623	3.40×10^-02
ENSG00000244687.11	UBE2V1	ubiquitin conjugating enzyme E2 V1	Nucleus	transcription regulator		-1,055	3.95×10^-02
ENSG00000110344.9	UBE4A	ubiquitination factor E4A	Cytoplasm	enzyme		-1,533	5.00×10^-02
ENSG00000135018.13	UBQLN1	ubiquilin 1	Cytoplasm	other		-1,592	1.03×10^-02
ENSG00000104517.12	UBR5	ubiquitin protein ligase E3 component n-recognin 5	Nucleus	enzyme		-1,503	7.29×10^-03
ENSG00000144224.16	UBXN4	UBX domain protein 4	Extracellular Space	other		-1,676	1.32×10^-03
ENSG00000136731.12	UGGT1	UDP-glucose glycoprotein glucosyltransferase 1	Cytoplasm	enzyme		-1,235	1.92×10^-02
ENSG00000102595.18	UGGT2	UDP-glucose glycoprotein glucosyltransferase 2	Cytoplasm	enzyme		-2,841	8.88×10^-03
ENSG00000198722.12	UNC13B	unc-13 homolog B	Cytoplasm	other		-2,575	1.81×10^-02
ENSG00000005007.12	UPF1	UPF1 RNA helicase and ATPase	Nucleus	enzyme		-1,839	4.01×10^-02
ENSG00000184076.12	UQCR10	ubiquinol-cytochrome c reductase, complex III subunit X	Cytoplasm	enzyme		-1,84	3.22×10^-02
ENSG00000127540.11	UQCR11	ubiquinol-cytochrome c reductase, complex III subunit XI	Cytoplasm	enzyme		-2,232	3.46×10^-02
ENSG00000010256.10	UQCRC1	ubiquinol-cytochrome c reductase core protein 1	Cytoplasm	enzyme		-2,25	2.38×10^-03
ENSG00000173660.11	UQCRH	ubiquinol-cytochrome c reductase hinge protein	Cytoplasm	enzyme		-2,255	6.57×10^-03
ENSG00000164405.10	UQCRQ	ubiquinol-cytochrome c reductase complex III subunit VII	Cytoplasm	enzyme		-1,982	6.90×10^-04
ENSG00000103005.11	USB1	U6 snRNA biogenesis phosphodiesterase 1	Nucleus	enzyme		-1,521	1.70×10^-03
ENSG00000105698.15	USF2	upstream transcription factor 2, c-fos interacting	Nucleus	transcription regulator		-1,849	2.51×10^-02
ENSG00000138768.14	USO1	USO1 vesicle transport factor	Cytoplasm	other		-2,096	3.68×10^-02
ENSG00000124422.11	USP22	ubiquitin specific peptidase 22	Nucleus	peptidase		-1,239	4.90×10^-02
ENSG00000134588.12	USP26	ubiquitin specific peptidase 26	Cytoplasm	peptidase		1,515	3.97×10^-02
ENSG00000156697.12	UTP14A	UTP14A small subunit processome component	Nucleus	other		-1,71	6.28×10^-03
ENSG00000152818.18	UTRN	utrophin	Plasma Membrane	transmembrane receptor		-3,011	5.12×10^-09
ENSG00000049245.12	VAMP3	vesicle associated membrane protein 3	Plasma Membrane	other		-2,592	1.24×10^-02
ENSG00000071246.10	VASH1	vasohibin 1	Extracellular Space	peptidase		-1,148	2.23×10^-02
ENSG00000108828.15	VAT1	vesicle amine transport 1	Plasma Membrane	transporter		-2,146	5.37×10^-03
ENSG00000171724.2	VAT1L	vesicle amine transport 1 like	Other	enzyme		1,858	7.80×10^-04
ENSG00000165280.15	VCP	valosin containing protein	Cytoplasm	enzyme	CB-5339, CB-5083	-3,19	5.31×10^-07
ENSG00000213585.10	VDAC1	voltage dependent anion channel 1	Cytoplasm	ion channel		-1,37	1.29×10^-02
ENSG00000165637.13	VDAC2	voltage dependent anion channel 2	Cytoplasm	ion channel		-1,772	1.66×10^-02
ENSG00000078668.13	VDAC3	voltage dependent anion channel 3	Cytoplasm	ion channel		-2,1	7.39×10^-03
ENSG00000112715.20	VEGFA	vascular endothelial growth factor A	Extracellular Space	growth factor	bevacizumab/capecitabine, bevacizumab/te	16,773	0.00×10^00
ENSG00000173511.9	VEGFB	vascular endothelial growth factor B	Extracellular Space	growth factor	aflibercept, aflibercept/irinotecan, aflibercept	1,566	1.18×10^-02
ENSG00000129003.15	VPS13C	vacuolar protein sorting 13 homolog C	Cytoplasm	other		-1,291	2.55×10^-02
ENSG00000149823.7	VPS51	VPS51 subunit of GARP complex	Cytoplasm	other		-3,265	2.50×10^-02
ENSG00000132821.11	VSTM2L	V-set and transmembrane domain containing 2 like	Extracellular Space	other		3,968	1.68×10^-02
ENSG00000110799.13	VWF	von Willebrand factor	Extracellular Space	other	F8, caplacizumab, turoctocog alfa	-4,951	2.83×10^-13
ENSG00000158195.10	WASF2	WASP family member 2	Plasma Membrane	other		-1,236	2.00×10^-02
ENSG00000099290.15	WASHC2A/WASHC2C	WASH complex subunit 2A	Cytoplasm	other		-1,521	2.35×10^-02
ENSG00000136051.13	WASHC4	WASH complex subunit 4	Cytoplasm	other		-1,606	3.45×10^-02
ENSG00000120688.8	WBP4	WW domain binding protein 4	Cytoplasm	other		-2,708	4.34×10^-02
ENSG00000071127.16	WDR1	WD repeat domain 1	Extracellular Space	other		-2,44	8.54×10^-05
ENSG00000163811.11	WDR43	WD repeat domain 43	Nucleus	transcription regulator		-2,548	1.97×10^-05
ENSG00000018408.14	WWTR1	WW domain containing transcription regulator 1	Nucleus	transcription regulator		-1,565	2.34×10^-03
ENSG00000229807.9	XIST	X inactive specific transcript	Nucleus	other		-1,962	8.80×10^-06
ENSG00000130227.16	XPO7	exportin 7	Nucleus	transporter		-1,91	2.21×10^-02
ENSG00000184575.11	XPOT	exportin for tRNA	Nucleus	other		-1,174	2.30×10^-02
ENSG00000079246.15	XRCC5	X-ray repair cross complementing 5	Nucleus	enzyme		-2,119	4.32×10^-05
ENSG00000196419.12	XRCC6	X-ray repair cross complementing 6	Nucleus	enzyme		-2,213	9.64×10^-05
ENSG00000088930.7	XRN2	5'-3' exoribonuclease 2	Nucleus	enzyme		-3,168	8.03×10^-03
ENSG00000065978.17	YBX1	Y-box binding protein 1	Nucleus	transcription regulator		-1,731	3.02×10^-03
ENSG00000176105.13	YES1	YES proto-oncogene 1, Src family tyrosine kinase	Cytoplasm	kinase	afatinib/dasatinib, dasatinib, JNJ-26483327,	-1,828	1.27×10^-02
ENSG00000250067.11	YJEFN3	YjeF N-terminal domain containing 3	Other	other		-1,658	3.89×10^-03
ENSG00000166913.12	YWHAB	tyrosine 3-monooxygenase/tryptophan 5-monooxygenase activation protein beta	Cytoplasm	other		-1,502	3.89×10^-03
ENSG00000108953.16	YWHAE	tyrosine 3-monooxygenase/tryptophan 5-monooxygenase activation protein epsilon	Cytoplasm	other		-1,869	7.80×10^-04
ENSG00000164924.17	YWHAZ	tyrosine 3-monooxygenase/tryptophan 5-monooxygenase activation protein zeta	Cytoplasm	enzyme		-1,885	6.22×10^-04
ENSG00000250802.6	ZBED3-AS1	ZBED3 antisense RNA 1	Other	other		1,303	4.62×10^-02
ENSG00000123200.16	ZC3H13	zinc finger CCCH-type containing 13	Extracellular Space	other		-2,017	1.14×10^-04
ENSG00000148516.21	ZEB1	zinc finger E-box binding homeobox 1	Nucleus	transcription regulator		-1,176	2.70×10^-02
ENSG00000172671.19	ZFAND4	zinc finger AN1-type containing 4	Other	other		1,524	7.17×10^-04
ENSG00000253661.1	ZFHX4-AS1	ZFHX4 antisense RNA 1	Other	other		2,478	1.34×10^-02
ENSG00000128016.5	ZFP36	ZFP36 ring finger protein	Nucleus	transcription regulator		1,447	1.28×10^-02
ENSG00000152518.6	ZFP36L2	ZFP36 ring finger protein like 2	Nucleus	transcription regulator		1,368	2.43×10^-03
ENSG00000083838.15	ZNF446	zinc finger protein 446	Nucleus	transcription regulator		-3,552	1.71×10^-02
ENSG00000265778.2	ZNF516-AS1	ZNF516 antisense RNA 1	Other	other		2,477	9.90×10^-03
ENSG00000198795.10	ZNF521	zinc finger protein 521	Nucleus	transcription regulator		-1,695	1.02×10^-02
ENSG00000167766.18	ZNF83	zinc finger protein 83	Nucleus	transcription regulator		-1,645	4.14×10^-02
ENSG00000185453.12	ZSWIM9	zinc finger SWIM-type containing 9	Other	other		-1,432	8.72×10^-03

Suppl. Table 2: Deregulated genes in extracellular vesicles secreted from HUVEC cells after LNP treatment

Ensembl ID	Symbol	Entrez Gene Name	Location	Type(s)	Drug(s)	logFC	FDR
ENSG00000275877.1	5_8S_rRNA		Other	other		3,779	1.67×10^-02
ENSG00000234969.1	AARSD1P1		Other	other		4,029	4.25×10^-02
ENSG00000179869.14	ABCA13	ATP binding cassette subfamily A member 13	Extracellular Space	transporter		1,069	1.94×10^-02
ENSG00000114626.17	ABTB1	ankyrin repeat and BTB domain containing 1	Cytoplasm	translation regulator		-1,142	2.05×10^-02
ENSG00000231043.3	AC0072381		Other	other		-1,566	2.17×10^-02
ENSG00000235725.1	AC0073893		Other	other		-1,557	3.79×10^-02
ENSG00000261079.1	AC009053.2		Other	other		-1,571	2.13×10^-02
ENSG00000238045.9	AC009133.12		Other	other		5,457	9.69×10^-05
ENSG00000229143.1	AC0094804		Other	other		5,096	4.06×10^-03
ENSG00000225840.2	AC010970.2		Other	other		9,18	6.01×10^-127
ENSG00000279426.1	AC0932302		Other	other		-1,378	4.74×10^-02
ENSG00000227878.2	AC1147601		Other	other		3,221	2.54×10^-02
ENSG00000275249.1	AC171558.3		Other	other		-3,939	1.17×10^-02
ENSG00000182827.8	ACBD3	acyl-CoA binding domain containing 3	Cytoplasm	other		2,255	1.70×10^-04
ENSG00000087085.13	ACHE	acetylcholinesterase (Cartwright blood group)	Plasma Membrane	enzyme	huperzine A, pyridostigmine,	-1,277	3.22×10^-02
ENSG00000177465.4	ACOT4	acyl-CoA thioesterase 4	Cytoplasm	enzyme		-2,107	3.37×10^-02
ENSG00000123130.16	ACOT9	acyl-CoA thioesterase 9	Cytoplasm	enzyme		1,73	1.73×10^-03
ENSG00000075624.13	ACTB	actin beta	Cytoplasm	other		2,915	3.84×10^-57
ENSG00000184009.9	ACTG1	actin gamma 1	Cytoplasm	other		2,681	1.81×10^-38
ENSG00000130402.11	ACTN4	actinin alpha 4	Cytoplasm	transcription regulator		1,486	8.41×10^-04
ENSG00000138071.13	ACTR2	actin related protein 2	Plasma Membrane	other		2,293	1.77×10^-05
ENSG00000105963.13	ADAP1	ArfGAP with dual PH domains 1	Nucleus	other		3,123	2.75×10^-07
ENSG00000129467.13	ADCY4	adenylate cyclase 4	Plasma Membrane	enzyme		4,783	4.81×10^-16
ENSG00000148700.13	ADD3	adducin 3	Cytoplasm	other		1,238	3.65×10^-02
ENSG00000256925.2	ADGRA1-AS1	ADGRA1 antisense RNA 1	Other	other		-1,442	3.93×10^-02
ENSG00000173698.17	ADGRG2	adhesion G protein-coupled receptor G2	Plasma Membrane	G-protein coupled receptor		1,339	1.75×10^-02
ENSG00000072071.16	ADGRL1	adhesion G protein-coupled receptor L1	Plasma Membrane	G-protein coupled receptor		-1,219	1.17×10^-02
ENSG00000162618.12	ADGRL4	adhesion G protein-coupled receptor L4	Plasma Membrane	G-protein coupled receptor		2,073	6.55×10^-03
ENSG00000254936.4	AF1312153		Other	other		1,367	9.63×10^-03
ENSG00000157510.13	AFAP1L1	actin filament associated protein 1 like 1	Other	other		2,003	1.16×10^-04
ENSG00000155966.13	AFF2	AF4/FMR2 family member 2	Nucleus	other		1,01	1.47×10^-02
ENSG00000006530.15	AGK	acylglycerol kinase	Cytoplasm	kinase	SM1-71	1,251	2.43×10^-03
ENSG00000155189.11	AGPAT5	1-acylglycerol-3-phosphate O-acyltransferase 5	Cytoplasm	enzyme		1,318	2.36×10^-02
ENSG00000234187.1	AIMP1P1	aminoacyl tRNA synthetase complex interacting multifunctional protein 1 pseudogene 1	Other	other		2,379	1.48×10^-02
ENSG00000131016.16	AKAP12	A-kinase anchoring protein 12	Cytoplasm	transporter		1,294	4.17×10^-11
ENSG00000143149.12	ALDH9A1	aldehyde dehydrogenase 9 family member A1	Cytoplasm	enzyme		2,021	3.63×10^-02
ENSG00000179148.9	ALOXE3	arachidonate lipoxygenase 3	Extracellular Space	enzyme		-2,51	3.85×10^-02
ENSG00000178171.10	AMER3	APC membrane recruitment protein 3	Other	other		-1,323	4.07×10^-02
ENSG00000135409.10	AMHR2	anti-Mullerian hormone receptor type 2	Plasma Membrane	kinase	GM102	-1,196	1.17×10^-02
ENSG00000101935.9	AMMECR1	AMMECR nuclear protein 1	Nucleus	other		1,592	1.93×10^-02
ENSG00000174606.12	ANGEL2	angel homolog 2	Nucleus	other		1,066	1.69×10^-02
ENSG00000198720.12	ANKRD13B	ankyrin repeat domain 13B	Plasma Membrane	other		-2,63	2.09×10^-02
ENSG00000107890.16	ANKRD26	ankyrin repeat domain 26	Nucleus	transcription regulator		1,251	4.24×10^-02
ENSG00000180777.13	ANKRD30B	ankyrin repeat domain 30B	Extracellular Space	other		1,764	2.72×10^-02
ENSG00000198483.12	ANKRD35	ankyrin repeat domain 35	Other	other		-2,524	3.03×10^-02
ENSG00000214262.4	ANKRD36BP1	ankyrin repeat domain 36B pseudogene 1	Other	other		3,151	2.03×10^-02
ENSG00000151458.11	ANKRD50	ankyrin repeat domain 50	Other	other		2,056	7.96×10^-04
ENSG00000011426.10	ANLN	anillin actin binding protein	Cytoplasm	other		1,335	3.23×10^-02
ENSG00000011201.10	ANOS1	anosmin 1	Extracellular Space	other		1,571	3.72×10^-02
ENSG00000136938.8	ANP32B	acidic nuclear phosphoprotein 32 family member B	Nucleus	other		3,005	3.21×10^-38
ENSG00000103254.9	ANTKMT	adenine nucleotide translocase lysine methyltransferase	Cytoplasm	enzyme		6,564	4.89×10^-05
ENSG00000182718.16	ANXA2	annexin A2	Plasma Membrane	other		1,524	1.75×10^-11
ENSG00000164111.14	ANXA5	annexin A5	Plasma Membrane	transporter		1,6	7.01×10^-03
ENSG00000132842.13	AP3B1	adaptor related protein complex 3 subunit beta 1	Plasma Membrane	transporter		1,534	4.53×10^-03
ENSG00000077420.15	APBB1IP	amyloid beta precursor protein binding family B member 1 interacting protein	Cytoplasm	other		3,357	1.03×10^-31
ENSG00000110244.6	APOA4	apolipoprotein A4	Extracellular Space	transporter		-3,125	4.62×10^-02
ENSG00000184730.10	APOBR	apolipoprotein B receptor	Plasma Membrane	transmembrane receptor		-1,374	9.06×10^-05
ENSG00000142192.20	APP	amyloid beta precursor protein	Plasma Membrane	other	bapineuzumab, florbetapir F1	2,138	5.88×10^-11
ENSG00000214293.8	APTR	Alu-mediated CDKN1A/p21 transcriptional regulator	Other	other		1,533	3.62×10^-02
ENSG00000101246.19	ARFRP1	ADP ribosylation factor related protein 1	Cytoplasm	enzyme		-1,401	4.79×10^-02
ENSG00000146376.10	ARHGAP18	Rho GTPase activating protein 18	Cytoplasm	other		1,866	4.78×10^-03
ENSG00000186517.13	ARHGAP30	Rho GTPase activating protein 30	Cytoplasm	other		-1,021	6.33×10^-03
ENSG00000111348.8	ARHGDIB	Rho GDP dissociation inhibitor beta	Cytoplasm	enzyme		2,165	1.14×10^-06
ENSG00000213214.4	ARHGEF35	Rho guanine nucleotide exchange factor 35	Other	other		-1,06	4.91×10^-03
ENSG00000129675.15	ARHGEF6	Rac/Cdc42 guanine nucleotide exchange factor 6	Cytoplasm	other		1,534	4.61×10^-02
ENSG00000182196.13	ARL6IP4	ADP ribosylation factor like GTPase 6 interacting protein 4	Nucleus	other		1,865	9.96×10^-05
ENSG00000163466.15	ARPC2	actin related protein 2/3 complex subunit 2	Cytoplasm	other		1,197	9.19×10^-04
ENSG00000111229.15	ARPC3	actin related protein 2/3 complex subunit 3	Cytoplasm	other		2,327	1.13×10^-08
ENSG00000183876.8	ARSI	arylsulfatase family member I	Extracellular Space	enzyme		-2,07	8.21×10^-03
ENSG00000188611.14	ASAH2	N-acylsphingosine amidohydrolase 2	Cytoplasm	enzyme		1,166	1.93×10^-02
ENSG00000153317.14	ASAP1	ArfGAP with SH3 domain, ankyrin repeat and PH domain 1	Plasma Membrane	other		1,282	1.44×10^-02
ENSG00000066279.16	ASPM	assembly factor for spindle microtubules	Nucleus	other		1,095	1.94×10^-03
ENSG00000188886.3	ASTL	astacin like metalloendopeptidase	Cytoplasm	peptidase		-3,625	4.04×10^-02
ENSG00000115966.16	ATF2	activating transcription factor 2	Nucleus	transcription regulator		1,972	3.81×10^-02
ENSG00000128272.14	ATF4	activating transcription factor 4	Nucleus	transcription regulator		1,391	1.60×10^-03
ENSG00000105409.15	ATP1A3	ATPase Na+/K+ transporting subunit alpha 3	Plasma Membrane	transporter	digoxin, ethacrynic acid, perp	-1,342	4.08×10^-02
ENSG00000132681.16	ATP1A4	ATPase Na+/K+ transporting subunit alpha 4	Plasma Membrane	transporter	digoxin, ethacrynic acid, perp	-1,069	8.45×10^-03
ENSG00000101892.11	ATP1B4	ATPase Na+/K+ transporting family member beta 4	Plasma Membrane	transporter		1,212	4.71×10^-02
ENSG00000169020.9	ATP5ME	ATP synthase membrane subunit e	Cytoplasm	transporter		3,967	2.05×10^-05
ENSG00000156411.9	ATP5MJ	ATP synthase membrane subunit j	Cytoplasm	other		1,839	1.17×10^-03
ENSG00000154723.12	ATP5PF	ATP synthase peripheral stalk subunit F6	Cytoplasm	transporter		2,575	2.71×10^-08
ENSG00000136888.6	ATP6V1G1	ATPase H+ transporting V1 subunit G1	Cytoplasm	transporter		2,738	2.63×10^-02
ENSG00000085224.20	ATRX	ATRX chromatin remodeler	Nucleus	transcription regulator		1,526	1.48×10^-08
ENSG00000198049.6	AVPR1B	arginine vasopressin receptor 1B	Plasma Membrane	G-protein coupled receptor	AVP, lypressin	-1,684	1.37×10^-03
ENSG00000176022.4	B3GALT6	beta-1,3-galactosyltransferase 6	Cytoplasm	enzyme		-1,919	4.29×10^-02
ENSG00000255545.7	B3GAT1-DT	B3GAT1 divergent transcript	Other	other		-1,055	2.56×10^-02
ENSG00000175711.8	B3GNTL1	UDP-GlcNAc:betaGal beta-1,3-N-acetylglucosaminyltransferase like 1	Other	enzyme		1,094	4.01×10^-03
ENSG00000139044.10	B4GALNT3	beta-1,4-N-acetyl-galactosaminyltransferase 3	Cytoplasm	enzyme		-1,404	5.18×10^-04
ENSG00000187172.15	BAGE2	BAGE family member 2	Other	other		-1,173	6.35×10^-04
ENSG00000266074.8	BAHCC1	BAH domain and coiled-coil containing 1	Other	other		-1,252	2.02×10^-02
ENSG00000140320.11	BAHD1	bromo adjacent homology domain containing 1	Nucleus	other		-1,38	4.05×10^-02
ENSG00000009954.10	BAZ1B	bromodomain adjacent to zinc finger domain 1B	Nucleus	transcription regulator		1,469	1.29×10^-05
ENSG00000138686.9	BBS7	Bardet-Biedl syndrome 7	Cytoplasm	other		1,682	4.57×10^-02
ENSG00000060982.14	BCAT1	branched chain amino acid transaminase 1	Cytoplasm	enzyme		1,044	8.35×10^-04
ENSG00000105552.14	BCAT2	branched chain amino acid transaminase 2	Cytoplasm	enzyme		-1,613	3.23×10^-02
ENSG00000083123.14	BCKDHB	branched chain keto acid dehydrogenase E1 subunit beta	Cytoplasm	enzyme		1,682	4.05×10^-02
ENSG00000153094.21	BCL2L11	BCL2 like 11	Cytoplasm	other		1,405	9.98×10^-03
ENSG00000116128.9	BCL9	BCL9 transcription coactivator	Nucleus	other		-1,221	3.87×10^-02
ENSG00000173681.16	BCLAF3	BCLAF1 and THRAP3 family member 3	Other	other		1,081	4.67×10^-02
ENSG00000168398.6	BDKRB2	bradykinin receptor B2	Plasma Membrane	G-protein coupled receptor	anatibant, icatibant, lobradiml	-1,077	1.82×10^-02
ENSG00000177951.17	BET1L	Bet1 golgi vesicular membrane trafficking protein like	Cytoplasm	transporter		-1,754	8.08×10^-03
ENSG00000166681.13	BEX3	brain expressed X-linked 3	Cytoplasm	other		2,513	2.00×10^-04
ENSG00000235831.6	BHLHE40-AS1	BHLHE40 antisense RNA 1	Other	other		2,095	2.35×10^-02
ENSG00000248684.1	BIN2P2	bridging integrator 2 pseudogene 2	Other	other		-3,832	2.66×10^-02
ENSG00000104765.14	BNIP3L	BCL2 interacting protein 3 like	Cytoplasm	other		2,013	1.87×10^-02
ENSG00000269040.1	BNIP3P24	BCL2 interacting protein 3 pseudogene 24	Other	other		4,548	3.02×10^-02
ENSG00000254901.7	BORCS8	BLOC-1 related complex subunit 8	Cytoplasm	other		-1,265	1.24×10^-02
ENSG00000279208.1	BP_21264C11		Other	other		1,49	3.62×10^-03
ENSG00000064726.9	BTBD1	BTB domain containing 1	Cytoplasm	other		1,171	3.06×10^-02
ENSG00000166323.12	C11orf65	chromosome 11 open reading frame 65	Other	other		1,546	3.51×10^-02
ENSG00000179088.14	C12orf42	chromosome 12 open reading frame 42	Other	other		1,535	4.11×10^-02
ENSG00000167194.7	C16orf92	chromosome 16 open reading frame 92	Other	other		-3,62	4.14×10^-02
ENSG00000119559.15	C19orf25	chromosome 19 open reading frame 25	Other	other		-2,585	2.63×10^-02
ENSG00000104979.8	C19orf53	chromosome 19 open reading frame 53	Nucleus	other		2,824	9.70×10^-04
ENSG00000239887.4	C1orf226	chromosome 1 open reading frame 226	Other	other		-1,141	2.99×10^-02
ENSG00000164008.13	C1orf50	chromosome 1 open reading frame 50	Other	other		3,269	8.17×10^-03
ENSG00000177994.15	C2orf73	chromosome 2 open reading frame 73	Other	other		2,478	1.41×10^-02
ENSG00000147894.14	C9orf72	C9orf72-SMCR8 complex subunit	Cytoplasm	other		1,842	4.89×10^-02
ENSG00000063180.8	CA11	carbonic anhydrase 11	Extracellular Space	enzyme		-1,832	1.18×10^-05
ENSG00000135932.10	CAB39	calcium binding protein 39	Cytoplasm	enzyme		1,409	2.54×10^-03
ENSG00000100314.3	CABP7	calcium binding protein 7	Cytoplasm	other		-2,018	4.39×10^-02
ENSG00000223770.5	CACNA2D1-AS1	CACNA2D1 antisense RNA 1	Other	other		2,127	8.97×10^-03
ENSG00000167535.7	CACNB3	calcium voltage-gated channel auxiliary subunit beta 3	Plasma Membrane	ion channel	verapamil, dexamethasone/ri	-1,115	7.23×10^-03
ENSG00000075461.5	CACNG4	calcium voltage-gated channel auxiliary subunit gamma 4	Plasma Membrane	ion channel		-1,836	3.16×10^-02
ENSG00000105605.7	CACNG7	calcium voltage-gated channel auxiliary subunit gamma 7	Plasma Membrane	ion channel		-1,692	4.76×10^-02
ENSG00000143933.16	CALM1 (includes others)	calmodulin 1	Cytoplasm	other		1,468	1.35×10^-05
ENSG00000198668.10	CALM1 (includes others)	calmodulin 1	Cytoplasm	other		1,487	4.36×10^-05
ENSG00000179218.13	CALR	calreticulin	Cytoplasm	transcription regulator		1,636	1.76×10^-06
ENSG00000127022.14	CANX	calnexin	Cytoplasm	other		1,675	4.69×10^-05
ENSG00000112186.11	CAP2	cyclase associated actin cytoskeleton regulatory protein 2	Plasma Membrane	other		1,93	3.36×10^-02
ENSG00000182472.8	CAPN12	calpain 12	Other	peptidase		-1,371	3.61×10^-02
ENSG00000135387.19	CAPRIN1	cell cycle associated protein 1	Plasma Membrane	other		1,161	1.15×10^-02
ENSG00000198898.12	CAPZA2	capping actin protein of muscle Z-line subunit alpha 2	Cytoplasm	other		1,122	1.67×10^-03

ENSG00000165233.17	CARD19	caspase recruitment domain family member 19	Cytoplasm	other		-1,406	4.73×10^-02
ENSG00000268001.1	CARD8-AS1	CARD8 antisense RNA 1	Other	other		2,383	1.85×10^-03
ENSG00000153048.10	CARHSP1	calcium regulated heat stable protein 1	Cytoplasm	transcription regulator		-1,422	3.86×10^-02
ENSG00000105141.5	CASP14	caspase 14	Cytoplasm	peptidase		-1,185	6.29×10^-03
ENSG00000165806.19	CASP7	caspase 7	Cytoplasm	peptidase		1,084	3.68×10^-03
ENSG00000121691.4	CAT	catalase	Cytoplasm	enzyme	fomepizole	2,075	2.29×10^-02
ENSG00000158428.3	CATIP	ciliogenesis associated TTC17 interacting protein	Cytoplasm	other		-1,414	3.70×10^-02
ENSG00000141668.9	CBLN2	cerebellin 2 precursor	Extracellular Space	other		1,104	1.94×10^-02
ENSG00000196873.15	CBWD3/CBWD6	COBW domain containing 3	Other	other		1,056	6.33×10^-04
ENSG00000060339.13	CCAR1	cell division cycle and apoptosis regulator 1	Nucleus	transcription regulator		1,477	5.70×10^-03
ENSG00000167131.16	CCDC103	coiled-coil domain containing 103	Cytoplasm	other		1,415	8.46×10^-04
ENSG00000160799.11	CCDC12	coiled-coil domain containing 12	Other	other		-1,056	1.44×10^-02
ENSG00000152076.18	CCDC74B	coiled-coil domain containing 74B	Other	other		-1,845	1.84×10^-02
ENSG00000149231.11	CCDC82	coiled-coil domain containing 82	Other	other		1,078	9.15×10^-03
ENSG00000142039.3	CCDC97	coiled-coil domain containing 97	Other	other		-1,162	1.09×10^-02
ENSG00000108700.4	CCL8	C-C motif chemokine ligand 8	Extracellular Space	cytokine		3,069	1.16×10^-02
ENSG00000100814.17	CCNB1IP1	cyclin B1 interacting protein 1	Nucleus	enzyme		-1,219	3.23×10^-02
ENSG00000113328.18	CCNG1	cyclin G1	Nucleus	other		1,598	1.80×10^-02
ENSG00000105219.8	CCNP	cyclin P	Nucleus	other		-2,479	3.78×10^-02
ENSG00000160791.13	CCR5	C-C motif chemokine receptor 5	Plasma Membrane	G-protein coupled receptor	PRO 140, maraviroc, vicriviro	-1,565	2.69×10^-05
ENSG00000150753.11	CCT5	chaperonin containing TCP1 subunit 5	Cytoplasm	other		1,619	7.94×10^-07
ENSG00000146731.10	CCT6A	chaperonin containing TCP1 subunit 6A	Cytoplasm	other		3,146	4.21×10^-03
ENSG00000174950.10	CD164L2	CD164 molecule like 2	Other	other		-1,652	1.77×10^-02
ENSG00000198821.10	CD247	CD247 molecule	Plasma Membrane	transmembrane receptor	blinatumomab	-1,224	3.67×10^-02
ENSG00000196352.13	CD55	CD55 molecule (Cromer blood group)	Plasma Membrane	other		1,947	8.38×10^-04
ENSG00000004897.11	CDC27	cell division cycle 27	Nucleus	other		1,649	1.69×10^-02
ENSG00000171219.8	CDC42BPG	CDC42 binding protein kinase gamma	Cytoplasm	kinase		-1,84	3.23×10^-06
ENSG00000107736.19	CDH23	cadherin related 23	Plasma Membrane	transporter		-1,005	2.29×10^-02
ENSG00000134058.10	CDK7	cyclin dependent kinase 7	Nucleus	kinase	roscovitine, XL102, BMS-387	3,45	1.68×10^-03
ENSG00000102384.13	CENPI	centromere protein I	Nucleus	other		2,405	1.77×10^-02
ENSG00000123219.12	CENPK	centromere protein K	Nucleus	other		1,062	3.18×10^-02
ENSG00000228615.1	CENPNP2	CENPN pseudogene 2	Other	other		-1,789	3.31×10^-02
ENSG00000174799.10	CEP135	centrosomal protein 135	Cytoplasm	other		1,251	4.94×10^-02
ENSG00000101639.18	CEP192	centrosomal protein 192	Cytoplasm	other		1,022	3.70×10^-02
ENSG00000160226.15	CFAP410	cilia and flagella associated protein 410	Cytoplasm	other		-2,751	4.38×10^-02
ENSG00000206530.8	CFAP44	cilia and flagella associated protein 44	Extracellular Space	peptidase		2,226	2.16×10^-25
ENSG00000181378.13	CFAP65	cilia and flagella associated protein 65	Cytoplasm	other		-1,488	1.27×10^-05
ENSG00000134389.9	CFHR5	complement factor H related 5	Extracellular Space	other		1,986	2.95×10^-02
ENSG00000172757.12	CFL1	cofilin 1	Nucleus	other		1,26	1.54×10^-03
ENSG00000196337.10	CGB7	chorionic gonadotropin subunit beta 7	Extracellular Space	other		-1,757	4.96×10^-02
ENSG00000143375.14	CGN	cingulin	Plasma Membrane	other		-1,273	8.22×10^-03
ENSG00000281181.1	CH507-513H4.3		Other	other		8,213	1.66×10^-13
ENSG00000280614.1	CH507-513H4.4		Other	other		6,947	1.06×10^-14
ENSG00000281383.1	CH507-513H4.5		Other	other		7,677	6.06×10^-11
ENSG00000280800.1	CH507-513H4.6		Other	other		7,186	3.65×10^-15
ENSG00000280179.1	CH507_42P116		Other	other		-4,898	5.46×10^-04
ENSG00000198824.5	CHAMP1	chromosome alignment maintaining phosphoprotein 1	Nucleus	other		-1,044	4.10×10^-02
ENSG00000272888.5	CHASERR	CHD2 adjacent suppressive regulatory RNA	Other	other		1,124	4.32×10^-02
ENSG00000106153.12	CHCHD2	coiled-coil-helix-coiled-coil-helix domain containing 2	Cytoplasm	other		3,577	3.63×10^-03
ENSG00000203668.1	CHML	CHM like Rab escort protein	Cytoplasm	enzyme		1,183	8.72×10^-03
ENSG00000278530.4	CHMP1B2P	charged multivesicular body protein 1B2, pseudogene	Other	other		1,175	1.05×10^-02
ENSG00000130724.8	CHMP2A	charged multivesicular body protein 2A	Cytoplasm	other		1,972	1.08×10^-03
ENSG00000054938.15	CHRDL2	chordin like 2	Cytoplasm	other		-1,177	3.65×10^-02
ENSG00000101204.15	CHRNA4	cholinergic receptor nicotinic alpha 4 subunit	Plasma Membrane	transmembrane receptor	succinylcholine, lobeline, rap	-1,228	5.71×10^-03
ENSG00000160716.4	CHRNB2	cholinergic receptor nicotinic beta 2 subunit	Plasma Membrane	transmembrane receptor	varenicline, ABT-089, ABT 4	-1,017	3.57×10^-02
ENSG00000232852.1	CICP4	capicua transcriptional repressor pseudogene 4	Other	other		-3,567	1.50×10^-02
ENSG00000160161.9	CILP2	cartilage intermediate layer protein 2	Extracellular Space	other		-4,379	4.57×10^-03
ENSG00000273145.1	CITF22_92A61		Other	other		-4,092	1.57×10^-02
ENSG00000171365.15	CLCN5	chloride voltage-gated channel 5	Plasma Membrane	ion channel		1,106	2.08×10^-03
ENSG00000164007.10	CLDN19	claudin 19	Plasma Membrane	other		-1,919	1.65×10^-02
ENSG00000213719.8	CLIC1	chloride intracellular channel 1	Nucleus	ion channel		2,326	3.80×10^-03
ENSG00000169504.14	CLIC4	chloride intracellular channel 4	Plasma Membrane	ion channel		1,863	9.31×10^-07
ENSG00000113282.13	CLINT1	clathrin interactor 1	Cytoplasm	other		1,081	7.21×10^-03
ENSG00000115295.19	CLIP4	CAP-Gly domain containing linker protein family member 4	Other	other		1,017	3.06×10^-02
ENSG00000134852.14	CLOCK	clock circadian regulator	Nucleus	transcription regulator		1,18	7.61×10^-03
ENSG00000122705.16	CLTA	clathrin light chain A	Plasma Membrane	other		1,884	5.03×10^-03
ENSG00000141367.11	CLTC	clathrin heavy chain	Plasma Membrane	other		1,022	1.72×10^-02
ENSG00000117519.15	CNN3	calponin 3	Cytoplasm	other		2,017	1.63×10^-04
ENSG00000168763.15	CNNM3	cyclin and CBS domain divalent metal cation transport mediator 3	Other	other		-1,413	2.46×10^-02
ENSG00000125107.16	CNOT1	CCR4-NOT transcription complex subunit 1	Cytoplasm	other		1,119	4.38×10^-02
ENSG00000115649.15	CNPPD1	cyclin Pas1/PHO80 domain containing 1	Other	other		-1,402	4.08×10^-02
ENSG00000168542.12	COL3A1	collagen type III alpha 1 chain	Extracellular Space	other	collagenase	1,493	1.10×10^-02
ENSG00000187498.14	COL4A1	collagen type IV alpha 1 chain	Extracellular Space	other	collagenase	1,026	2.28×10^-03
ENSG00000134871.17	COL4A2	collagen type IV alpha 2 chain	Extracellular Space	other	collagenase	1,289	9.19×10^-04
ENSG00000130635.15	COL5A1	collagen type V alpha 1 chain	Extracellular Space	other	collagenase	1,698	9.98×10^-13
ENSG00000198756.10	COLGALT2	collagen beta(1-O)galactosyltransferase 2	Cytoplasm	enzyme		1,031	3.00×10^-02
ENSG00000135940.6	COX5B	cytochrome c oxidase subunit 5B	Cytoplasm	enzyme		1,552	3.36×10^-02
ENSG00000226024.1	COX5BP7	cytochrome c oxidase subunit 5B pseudogene 7	Other	other		6,081	5.84×10^-12
ENSG00000127184.10	COX7C	cytochrome c oxidase subunit 7C	Cytoplasm	enzyme		1,35	8.41×10^-04
ENSG00000153002.11	CPB1	carboxypeptidase B1	Extracellular Space	peptidase		1,418	4.69×10^-03
ENSG00000168993.14	CPLX1	complexin 1	Plasma Membrane	transporter		-3,149	4.05×10^-02
ENSG00000178772.6	CPN2	carboxypeptidase N subunit 2	Extracellular Space	peptidase		-1,388	2.15×10^-02
ENSG00000113851.13	CRBN	cereblon	Cytoplasm	enzyme	lenalidomide, lenalidomide/te	1,169	2.88×10^-02
ENSG00000215908.7	CROCCP2	CROCC pseudogene 2	Other	other		-1,059	4.33×10^-02
ENSG00000176092.13	CRYBG2	crystallin beta-gamma domain containing 2	Extracellular Space	other		-2,321	3.14×10^-02
ENSG00000233025.1	CRYZP1	crystallin zeta pseudogene 1	Other	other		2,854	1.79×10^-02
ENSG00000009307.15	CSDE1	cold shock domain containing E1	Cytoplasm	enzyme		2,522	1.21×10^-11
ENSG00000268940.5	CT45A1 (includes others)	cancer/testis antigen family 45 member A2	Other	other		5,541	2.79×10^-03
ENSG00000232872.2	CTAGE3P	CTAGE family member 3, pseudogene	Other	other		-1,331	3.16×10^-02
ENSG00000276251.1	CTB_32O43		Other	other		-2,066	3.75×10^-02
ENSG00000253422.1	CTB_47B84		Other	other		3,647	4.74×10^-03
ENSG00000267385.1	CTB_50L1714		Other	other		-2,08	2.34×10^-02
ENSG00000175029.16	CTBP2	C-terminal binding protein 2	Nucleus	transcription regulator		1,117	8.66×10^-03
ENSG00000263571.1	CTC_304I175		Other	other		-3,681	2.49×10^-02
ENSG00000268906.1	CTC_518B29		Other	other		-2,323	1.32×10^-02
ENSG00000281348.1	CTD-2574D22.6		Other	other		-4,194	8.73×10^-03
ENSG00000275454.1	CTD_2026K114		Other	other		-3,359	4.97×10^-02
ENSG00000276107.1	CTD_2033D152		Other	other		2,63	4.07×10^-02
ENSG00000264932.3	CTD_2104P172		Other	other		4,774	1.22×10^-02
ENSG00000248288.1	CTD_2194F42		Other	other		2,857	9.35×10^-03
ENSG00000278898.1	CTD_2342N231		Other	other		1,437	4.83×10^-02
ENSG00000276570.1	CTD_2587H2414		Other	other		-1,567	4.82×10^-02
ENSG00000278972.1	CTD_3022L241		Other	other		1,62	4.38×10^-03
ENSG00000268120.1	CTD_3193O1311		Other	other		2,798	6.50×10^-04
ENSG00000214248.2	CTD_3193O1312		Other	other		-1,902	1.46×10^-03
ENSG00000168036.16	CTNNB1	catenin beta 1	Nucleus	transcription regulator	PRI-724	1,291	4.16×10^-02
ENSG00000055130.15	CUL1	cullin 1	Nucleus	enzyme		3,231	2.58×10^-02
ENSG00000166266.13	CUL5	cullin 5	Nucleus	ion channel		1,36	4.96×10^-02
ENSG00000163510.13	CWC22	CWC22 spliceosome associated protein homolog	Nucleus	other		2,331	8.87×10^-04
ENSG00000178927.16	CYBC1	cytochrome b-245 chaperone 1	Cytoplasm	other		-1,904	1.60×10^-02
ENSG00000198077.10	CYP2A6 (includes others)	cytochrome P450 family 2 subfamily A member 6	Cytoplasm	enzyme		-1,642	2.76×10^-02
ENSG00000138115.13	CYP2C8	cytochrome P450 family 2 subfamily C member 8	Cytoplasm	enzyme		1,798	1.27×10^-02
ENSG00000130649.9	CYP2E1	cytochrome P450 family 2 subfamily E member 1	Cytoplasm	enzyme		-1,147	1.78×10^-03
ENSG00000073067.13	CYP2W1	cytochrome P450 family 2 subfamily W member 1	Cytoplasm	enzyme		2,058	6.18×10^-04
ENSG00000172817.3	CYP7B1	cytochrome P450 family 7 subfamily B member 1	Cytoplasm	enzyme		2,226	3.06×10^-02
ENSG00000180432.5	CYP8B1	cytochrome P450 family 8 subfamily B member 1	Cytoplasm	enzyme		-1,147	4.29×10^-02
ENSG00000105443.13	CYTH2	cytohesin 2	Cytoplasm	other		-1,206	1.01×10^-03
ENSG00000155368.16	DBI	diazepam binding inhibitor, acyl-CoA binding protein	Cytoplasm	other		2,15	1.39×10^-06
ENSG00000163257.10	DCAF16	DDB1 and CUL4 associated factor 16	Nucleus	other		-1,517	1.09×10^-03
ENSG00000236397.3	DDX11L2	DEAD/H-box helicase 11 like 2 (pseudogene)	Other	other		-1,422	4.60×10^-02
ENSG00000215301.9	DDX3X	DEAD-box helicase 3 X-linked	Cytoplasm	enzyme		1,316	1.94×10^-03
ENSG00000108654.11	DDX5	DEAD-box helicase 5	Nucleus	enzyme		1,661	2.50×10^-08
ENSG00000174485.14	DENND4A	DENN domain containing 4A	Nucleus	other		1,118	2.66×10^-02
ENSG00000246422.2	DIAPH1-AS1	DIAPH1 antisense RNA 1	Other	other		1,274	3.17×10^-02
ENSG00000151208.16	DLG5	discs large MAGUK scaffold protein 5	Plasma Membrane	other		-1,757	7.87×10^-03
ENSG00000261520.5	DLGAP1-AS5	DLGAP1 antisense RNA 5	Other	other		1,162	1.77×10^-02
ENSG00000256061.7	DNAAF4	dynein axonemal assembly factor 4	Nucleus	other		3,037	3.27×10^-03
ENSG00000250091.2	DNAH10OS	dynein axonemal heavy chain 10 opposite strand	Other	other		-1,276	1.80×10^-02
ENSG00000183914.14	DNAH2	dynein axonemal heavy chain 2	Cytoplasm	other		3,276	1.31×10^-53
ENSG00000115423.18	DNAH6	dynein axonemal heavy chain 6	Extracellular Space	other		-2,771	4.28×10^-02
ENSG00000086061.15	DNAJA1	DnaJ heat shock protein family (Hsp40) member A1	Nucleus	other		2,146	1.84×10^-06
ENSG00000120675.5	DNAJC15	DnaJ heat shock protein family (Hsp40) member C15	Cytoplasm	other		1,081	1.01×10^-03
ENSG00000272636.3	DOC2B	double C2 domain beta	Cytoplasm	transporter		-1,429	2.93×10^-02
ENSG00000128512.19	DOCK4	dedicator of cytokinesis 4	Plasma Membrane	other		1,241	3.77×10^-03
ENSG00000088387.17	DOCK9	dedicator of cytokinesis 9	Cytoplasm	other		1,729	1.49×10^-07
ENSG00000015413.9	DPEP1	dipeptidase 1	Cytoplasm	peptidase	cilastatin	7,401	4.66×10^-76
ENSG00000136908.17	DPM2	dolichyl-phosphate mannosyltransferase subunit 2, regulatory	Cytoplasm	enzyme		-1,207	2.71×10^-02
ENSG00000224129.1	DPPA2P2	developmental pluripotency associated 2 pseudogene 2	Other	other		2,67	2.89×10^-02

ENSG00000092964.16	DPYSL2	dihydropyrimidinase like 2	Cytoplasm	enzyme		1,284	1.46×10^-04
ENSG00000171962.17	DRC3	dynein regulatory complex subunit 3	Cytoplasm	other		-1,181	2.93×10^-02
ENSG00000125868.15	DSTN	destrin, actin depolymerizing factor	Cytoplasm	other		1,997	2.28×10^-06
ENSG00000112679.14	DUSP22	dual specificity phosphatase 22	Cytoplasm	phosphatase		1,927	9.74×10^-08
ENSG00000197102.10	DYNC1H1	dynein cytoplasmic 1 heavy chain 1	Cytoplasm	peptidase		1,194	1.97×10^-05
ENSG00000167967.15	E4F1	E4F transcription factor 1	Nucleus	transcription regulator		-1,764	2.24×10^-02
ENSG00000156508.17	EEF1A1	eukaryotic translation elongation factor 1 alpha 1	Cytoplasm	translation regulator		3,427	1.86×10^-43
ENSG00000196205.8	EEF1A1P5	eukaryotic translation elongation factor 1 alpha 1 pseudogene 5	Extracellular Space	other		1,941	2.63×10^-02
ENSG00000114942.13	EEF1B2	eukaryotic translation elongation factor 1 beta 2	Cytoplasm	translation regulator		2,429	1.81×10^-09
ENSG00000104529.17	EEF1D	eukaryotic translation elongation factor 1 delta	Cytoplasm	translation regulator		1,275	5.25×10^-04
ENSG00000254772.9	EEF1G	eukaryotic translation elongation factor 1 gamma	Cytoplasm	translation regulator		2,939	7.60×10^-24
ENSG00000167658.15	EEF2	eukaryotic translation elongation factor 2	Cytoplasm	translation regulator	tagraxofusp, MDNA55	2,718	1.76×10^-27
ENSG00000115380.18	EFEMP1	EGF containing fibulin extracellular matrix protein 1	Extracellular Space	enzyme		1,192	1.57×10^-02
ENSG00000108947.4	EFNB3	ephrin B3	Plasma Membrane	kinase		-1,38	5.19×10^-03
ENSG00000255302.3	EID1	EP300 interacting inhibitor of differentiation 1	Nucleus	transcription regulator		1,748	1.09×10^-03
ENSG00000173812.10	EIF1	eukaryotic translation initiation factor 1	Cytoplasm	translation regulator		1,079	3.87×10^-02
ENSG00000262154.1	EIF1P4	eukaryotic translation initiation factor 1 pseudogene 4	Other	other		-3,933	1.78×10^-02
ENSG00000125977.6	EIF2S2	eukaryotic translation initiation factor 2 subunit beta	Cytoplasm	translation regulator		1,798	4.09×10^-05
ENSG00000130741.10	EIF2S3	eukaryotic translation initiation factor 2 subunit gamma	Cytoplasm	translation regulator		1,782	5.13×10^-03
ENSG00000107581.12	EIF3A	eukaryotic translation initiation factor 3 subunit A	Cytoplasm	translation regulator		1,805	1.20×10^-16
ENSG00000100353.17	EIF3D	eukaryotic translation initiation factor 3 subunit D	Cytoplasm	translation regulator		1,519	8.73×10^-03
ENSG00000104131.12	EIF3J	eukaryotic translation initiation factor 3 subunit J	Cytoplasm	translation regulator		2,258	3.51×10^-04
ENSG00000100129.17	EIF3L	eukaryotic translation initiation factor 3 subunit L	Cytoplasm	translation regulator		1,462	3.71×10^-03
ENSG00000161960.14	EIF4A1	eukaryotic translation initiation factor 4A1	Cytoplasm	translation regulator		1,453	3.27×10^-05
ENSG00000063046.17	EIF4B	eukaryotic translation initiation factor 4B	Cytoplasm	translation regulator		1,476	4.39×10^-06
ENSG00000110321.15	EIF4G2	eukaryotic translation initiation factor 4 gamma 2	Cytoplasm	translation regulator		1,583	1.47×10^-10
ENSG00000132507.17	EIF5A	eukaryotic translation initiation factor 5A	Cytoplasm	translation regulator	SNS01-T nanoparticles	1,626	8.83×10^-04
ENSG00000236081.1	ELFN1-AS1	ELFN1 antisense RNA 1	Other	other		-2,427	1.25×10^-03
ENSG00000183791.4	ELOA3P (includes others)	elongin A3, pseudogene	Nucleus	other		-3,615	1.75×10^-07
ENSG00000235640.1	ELOAP1	ELOA pseudogene 1	Other	other		1,785	1.42×10^-02
ENSG00000103363.14	ELOB	elongin B	Nucleus	transcription regulator		2,891	2.16×10^-03
ENSG00000161671.16	EMC10	ER membrane protein complex subunit 10	Cytoplasm	other		-1,352	2.68×10^-07
ENSG00000131148.8	EMC8	ER membrane protein complex subunit 8	Cytoplasm	other		1,173	4.55×10^-02
ENSG00000149499.11	EML3	EMAP like 3	Cytoplasm	other		1,318	4.56×10^-03
ENSG00000142227.10	EMP3	epithelial membrane protein 3	Plasma Membrane	other		-2,702	4.80×10^-02
ENSG00000135638.13	EMX1	empty spiracles homeobox 1	Nucleus	transcription regulator		-1,847	1.08×10^-03
ENSG00000074800.13	ENO1	enolase 1	Cytoplasm	enzyme		1,367	1.36×10^-03
ENSG00000108515.17	ENO3	enolase 3	Cytoplasm	enzyme		-1,302	3.61×10^-02
ENSG00000136960.12	ENPP2	ectonucleotide pyrophosphatase/phosphodiesterase 2	Plasma Membrane	enzyme	VPC8a202	1,35	4.15×10^-02
ENSG00000183317.16	EPHA10	EPH receptor A10	Plasma Membrane	transmembrane receptor	SM1-71	-1,497	3.65×10^-02
ENSG00000085832.16	EPS15	epidermal growth factor receptor pathway substrate 15	Cytoplasm	other		1,031	3.57×10^-02
ENSG00000225830.10	ERCC6	ERCC excision repair 6, chromatin remodeling factor	Nucleus	transcription regulator		1,133	9.94×10^-05
ENSG00000186871.6	ERCC6L	ERCC excision repair 6 like, spindle assembly checkpoint helicase	Nucleus	enzyme		1,517	1.74×10^-02
ENSG00000157554.18	ERG	ETS transcription factor ERG	Nucleus	transcription regulator		1,991	1.46×10^-12
ENSG00000135476.11	ESPL1	extra spindle pole bodies like 1, separase	Nucleus	peptidase		-1,501	2.75×10^-02
ENSG00000139641.12	ESYT1	extended synaptotagmin 1	Cytoplasm	other		1,8	1.22×10^-02
ENSG00000205707.10	ETFRF1	electron transfer flavoprotein regulatory factor 1	Cytoplasm	other		4,378	5.89×10^-06
ENSG00000151348.13	EXT2	exostosin glycosyltransferase 2	Cytoplasm	enzyme		1,196	2.88×10^-02
ENSG00000092820.17	EZR	ezrin	Plasma Membrane	other		1,612	2.44×10^-02
ENSG00000185010.13	F8	coagulation factor VIII	Extracellular Space	other	drotrecogin alfa, argatroban,	1,15	2.58×10^-02
ENSG00000170323.8	FABP4	fatty acid binding protein 4	Cytoplasm	transporter		3,159	3.66×10^-04
ENSG00000164687.10	FABP5	fatty acid binding protein 5	Cytoplasm	transporter		2,273	7.24×10^-15
ENSG00000135472.8	FAIM2	Fas apoptotic inhibitory molecule 2	Plasma Membrane	other		-1,045	2.99×10^-02
ENSG00000184083.11	FAM120C	family with sequence similarity 120C	Other	other		1,284	3.94×10^-02
ENSG00000138640.14	FAM13A	family with sequence similarity 13 member A	Cytoplasm	other		1,563	1.58×10^-08
ENSG00000031003.10	FAM13B	family with sequence similarity 13 member B	Cytoplasm	other		2,22	4.77×10^-04
ENSG00000143340.6	FAM163A	family with sequence similarity 163 member A	Other	other		-1,995	4.38×10^-02
ENSG00000234736.5	FAM170B-AS1	FAM170B antisense RNA 1	Other	other		-1,133	4.12×10^-02
ENSG00000214886.4	FAM177A1P1	Putative protein FAM177A2	Other	other		-2,277	8.77×10^-03
ENSG00000262497.1	FAM187B2P	family with sequence similarity 187, member B pseudogene	Other	other		-4,266	3.56×10^-03
ENSG00000225560.6	FAM197Y3 (includes others)	family with sequence similarity 197 Y-linked member 9	Other	other		-5,324	7.23×10^-05
ENSG00000124098.9	FAM210B	family with sequence similarity 210 member B	Extracellular Space	other		1,274	1.62×10^-02
ENSG00000178248.11	FAM230I	family with sequence similarity 230 member I	Other	other		-3,566	4.47×10^-02
ENSG00000223885.4	FAM90A26 (includes others)	family with sequence similarity 90 member A26	Other	other		-3,743	4.09×10^-02
ENSG00000149806.10	FAU	FAU ubiquitin like and ribosomal protein S30 fusion	Cytoplasm	other		1,308	1.61×10^-03
ENSG00000138081.19	FBXO11	F-box protein 11	Cytoplasm	enzyme		1,364	4.13×10^-02
ENSG00000159069.13	FBXW5	F-box and WD repeat domain containing 5	Cytoplasm	other		1,415	1.15×10^-02
ENSG00000109670.13	FBXW7	F-box and WD repeat domain containing 7	Nucleus	enzyme		1,06	5.97×10^-04
ENSG00000162897.14	FCAMR	Fc fragment of IgA and IgM receptor	Plasma Membrane	transmembrane receptor		-2,172	1.56×10^-02
ENSG00000275395.4	FCGBP	Fc fragment of IgG binding protein	Extracellular Space	other		-1,811	1.57×10^-05
ENSG00000150337.13	FCGR1A	Fc fragment of IgG receptor Ia	Plasma Membrane	transmembrane receptor	IgG	1,466	5.59×10^-03
ENSG00000275340.1	FGD5P1	FYVE, RhoGEF and PH domain containing 5 pseudogene 1	Other	other		-1,641	4.69×10^-05
ENSG00000160867.14	FGFR4	fibroblast growth factor receptor 4	Plasma Membrane	kinase	roblitinib, ASP5878, PRN137	-1,664	3.55×10^-02
ENSG00000176971.3	FIBIN	fin bud initiation factor homolog	Cytoplasm	other		5,775	2.81×10^-77
ENSG00000088832.14	FKBP1A	FKBP prolyl isomerase 1A	Cytoplasm	enzyme	methotrexate/sirolimus/tacrol	1,333	6.90×10^-03
ENSG00000143631.10	FLG	filaggrin	Cytoplasm	other		-1,209	1.85×10^-05
ENSG00000275620.1	FLJ16779	uncharacterized LOC100192386	Other	other		-1,067	7.33×10^-04
ENSG00000196924.14	FLNA	filamin A	Cytoplasm	other		1,517	8.75×10^-05
ENSG00000122025.14	FLT3	fms related receptor tyrosine kinase 3	Plasma Membrane	kinase	ningetinib, regorafenib/sunitir	3,055	3.79×10^-03
ENSG00000102081.13	FMR1	FMRP translational regulator 1	Cytoplasm	translation regulator		1,533	5.82×10^-06
ENSG00000115414.18	FN1	fibronectin 1	Extracellular Space	enzyme	ocriplasmin, bifikafusp alfa, L	1,164	9.52×10^-11
ENSG00000065970.8	FOXJ2	forkhead box J2	Nucleus	transcription regulator		6,891	4.20×10^-133
ENSG00000153303.16	FRMD1	FERM domain containing 1	Other	other		-1,241	3.37×10^-02
ENSG00000167996.15	FTH1	ferritin heavy chain 1	Cytoplasm	enzyme		3,042	3.71×10^-50
ENSG00000087086.13	FTL	ferritin light chain	Cytoplasm	enzyme		3,346	4.11×10^-21
ENSG00000162613.16	FUBP1	far upstream element binding protein 1	Nucleus	transcription regulator		1,677	6.97×10^-03
ENSG00000130383.7	FUT5	fucosyltransferase 5	Cytoplasm	enzyme		-3,708	9.84×10^-03
ENSG00000114416.17	FXR1	FMR1 autosomal homolog 1	Cytoplasm	other		1,221	6.90×10^-04
ENSG00000266964.5	FXYD1	FXYD domain containing ion transport regulator 1	Plasma Membrane	ion channel		-2,299	1.39×10^-02
ENSG00000137731.13	FXYD2	FXYD domain containing ion transport regulator 2	Plasma Membrane	ion channel	cyclothiazide	-1,852	2.73×10^-02
ENSG00000163251.3	FZD5	frizzled class receptor 5	Plasma Membrane	G-protein coupled receptor	wnt-5a mimic hexapeptide fo	1,855	2.05×10^-12
ENSG00000138757.14	G3BP2	G3BP stress granule assembly factor 2	Cytoplasm	enzyme		1,086	2.65×10^-02
ENSG00000131482.9	G6PC1	glucose-6-phosphatase catalytic subunit 1	Cytoplasm	phosphatase		-1,41	4.56×10^-02
ENSG00000160211.15	G6PD	glucose-6-phosphate dehydrogenase	Cytoplasm	enzyme		3,191	2.56×10^-02
ENSG00000011677.12	GABRA3	gamma-aminobutyric acid type A receptor subunit alpha3	Plasma Membrane	ion channel	flunitrazepam, sevoflurane, a	1,368	1.26×10^-02
ENSG00000099860.8	GADD45B	growth arrest and DNA damage inducible beta	Cytoplasm	other		-2,417	1.13×10^-02
ENSG00000197093.10	GAL3ST4	galactose-3-O-sulfotransferase 4	Cytoplasm	enzyme		-1,292	4.08×10^-02
ENSG00000182870.12	GALNT9	polypeptide N-acetylgalactosaminyltransferase 9	Cytoplasm	enzyme		-2,115	1.66×10^-02
ENSG00000111640.14	GAPDH	glyceraldehyde-3-phosphate dehydrogenase	Cytoplasm	enzyme		3,036	7.85×10^-34
ENSG00000166398.12	GARRE1	granule associated Rac and RHOG effector 1	Extracellular Space	other		1,222	3.08×10^-03
ENSG00000117226.11	GBP3	guanylate binding protein 3	Cytoplasm	enzyme		1,214	4.02×10^-02
ENSG00000205318.5	GCNT2P1	GCNT2 pseudogene 1	Other	other		3,064	4.92×10^-04
ENSG00000092208.16	GEMIN2	gem nuclear organelle associated protein 2	Nucleus	other		1,924	3.01×10^-02
ENSG00000100083.18	GGA1	golgi associated, gamma adaptin ear containing, ARF binding protein 1	Cytoplasm	transporter		-1,565	9.72×10^-03
ENSG00000115486.11	GGCX	gamma-glutamyl carboxylase	Cytoplasm	enzyme	anisindione, vitamin K3	1,03	6.23×10^-03
ENSG00000101193.7	GID8	GID complex subunit 8 homolog	Nucleus	other		1,117	3.57×10^-02
ENSG00000274944.4	GJA9-MYCBP	GJA9-MYCBP readthrough	Other	other		2,195	1.43×10^-04
ENSG00000198814.12	GK	glycerol kinase	Cytoplasm	kinase		2,175	3.70×10^-02
ENSG00000127920.5	GNG11	G protein subunit gamma 11	Plasma Membrane	enzyme		1,619	4.91×10^-03
ENSG00000186469.8	GNG2	G protein subunit gamma 2	Plasma Membrane	enzyme		1,34	3.90×10^-03
ENSG00000100522.8	GNPNAT1	glucosamine-phosphate N-acetyltransferase 1	Cytoplasm	enzyme		1,576	6.57×10^-03
ENSG00000111670.14	GNPTAB	N-acetylglucosamine-1-phosphate transferase subunits alpha and beta	Cytoplasm	enzyme		3,156	2.36×10^-07
ENSG00000144674.16	GOLGA4	golgin A4	Cytoplasm	other		1,124	6.87×10^-03
ENSG00000223698.3	GOLGA6L11P	golgin A6 family like 11, pseudogene	Other	other		-3,961	4.08×10^-02
ENSG00000261649.5	GOLGA6L7	golgin A6 family like 7	Other	other		-1,017	2.47×10^-05
ENSG00000278662.4	GOLGA6L9 (includes others)	golgin A6 family like 9	Other	other		1,116	1.54×10^-02
ENSG00000135052.16	GOLM1	golgi membrane protein 1	Cytoplasm	other		1,299	3.36×10^-02
ENSG00000152133.14	GPATCH11	G-patch domain containing 11	Nucleus	other		1,473	1.72×10^-02
ENSG00000236240.1	GPC5-IT1	GPC5 intronic transcript 1	Other	other		1,741	4.05×10^-02
ENSG00000175514.2	GPR152	G protein-coupled receptor 152	Plasma Membrane	G-protein coupled receptor		-3,825	1.46×10^-02
ENSG00000180758.11	GPR157	G protein-coupled receptor 157	Plasma Membrane	G-protein coupled receptor		-1,465	2.68×10^-02
ENSG00000178623.11	GPR35	G protein-coupled receptor 35	Plasma Membrane	G-protein coupled receptor		-1,857	2.66×10^-02
ENSG00000234696.1	GPR50-AS1	GPR50 antisense RNA 1	Other	other		2,272	3.16×10^-02
ENSG00000170412.16	GPRC5C	G protein-coupled receptor class C group 5 member C	Plasma Membrane	G-protein coupled receptor		-1,23	3.83×10^-02
ENSG00000204175.5	GPRIN2	G protein regulated inducer of neurite outgrowth 2	Other	other		-3,596	1.56×10^-02
ENSG00000167468.16	GPX4	glutathione peroxidase 4	Cytoplasm	enzyme		2,253	6.14×10^-11
ENSG00000105737.9	GRIK5	glutamate ionotropic receptor kainate type subunit 5	Plasma Membrane	ion channel		-2,119	2.98×10^-02
ENSG00000164082.14	GRM2	glutamate metabotropic receptor 2	Plasma Membrane	G-protein coupled receptor	talaglumetad, fasoracetam	-1,201	5.81×10^-04
ENSG00000229180.5	GS1_124K511		Other	other		-1,638	4.09×10^-05
ENSG00000254219.1	GS1_251I92		Other	other		4,564	8.75×10^-03
ENSG00000104518.10	GSDMD	gasdermin D	Extracellular Space	transporter		1,391	2.67×10^-02
ENSG00000180613.10	GSX2	GS homeobox 2	Nucleus	transcription regulator		2,342	1.61×10^-03
ENSG00000110768.11	GTF2H1	general transcription factor IIH subunit 1	Nucleus	transcription regulator		1,924	5.90×10^-04
ENSG00000155115.6	GTF3C6	general transcription factor IIIC subunit 6	Nucleus	transcription regulator		3,445	7.04×10^-03
ENSG00000187166.1	H1-7	H1.7 linker histone	Nucleus	other		-2,934	2.80×10^-02
ENSG00000130600.15	H19	H19 imprinted maternally expressed transcript	Cytoplasm	other		-3,472	1.88×10^-02
ENSG00000246705.4	H2AJ	H2A.J histone	Cytoplasm	other		1,587	3.61×10^-03
ENSG00000188486.3	H2AX	H2A.X variant histone	Nucleus	transcription regulator		1,948	2.46×10^-03

ENSG00000164032.11	H2AZ1	H2A.Z variant histone 1	Nucleus	other		1,308	4.34×10^-02
ENSG00000085382.11	HACE1	HECT domain and ankyrin repeat containing E3 ubiquitin protein ligase 1	Cytoplasm	enzyme		1,155	4.17×10^-02
ENSG00000084754.10	HADHA	hydroxyacyl-CoA dehydrogenase trifunctional multienzyme complex subunit alpha	Cytoplasm	enzyme		2,84	2.36×10^-04
ENSG00000061273.17	HDAC7	histone deacetylase 7	Nucleus	transcription regulator	tributyrin, belinostat, pyroxam	-1,153	9.35×10^-03
ENSG00000180229.12	HERC2P3	hect domain and RLD 2 pseudogene 3	Other	other		1,054	3.64×10^-04
ENSG00000049860.13	HEXB	hexosaminidase subunit beta	Cytoplasm	enzyme		1,951	2.43×10^-03
ENSG00000019991.15	HGF	hepatocyte growth factor	Extracellular Space	growth factor	rilotumumab, SCH 900105, b	7,698	5.68×10^-145
ENSG00000071794.15	HLTF	helicase like transcription factor	Nucleus	transcription regulator		1,339	6.90×10^-03
ENSG00000256269.6	HMBS	hydroxymethylbilane synthase	Cytoplasm	enzyme		-2,082	4.38×10^-02
ENSG00000137309.19	HMGA1	high mobility group AT-hook 1	Nucleus	transcription regulator		1,987	6.95×10^-07
ENSG00000189403.14	HMGB1	high mobility group box 1	Nucleus	transcription regulator		1,023	4.23×10^-04
ENSG00000254146.1	HMGB1P46	high mobility group box 1 pseudogene 46	Other	other		2,589	1.77×10^-02
ENSG00000205581.10	HMGN1	high mobility group nucleosome binding domain 1	Nucleus	transcription regulator		1,649	5.78×10^-05
ENSG00000182952.4	HMGN4	high mobility group nucleosomal binding domain 4	Nucleus	other		2,328	3.81×10^-02
ENSG00000198157.10	HMGN5	high mobility group nucleosome binding domain 5	Nucleus	transcription regulator		1,527	4.24×10^-02
ENSG00000135486.17	HNRNPA1	heterogeneous nuclear ribonucleoprotein A1	Nucleus	other		1,979	4.98×10^-22
ENSG00000122566.20	HNRNPA2B1	heterogeneous nuclear ribonucleoprotein A2/B1	Nucleus	other		1,895	1.42×10^-08
ENSG00000170144.18	HNRNPA3	heterogeneous nuclear ribonucleoprotein A3	Nucleus	other		1,28	6.17×10^-07
ENSG00000197451.10	HNRNPAB	heterogeneous nuclear ribonucleoprotein A/B	Nucleus	enzyme		2,109	1.48×10^-03
ENSG00000092199.17	HNRNPC	heterogeneous nuclear ribonucleoprotein C	Nucleus	other		1,797	1.09×10^-06
ENSG00000126945.8	HNRNPH2	heterogeneous nuclear ribonucleoprotein H2	Nucleus	other		2,182	1.23×10^-03
ENSG00000165119.18	HNRNPK	heterogeneous nuclear ribonucleoprotein K	Nucleus	other		1,926	2.06×10^-10
ENSG00000273046.1	HOXC5	homeobox C5	Nucleus	transcription regulator		-3,049	3.66×10^-03
ENSG00000130528.11	HRC	histidine rich calcium binding protein	Cytoplasm	other		-1,515	2.23×10^-04
ENSG00000153976.2	HS3ST3A1	heparan sulfate-glucosamine 3-sulfotransferase 3A1	Cytoplasm	enzyme		-4,726	1.31×10^-03
ENSG00000133835.14	HSD17B4	hydroxysteroid 17-beta dehydrogenase 4	Cytoplasm	enzyme		1,455	2.37×10^-03
ENSG00000080824.18	HSP90AA1	heat shock protein 90 alpha family class A member 1	Cytoplasm	enzyme	alvespimycin, retaspimycin, I	1,901	1.27×10^-14
ENSG00000096384.19	HSP90AB1	heat shock protein 90 alpha family class B member 1	Cytoplasm	enzyme	alvespimycin, retaspimycin, T	3,118	5.10×10^-32
ENSG00000109971.13	HSPA8	heat shock protein family A (Hsp70) member 8	Cytoplasm	enzyme		2,507	3.84×10^-24
ENSG00000251614.1	HSPA8P19	heat shock protein family A (Hsp70) member pseudogene 19	Other	other		3,495	4.97×10^-02
ENSG00000224773.2	HSPA8P7	heat shock protein family A (Hsp70) member 8 pseudogene 7	Other	other		2,278	3.97×10^-02
ENSG00000113013.12	HSPA9	heat shock protein family A (Hsp70) member 9	Cytoplasm	other		1,48	1.39×10^-03
ENSG00000106211.8	HSPB1	heat shock protein family B (small) member 1	Cytoplasm	other		2,283	2.72×10^-09
ENSG00000144381.16	HSPD1	heat shock protein family D (Hsp60) member 1	Cytoplasm	enzyme		2,211	1.97×10^-05
ENSG00000142798.16	HSPG2	heparan sulfate proteoglycan 2	Extracellular Space	enzyme		2,76	2.71×10^-46
ENSG00000102241.11	HTATSF1	HIV-1 Tat specific factor 1	Nucleus	transcription regulator		1,879	2.47×10^-03
ENSG00000086758.15	HUWE1	HECT, UBA and WWE domain containing E3 ubiquitin protein ligase 1	Nucleus	transcription regulator		1,044	4.61×10^-03
ENSG00000005700.14	IBTK	inhibitor of Bruton tyrosine kinase	Cytoplasm	other		1,59	3.81×10^-04
ENSG00000182054.9	IDH2	isocitrate dehydrogenase (NADP(+)) 2	Cytoplasm	enzyme	enasidenib, TQB3455, HMPL	2,469	3.58×10^-02
ENSG00000134049.4	IER3IP1	immediate early response 3 interacting protein 1	Cytoplasm	other		1,734	3.70×10^-03
ENSG00000255733.5	IFNG-AS1	IFNG antisense RNA 1	Other	other		3,233	4.27×10^-03
ENSG00000027697.12	IFNGR1	interferon gamma receptor 1	Plasma Membrane	transmembrane receptor	interferon gamma-1b	2,294	1.05×10^-02
ENSG00000101052.12	IFT52	intraflagellar transport 52	Cytoplasm	other		2,128	2.51×10^-03
ENSG00000163915.7	IGF2BP2-AS1	IGF2BP2 antisense RNA 1	Other	other		-1,427	3.29×10^-02
ENSG00000141753.6	IGFBP4	insulin like growth factor binding protein 4	Extracellular Space	other		3,24	3.27×10^-04
ENSG00000276566.1	IGKV1D-13	immunoglobulin kappa variable 1D-13	Other	other		-3,79	1.49×10^-02
ENSG00000113302.4	IL12B	interleukin 12B	Extracellular Space	cytokine	ustekinumab, methotrexate/u	1,51	3.67×10^-04
ENSG00000134352.19	IL6ST	interleukin 6 cytokine family signal transducer	Plasma Membrane	transmembrane receptor		1,047	1.61×10^-02
ENSG00000169592.14	INO80E	INO80 complex subunit E	Nucleus	other		-1,205	1.97×10^-02
ENSG00000125629.14	INSIG2	insulin induced gene 2	Cytoplasm	other		1,068	4.44×10^-02
ENSG00000130518.16	IQCN	IQ motif containing N	Cytoplasm	other		-1,351	1.55×10^-02
ENSG00000120645.11	IQSEC3	IQ motif and Sec7 domain ArfGEF 3	Cytoplasm	other		-1,127	2.84×10^-02
ENSG00000091409.14	ITGA6	integrin subunit alpha 6	Plasma Membrane	transmembrane receptor		1,712	4.78×10^-03
ENSG00000150093.18	ITGB1	integrin subunit beta 1	Plasma Membrane	transmembrane receptor	OS2966	1,774	1.56×10^-03
ENSG00000160255.16	ITGB2	integrin subunit beta 2	Plasma Membrane	transmembrane receptor		-1,21	2.35×10^-02
ENSG00000163207.6	IVL	involucrin	Cytoplasm	other		-1,605	4.39×10^-02
ENSG00000116679.15	IVNS1ABP	influenza virus NS1A binding protein	Nucleus	other		1,672	5.90×10^-04
ENSG00000162434.11	JAK1	Janus kinase 1	Cytoplasm	kinase	SHR0302, solcitinib, tofacitin	1,437	4.91×10^-03
ENSG00000105639.18	JAK3	Janus kinase 3	Cytoplasm	kinase	tofacitinib, R-348, decernotin	-1,564	4.11×10^-02
ENSG00000235012.1	JCADP1	JCAD pseudogene 1	Other	other		-1,683	1.83×10^-02
ENSG00000168970.20	JMJD7-PLA2G4B	JMJD7-PLA2G4B readthrough	Cytoplasm	other		-1,748	2.75×10^-02
ENSG00000146049.1	KAAG1	kidney associated antigen 1	Extracellular Space	other		-2,736	4.43×10^-02
ENSG00000274225.1	KB_68A71		Other	other		-4,33	4.35×10^-03
ENSG00000153822.13	KCNJ16	potassium inwardly rectifying channel subfamily J member 16	Plasma Membrane	ion channel		1,104	4.79×10^-03
ENSG00000075043.17	KCNQ2	potassium voltage-gated channel subfamily Q member 2	Plasma Membrane	ion channel		-1,465	3.88×10^-02
ENSG00000170745.11	KCNS3	potassium voltage-gated channel modifier subfamily S member 3	Plasma Membrane	ion channel		-1,374	1.79×10^-02
ENSG00000260231.1	KDM7A-DT	KDM7A divergent transcript	Other	other		-1,138	3.11×10^-02
ENSG00000189367.14	KIAA0408	KIAA0408	Other	other		-3,858	2.35×10^-02
ENSG00000130294.14	KIF1A	kinesin family member 1A	Cytoplasm	other		-1,744	1.23×10^-04
ENSG00000090889.11	KIF4A	kinesin family member 4A	Nucleus	other		2,8	1.54×10^-02
ENSG00000170759.10	KIF5B	kinesin family member 5B	Cytoplasm	other		1,781	3.61×10^-06
ENSG00000167633.16	KIR3DL1	killer cell immunoglobulin like receptor, three Ig domains and long cytoplasmic tail 1	Plasma Membrane	transmembrane receptor		-2,813	2.62×10^-03
ENSG00000003096.13	KLHL13	kelch like family member 13	Cytoplasm	other		1,85	2.62×10^-03
ENSG00000167548.14	KMT2D	lysine methyltransferase 2D	Nucleus	transcription regulator		-1,044	2.23×10^-03
ENSG00000171798.17	KNDC1	kinase non-catalytic C-lobe domain containing 1	Plasma Membrane	other		-2,125	3.30×10^-02
ENSG00000128944.13	KNSTRN	kinetochore localized astrin (SPAG5) binding protein	Cytoplasm	other		1,803	3.91×10^-02
ENSG00000205215.8	KRT17P7	keratin 17 pseudogene 7	Other	other		-3,648	4.28×10^-02
ENSG00000215606.4	KRT18P35	keratin 18 pseudogene 35	Other	other		3,829	3.18×10^-04
ENSG00000214514.7	KRT42P	keratin 42 pseudogene	Other	other		-1,036	4.86×10^-02
ENSG00000170454.5	KRT75	keratin 75	Cytoplasm	other		-2,289	3.64×10^-02
ENSG00000170423.12	KRT78	keratin 78	Cytoplasm	other		-1,172	4.10×10^-02
ENSG00000170421.11	KRT8	keratin 8	Cytoplasm	other		-1,131	3.65×10^-02
ENSG00000205445.3	KRTAP10-2	keratin associated protein 10-2	Cytoplasm	other		-3,545	2.90×10^-02
ENSG00000183640.5	KRTAP8-1	keratin associated protein 8-1	Cytoplasm	other		4,479	1.34×10^-03
ENSG00000126777.17	KTN1	kinectin 1	Plasma Membrane	transmembrane receptor		1,08	6.16×10^-05
ENSG00000198945.7	L3MBTL3	L3MBTL histone methyl-lysine binding protein 3	Nucleus	other		1,947	1.90×10^-04
ENSG00000262132.2	LA16C_390H24		Other	other		-2,19	4.62×10^-02
ENSG00000133706.17	LARS1	leucyl-tRNA synthetase 1	Cytoplasm	enzyme		1,516	1.00×10^-02
ENSG00000213626.11	LBH	LBH regulator of WNT signaling pathway	Nucleus	transcription regulator		-1,155	3.10×10^-02
ENSG00000196233.11	LCOR	ligand dependent nuclear receptor corepressor	Nucleus	transcription regulator		1,264	3.58×10^-05
ENSG00000115850.9	LCT	lactase	Plasma Membrane	enzyme		-1,287	2.06×10^-02
ENSG00000134333.13	LDHA	lactate dehydrogenase A	Cytoplasm	enzyme		1,244	8.59×10^-03
ENSG00000111716.12	LDHB	lactate dehydrogenase B	Cytoplasm	enzyme		2,782	2.71×10^-09
ENSG00000166477.12	LEO1	LEO1 homolog, Paf1/RNA polymerase II complex component	Nucleus	other		1,087	1.62×10^-02
ENSG00000168924.14	LETM1	leucine zipper and EF-hand containing transmembrane protein 1	Cytoplasm	transporter		2,519	3.29×10^-05
ENSG00000268660.1	LETM1P2	leucine zipper and EF-hand containing transmembrane protein 1 pseudogene 2	Other	other		-1,878	9.15×10^-03
ENSG00000100097.11	LGALS1	galectin 1	Extracellular Space	other	OTX008	4,258	1.42×10^-06
ENSG00000205076.4	LGALS7/LGALS7B	galectin 7	Extracellular Space	other		-2,335	5.77×10^-03
ENSG00000156959.8	LHFPL4	LHFPL tetraspan subfamily member 4	Plasma Membrane	other		-1,298	2.16×10^-04
ENSG00000205837.7	LINC00487	long intergenic non-protein coding RNA 487	Other	other		-2,209	4.07×10^-02
ENSG00000264575.1	LINC00526	long intergenic non-protein coding RNA 526	Other	other		-3,647	4.03×10^-02
ENSG00000234556.1	LINC00701	long intergenic non-protein coding RNA 701	Other	other		2,925	3.40×10^-02
ENSG00000235049.1	LINC00940	long intergenic non-protein coding RNA 940	Other	other		-1,906	9.00×10^-05
ENSG00000235314.1	LINC00957	long intergenic non-protein coding RNA 957	Other	other		-1,866	1.11×10^-03
ENSG00000244041.7	LINC01011	long intergenic non-protein coding RNA 1011	Other	other		1,207	3.57×10^-02
ENSG00000237094.11	LINC01347 (includes others)	long intergenic non-protein coding RNA 1347	Other	other		-1,094	2.33×10^-02
ENSG00000183250.11	LINC01547	long intergenic non-protein coding RNA 1547	Cytoplasm	other		-3,127	4.90×10^-02
ENSG00000196741.5	LINC01560	long intergenic non-protein coding RNA 1560	Other	other		1,672	3.37×10^-02
ENSG00000280350.1	LINC01726	long intergenic non-protein coding RNA 1726	Other	other		4,84	1.49×10^-05
ENSG00000267452.1	LINC02073		Other	other		-1,584	4.12×10^-02
ENSG00000248132.2	LINC02101	long intergenic non-protein coding RNA 2101	Other	other		4,417	8.14×10^-03
ENSG00000250387.2	LINC02197	long intergenic non-protein coding RNA 2197	Other	other		1,305	7.11×10^-03
ENSG00000282012.1	LL22NC03-N95F10.1		Other	other		5,718	1.60×10^-03
ENSG00000282416.1	LLNLF-173C4.2		Other	other		-3,936	2.14×10^-02
ENSG00000136944.17	LMX1B	LIM homeobox transcription factor 1 beta	Nucleus	transcription regulator		-1,333	2.36×10^-02
ENSG00000230000.2	LOC100287704/LOC100287834	uncharacterized LOC100287704	Other	other		-2,132	2.07×10^-02
ENSG00000229727.6	LOC100506274	uncharacterized LOC100506274	Other	other		-1,448	4.05×10^-02
ENSG00000259118.5	LOC100506321	uncharacterized LOC100506321	Other	other		3,177	2.86×10^-02
ENSG00000236252.2	LOC101928381	uncharacterized LOC101928381	Other	other		2,091	1.57×10^-02
ENSG00000241945.7	LOC102724159/PWP2	PWP2 small subunit processome component	Nucleus	other		3,242	4.62×10^-02
ENSG00000275464.3	LOC102724159/PWP2	PWP2 small subunit processome component	Nucleus	other		-1,904	1.61×10^-02
ENSG00000259774.1	LOC103171574	uncharacterized LOC103171574	Other	other		-1,508	2.56×10^-02
ENSG00000237070.1	LOC105375166	uncharacterized LOC105375166	Other	other		2,778	8.56×10^-03
ENSG00000228549.3	LOC112267871		Other	other		-1,464	3.48×10^-03
ENSG00000250658.1	LOC339975	uncharacterized LOC339975	Other	other		-2,449	3.03×10^-02
ENSG00000276077.4	LOC389831 (includes others)	uncharacterized LOC389831	Other	other		-5,122	1.70×10^-04
ENSG00000267665.1	LOC400622	uncharacterized LOC400622	Other	other		-1,84	1.36×10^-02
ENSG00000123384.13	LRP1	LDL receptor related protein 1	Plasma Membrane	transmembrane receptor	paclitaxel-angiopep-2 conjug	-1,666	5.52×10^-07
ENSG00000108829.9	LRRC59	leucine rich repeat containing 59	Cytoplasm	other		1,314	1.99×10^-03
ENSG00000166159.10	LRTM2	leucine rich repeats and transmembrane domains 2	Other	other		-1,334	4.57×10^-02
ENSG00000143429.9	LSP1P4	LSP1 pseudogene 4	Other	other		1,018	2.66×10^-02
ENSG00000090006.17	LTBP4	latent transforming growth factor beta binding protein 4	Extracellular Space	growth factor		-1,341	3.99×10^-03
ENSG00000079257.7	LXN	latexin	Cytoplasm	other		2,803	7.02×10^-04
ENSG00000272142.1	LYRM4-AS1	LYRM4 antisense RNA 1	Other	other		2,238	3.97×10^-02
ENSG00000163818.16	LZTFL1	leucine zipper transcription factor like 1	Cytoplasm	other		1,245	1.65×10^-02
ENSG00000107816.17	LZTS2	leucine zipper tumor suppressor 2	Cytoplasm	other		-1,338	1.17×10^-02
ENSG00000002822.15	MAD1L1	mitotic arrest deficient 1 like 1	Nucleus	other		-1,89	2.10×10^-03
ENSG00000183305.13	MAGEA2/MAGEA2B	MAGE family member A2	Nucleus	other		6,105	6.54×10^-16

ENSG00000162972.10	MAIP1	matrix AAA peptidase interacting protein 1	Cytoplasm	other		3,704	6.35×10^-04
ENSG00000117643.14	MAN1C1	mannosidase alpha class 1C member 1	Cytoplasm	enzyme		-1,06	2.01×10^-02
ENSG00000140941.12	MAP1LC3B	microtubule associated protein 1 light chain 3 beta	Cytoplasm	other		1,654	1.65×10^-03
ENSG00000095015.5	MAP3K1	mitogen-activated protein kinase kinase kinase 1	Cytoplasm	kinase	SM1-71, E 6201	1,036	2.33×10^-02
ENSG00000130758.7	MAP3K10	mitogen-activated protein kinase kinase kinase 10	Cytoplasm	kinase		-3,62	2.64×10^-02
ENSG00000006062.13	MAP3K14	mitogen-activated protein kinase kinase kinase 14	Cytoplasm	kinase		-2,508	3.40×10^-02
ENSG00000135341.17	MAP3K7	mitogen-activated protein kinase kinase kinase 7	Cytoplasm	kinase	takinib	1,472	3.78×10^-02
ENSG00000215179.5	MAPK6P4	mitogen-activated protein kinase 6 pseudogene 4	Other	other		1,253	1.32×10^-02
ENSG00000107643.15	MAPK8	mitogen-activated protein kinase 8	Cytoplasm	kinase	JNK-IN-8, cobimetinib/encora	1,066	2.74×10^-02
ENSG00000008735.13	MAPK8IP2	mitogen-activated protein kinase 8 interacting protein 2	Cytoplasm	other		-1,237	2.35×10^-02
ENSG00000138834.12	MAPK8IP3	mitogen-activated protein kinase 8 interacting protein 3	Cytoplasm	other		-1,324	9.52×10^-03
ENSG00000175130.6	MARCKSL1	MARCKS like 1	Cytoplasm	other		1,291	1.21×10^-02
ENSG00000162510.5	MATN1	matrilin 1	Extracellular Space	other		-1,469	1.88×10^-02
ENSG00000015479.17	MATR3	matrin 3	Nucleus	other		1,5	1.12×10^-05
ENSG00000103495.13	MAZ	MYC associated zinc finger protein	Nucleus	transcription regulator		6,034	3.50×10^-14
ENSG00000071655.17	MBD3	methyl-CpG binding domain protein 3	Nucleus	other		-2,111	4.87×10^-09
ENSG00000129071.9	MBD4	methyl-CpG binding domain 4, DNA glycosylase	Nucleus	enzyme		1,584	4.57×10^-02
ENSG00000229619.3	MBNL1-AS1	MBNL1 antisense RNA 1	Other	other		1,31	1.71×10^-07
ENSG00000076770.14	MBNL3	muscleblind like splicing regulator 3	Nucleus	other		1,172	3.57×10^-02
ENSG00000012174.11	MBTPS2	membrane bound transcription factor peptidase, site 2	Cytoplasm	peptidase		1,336	4.48×10^-02
ENSG00000076003.4	MCM6	minichromosome maintenance complex component 6	Nucleus	enzyme		1,78	3.51×10^-02
ENSG00000178460.17	MCMDC2	minichromosome maintenance domain containing 2	Other	other		1,199	1.72×10^-02
ENSG00000065833.8	ME1	malic enzyme 1	Cytoplasm	enzyme		2,005	1.47×10^-02
ENSG00000108510.9	MED13	mediator complex subunit 13	Nucleus	transcription regulator		1,084	2.85×10^-02
ENSG00000180182.10	MED14	mediator complex subunit 14	Nucleus	transcription regulator		1,885	3.43×10^-04
ENSG00000229431.1	MED8-AS1		Other	other		1,596	4.77×10^-02
ENSG00000068305.17	MEF2A	myocyte enhancer factor 2A	Nucleus	transcription regulator		1,271	2.93×10^-02
ENSG00000105429.12	MEGF8	multiple EGF like domains 8	Extracellular Space	other		-2,206	6.50×10^-05
ENSG00000197889.9	MEIG1	meiosis/spermiogenesis associated 1	Nucleus	other		-3,816	2.58×10^-02
ENSG00000164024.11	METAP1	methionyl aminopeptidase 1	Cytoplasm	peptidase		1,511	2.73×10^-02
ENSG00000111142.13	METAP2	methionyl aminopeptidase 2	Cytoplasm	peptidase	M8891, nitroxoline, beloranib	1,53	4.29×10^-05
ENSG00000103260.8	METRN	meteorin, glial cell differentiation regulator	Extracellular Space	other		1,627	5.48×10^-07
ENSG00000254726.2	MEX3A	mex-3 RNA binding family member A	Cytoplasm	other		-1,153	1.35×10^-02
ENSG00000111341.9	MGP	matrix Gla protein	Extracellular Space	other		1,253	3.10×10^-02
ENSG00000261857.6	MIA	MIA SH3 domain containing	Extracellular Space	other		2,794	3.37×10^-02
ENSG00000240972.1	MIF	macrophage migration inhibitory factor	Extracellular Space	cytokine	imalumab	1,774	2.66×10^-02
ENSG00000233006.6	MIR3936HG	MIR3936 host gene	Other	other		2,138	3.75×10^-02
ENSG00000224141.5	MIR548XHG	MIR548X host gene	Other	other		3,761	3.40×10^-13
ENSG00000227195.8	MIR663AHG	MIR663A host gene	Other	other		2,507	5.87×10^-07
ENSG00000196611.4	MMP1	matrix metallopeptidase 1	Extracellular Space	peptidase	rebimastat, marimastat	4,395	1.75×10^-14
ENSG00000087245.12	MMP2	matrix metallopeptidase 2	Extracellular Space	peptidase	prinomastat, rebimastat, mar	1,822	5.55×10^-04
ENSG00000166391.14	MOGAT2	monoacylglycerol O-acyltransferase 2	Cytoplasm	enzyme		-1,452	6.00×10^-03
ENSG00000123562.16	MORF4L2	mortality factor 4 like 2	Nucleus	other		3,119	7.80×10^-05
ENSG00000172938.3	MRGPRD	MAS related GPR family member D	Plasma Membrane	G-protein coupled receptor		-3,646	2.37×10^-02
ENSG00000182170.3	MRGPRG	MAS related GPR family member G	Plasma Membrane	G-protein coupled receptor		-3,718	3.08×10^-02
ENSG00000254529.1	MRGPRX7P	MAS related GPR family member X7, pseudogene	Other	other		-1,59	1.70×10^-02
ENSG00000169288.17	MRPL1	mitochondrial ribosomal protein L1	Cytoplasm	other		3,59	1.21×10^-02
ENSG00000172172.7	MRPL13	mitochondrial ribosomal protein L13	Cytoplasm	other		1,54	2.41×10^-02
ENSG00000112110.9	MRPL18	mitochondrial ribosomal protein L18	Cytoplasm	translation regulator		2,081	1.15×10^-02
ENSG00000224870.6	MRPL20-AS1	MRPL20 antisense RNA 1	Other	other		-2,664	2.23×10^-02
ENSG00000005302.17	MSL3	MSL complex subunit 3	Nucleus	transcription regulator		1,934	8.43×10^-06
ENSG00000147065.16	MSN	moesin	Plasma Membrane	other		2,136	8.56×10^-17
ENSG00000166343.9	MSS51	MSS51 mitochondrial translational activator	Other	enzyme		1,994	4.57×10^-02
ENSG00000198727.2	MT-CYB	cytochrome b	Cytoplasm	enzyme	atovaquone, atovaquone/pro	-1,169	4.26×10^-08
ENSG00000198763.3	MT-ND2	NADH dehydrogenase subunit 2	Cytoplasm	enzyme		-1,38	1.14×10^-10
ENSG00000211459.2	MT-RNR1	s-rRNA	Cytoplasm	other		3,74	3.49×10^-35
ENSG00000210082.2	MT-RNR2	l-rRNA	Cytoplasm	other		1,783	2.35×10^-05
ENSG00000210194.1	MT-TE	tRNA	Cytoplasm	other		3,327	2.21×10^-04
ENSG00000210077.1	MT-TV	tRNA	Cytoplasm	other		2,753	7.82×10^-03
ENSG00000169715.14	MT1E	metallothionein 1E	Other	other		4,15	4.07×10^-05
ENSG00000125148.6	MT2A	metallothionein 2A	Cytoplasm	other		3,788	1.54×10^-24
ENSG00000248527.1	MTATP6P1	MT-ATP6 pseudogene 1	Other	other		-1,096	4.43×10^-07
ENSG00000172167.7	MTBP	MDM2 binding protein	Cytoplasm	other		1,397	1.13×10^-02
ENSG00000229080.1	MTCO3P30		Other	other		3,485	1.62×10^-02
ENSG00000226579.1	MTCYBP31		Other	other		2,069	4.38×10^-02
ENSG00000226414.1	MTCYBP38		Other	other		2,074	1.41×10^-02
ENSG00000063601.16	MTMR1	myotubularin related protein 1	Cytoplasm	phosphatase		1,782	8.38×10^-04
ENSG00000227225.1	MTND1P14	MT-ND1 pseudogene 14	Other	other		1,507	4.65×10^-02
ENSG00000264168.1	MTND1P15	MT-ND1 pseudogene 15	Other	other		2,04	8.24×10^-03
ENSG00000243658.1	MTND5P16	MT-ND5 pseudogene 16	Other	other		1,84	8.34×10^-03
ENSG00000261904.1	MTND5P33		Other	other		-1,217	3.06×10^-02
ENSG00000256045.2	MTRNR2L10	MT-RNR2 like 10	Other	other		2,85	3.38×10^-03
ENSG00000269028.3	MTRNR2L12	MT-RNR2 like 12	Other	other		1,745	3.80×10^-02
ENSG00000255823.2	MTRNR2L8	MT-RNR2 like 8	Other	other		1,812	4.82×10^-02
ENSG00000170873.18	MTSS1	MTSS I-BAR domain containing 1	Cytoplasm	other		1,865	3.34×10^-07
ENSG00000129422.13	MTUS1	microtubule associated scaffold protein 1	Extracellular Space	other		1,016	1.94×10^-03
ENSG00000198788.8	MUC2	mucin 2, oligomeric mucus/gel-forming	Extracellular Space	other		-1,498	1.63×10^-07
ENSG00000213347.10	MXD3	MAX dimerization protein 3	Nucleus	transcription regulator		-2,095	1.04×10^-02
ENSG00000105357.15	MYH14	myosin heavy chain 14	Extracellular Space	enzyme		-1,193	4.89×10^-04
ENSG00000100345.20	MYH9	myosin heavy chain 9	Cytoplasm	enzyme		1,451	5.51×10^-11
ENSG00000101608.12	MYL12A	myosin light chain 12A	Cytoplasm	other		1,766	2.47×10^-05
ENSG00000118680.12	MYL12B	myosin light chain 12B	Cytoplasm	other		2,6	3.93×10^-07
ENSG00000092841.18	MYL6	myosin light chain 6	Cytoplasm	enzyme		1,232	4.82×10^-06
ENSG00000101335.9	MYL9	myosin light chain 9	Cytoplasm	other		-1,891	1.40×10^-02
ENSG00000145949.9	MYLK4	myosin light chain kinase family member 4	Cytoplasm	kinase		1,095	2.76×10^-02
ENSG00000137474.19	MYO7A	myosin VIIA	Cytoplasm	enzyme		-1,486	2.33×10^-02
ENSG00000196132.11	MYT1	myelin transcription factor 1	Nucleus	transcription regulator		-1,006	2.81×10^-02
ENSG00000244754.8	N4BP2L2	NEDD4 binding protein 2 like 2	Nucleus	transcription regulator		3,189	2.31×10^-45
ENSG00000152620.12	NADK2	NAD kinase 2, mitochondrial	Cytoplasm	kinase		1,737	4.03×10^-02
ENSG00000105835.11	NAMPT	nicotinamide phosphoribosyltransferase	Extracellular Space	cytokine	daporinad, KPT-9274	1,11	1.64×10^-02
ENSG00000187109.13	NAP1L1	nucleosome assembly protein 1 like 1	Nucleus	other		1,82	4.38×10^-16
ENSG00000204272.10	NBDY	negative regulator of P-body association	Other	other		5,23	1.41×10^-03
ENSG00000104320.13	NBN	nibrin	Nucleus	other		1,241	3.93×10^-02
ENSG00000115053.15	NCL	nucleolin	Nucleus	other	AGRO 100	1,792	4.24×10^-20
ENSG00000196498.13	NCOR2	nuclear receptor corepressor 2	Nucleus	transcription regulator		1,905	2.16×10^-17
ENSG00000107130.9	NCS1	neuronal calcium sensor 1	Plasma Membrane	other		-1,518	2.18×10^-02
ENSG00000174886.12	NDUFA11	NADH:ubiquinone oxidoreductase subunit A11	Cytoplasm	enzyme		-1,568	4.00×10^-02
ENSG00000168653.10	NDUFS5	NADH:ubiquinone oxidoreductase subunit S5	Cytoplasm	enzyme		2,431	5.46×10^-04
ENSG00000100285.9	NEFH	neurofilament heavy	Cytoplasm	other		-1,297	2.93×10^-03
ENSG00000223489.1	NEFHP1	neurofilament heavy pseudogene 1	Other	other		-1,147	7.35×10^-03
ENSG00000173848.18	NET1	neuroepithelial cell transforming 1	Nucleus	other		1,077	2.07×10^-02
ENSG00000204099.11	NEU4	neuraminidase 4	Cytoplasm	enzyme		-2,242	2.19×10^-02
ENSG00000188158.14	NHS	NHS actin remodeling regulator	Nucleus	other		1,563	1.25×10^-06
ENSG00000010322.15	NISCH	nischarin	Plasma Membrane	other	moxonidine, agmatine	-1,333	3.25×10^-03
ENSG00000179846.8	NKPD1	NTPase KAP family P-loop domain containing 1	Other	other		-2,038	1.80×10^-02
ENSG00000183072.9	NKX2-5	NK2 homeobox 5	Nucleus	transcription regulator		2,17	5.36×10^-04
ENSG00000148826.8	NKX6-2	NK6 homeobox 2	Nucleus	transcription regulator		-2,806	4.05×10^-02
ENSG00000259167.2	NMNAT1P1	NMNAT1 pseudogene 1	Other	other		2,608	4.77×10^-02
ENSG00000147140.15	NONO	non-POU domain containing octamer binding	Nucleus	transcription regulator		1,93	3.69×10^-08
ENSG00000149308.16	NPAT	nuclear protein, coactivator of histone transcription	Nucleus	transcription regulator		1,169	3.86×10^-02
ENSG00000196796.5	NPIPB10P	nuclear pore complex interacting protein family, member B10, pseudogene	Other	other		-4,275	5.19×10^-03
ENSG00000181163.13	NPM1	nucleophosmin 1	Nucleus	transcription regulator		3,071	1.87×10^-36
ENSG00000221890.2	NPTXR	neuronal pentraxin receptor	Plasma Membrane	transmembrane receptor		-2,258	3.67×10^-04
ENSG00000137404.14	NRM	nurim	Nucleus	other		-1,686	4.79×10^-02
ENSG00000110076.18	NRXN2	neurexin 2	Plasma Membrane	transporter		-1,501	8.35×10^-04
ENSG00000223705.9	NSUN5P1	NSUN5 pseudogene 1	Other	other		-1,092	1.47×10^-02
ENSG00000256355.1	NTAN1P3	N-terminal asparagine amidase pseudogene 3	Other	other		3,489	2.40×10^-02
ENSG00000069275.12	NUCKS1	nuclear casein kinase and cyclin dependent kinase substrate 1	Nucleus	kinase		1,152	3.07×10^-04
ENSG00000196368.4	NUDT11	nudix hydrolase 11	Cytoplasm	phosphatase		1,372	4.41×10^-02
ENSG00000143552.9	NUP210L	nucleoporin 210 like	Other	other		2,188	6.94×10^-04
ENSG00000184923.12	NUTM2A/NUTM2B	NUT family member 2A	Other	other		-2,26	2.32×10^-02
ENSG00000205978.5	NYNRIN	NYN domain and retroviral integrase containing	Other	other		-1,111	1.22×10^-02
ENSG00000149635.2	OCSTAMP	osteoclast stimulatory transmembrane protein	Other	other		1,655	3.26×10^-02
ENSG00000105479.15	ODAD1	outer dynein arm docking complex subunit 1	Extracellular Space	other		-1,093	2.86×10^-02
ENSG00000257135.5	ODC1-DT	ODC1 divergent transcript	Other	other		3,533	9.83×10^-08
ENSG00000122417.15	ODF2L	outer dense fiber of sperm tails 2 like	Cytoplasm	other		1,157	4.68×10^-02
ENSG00000060491.16	OGFR	opioid growth factor receptor	Plasma Membrane	other	enkephalin, methionine	-2,802	4.96×10^-04
ENSG00000185585.19	OLFML2A	olfactomedin like 2A	Extracellular Space	other		-1,604	9.00×10^-03
ENSG00000125741.4	OPA3	outer mitochondrial membrane lipid metabolism regulator OPA3	Cytoplasm	other		-1,214	2.70×10^-04
ENSG00000254403.1	OR10Y1P	olfactory receptor family 10 subfamily Y member 1 pseudogene	Other	other		-2,485	3.97×10^-02
ENSG00000262628.1	OR1D5	olfactory receptor family 1 subfamily D member 5	Plasma Membrane	G-protein coupled receptor		-1,809	1.16×10^-02
ENSG00000185372.2	OR2V1	olfactory receptor family 2 subfamily V member 1	Plasma Membrane	G-protein coupled receptor		-2,344	3.50×10^-02
ENSG00000184789.6	OR4C10P	olfactory receptor family 4 subfamily C member 10 pseudogene	Other	other		2,632	2.13×10^-02
ENSG00000233301.1	OR4C14P	olfactory receptor family 4 subfamily C member 14 pseudogene	Other	other		-3,32	2.83×10^-02
ENSG00000217874.2	OR4F7P	olfactory receptor family 4 subfamily F member 7 pseudogene	Other	other		-4,572	1.56×10^-03
ENSG00000240361.1	OR4G11P	olfactory receptor family 4 subfamily G member 11 pseudogene	Other	other		2,442	4.99×10^-02
ENSG00000282137.1	OR4G3P	olfactory receptor family 4 subfamily G member 3 pseudogene	Other	other		-3,789	1.47×10^-02
ENSG00000155249.4	OR4K1	olfactory receptor family 4 subfamily K member 1	Plasma Membrane	G-protein coupled receptor		1,922	1.42×10^-02

ENSG00000254490.1	OR5M7P	olfactory receptor family 5 subfamily M member 7 pseudogene	Other	other		1,751	4.23×10^-02
ENSG00000186513.2	OR9Q2	olfactory receptor family 9 subfamily Q member 2	Plasma Membrane	G-protein coupled receptor		-3,434	4.20×10^-02
ENSG00000006025.11	OSBPL7	oxysterol binding protein like 7	Cytoplasm	other		-1,4	4.06×10^-02
ENSG00000198856.12	OSTC	oligosaccharyltransferase complex non-catalytic subunit	Cytoplasm	enzyme		2,222	2.33×10^-02
ENSG00000189401.2	OTUD6A	OTU deubiquitinase 6A	Other	peptidase		2,146	4.70×10^-02
ENSG00000198754.5	OXCT2	3-oxoacid CoA-transferase 2	Cytoplasm	enzyme		-4,262	6.57×10^-03
ENSG00000164830.17	OXR1	oxidation resistance 1	Cytoplasm	enzyme		1,022	2.61×10^-02
ENSG00000185624.14	P4HB	prolyl 4-hydroxylase subunit beta	Cytoplasm	enzyme		2,39	4.72×10^-20
ENSG00000070756.13	PABPC1	poly(A) binding protein cytoplasmic 1	Cytoplasm	translation regulator		2,637	2.61×10^-30
ENSG00000225638.1	PABPC1P12	poly(A) binding protein cytoplasmic 1 pseudogene 12	Other	other		1,131	4.08×10^-02
ENSG00000250651.1	PABPC1P7	poly(A) binding protein cytoplasmic 1 pseudogene 7	Other	other		2,22	3.59×10^-02
ENSG00000228755.1	PABPC1P8	poly(A) binding protein cytoplasmic 1 pseudogene 8	Other	other		-1,378	4.56×10^-02
ENSG00000124507.10	PACSIN1	protein kinase C and casein kinase substrate in neurons 1	Cytoplasm	kinase		-1,534	1.89×10^-02
ENSG00000223973.2	PAFAH1B1P2	platelet activating factor acetylhydrolase 1b regulatory subunit 1 pseudogene 2	Other	other		-3,341	4.89×10^-02
ENSG00000168092.13	PAFAH1B2	platelet activating factor acetylhydrolase 1b catalytic subunit 2	Cytoplasm	enzyme		1,14	2.95×10^-03
ENSG00000107719.8	PALD1	phosphatase domain containing paladin 1	Cytoplasm	phosphatase		-2,073	4.34×10^-02
ENSG00000187867.8	PALM3	paralemmin 3	Other	other		-1,296	5.84×10^-06
ENSG00000115421.12	PAPOLG	poly(A) polymerase gamma	Nucleus	enzyme		1,166	3.72×10^-02
ENSG00000257243.1	PARK7P1	PARK7 pseudogene 1	Other	other		3,067	1.42×10^-02
ENSG00000197111.15	PCBP2	poly(rC) binding protein 2	Nucleus	other		1,702	8.62×10^-08
ENSG00000102290.21	PCDH11X	protocadherin 11 X-linked	Plasma Membrane	other		1,333	3.66×10^-03
ENSG00000099715.14	PCDH11Y	protocadherin 11 Y-linked	Other	other		-2,161	1.73×10^-02
ENSG00000113212.6	PCDHB7	protocadherin beta 7	Plasma Membrane	other		1,245	8.39×10^-05
ENSG00000120322.3	PCDHB8	protocadherin beta 8	Plasma Membrane	other		1,608	8.72×10^-07
ENSG00000168300.13	PCMTD1	protein-L-isoaspartate (D-aspartate) O-methyltransferase domain containing 1	Cytoplasm	enzyme		1,363	1.27×10^-02
ENSG00000155660.10	PDIA4	protein disulfide isomerase family A member 4	Cytoplasm	enzyme		1,911	6.55×10^-03
ENSG00000172367.15	PDZD3	PDZ domain containing 3	Plasma Membrane	transporter		-1,822	2.78×10^-03
ENSG00000261371.5	PECAM1	platelet and endothelial cell adhesion molecule 1	Plasma Membrane	other		1,307	1.29×10^-05
ENSG00000282164.1	PEG13	paternally expressed 13	Other	other		-1,135	1.80×10^-02
ENSG00000197329.11	PELI1	pellino E3 ubiquitin protein ligase 1	Cytoplasm	enzyme		1,195	2.78×10^-02
ENSG00000179094.13	PER1	period circadian regulator 1	Nucleus	transcription regulator	avibactam	-1,409	1.80×10^-02
ENSG00000248191.1	PES1P1	pescadillo ribosomal biogenesis factor 1 pseudogene 1	Other	other		-1,725	2.80×10^-03
ENSG00000139197.10	PEX5	peroxisomal biogenesis factor 5	Cytoplasm	other		-1,865	2.03×10^-02
ENSG00000178921.13	PFAS	phosphoribosylformylglycinamidine synthase	Cytoplasm	enzyme		-1,788	2.04×10^-02
ENSG00000123349.13	PFDN5	prefoldin subunit 5	Nucleus	transcription regulator		1,14	8.77×10^-03
ENSG00000102144.13	PGK1	phosphoglycerate kinase 1	Cytoplasm	kinase	CBR-470-1	1,743	3.17×10^-05
ENSG00000164902.13	PHAX	phosphorylated adaptor for RNA export	Cytoplasm	other		1,285	1.60×10^-02
ENSG00000122733.12	PHF24	PHD finger protein 24	Other	other		-1,362	3.91×10^-02
ENSG00000143393.16	PI4KB	phosphatidylinositol 4-kinase beta	Cytoplasm	kinase	STF-1019, viroxime, PIK-93	-1,189	1.79×10^-02
ENSG00000073921.17	PICALM	phosphatidylinositol binding clathrin assembly protein	Cytoplasm	other		2,041	9.84×10^-03
ENSG00000165195.13	PIGA	phosphatidylinositol glycan anchor biosynthesis class A	Cytoplasm	enzyme		1,797	2.33×10^-03
ENSG00000051382.8	PIK3CB	phosphatidylinositol-4,5-bisphosphate 3-kinase catalytic subunit beta	Cytoplasm	kinase	TGX-221, AZD8186, GNE-49	1,553	1.28×10^-03
ENSG00000141506.13	PIK3R5	phosphoinositide-3-kinase regulatory subunit 5	Cytoplasm	kinase		-1,688	2.59×10^-03
ENSG00000102309.12	PIN4	peptidylprolyl cis/trans isomerase, NIMA-interacting 4	Nucleus	enzyme		1,375	3.78×10^-02
ENSG00000198961.9	PJA2	praja ring finger ubiquitin ligase 2	Cytoplasm	enzyme		2,006	4.87×10^-03
ENSG00000165495.15	PKNOX2	PBX/knotted 1 homeobox 2	Nucleus	transcription regulator		-1,059	7.50×10^-03
ENSG00000153246.11	PLA2R1	phospholipase A2 receptor 1	Plasma Membrane	transmembrane receptor		1,316	4.79×10^-02
ENSG00000196155.12	PLEKHG4	pleckstrin homology and RhoGEF domain containing G4	Cytoplasm	other		-2,033	3.70×10^-02
ENSG00000102007.10	PLP2	proteolipid protein 2	Cytoplasm	transporter		2,426	1.54×10^-02
ENSG00000102024.17	PLS3	plastin 3	Cytoplasm	other		1,995	2.11×10^-03
ENSG00000141682.11	PMAIP1	phorbol-12-myristate-13-acetate-induced protein 1	Cytoplasm	other		1,985	2.72×10^-02
ENSG00000147669.10	POLR2K	RNA polymerase II, I and III subunit K	Nucleus	enzyme		2,882	5.46×10^-03
ENSG00000205578.5	POM121B	POM121 transmembrane nucleoporin B (pseudogene)	Other	other		-3,794	2.36×10^-02
ENSG00000221900.5	POM121L12	POM121 transmembrane nucleoporin like 12	Other	other		4,535	2.27×10^-76
ENSG00000239511.2	POM121L7P	POM121 transmembrane nucleoporin like 7 pseudogene	Other	other		-3,866	2.73×10^-02
ENSG00000236870.2	POMPP1	POMP pseudogene 1	Other	other		5,632	1.89×10^-03
ENSG00000177380.13	PPFIA3	PTPRF interacting protein alpha 3	Plasma Membrane	phosphatase		-1,09	3.97×10^-02
ENSG00000196262.13	PPIA	peptidylprolyl isomerase A	Cytoplasm	enzyme	cyclosporin A/methotrexate,	1,607	4.28×10^-06
ENSG00000228979.4	PPIAP55	peptidylprolyl isomerase A pseudogene 55	Other	other		3,634	4.66×10^-02
ENSG00000168781.21	PPIP5K1	diphosphoinositol pentakisphosphate kinase 1	Nucleus	phosphatase		-1,141	2.71×10^-02
ENSG00000087074.7	PPP1R15A	protein phosphatase 1 regulatory subunit 15A	Cytoplasm	other		-1,006	2.74×10^-02
ENSG00000131771.13	PPP1R1B	protein phosphatase 1 regulatory inhibitor subunit 1B	Cytoplasm	phosphatase		-1,459	1.94×10^-02
ENSG00000273643.1	PPP1R26P2	protein phosphatase 1 regulatory subunit 26 pseudogene 2	Other	other		-1,799	9.84×10^-03
ENSG00000104866.10	PPP1R37	protein phosphatase 1 regulatory subunit 37	Other	other		-1,2	3.36×10^-02
ENSG00000113575.9	PPP2CA	protein phosphatase 2 catalytic subunit alpha	Cytoplasm	phosphatase		1,363	6.47×10^-03
ENSG00000175470.18	PPP2R2D	protein phosphatase 2 regulatory subunit Bdelta	Nucleus	other		-1,207	2.47×10^-05
ENSG00000224960.3	PPP4R3C	protein phosphatase 4 regulatory subunit 3C	Other	other		1,903	4.24×10^-02
ENSG00000105063.18	PPP6R1	protein phosphatase 6 regulatory subunit 1	Cytoplasm	other		-1,703	8.75×10^-03
ENSG00000142611.16	PRDM16	PR/SET domain 16	Nucleus	transcription regulator		-1,462	1.32×10^-02
ENSG00000117450.13	PRDX1	peroxiredoxin 1	Cytoplasm	enzyme		2,863	1.59×10^-02
ENSG00000126432.13	PRDX5	peroxiredoxin 5	Cytoplasm	enzyme	auranofin	2,482	1.03×10^-03
ENSG00000005249.12	PRKAR2B	protein kinase cAMP-dependent type II regulatory subunit beta	Cytoplasm	kinase		3,234	1.15×10^-07
ENSG00000115825.9	PRKD3	protein kinase D3	Nucleus	kinase	SM1-71, kb-NB 142-70	1,168	9.70×10^-04
ENSG00000155066.15	PROM2	prominin 2	Plasma Membrane	transmembrane receptor		-1,133	2.06×10^-02
ENSG00000196504.15	PRPF40A	pre-mRNA processing factor 40 homolog A	Nucleus	other		1,41	3.30×10^-04
ENSG00000135406.13	PRPH	peripherin	Plasma Membrane	other		-3,318	2.65×10^-02
ENSG00000183248.11	PRR36	proline rich 36	Other	other		-1,45	2.45×10^-03
ENSG00000130032.15	PRRG3	proline rich and Gla domain 3	Plasma Membrane	other		1,172	5.45×10^-04
ENSG00000188086.12	PRSS45P	serine protease 45, pseudogene	Other	peptidase		-3,235	9.35×10^-03
ENSG00000101182.14	PSMA7	proteasome 20S subunit alpha 7	Cytoplasm	peptidase		2,288	7.84×10^-03
ENSG00000161057.10	PSMC2	proteasome 26S subunit, ATPase 2	Nucleus	peptidase		1,944	1.16×10^-03
ENSG00000095261.13	PSMD5	proteasome 26S subunit, non-ATPase 5	Cytoplasm	other		1,601	1.49×10^-02
ENSG00000103035.10	PSMD7	proteasome 26S subunit, non-ATPase 7	Cytoplasm	other		2,078	1.05×10^-03
ENSG00000128789.20	PSMG2	proteasome assembly chaperone 2	Nucleus	other		2,121	2.33×10^-05
ENSG00000187514.14	PTMA	prothymosin alpha	Nucleus	other		1,77	5.42×10^-14
ENSG00000104960.15	PTOV1	PTOV1 extended AT-hook containing adaptor protein	Nucleus	other		-2,531	1.47×10^-02
ENSG00000112245.9	PTP4A1	protein tyrosine phosphatase 4A1	Cytoplasm	phosphatase		1,386	2.12×10^-02
ENSG00000184007.17	PTP4A2	protein tyrosine phosphatase 4A2	Cytoplasm	phosphatase		1,506	1.29×10^-04
ENSG00000179295.15	PTPN11	protein tyrosine phosphatase non-receptor type 11	Cytoplasm	phosphatase	BBP-398, ERAS-601, PF-07	1,751	2.40×10^-07
ENSG00000134242.15	PTPN22	protein tyrosine phosphatase non-receptor type 22	Cytoplasm	phosphatase		2,686	8.95×10^-03
ENSG00000054356.13	PTPRN	protein tyrosine phosphatase receptor type N	Plasma Membrane	phosphatase		-1,961	2.50×10^-04
ENSG00000147485.12	PXDNL	peroxidasin like	Extracellular Space	enzyme		1,372	4.26×10^-02
ENSG00000145337.4	PYURF	PIGY upstream reading frame	Cytoplasm	other		2,914	3.91×10^-02
ENSG00000206418.3	RAB12	RAB12, member RAS oncogene family	Cytoplasm	enzyme		2,473	3.53×10^-02
ENSG00000143545.8	RAB13	RAB13, member RAS oncogene family	Plasma Membrane	enzyme		1,849	6.24×10^-16
ENSG00000139998.14	RAB15	RAB15, member RAS oncogene family	Cytoplasm	other		-2,018	1.02×10^-03
ENSG00000102128.7	RAB40AL	RAB40A like	Plasma Membrane	other		1,912	3.65×10^-02
ENSG00000075785.12	RAB7A	RAB7A, member RAS oncogene family	Cytoplasm	enzyme		1,414	4.91×10^-03
ENSG00000167461.11	RAB8A	RAB8A, member RAS oncogene family	Plasma Membrane	enzyme		-2,391	9.84×10^-03
ENSG00000166128.12	RAB8B	RAB8B, member RAS oncogene family	Cytoplasm	enzyme		1,55	4.34×10^-02
ENSG00000204628.11	RACK1	receptor for activated C kinase 1	Cytoplasm	enzyme		2,174	1.23×10^-14
ENSG00000002016.16	RAD52	RAD52 homolog, DNA repair protein	Nucleus	other		1,509	3.20×10^-02
ENSG00000157927.16	RADIL	Rap associating with DIL domain	Cytoplasm	other		-1,907	8.10×10^-03
ENSG00000039560.13	RAI14	retinoic acid induced 14	Nucleus	transcription regulator		1,1	4.66×10^-03
ENSG00000006451.7	RALA	RAS like proto-oncogene A	Cytoplasm	enzyme		2,603	4.44×10^-10
ENSG00000144118.13	RALB	RAS like proto-oncogene B	Cytoplasm	enzyme		1,244	1.65×10^-03
ENSG00000153201.15	RANBP2	RAN binding protein 2	Nucleus	enzyme		1,207	1.50×10^-05
ENSG00000171777.15	RASGRP4	RAS guanyl releasing protein 4	Cytoplasm	other		-1,496	1.32×10^-02
ENSG00000153179.11	RASSF3	Ras association domain family member 3	Other	other		1,331	1.27×10^-03
ENSG00000103479.14	RBL2	RB transcriptional corepressor like 2	Nucleus	other		1,201	1.69×10^-02
ENSG00000089682.16	RBM41	RNA binding motif protein 41	Other	other		1,233	1.61×10^-02
ENSG00000127993.14	RBM48	RNA binding motif protein 48	Nucleus	other		1,537	1.90×10^-02
ENSG00000147274.14	RBMX	RNA binding motif protein X-linked	Nucleus	other		1,425	2.12×10^-04
ENSG00000134597.13	RBMX2	RNA binding motif protein X-linked 2	Nucleus	other		2,149	4.51×10^-02
ENSG00000170748.6	RBMXL2	RBMX like 2	Nucleus	other		1,365	4.34×10^-02
ENSG00000049449.8	RCN1	reticulocalbin 1	Cytoplasm	other		1,413	2.59×10^-03
ENSG00000137710.14	RDX	radixin	Cytoplasm	other		1,443	3.13×10^-05
ENSG00000172023.7	REG1B	regenerating family member 1 beta	Extracellular Space	other		1,699	1.77×10^-02
ENSG00000169891.17	REPS2	RALBP1 associated Eps domain containing 2	Cytoplasm	other		1,053	7.44×10^-03
ENSG00000163515.6	RETNLB	resistin like beta	Extracellular Space	other		-3,438	2.65×10^-02
ENSG00000276998.1	REXO1L2P	REXO1 like 2, pseudogene	Other	other		-6,753	1.18×10^-09
ENSG00000277422.1	REXO1L6P	REXO1 like 6, pseudogene	Other	other		-1,202	1.63×10^-08
ENSG00000225465.8	RFPL1S		Other	other		-1,406	4.27×10^-03
ENSG00000143390.17	RFX5	regulatory factor X5	Nucleus	transcription regulator		3,176	2.76×10^-23
ENSG00000169629.11	RGPD4 (includes others)	RANBP2 like and GRIP domain containing 5	Nucleus	enzyme		1,083	4.14×10^-02
ENSG00000169220.17	RGS14	regulator of G protein signaling 14	Cytoplasm	enzyme		-2,471	6.90×10^-03
ENSG00000103269.13	RHBDL1	rhomboid like 1	Plasma Membrane	peptidase		3,538	1.10×10^-02
ENSG00000187010.18	RHCE/RHD	Rh blood group D antigen	Plasma Membrane	transporter		1,882	4.85×10^-02
ENSG00000067560.10	RHOA	ras homolog family member A	Cytoplasm	enzyme		2,512	3.68×10^-10
ENSG00000143878.9	RHOB	ras homolog family member B	Cytoplasm	enzyme		1,546	1.62×10^-02
ENSG00000008853.16	RHOBTB2	Rho related BTB domain containing 2	Cytoplasm	enzyme		-1,136	5.77×10^-03
ENSG00000126785.12	RHOJ	ras homolog family member J	Cytoplasm	enzyme		1,46	4.64×10^-05
ENSG00000150977.10	RILPL2	Rab interacting lysosomal protein like 2	Cytoplasm	other		-1,6	1.14×10^-04
ENSG00000275793.1	RIMBP3 (includes others)	RIMS binding protein 3	Nucleus	other		-1,725	8.62×10^-03
ENSG00000101098.12	RIMS4	regulating synaptic membrane exocytosis 4	Plasma Membrane	other		-1,67	3.50×10^-02
ENSG00000117000.8	RLF	RLF zinc finger	Nucleus	transcription regulator		1,136	4.57×10^-03
ENSG00000131263.12	RLIM	ring finger protein, LIM domain interacting	Nucleus	enzyme		1,473	1.54×10^-03
ENSG00000153561.12	RMND5A	required for meiotic nuclear division 5 homolog A	Nucleus	enzyme		1,498	1.19×10^-03

ENSG00000269900.3	RMRP	RNA component of mitochondrial RNA processing endoribonuclease	Cytoplasm	other		5,018	5.17×10^-36
ENSG00000276168.1	RN7SL1	RNA component of signal recognition particle 7SL1	Other	other		2,121	6.91×10^-15
ENSG00000274012.1	RN7SL2	RNA component of signal recognition particle 7SL2	Other	other		1,864	1.14×10^-10
ENSG00000278771.1	RN7SL3	RNA component of signal recognition particle 7SL3	Other	other		2,056	2.12×10^-15
ENSG00000263426.2	RN7SL471P		Other	other		3,216	1.01×10^-02
ENSG00000263740.2	RN7SL4P	RNA, 7SL, cytoplasmic 4, pseudogene	Other	other		1,442	1.37×10^-05
ENSG00000265735.2	RN7SL5P	RNA, 7SL, cytoplasmic 5, pseudogene	Other	other		3,309	2.83×10^-04
ENSG00000277739.1	RNA5-8S5	RNA, 5.8S ribosomal 5	Other	other		6,108	2.33×10^-05
ENSG00000275757.1	RNA5-8SN1	RNA, 5.8S ribosomal N1	Other	other		4,887	2.51×10^-21
ENSG00000278189.1	RNA5-8SN1	RNA, 5.8S ribosomal N1	Other	other		4,911	2.60×10^-19
ENSG00000273730.1	RNA5-8SN2	RNA, 5.8S ribosomal N2	Other	other		4,689	1.10×10^-22
ENSG00000278233.1	RNA5-8SN2	RNA, 5.8S ribosomal N2	Other	other		6,93	1.22×10^-07
ENSG00000275215.1	RNA5-8SN3	RNA, 5.8S ribosomal N3	Other	other		6,891	1.73×10^-07
ENSG00000276700.1	RNA5-8SN4	RNA, 5.8S ribosomal N4	Other	other		6,79	7.05×10^-07
ENSG00000274917.1	RNA5-8SN5	RNA, 5.8S ribosomal N5	Other	other		6,618	2.01×10^-06
ENSG00000200434.1	RNA5-8SP2	RNA, 5.8S ribosomal pseudogene 2	Other	other		8,457	3.98×10^-28
ENSG00000251705.1	RNA5-8SP6	RNA, 5.8S ribosomal pseudogene 6	Other	other		9,277	1.37×10^-02
ENSG00000201321.1	RNA5S9	RNA, 5S ribosomal 9	Other	other		4,715	1.44×10^-56
ENSG00000201413.1	RNA5SP141	RNA, 5S ribosomal pseudogene 141	Other	other		4,568	4.94×10^-02
ENSG00000201822.1	RNA5SP149	RNA, 5S ribosomal pseudogene 149	Other	other		5,122	6.03×10^-51
ENSG00000201185.1	RNA5SP202	RNA, 5S ribosomal pseudogene 202	Other	other		11,057	8.63×10^-18
ENSG00000202386.1	RNA5SP211	RNA, 5S ribosomal pseudogene 211	Other	other		7,877	6.40×10^-12
ENSG00000200310.1	RNA5SP215	RNA, 5S ribosomal pseudogene 215	Other	other		5,501	3.22×10^-03
ENSG00000251920.1	RNA5SP216	RNA, 5S ribosomal pseudogene 216	Other	other		5,375	7.10×10^-03
ENSG00000222806.1	RNA5SP225	RNA, 5S ribosomal pseudogene 225	Other	other		5,886	2.50×10^-04
ENSG00000252261.1	RNA5SP262	RNA, 5S ribosomal pseudogene 262	Other	other		7,01	4.84×10^-08
ENSG00000200246.1	RNA5SP263		Other	other		5,88	3.71×10^-04
ENSG00000201763.1	RNA5SP267	RNA, 5S ribosomal pseudogene 267	Other	other		6,319	2.33×10^-05
ENSG00000201861.1	RNA5SP298		Other	other		4,531	6.52×10^-03
ENSG00000200225.1	RNA5SP382	RNA, 5S ribosomal pseudogene 382	Other	other		5,51	2.43×10^-03
ENSG00000222268.1	RNA5SP425	RNA, 5S ribosomal pseudogene 425	Other	other		6,111	7.23×10^-05
ENSG00000252680.1	RNA5SP449	RNA, 5S ribosomal pseudogene 449	Other	other		8,181	6.86×10^-12
ENSG00000252546.1	RNA5SP466	RNA, 5S ribosomal pseudogene 466	Other	other		5,118	2.46×10^-02
ENSG00000200839.1	RNA5SP48	RNA, 5S ribosomal pseudogene 48	Other	other		6,048	9.96×10^-05
ENSG00000251770.1	RNA5SP486	RNA, 5S ribosomal pseudogene 486	Other	other		6,992	1.57×10^-07
ENSG00000275305.1	RNA5SP528		Other	other		5,601	1.99×10^-03
ENSG00000219200.10	RNASEK	ribonuclease K	Other	peptidase		-1,999	1.27×10^-02
ENSG00000115963.13	RND3	Rho family GTPase 3	Cytoplasm	enzyme		1,281	2.18×10^-02
ENSG00000013561.17	RNF14	ring finger protein 14	Cytoplasm	transcription regulator		1,211	2.37×10^-02
ENSG00000223500.1	RNF14P4	RNF14 pseudogene 4	Other	other		-1,467	3.86×10^-02
ENSG00000173456.4	RNF26	ring finger protein 26	Cytoplasm	enzyme		-1,783	1.29×10^-02
ENSG00000092098.16	RNF31	ring finger protein 31	Cytoplasm	enzyme		1,829	2.84×10^-04
ENSG00000200340.1	RNU1-105P	RNA, U1 small nuclear 105, pseudogene	Other	other		4,407	3.73×10^-07
ENSG00000202199.1	RNU1-115P		Other	other		5,991	4.05×10^-26
ENSG00000212424.1	RNU1-119P		Other	other		3,19	2.55×10^-05
ENSG00000206702.1	RNU1-11P	RNA, U1 small nuclear 11, pseudogene	Other	other		3,357	4.17×10^-04
ENSG00000200800.1	RNU1-130P		Other	other		4,884	1.91×10^-17
ENSG00000199805.1	RNU1-134P	RNA, U1 small nuclear 134, pseudogene	Other	other		4,442	9.70×10^-44
ENSG00000206908.1	RNU1-136P	RNA, U1 small nuclear 136, pseudogene	Other	other		3,422	3.22×10^-07
ENSG00000206820.1	RNU1-138P		Other	other		3,622	3.60×10^-02
ENSG00000212609.1	RNU1-139P		Other	other		5,223	8.49×10^-95
ENSG00000201910.1	RNU1-140P		Other	other		4,7	2.68×10^-05
ENSG00000200885.1	RNU1-146P	RNA, U1 small nuclear 146, pseudogene	Other	other		4,749	1.50×10^-05
ENSG00000199629.1	RNU1-14P	RNA, U1 small nuclear 14, pseudogene	Other	other		5,131	2.01×10^-72
ENSG00000202347.1	RNU1-16P	RNA, U1 small nuclear 16, pseudogene	Other	other		2,21	4.98×10^-06
ENSG00000200176.1	RNU1-19P	RNA, U1 small nuclear 19, pseudogene	Other	other		3,862	6.28×10^-09
ENSG00000200184.1	RNU1-20P	RNA, U1 small nuclear 20, pseudogene	Other	other		5,104	4.78×10^-03
ENSG00000200197.1	RNU1-21P	RNA, U1 small nuclear 21, pseudogene	Other	other		5,216	5.19×10^-71
ENSG00000200204.1	RNU1-22P	RNA, U1 small nuclear 22, pseudogene	Other	other		5,531	6.73×10^-17
ENSG00000206596.1	RNU1-27P	RNA, U1 small nuclear 27, pseudogene	Other	other		2,997	4.52×10^-05
ENSG00000200807.1	RNU1-32P	RNA, U1 small nuclear 32, pseudogene	Other	other		4,176	3.25×10^-14
ENSG00000201119.1	RNU1-33P		Other	other		4,458	8.94×10^-63
ENSG00000206624.1	RNU1-39P	RNA, U1 small nuclear 39, pseudogene	Other	other		3,504	1.92×10^-32
ENSG00000253000.1	RNU1-45P		Other	other		2,828	9.19×10^-04
ENSG00000207154.1	RNU1-46P		Other	other		4,308	5.96×10^-09
ENSG00000206917.1	RNU1-52P		Other	other		4,846	2.12×10^-03
ENSG00000202380.1	RNU1-55P		Other	other		3,54	7.92×10^-11
ENSG00000207175.1	RNU1-67P	RNA, U1 small nuclear 67, pseudogene	Other	other		4,073	3.37×10^-02
ENSG00000212550.1	RNU1-78P	RNA, U1 small nuclear 78, pseudogene	Other	other		5,009	2.09×10^-25
ENSG00000207322.1	RNU1-89P	RNA, U1 small nuclear 89, pseudogene	Other	other		3,175	3.49×10^-08
ENSG00000239023.1	RNU1-98P		Other	other		3,826	4.12×10^-42
ENSG00000270103.3	RNU11		Other	other		2,025	4.48×10^-12
ENSG00000276027.1	RNU12	RNA, U12 small nuclear	Nucleus	other		4,829	2.84×10^-84
ENSG00000222328.1	RNU2-2P	RNA, U2 small nuclear 2, pseudogene	Nucleus	other		3,906	5.91×10^-55
ENSG00000252604.1	RNU2-44P		Other	other		3,878	2.81×10^-05
ENSG00000222426.1	RNU2-50P	RNA, U2 small nuclear 50, pseudogene	Other	other		4,764	4.53×10^-15
ENSG00000222810.1	RNU2-68P		Other	other		4,65	2.44×10^-24
ENSG00000222726.1	RNU2-7P	RNA, U2 small nuclear 7, pseudogene	Other	other		4,885	1.72×10^-02
ENSG00000202538.1	RNU4-2	RNA, U4 small nuclear 2	Other	other		4,087	1.21×10^-03
ENSG00000199568.1	RNU5A-1	RNA, U5A small nuclear 1	Other	other		5,665	1.10×10^-05
ENSG00000206863.1	RNU5A-6P	RNA, U5A small nuclear 6, pseudogene	Other	other		5,53	3.04×10^-03
ENSG00000200156.1	RNU5B-1	RNA, U5B small nuclear 1	Other	other		6,282	3.78×10^-02
ENSG00000199347.1	RNU5E-1	RNA, U5E small nuclear 1	Other	other		7,561	2.88×10^-26
ENSG00000207340.1	RNVU1-1	RNA, variant U1 small nuclear 1	Other	other		5,549	2.35×10^-16
ENSG00000207205.1	RNVU1-15	RNA, variant U1 small nuclear 15	Other	other		2,207	5.71×10^-07
ENSG00000207349.1	RNVU1-17	RNA, variant U1 small nuclear 17	Other	other		4,087	1.35×10^-05
ENSG00000275538.1	RNVU1-19	RNA, variant U1 small nuclear 19	Other	other		4,303	2.05×10^-31
ENSG00000238825.1	RNVU1-2	RNA, variant U1 small nuclear 2	Other	other		2,842	3.54×10^-02
ENSG00000199879.1	RNVU1-22		Other	other		5,842	2.00×10^-32
ENSG00000274210.1	RNVU1-27		Other	other		4,724	2.59×10^-06
ENSG00000277918.1	RNVU1-28		Other	other		1,607	7.51×10^-06
ENSG00000273768.1	RNVU1-29		Other	other		3,077	1.63×10^-02
ENSG00000278099.1	RNVU1-2A	RNA, variant U1 small nuclear 2A	Other	other		5,211	2.40×10^-39
ENSG00000270722.1	RNVU1-31	RNA, variant U1 small nuclear 31	Other	other		3,338	1.94×10^-06
ENSG00000207110.1	RNVU1-32	RNA, variant U1 small nuclear 32	Other	other		5,086	2.81×10^-11
ENSG00000201098.1	RNY1	RNA, Ro60-associated Y1	Nucleus	other		2,97	8.74×10^-07
ENSG00000202354.1	RNY3	RNA, Ro60-associated Y3	Other	other		5,438	6.24×10^-07
ENSG00000252316.1	RNY4	RNA, Ro60-associated Y4	Other	other		5,069	5.33×10^-09
ENSG00000116747.12	RO60	Ro60, Y RNA binding protein	Nucleus	other		1,077	9.38×10^-03
ENSG00000282306.1	RP11-126O22.2		Other	other		2,495	2.23×10^-04
ENSG00000282299.1	RP11-126O22.6		Other	other		2,793	1.01×10^-02
ENSG00000282697.1	RP11-126O22.7		Other	other		1,179	4.63×10^-02
ENSG00000282222.1	RP11-126O22.8		Other	other		1,657	4.82×10^-02
ENSG00000258461.5	RP11-164J13.1		Other	other		-1,498	1.80×10^-02
ENSG00000280222.1	RP11-174G17.3		Other	other		-1,044	2.08×10^-03
ENSG00000224722.3	RP11-255B23.1		Other	other		-1,538	4.01×10^-02
ENSG00000258130.7	RP11-347C12.3		Other	other		-1,351	1.49×10^-02
ENSG00000272566.1	RP11-462G22.2		Other	other		4,471	1.05×10^-02
ENSG00000261187.1	RP11_1007O242		Other	other		2,474	3.78×10^-02
ENSG00000275944.1	RP11_104J231		Other	other		-1,834	3.40×10^-02
ENSG00000279196.1	RP11_1072A33		Other	other		-1,612	1.66×10^-02
ENSG00000279166.1	RP11_107E53		Other	other		1,173	1.80×10^-02
ENSG00000251101.1	RP11_1267H101		Other	other		3,356	1.86×10^-02
ENSG00000266371.1	RP11_142O61		Other	other		-2,337	4.88×10^-02
ENSG00000261009.1	RP11_1437A85		Other	other		8,345	4.89×10^-02
ENSG00000277728.1	RP11_143K117		Other	other		5,679	2.36×10^-03
ENSG00000230759.1	RP11_153F12		Other	other		-2,115	4.10×10^-02
ENSG00000244669.1	RP11_158O161		Other	other		4,064	3.70×10^-02
ENSG00000254800.2	RP11_163O193		Other	other		3,012	3.89×10^-02
ENSG00000279311.1	RP11_170K42		Other	other		-1,44	4.53×10^-02
ENSG00000228566.1	RP11_170M171		Other	other		-3,625	4.23×10^-02
ENSG00000278935.1	RP11_173D34		Other	other		-1,536	8.39×10^-03
ENSG00000225531.1	RP11_196I183		Other	other		4,185	3.07×10^-02
ENSG00000279520.1	RP11_20I232		Other	other		-1,993	8.39×10^-03
ENSG00000240579.1	RP11_20L241		Other	other		-3,702	3.18×10^-02
ENSG00000237846.1	RP11_216M211		Other	other		-1,636	2.41×10^-02
ENSG00000250371.1	RP11_22A32		Other	other		2,361	1.70×10^-03
ENSG00000260823.1	RP11_249C2410		Other	other		3,679	4.15×10^-02
ENSG00000249731.1	RP11_259O23		Other	other		-1,03	2.36×10^-02
ENSG00000274204.1	RP11_272L142		Other	other		3,226	2.63×10^-02
ENSG00000243635.1	RP11_281P111		Other	other		6,824	8.11×10^-08
ENSG00000278698.1	RP11_282A113		Other	other		-1,702	8.50×10^-03
ENSG00000278963.1	RP11_294J227		Other	other		1,305	4.57×10^-02
ENSG00000257991.1	RP11_2H83		Other	other		8,913	1.14×10^-10
ENSG00000227700.1	RP11_301M171		Other	other		-3,509	1.70×10^-02
ENSG00000271519.1	RP11_307A172		Other	other		1,432	2.66×10^-02
ENSG00000261642.1	RP11_309H213		Other	other		1,827	5.77×10^-03
ENSG00000261298.1	RP11_309M71		Other	other		1,677	4.43×10^-02

ENSG00000272551.1	RP11_324L171		Other	other		3,029	1.01×10^-02
ENSG00000272583.1	RP11_344P136		Other	other		2,59	4.84×10^-02
ENSG00000231590.1	RP11_353J173		Other	other		1,804	3.23×10^-02
ENSG00000250430.1	RP11_366M415		Other	other		-3,549	3.50×10^-02
ENSG00000227217.1	RP11_367J73		Other	other		3,031	4.03×10^-02
ENSG00000272247.1	RP11_379F49		Other	other		2,228	2.71×10^-02
ENSG00000219329.1	RP11_397G52		Other	other		-3,53	3.35×10^-02
ENSG00000254258.1	RP11_398H61		Other	other		2,109	4.41×10^-02
ENSG00000279715.1	RP11_403N164		Other	other		1,022	3.21×10^-02
ENSG00000261519.3	RP11_403P174		Other	other		1,725	3.87×10^-02
ENSG00000279171.1	RP11_407A165		Other	other		-2,895	3.90×10^-02
ENSG00000247903.1	RP11_421F163		Other	other		2,994	4.58×10^-03
ENSG00000279862.1	RP11_423E72		Other	other		1,479	2.34×10^-02
ENSG00000267075.1	RP11_434D23		Other	other		-1,207	1.85×10^-02
ENSG00000271623.1	RP11_435I105		Other	other		-4,209	4.07×10^-03
ENSG00000260855.1	RP11_439E1910		Other	other		1,096	2.98×10^-02
ENSG00000271379.1	RP11_440G22		Other	other		3,093	2.75×10^-02
ENSG00000250596.1	RP11_440I142		Other	other		2,217	2.67×10^-02
ENSG00000275088.1	RP11_442O13		Other	other		-2,145	1.78×10^-02
ENSG00000231291.3	RP11_445O31		Other	other		2,445	3.01×10^-02
ENSG00000278060.1	RP11_466M211		Other	other		4,994	4.65×10^-03
ENSG00000248176.1	RP11_472K221		Other	other		4,588	3.01×10^-02
ENSG00000273064.1	RP11_474G233		Other	other		2,256	3.64×10^-02
ENSG00000272049.1	RP11_480D46		Other	other		4,776	2.65×10^-02
ENSG00000237850.7	RP11_483E232		Other	other		-1,16	3.00×10^-06
ENSG00000249926.2	RP11_495K96		Other	other		-2,531	3.59×10^-02
ENSG00000278330.1	RP11_4B164		Other	other		-1,39	2.83×10^-02
ENSG00000216663.3	RP11_506B65		Other	other		2,296	3.94×10^-02
ENSG00000280157.1	RP11_520H147		Other	other		1,509	3.02×10^-02
ENSG00000272335.1	RP11_53O193		Other	other		1,208	4.76×10^-02
ENSG00000280086.1	RP11_544P13		Other	other		-1,795	4.45×10^-02
ENSG00000275363.1	RP11_566K1912		Other	other		1,126	1.65×10^-02
ENSG00000232172.1	RP11_57C192		Other	other		-3,386	4.40×10^-02
ENSG00000257884.2	RP11_597A114		Other	other		-1,545	2.73×10^-02
ENSG00000279778.1	RP11_60A141		Other	other		1,731	2.33×10^-02
ENSG00000260182.1	RP11_616M225		Other	other		7,334	9.05×10^-09
ENSG00000225673.3	RP11_641C173		Other	other		4,976	7.04×10^-03
ENSG00000259648.1	RP11_643G164		Other	other		1,348	4.77×10^-02
ENSG00000234428.2	RP11_666F171		Other	other		2,395	2.01×10^-02
ENSG00000261734.1	RP11_669C191		Other	other		-1,194	1.80×10^-02
ENSG00000262181.1	RP11_683L232		Other	other		-3,47	4.04×10^-02
ENSG00000267349.1	RP11_686D229		Other	other		3,696	3.69×10^-02
ENSG00000277501.1	RP11_697E222		Other	other		1,675	1.80×10^-06
ENSG00000255234.5	RP11_727A2310		Other	other		-1,073	4.09×10^-03
ENSG00000258975.1	RP11_753D201		Other	other		-3,634	4.68×10^-02
ENSG00000257086.1	RP11_783K1613		Other	other		-3,59	4.59×10^-02
ENSG00000272703.1	RP11_78A194		Other	other		-2,526	3.52×10^-02
ENSG00000279682.1	RP11_791N191		Other	other		1,998	8.08×10^-03
ENSG00000270401.1	RP11_812E1914		Other	other		-3,51	4.89×10^-02
ENSG00000233974.3	RP11_823P93		Other	other		-3,078	7.51×10^-10
ENSG00000260470.1	RP11_834C1111		Other	other		-1,412	2.75×10^-02
ENSG00000280122.1	RP11_87G242		Other	other		-1,383	7.50×10^-03
ENSG00000232952.1	RP11_90H31		Other	other		1,569	1.52×10^-02
ENSG00000258505.1	RP11_90P161		Other	other		3,983	1.23×10^-03
ENSG00000266998.1	RP11_936I51		Other	other		-2,036	2.87×10^-03
ENSG00000255923.1	RP11_983C21		Other	other		-2,772	4.05×10^-02
ENSG00000260610.1	RP11_989E611		Other	other		-2,333	4.56×10^-03
ENSG00000251158.1	RP11_98J231		Other	other		-1,067	1.82×10^-04
ENSG00000273211.1	RP13_131K197		Other	other		-3,554	4.34×10^-02
ENSG00000260585.1	RP13_192B192		Other	other		1,561	4.74×10^-02
ENSG00000279072.1	RP13_580B184		Other	other		-1,024	4.49×10^-06
ENSG00000279942.1	RP1_153P147		Other	other		-1,03	2.35×10^-02
ENSG00000230973.1	RP1_154J132		Other	other		4,073	4.07×10^-02
ENSG00000278206.1	RP1_20N28		Other	other		1,617	3.91×10^-02
ENSG00000212939.2	RP1_29C1810		Other	other		-1,812	4.73×10^-02
ENSG00000220960.1	RP1_72A231		Other	other		4,747	4.36×10^-02
ENSG00000216917.2	RP5_988G151		Other	other		3,268	2.15×10^-02
ENSG00000147403.16	RPL10	ribosomal protein L10	Cytoplasm	translation regulator		2,225	1.58×10^-09
ENSG00000142676.12	RPL11	ribosomal protein L11	Cytoplasm	other		3,024	1.26×10^-14
ENSG00000197958.12	RPL12	ribosomal protein L12	Nucleus	other		1,555	1.42×10^-08
ENSG00000142541.16	RPL13A	ribosomal protein L13a	Cytoplasm	other		2,87	9.72×10^-35
ENSG00000188846.13	RPL14	ribosomal protein L14	Cytoplasm	other		1,433	2.65×10^-11
ENSG00000174748.18	RPL15	ribosomal protein L15	Cytoplasm	other		1,894	5.51×10^-11
ENSG00000265681.6	RPL17	ribosomal protein L17	Cytoplasm	other		2,719	6.89×10^-24
ENSG00000105640.12	RPL18A	ribosomal protein L18a	Cytoplasm	other		2,089	3.23×10^-14
ENSG00000108298.9	RPL19	ribosomal protein L19	Cytoplasm	other		2,071	8.52×10^-18
ENSG00000122026.10	RPL21	ribosomal protein L21	Cytoplasm	other		2,175	7.60×10^-10
ENSG00000116251.9	RPL22	ribosomal protein L22	Cytoplasm	translation regulator		1,972	6.11×10^-12
ENSG00000225483.1	RPL22P4	ribosomal protein L22 pseudogene 4	Other	other		4,534	3.40×10^-02
ENSG00000125691.12	RPL23	ribosomal protein L23	Cytoplasm	other		1,63	6.06×10^-11
ENSG00000198242.13	RPL23A	ribosomal protein L23a	Cytoplasm	other		2,596	6.45×10^-26
ENSG00000213122.4	RPL23AP46	ribosomal protein L23a pseudogene 46	Other	other		3,586	4.79×10^-02
ENSG00000114391.12	RPL24	ribosomal protein L24	Cytoplasm	other		1,442	3.64×10^-04
ENSG00000161970.12	RPL26	ribosomal protein L26	Cytoplasm	other		2,57	1.03×10^-24
ENSG00000131469.12	RPL27	ribosomal protein L27	Cytoplasm	other		1,758	3.90×10^-08
ENSG00000166441.12	RPL27A	ribosomal protein L27a	Cytoplasm	other		1,133	1.72×10^-04
ENSG00000108107.12	RPL28	ribosomal protein L28	Cytoplasm	other		1,689	1.05×10^-14
ENSG00000162244.10	RPL29	ribosomal protein L29	Cytoplasm	other		1,594	8.20×10^-04
ENSG00000100316.15	RPL3	ribosomal protein L3	Nucleus	other	cytarabine/daunorubicin/oma	2,356	4.61×10^-27
ENSG00000071082.10	RPL31	ribosomal protein L31	Cytoplasm	other		1,098	1.67×10^-03
ENSG00000144713.12	RPL32	ribosomal protein L32	Cytoplasm	other		1,983	2.35×10^-11
ENSG00000109475.16	RPL34	ribosomal protein L34	Cytoplasm	other		1,872	1.68×10^-08
ENSG00000136942.14	RPL35	ribosomal protein L35	Cytoplasm	other		2,808	1.27×10^-16
ENSG00000182899.14	RPL35A	ribosomal protein L35a	Cytoplasm	other		2,112	3.14×10^-03
ENSG00000130255.12	RPL36	ribosomal protein L36	Cytoplasm	other		1,136	3.70×10^-02
ENSG00000241343.9	RPL36A	ribosomal protein L36a	Cytoplasm	other		2,621	1.14×10^-22
ENSG00000165502.6	RPL36AL	ribosomal protein L36a like	Cytoplasm	other		2,243	2.33×10^-07
ENSG00000172809.12	RPL38	ribosomal protein L38	Cytoplasm	other		2,221	3.04×10^-16
ENSG00000198918.7	RPL39	ribosomal protein L39	Cytoplasm	other		1,483	5.44×10^-03
ENSG00000174444.14	RPL4	ribosomal protein L4	Cytoplasm	enzyme		2,202	2.88×10^-26
ENSG00000122406.12	RPL5	ribosomal protein L5	Cytoplasm	other		3,707	1.86×10^-46
ENSG00000243521.2	RPL5P33	ribosomal protein L5 pseudogene 33	Other	other		2,352	4.46×10^-02
ENSG00000089009.15	RPL6	ribosomal protein L6	Nucleus	other		1,489	7.86×10^-07
ENSG00000147604.13	RPL7	ribosomal protein L7	Nucleus	transcription regulator		2,265	5.95×10^-11
ENSG00000148303.16	RPL7A	ribosomal protein L7a	Cytoplasm	other		1,762	2.63×10^-10
ENSG00000240622.1	RPL7P15	ribosomal protein L7 pseudogene 15	Other	other		4,222	3.40×10^-02
ENSG00000161016.15	RPL8	ribosomal protein L8	Cytoplasm	other		1,617	1.49×10^-03
ENSG00000163682.15	RPL9	ribosomal protein L9	Nucleus	other		1,261	3.32×10^-02
ENSG00000237550.5	RPL9P9		Other	other		4,356	1.13×10^-06
ENSG00000089157.15	RPLP0	ribosomal protein lateral stalk subunit P0	Cytoplasm	other		1,846	6.61×10^-13
ENSG00000137818.11	RPLP1	ribosomal protein lateral stalk subunit P1	Cytoplasm	other		2,717	3.72×10^-22
ENSG00000177600.8	RPLP2	ribosomal protein lateral stalk subunit P2	Cytoplasm	other		3,109	2.09×10^-25
ENSG00000259001.3	RPPH1		Other	other		1,98	2.22×10^-11
ENSG00000277209.1	RPPH1	ribonuclease P RNA component H1	Other	other		6,169	9.30×10^-04
ENSG00000124614.13	RPS10	ribosomal protein S10	Cytoplasm	other		2,861	1.38×10^-08
ENSG00000142534.6	RPS11	ribosomal protein S11	Cytoplasm	other		1,859	3.28×10^-04
ENSG00000112306.7	RPS12	ribosomal protein S12	Cytoplasm	other	neomycin	3,694	2.05×10^-07
ENSG00000110700.6	RPS13	ribosomal protein S13	Cytoplasm	other		1,308	2.76×10^-02
ENSG00000115268.9	RPS15	ribosomal protein S15	Cytoplasm	other		2,179	2.21×10^-08
ENSG00000134419.15	RPS15A	ribosomal protein S15a	Cytoplasm	other		1,26	7.95×10^-06
ENSG00000182774.10	RPS17	ribosomal protein S17	Cytoplasm	other		1,335	1.43×10^-05
ENSG00000231500.6	RPS18	ribosomal protein S18	Cytoplasm	other		1,785	1.58×10^-08
ENSG00000105372.6	RPS19	ribosomal protein S19	Cytoplasm	other		1,64	4.92×10^-08
ENSG00000140988.15	RPS2	ribosomal protein S2	Cytoplasm	other		1,951	7.36×10^-14
ENSG00000008988.9	RPS20	ribosomal protein S20	Cytoplasm	other		2,259	3.81×10^-15
ENSG00000186468.12	RPS23	ribosomal protein S23	Cytoplasm	translation regulator		2,453	4.90×10^-24
ENSG00000138326.18	RPS24	ribosomal protein S24	Cytoplasm	other		1,345	2.13×10^-05
ENSG00000118181.10	RPS25	ribosomal protein S25	Cytoplasm	other		2,01	8.14×10^-06
ENSG00000197728.9	RPS26	ribosomal protein S26	Cytoplasm	other		2,222	1.27×10^-09
ENSG00000177954.11	RPS27	ribosomal protein S27	Cytoplasm	other	empesertib	1,869	2.55×10^-07
ENSG00000143947.12	RPS27A	ribosomal protein S27a	Cytoplasm	other		2,106	9.32×10^-09
ENSG00000185088.12	RPS27L	ribosomal protein S27 like	Cytoplasm	translation regulator		1,033	1.98×10^-03
ENSG00000149273.14	RPS3	ribosomal protein S3	Cytoplasm	enzyme		1,5	4.87×10^-09
ENSG00000145425.9	RPS3A	ribosomal protein S3A	Nucleus	other		2,795	2.39×10^-19
ENSG00000226292.1	RPS3AP10	RPS3A pseudogene 10	Other	other		-3,652	3.42×10^-02
ENSG00000198034.10	RPS4X	ribosomal protein S4 X-linked	Cytoplasm	other		2,993	8.97×10^-28
ENSG00000083845.8	RPS5	ribosomal protein S5	Cytoplasm	other		1,024	4.34×10^-02
ENSG00000137154.12	RPS6	ribosomal protein S6	Cytoplasm	other		2,672	6.42×10^-19

ENSG00000072133.10	RPS6KA6	ribosomal protein S6 kinase A6	Cytoplasm	kinase	PMD-026	1,809	4.19×10^-03
ENSG00000142937.11	RPS8	ribosomal protein S8	Cytoplasm	other		2,247	7.30×10^-09
ENSG00000170889.13	RPS9	ribosomal protein S9	Cytoplasm	translation regulator		1,577	1.16×10^-05
ENSG00000168028.13	RPSA	ribosomal protein SA	Cytoplasm	translation regulator		2,064	2.98×10^-09
ENSG00000167325.14	RRM1	ribonucleotide reductase catalytic subunit M1	Nucleus	enzyme	L-asparaginase/gemcitabine/	1,764	4.94×10^-02
ENSG00000214018.3	RRM2P3	ribonucleotide reductase M2 polypeptide pseudogene 3	Other	other		2,251	2.21×10^-02
ENSG00000026036.20	RTEL1-TNFRSF6B	RTEL1-TNFRSF6B readthrough (NMD candidate)	Other	other		-2,933	2.82×10^-02
ENSG00000140688.16	RUSF1	RUS family member 1	Other	other		-1,479	4.15×10^-02
ENSG00000197747.8	S100A10	S100 calcium binding protein A10	Cytoplasm	other		1,945	4.50×10^-04
ENSG00000163191.5	S100A11	S100 calcium binding protein A11	Cytoplasm	other		4,46	5.09×10^-09
ENSG00000188643.10	S100A16	S100 calcium binding protein A16	Nucleus	other		2,138	3.62×10^-03
ENSG00000197956.9	S100A6	S100 calcium binding protein A6	Cytoplasm	transporter		2,711	4.09×10^-05
ENSG00000165821.11	SALL2	spalt like transcription factor 2	Nucleus	transcription regulator		-1,023	1.38×10^-02
ENSG00000161526.14	SAP30BP	SAP30 binding protein	Nucleus	transcription regulator		-1,067	3.58×10^-02
ENSG00000079332.14	SAR1A	secretion associated Ras related GTPase 1A	Cytoplasm	enzyme		1,235	1.85×10^-03
ENSG00000205323.8	SARNP	SAP domain containing ribonucleoprotein	Nucleus	transcription regulator		2,402	1.85×10^-03
ENSG00000130066.16	SAT1	spermidine/spermine N1-acetyltransferase 1	Cytoplasm	enzyme		2,765	5.57×10^-16
ENSG00000248522.1	SBF1P1	SET binding factor 1 pseudogene 1	Other	other		-1,751	1.80×10^-02
ENSG00000252481.1	SCARNA13	small Cajal body-specific RNA 13	Nucleus	other		2,942	2.26×10^-02
ENSG00000280466.1	SCARNA4	small Cajal body-specific RNA 4	Other	other		4,755	1.16×10^-02
ENSG00000161929.14	SCIMP	SLP adaptor and CSK interacting membrane protein	Plasma Membrane	other		-2,006	2.73×10^-02
ENSG00000007314.11	SCN4A	sodium voltage-gated channel alpha subunit 4	Plasma Membrane	ion channel	diclofenac/omeprazole, diclof	-1,121	1.27×10^-02
ENSG00000144306.13	SCRN3	secernin 3	Other	other		1,304	4.74×10^-02
ENSG00000150961.14	SEC24D	SEC24 homolog D, COPII coat complex component	Cytoplasm	transporter		1,145	2.65×10^-02
ENSG00000132432.13	SEC61G	SEC61 translocon subunit gamma	Plasma Membrane	transporter		2,628	2.85×10^-05
ENSG00000131871.14	SELENOS	selenoprotein S	Cytoplasm	other		1,302	1.77×10^-03
ENSG00000196189.12	SEMA4A	semaphorin 4A	Plasma Membrane	other		-1,362	2.18×10^-03
ENSG00000167680.15	SEMA6B	semaphorin 6B	Plasma Membrane	other		1,643	3.50×10^-02
ENSG00000138468.15	SENP7	SUMO specific peptidase 7	Nucleus	peptidase		1,928	4.38×10^-02
ENSG00000186522.14	SEPTIN10	septin 10	Cytoplasm	transcription regulator		1,731	3.18×10^-04
ENSG00000281379.2	SEPTIN14P19	septin 14 pseudogene 19	Other	other		2,35	3.56×10^-02
ENSG00000168385.17	SEPTIN2	septin 2	Cytoplasm	enzyme		1,161	1.61×10^-02
ENSG00000259090.1	SEPTIN7P1	septin 7 pseudogene 1	Other	other		3,701	3.48×10^-04
ENSG00000142864.14	SERBP1	SERPINE1 mRNA binding protein 1	Cytoplasm	other		1,237	2.01×10^-03
ENSG00000205572.9	SERF1A/SERF1B	small EDRK-rich factor 1A	Other	other		-1,659	2.66×10^-02
ENSG00000119335.16	SET	SET nuclear proto-oncogene	Nucleus	phosphatase		1,522	1.57×10^-06
ENSG00000136169.16	SETDB2	SET domain bifurcated histone lysine methyltransferase 2	Nucleus	enzyme		1,663	9.54×10^-05
ENSG00000227541.1	SFR1P1	SFR1 pseudogene 1	Other	other		4,115	3.49×10^-02
ENSG00000145423.4	SFRP2	secreted frizzled related protein 2	Plasma Membrane	transmembrane receptor		3,332	4.63×10^-24
ENSG00000181523.12	SGSH	N-sulfoglucosamine sulfohydrolase	Cytoplasm	enzyme		-1,427	4.14×10^-02
ENSG00000131171.12	SH3BGRL	SH3 domain binding glutamate rich protein like	Cytoplasm	other		2,686	3.23×10^-02
ENSG00000105251.10	SHD	Src homology 2 domain containing transforming protein D	Cytoplasm	other		-1,611	2.75×10^-02
ENSG00000198892.6	SHISA4	shisa family member 4	Other	other		-3,743	2.66×10^-02
ENSG00000171984.14	SHLD1	shieldin complex subunit 1	Nucleus	other		1,89	9.53×10^-03
ENSG00000165874.12	SHLD2P1	shieldin complex subunit 2 pseudogene 1	Other	other		-1,214	1.80×10^-02
ENSG00000275993.2	SIK1/SIK1B	salt inducible kinase 1	Nucleus	kinase	GRN-300, dabrafenib, dabraf	2,037	9.35×10^-03
ENSG00000138083.4	SIX3	SIX homeobox 3	Nucleus	transcription regulator		-1,456	2.39×10^-04
ENSG00000136603.13	SKIL	SKI like proto-oncogene	Nucleus	transcription regulator		1,68	1.67×10^-04
ENSG00000145604.15	SKP2	S-phase kinase associated protein 2	Nucleus	other		1,442	2.18×10^-02
ENSG00000104888.9	SLC17A7	solute carrier family 17 member 7	Plasma Membrane	transporter		-1,643	2.11×10^-10
ENSG00000168065.15	SLC22A11	solute carrier family 22 member 11	Plasma Membrane	transporter	colchicine/probenecid, lesinu	-1,54	1.12×10^-02
ENSG00000102743.14	SLC25A15	solute carrier family 25 member 15	Cytoplasm	transporter		2,032	1.56×10^-02
ENSG00000085491.15	SLC25A24	solute carrier family 25 member 24	Cytoplasm	transporter		1,254	4.40×10^-03
ENSG00000005022.5	SLC25A5	solute carrier family 25 member 5	Cytoplasm	transporter	clodronic acid	2,781	1.78×10^-03
ENSG00000169100.12	SLC25A6	solute carrier family 25 member 6	Cytoplasm	transporter	clodronic acid	2,295	2.55×10^-07
ENSG00000135502.16	SLC26A10	solute carrier family 26 member 10	Other	other		1,735	1.10×10^-04
ENSG00000091137.11	SLC26A4	solute carrier family 26 member 4	Plasma Membrane	transporter		1,767	2.34×10^-02
ENSG00000225697.10	SLC26A6	solute carrier family 26 member 6	Cytoplasm	transporter		-1,299	1.71×10^-02
ENSG00000158014.14	SLC30A2	solute carrier family 30 member 2	Plasma Membrane	transporter		-2,022	3.93×10^-03
ENSG00000131183.10	SLC34A1	solute carrier family 34 member 1	Plasma Membrane	transporter		-1,519	3.57×10^-02
ENSG00000134294.13	SLC38A2	solute carrier family 38 member 2	Plasma Membrane	transporter		1,299	6.57×10^-04
ENSG00000138079.13	SLC3A1	solute carrier family 3 member 1	Plasma Membrane	transporter		2,844	2.53×10^-24
ENSG00000164889.12	SLC4A2	solute carrier family 4 member 2	Plasma Membrane	transporter		-1,301	7.44×10^-03
ENSG00000063127.15	SLC6A16	solute carrier family 6 member 16	Plasma Membrane	transporter		-1,475	1.05×10^-02
ENSG00000164363.9	SLC6A18	solute carrier family 6 member 18	Plasma Membrane	transporter		4,23	4.70×10^-03
ENSG00000011083.8	SLC6A7	solute carrier family 6 member 7	Plasma Membrane	transporter		-1,626	5.32×10^-03
ENSG00000103257.8	SLC7A5	solute carrier family 7 member 5	Plasma Membrane	transporter		-2,417	4.62×10^-02
ENSG00000115616.2	SLC9A2	solute carrier family 9 member A2	Plasma Membrane	transporter		1,694	8.06×10^-08
ENSG00000179542.15	SLITRK4	SLIT and NTRK like family member 4	Extracellular Space	other		1,114	3.00×10^-02
ENSG00000065613.13	SLK	STE20 like kinase	Nucleus	kinase		1,188	1.22×10^-02
ENSG00000163681.14	SLMAP	sarcolemma associated protein	Plasma Membrane	other		1,157	3.17×10^-05
ENSG00000137776.16	SLTM	SAFB like transcription modulator	Nucleus	other		1,11	6.57×10^-03
ENSG00000170365.9	SMAD1	SMAD family member 1	Nucleus	transcription regulator		1,234	3.65×10^-02
ENSG00000080503.19	SMARCA2	SWI/SNF related, matrix associated, actin dependent regulator of chromatin, subfamily a, member 2	Nucleus	transcription regulator		1,382	2.81×10^-02
ENSG00000127616.17	SMARCA4	SWI/SNF related, matrix associated, actin dependent regulator of chromatin, subfamily a, member 4	Nucleus	transcription regulator		1,111	1.77×10^-02
ENSG00000224531.5	SMIM13	small integral membrane protein 13	Other	other		1,512	3.08×10^-02
ENSG00000262543.1	SMIM28	small integral membrane protein 28	Other	other		-3,578	2.73×10^-02
ENSG00000205571.12	SMN1/SMN2	survival of motor neuron 1, telomeric	Nucleus	other	nusinersen, risdiplam	2,834	8.14×10^-03
ENSG00000103056.11	SMPD3	sphingomyelin phosphodiesterase 3	Cytoplasm	enzyme		-1,424	2.73×10^-02
ENSG00000102172.15	SMS	spermine synthase	Cytoplasm	enzyme	(±)-2-hydroxyoleic acid	2,403	5.34×10^-04
ENSG00000242125.3	SNHG3	small nucleolar RNA host gene 3	Nucleus	other		1,096	1.56×10^-03
ENSG00000281398.2	SNHG4	small nucleolar RNA host gene 4	Other	other		2,404	7.00×10^-04
ENSG00000207181.1	SNORA14B	small nucleolar RNA, H/ACA box 14B	Other	other		2,503	1.41×10^-02
ENSG00000278274.1	SNORA61	small nucleolar RNA, H/ACA box 61	Other	other		6,164	4.07×10^-05
ENSG00000274266.1	SNORA73A	small nucleolar RNA, H/ACA box 73A	Nucleus	other		7,767	2.01×10^-06
ENSG00000200087.1	SNORA73B	small nucleolar RNA, H/ACA box 73B	Other	other		3,447	5.69×10^-04
ENSG00000199753.1	SNORD104	small nucleolar RNA, C/D box 104	Other	other		5,471	7.41×10^-04
ENSG00000276788.1	SNORD26	small nucleolar RNA, C/D box 26	Other	other		4,691	2.34×10^-02
ENSG00000167088.10	SNRPD1	small nuclear ribonucleoprotein D1 polypeptide	Nucleus	other		1,454	1.49×10^-03
ENSG00000104497.13	SNX16	sorting nexin 16	Cytoplasm	transporter		1,976	1.47×10^-02
ENSG00000112335.14	SNX3	sorting nexin 3	Cytoplasm	transporter		2,171	2.59×10^-04
ENSG00000173548.8	SNX33	sorting nexin 33	Cytoplasm	other		-1,335	4.74×10^-03
ENSG00000185338.4	SOCS1	suppressor of cytokine signaling 1	Cytoplasm	other		3,107	7.40×10^-31
ENSG00000142168.14	SOD1	superoxide dismutase 1	Cytoplasm	enzyme		2,307	1.52×10^-02
ENSG00000113140.10	SPARC	secreted protein acidic and cysteine rich	Extracellular Space	other		1,422	9.63×10^-07
ENSG00000152583.12	SPARCL1	SPARC like 1	Extracellular Space	other		1,561	6.11×10^-03
ENSG00000133104.12	SPART	spartin	Cytoplasm	other		1,262	3.11×10^-02
ENSG00000189357.8	SPATA31D1 (includes others)	SPATA31 subfamily D member 1	Other	other		-3,525	2.99×10^-03
ENSG00000066336.11	SPI1	Spi-1 proto-oncogene	Nucleus	transcription regulator		1,371	3.55×10^-04
ENSG00000106723.16	SPIN1	spindlin 1	Nucleus	other		2,027	6.60×10^-05
ENSG00000107742.12	SPOCK2	SPARC (osteonectin), cwcv and kazal like domains proteoglycan 2	Extracellular Space	other		-1,073	3.70×10^-02
ENSG00000171621.13	SPSB1	splA/ryanodine receptor domain and SOCS box containing 1	Cytoplasm	other		2,043	6.20×10^-07
ENSG00000160460.15	SPTBN4	spectrin beta, non-erythrocytic 4	Cytoplasm	other		-1,417	1.52×10^-02
ENSG00000165389.6	SPTSSA	serine palmitoyltransferase small subunit A	Cytoplasm	enzyme		2,126	5.21×10^-05
ENSG00000161011.19	SQSTM1	sequestosome 1	Cytoplasm	transcription regulator		1,606	4.91×10^-06
ENSG00000140319.10	SRP14	signal recognition particle 14	Cytoplasm	other		3	7.58×10^-21
ENSG00000143742.12	SRP9	signal recognition particle 9	Cytoplasm	other		2,982	5.19×10^-05
ENSG00000136450.12	SRSF1	serine and arginine rich splicing factor 1	Nucleus	other		1,317	1.69×10^-02
ENSG00000116754.13	SRSF11	serine and arginine rich splicing factor 11	Nucleus	other		1,208	1.76×10^-04
ENSG00000112081.16	SRSF3	serine and arginine rich splicing factor 3	Nucleus	other		1,031	3.14×10^-02
ENSG00000111786.8	SRSF9	serine and arginine rich splicing factor 9	Nucleus	enzyme		1,885	8.62×10^-03
ENSG00000179954.14	SSC5D	scavenger receptor cysteine rich family member with 5 domains	Plasma Membrane	transmembrane receptor		-1,18	1.22×10^-03
ENSG00000123096.11	SSPN	sarcospan	Plasma Membrane	other		1,084	1.36×10^-02
ENSG00000235374.2	SSR4P1	signal sequence receptor subunit 4 pseudogene 1	Other	other		2,547	8.00×10^-17
ENSG00000278195.1	SSTR3	somatostatin receptor 3	Plasma Membrane	G-protein coupled receptor	pasireotide, everolimus/pasir	-1,041	3.97×10^-02
ENSG00000268447.5	SSX2/SSX2B	SSX family member 2	Nucleus	other		2,468	3.80×10^-02
ENSG00000100380.12	ST13	ST13 Hsp70 interacting protein	Cytoplasm	other		1,356	2.91×10^-03
ENSG00000149418.10	ST14	ST14 transmembrane serine protease matriptase	Plasma Membrane	peptidase		-1,995	3.23×10^-02
ENSG00000007341.18	ST7L	suppression of tumorigenicity 7 like	Other	other		-1,256	2.95×10^-02
ENSG00000010327.10	STAB1	stabilin 1	Plasma Membrane	transporter	FP-1305	-1,729	3.91×10^-02
ENSG00000101972.18	STAG2	stromal antigen 2	Nucleus	other		2,008	6.83×10^-04
ENSG00000040341.17	STAU2	staufen double-stranded RNA binding protein 2	Cytoplasm	other		1,365	3.68×10^-03
ENSG00000167323.9	STIM1	stromal interaction molecule 1	Plasma Membrane	ion channel		-1,48	1.03×10^-02
ENSG00000184584.12	STING1	stimulator of interferon response cGAMP interactor 1	Cytoplasm	other	CDK-002, TAK-676, SNX281	-1,802	1.15×10^-02
ENSG00000144589.20	STK11IP	serine/threonine kinase 11 interacting protein	Cytoplasm	other		-1,342	1.34×10^-02
ENSG00000204344.14	STK19	serine/threonine kinase 19	Nucleus	kinase		-1,373	4.02×10^-03
ENSG00000068781.20	STON1-GTF2A1L	STON1-GTF2A1L readthrough	Cytoplasm	other		-2,869	8.39×10^-03
ENSG00000023734.10	STRAP	serine/threonine kinase receptor associated protein	Plasma Membrane	other		3,256	2.72×10^-02
ENSG00000164506.14	STXBP5	syntaxin binding protein 5	Plasma Membrane	other		1,326	1.72×10^-02
ENSG00000188612.11	SUMO2	small ubiquitin like modifier 2	Nucleus	enzyme		1,207	3.97×10^-02
ENSG00000248278.1	SUMO2P17		Other	other		4,539	1.07×10^-06
ENSG00000184900.15	SUMO3	small ubiquitin like modifier 3	Nucleus	other		1,451	4.53×10^-02
ENSG00000196235.13	SUPT5H	SPT5 homolog, DSIF elongation factor subunit	Nucleus	transcription regulator		-1,092	1.24×10^-02
ENSG00000198168.8	SVIP	small VCP interacting protein	Cytoplasm	other		1,316	3.91×10^-02
ENSG00000135316.17	SYNCRIP	synaptotagmin binding cytoplasmic RNA interacting protein	Nucleus	other	supinoxin	1,181	2.59×10^-03
ENSG00000101463.5	SYNDIG1	synapse differentiation inducing 1	Plasma Membrane	other		-2,986	2.63×10^-02
ENSG00000183379.8	SYNDIG1L	synapse differentiation inducing 1 like	Cytoplasm	other		-1,599	2.51×10^-02
ENSG00000182253.14	SYNM	synemin	Cytoplasm	other		-1,04	1.07×10^-02

ENSG00000143028.8	SYPL2	synaptophysin like 2	Other	other		-1,187	1.87×10^-02
ENSG00000213023.9	SYT3	synaptotagmin 3	Cytoplasm	transporter		-1,44	5.25×10^-04
ENSG00000157625.15	TAB3	TGF-beta activated kinase 1 (MAP3K7) binding protein 3	Cytoplasm	other		1,007	1.32×10^-02
ENSG00000231925.11	TAPBP	TAP binding protein	Cytoplasm	transporter		-1,365	1.43×10^-02
ENSG00000212128.2	TAS2R13	taste 2 receptor member 13	Plasma Membrane	G-protein coupled receptor		2,124	4.10×10^-02
ENSG00000164532.10	TBX20	T-box transcription factor 20	Nucleus	transcription regulator		1,974	1.65×10^-03
ENSG00000133142.17	TCEAL4	transcription elongation factor A like 4	Other	other		1,176	1.76×10^-02
ENSG00000185222.7	TCEAL9	transcription elongation factor A like 9	Other	other		2,343	4.06×10^-05
ENSG00000215029.8	TCP11X2	t-complex 11 family, X-linked 2	Other	other		2,056	2.79×10^-02
ENSG00000223824.1	TERF1P6	TERF1 pseudogene 6	Other	other		2,817	6.58×10^-03
ENSG00000131126.18	TEX101	testis expressed 101	Plasma Membrane	other		3,057	7.39×10^-11
ENSG00000140682.18	TGFB1I1	transforming growth factor beta 1 induced transcript 1	Nucleus	transcription regulator		-1,131	4.91×10^-03
ENSG00000137801.10	THBS1	thrombospondin 1	Extracellular Space	other		1,775	2.47×10^-18
ENSG00000130775.15	THEMIS2	thymocyte selection associated family member 2	Other	other		-1,149	1.20×10^-02
ENSG00000227339.1	THRAP3P1	THRAP3 pseudogene 1	Other	other		-1,345	4.53×10^-02
ENSG00000255833.1	TIFAB	TIFA inhibitor	Other	other		-1,392	1.47×10^-02
ENSG00000137221.14	TJAP1	tight junction associated protein 1	Plasma Membrane	other		-1,566	1.09×10^-03
ENSG00000196781.13	TLE1	TLE family member 1, transcriptional corepressor	Nucleus	transcription regulator		1,222	4.22×10^-02
ENSG00000104953.18	TLE6	TLE family member 6, subcortical maternal complex member	Nucleus	other		-3,408	2.09×10^-02
ENSG00000239732.3	TLR9	toll like receptor 9	Plasma Membrane	transmembrane receptor	cavrotolimod, MGN1703, ISS	-4,503	2.23×10^-03
ENSG00000136404.15	TM6SF1	transmembrane 6 superfamily member 1	Plasma Membrane	other		5,579	1.59×10^-07
ENSG00000232112.3	TMA7	translation machinery associated 7 homolog	Other	other		1,487	2.57×10^-02
ENSG00000149488.12	TMC2	transmembrane channel like 2	Plasma Membrane	ion channel		-1,151	4.68×10^-02
ENSG00000162542.13	TMCO4	transmembrane and coiled-coil domains 4	Other	other		2,005	2.68×10^-02
ENSG00000091947.9	TMEM101	transmembrane protein 101	Extracellular Space	other		-1,653	4.12×10^-02
ENSG00000183307.3	TMEM121B	transmembrane protein 121B	Nucleus	transcription regulator		-1,455	9.35×10^-03
ENSG00000152558.14	TMEM123	transmembrane protein 123	Plasma Membrane	other		2,12	2.49×10^-02
ENSG00000184497.12	TMEM255B	transmembrane protein 255B	Other	other		-1,625	6.06×10^-03
ENSG00000180694.13	TMEM64	transmembrane protein 64	Cytoplasm	other		1,757	2.25×10^-03
ENSG00000224533.4	TMLHE-AS1	TMLHE antisense RNA 1	Other	other		2,225	1.90×10^-02
ENSG00000034510.5	TMSB10/TMSB4X	thymosin beta 4 X-linked	Cytoplasm	other		3,252	1.78×10^-19
ENSG00000205542.10	TMSB10/TMSB4X	thymosin beta 4 X-linked	Cytoplasm	other		2,371	5.70×10^-12
ENSG00000240505.8	TNFRSF13B	TNF receptor superfamily member 13B	Plasma Membrane	transmembrane receptor		-1,556	9.82×10^-07
ENSG00000061938.16	TNK2	tyrosine kinase non receptor 2	Cytoplasm	kinase	vemurafenib, osimertinib, ent	-1,144	4.56×10^-03
ENSG00000083312.17	TNPO1	transportin 1	Nucleus	transporter		1,578	3.52×10^-05
ENSG00000230662.1	TNPO1P2	transportin 1 pseudogene 2	Other	other		5,849	3.77×10^-06
ENSG00000182095.14	TNRC18	trinucleotide repeat containing 18	Nucleus	other		-1,513	1.98×10^-05
ENSG00000223566.1	TNRC18P2	trinucleotide repeat containing 18 pseudogene 2	Other	other		-1,645	2.23×10^-04
ENSG00000168477.17	TNXB	tenascin XB	Extracellular Space	other		-2,082	6.16×10^-04
ENSG00000173726.10	TOMM20	translocase of outer mitochondrial membrane 20	Cytoplasm	transporter		2,027	7.27×10^-05
ENSG00000198900.5	TOP1	DNA topoisomerase I	Nucleus	enzyme	bevacizumab/irinotecan/oxali	1,604	1.23×10^-02
ENSG00000077097.13	TOP2B	DNA topoisomerase II beta	Nucleus	enzyme	daunorubicin/tretinoin, chlora	1,957	4.53×10^-03
ENSG00000103460.16	TOX3	TOX high mobility group box family member 3	Nucleus	transcription regulator		1,265	4.57×10^-02
ENSG00000143549.19	TPM3	tropomyosin 3	Cytoplasm	other	AZD-7451	1,075	3.20×10^-05
ENSG00000167460.14	TPM4	tropomyosin 4	Cytoplasm	other		2,103	4.28×10^-14
ENSG00000047410.13	TPR	translocated promoter region, nuclear basket protein	Nucleus	other		1,379	2.03×10^-03
ENSG00000188001.9	TPRG1	tumor protein p63 regulated 1	Cytoplasm	other		1,079	5.33×10^-04
ENSG00000116176.6	TPSG1	tryptase gamma 1	Extracellular Space	peptidase		-3,53	4.63×10^-02
ENSG00000133112.16	TPT1	tumor protein, translationally-controlled 1	Cytoplasm	other		1,918	1.91×10^-17
ENSG00000115993.11	TRAK2	trafficking kinesin protein 2	Plasma Membrane	transporter		1,413	5.44×10^-08
ENSG00000225992.1	TRGVA	T cell receptor gamma variable A (pseudogene)	Other	other		5,622	2.92×10^-03
ENSG00000130726.11	TRIM28	tripartite motif containing 28	Nucleus	transcription regulator		2,73	1.81×10^-04
ENSG00000166007.11	TRIM51HP	tripartite motif-containing 51H, pseudogene	Other	other		1,535	3.53×10^-02
ENSG00000223709.5	TRIM64EP	tripartite motif containing 64E, pseudogene	Other	other		4,452	8.75×10^-03
ENSG00000178809.11	TRIM73/TRIM74	tripartite motif containing 74	Cytoplasm	other		-1,285	1.89×10^-02
ENSG00000204025.6	TRPC5OS	TRPC5 opposite strand	Other	other		1,652	1.02×10^-02
ENSG00000196689.10	TRPV1	transient receptor potential cation channel subfamily V member 1	Plasma Membrane	ion channel	AZD1386, capsaicin, resinife	-2,231	1.66×10^-02
ENSG00000165832.5	TRUB1	TruB pseudouridine synthase family member 1	Other	enzyme		1,507	4.68×10^-02
ENSG00000255032.1	TSPAN18-AS1		Other	other		4,616	2.75×10^-02
ENSG00000156298.12	TSPAN7	tetraspanin 7	Plasma Membrane	other		2,97	2.75×10^-02
ENSG00000233803.8	TSPY1 (includes others)	testis specific protein Y-linked 1	Other	other		-2,317	5.85×10^-03
ENSG00000258992.5	TSPY1 (includes others)	testis specific protein Y-linked 1	Other	other		-3,858	2.30×10^-02
ENSG00000146216.11	TTBK1	tau tubulin kinase 1	Other	kinase		-1,854	4.17×10^-04
ENSG00000215105.4	TTC3P1	tetratricopeptide repeat domain 3 pseudogene 1	Other	other		1,573	2.11×10^-02
ENSG00000205231.1	TTLL10-AS1	TTLL10 antisense RNA 1	Other	other		2,632	2.13×10^-03
ENSG00000233061.1	TTLL7-IT1	TTLL7 intronic transcript 1	Other	other		1,684	2.11×10^-02
ENSG00000225154.2	TUBAP9	tubulin alpha pseudogene 9	Other	other		-4,547	1.61×10^-03
ENSG00000196230.12	TUBB	tubulin beta class I	Cytoplasm	other	ixabepilone/rifampin, allopuri	1,387	8.24×10^-06
ENSG00000137267.5	TUBB2A	tubulin beta 2A class IIa	Cytoplasm	other	docetaxel/gemcitabine/vincris	2,265	2.01×10^-02
ENSG00000104833.10	TUBB4A	tubulin beta 4A class IVa	Cytoplasm	other	docetaxel/gemcitabine/vincris	-2,125	3.08×10^-02
ENSG00000176014.12	TUBB6	tubulin beta 6 class V	Cytoplasm	other		1,596	8.35×10^-04
ENSG00000086712.12	TXLNG	taxilin gamma	Nucleus	other		1,916	1.33×10^-03
ENSG00000239264.8	TXNDC5	thioredoxin domain containing 5	Cytoplasm	enzyme		2,163	7.80×10^-05
ENSG00000176890.15	TYMS	thymidylate synthetase	Nucleus	enzyme	pemetrexed, capecitabine/ter	1,986	1.47×10^-02
ENSG00000278774.1	U2		Other	other		9,433	7.21×10^-03
ENSG00000137831.14	UACA	uveal autoantigen with coiled-coil domains and ankyrin repeats	Cytoplasm	other		1,446	1.21×10^-10
ENSG00000117143.13	UAP1	UDP-N-acetylglucosamine pyrophosphorylase 1	Nucleus	enzyme		1,929	3.90×10^-02
ENSG00000170315.13	UBB	ubiquitin B	Cytoplasm	enzyme		2,017	1.30×10^-06
ENSG00000150991.14	UBC	ubiquitin C	Cytoplasm	enzyme		1,062	1.11×10^-02
ENSG00000131508.15	UBE2D2	ubiquitin conjugating enzyme E2 D2	Cytoplasm	enzyme		1,372	4.83×10^-02
ENSG00000132388.12	UBE2G1	ubiquitin conjugating enzyme E2 G1	Cytoplasm	enzyme		1,531	6.13×10^-05
ENSG00000104517.12	UBR5	ubiquitin protein ligase E3 component n-recognin 5	Nucleus	enzyme		1,344	3.37×10^-02
ENSG00000233007.1	UBTFL11	UBTF like 11 (pseudogene)	Other	other		1,658	4.38×10^-02
ENSG00000162191.13	UBXN1	UBX domain protein 1	Cytoplasm	other		1,407	4.35×10^-04
ENSG00000135220.10	UGT2A3	UDP glucuronosyltransferase family 2 member A3	Other	enzyme		2,445	5.58×10^-03
ENSG00000147854.16	UHRF2	ubiquitin like with PHD and ring finger domains 2	Nucleus	enzyme		1,028	9.84×10^-03
ENSG00000233392.5	UICLM	up-regulated in colorectal cancer liver metastasis	Other	other		-3,447	3.81×10^-04
ENSG00000184385.2	UMODL1-AS1	UMODL1 antisense RNA 1	Other	other		-2,309	1.02×10^-02
ENSG00000198722.12	UNC13B	unc-13 homolog B	Cytoplasm	other		1,631	2.62×10^-03
ENSG00000140553.16	UNC45A	unc-45 myosin chaperone A	Plasma Membrane	other		-1,122	4.72×10^-02
ENSG00000115446.11	UNC50	unc-50 inner nuclear membrane RNA binding protein	Cytoplasm	other		1,489	5.03×10^-03
ENSG00000204922.4	UQCC3	ubiquinol-cytochrome c reductase complex assembly factor 3	Extracellular Space	other		-1,789	2.18×10^-02
ENSG00000140740.10	UQCRC2	ubiquinol-cytochrome c reductase core protein 2	Cytoplasm	enzyme		1,112	4.32×10^-02
ENSG00000105176.17	URI1	URI1 prefoldin like chaperone	Nucleus	transcription regulator		1,741	1.94×10^-03
ENSG00000233327.10	USP32P2	ubiquitin specific peptidase 32 pseudogene 2	Other	other		1,816	1.81×10^-05
ENSG00000118369.12	USP35	ubiquitin specific peptidase 35	Extracellular Space	peptidase		-1,144	1.15×10^-02
ENSG00000170236.14	USP50	ubiquitin specific peptidase 50	Other	peptidase		5,389	6.97×10^-03
ENSG00000132467.3	UTP3	UTP3 small subunit processome component	Nucleus	other		-1,231	1.64×10^-02
ENSG00000152818.18	UTRN	utrophin	Plasma Membrane	transmembrane receptor		1,379	5.64×10^-04
ENSG00000049245.12	VAMP3	vesicle associated membrane protein 3	Plasma Membrane	other		1,65	3.36×10^-02
ENSG00000117533.14	VAMP4	vesicle associated membrane protein 4	Cytoplasm	other		1,777	3.78×10^-04
ENSG00000204394.12	VARS1	valyl-tRNA synthetase 1	Cytoplasm	enzyme		2,478	5.52×10^-04
ENSG00000165637.13	VDAC2	voltage dependent anion channel 2	Cytoplasm	ion channel		1,475	4.82×10^-02
ENSG00000078668.13	VDAC3	voltage dependent anion channel 3	Cytoplasm	ion channel		2,531	4.91×10^-03
ENSG00000112715.20	VEGFA	vascular endothelial growth factor A	Extracellular Space	growth factor	bevacizumab/capecitabine, b	20,43	0.00×10^00
ENSG00000173511.9	VEGFB	vascular endothelial growth factor B	Extracellular Space	growth factor	aflibercept, aflibercept/irinote	2,843	7.94×10^-07
ENSG00000026025.13	VIM	vimentin	Cytoplasm	other		2,942	1.20×10^-44
ENSG00000106018.13	VIPR2	vasoactive intestinal peptide receptor 2	Plasma Membrane	G-protein coupled receptor		-2,157	2.58×10^-02
ENSG00000197617.6	VN1R5	vomeronasal 1 receptor 5 (gene/pseudogene)	Other	other		-3,657	3.77×10^-02
ENSG00000093134.13	VNN3	vanin 3	Extracellular Space	enzyme		-1,289	3.27×10^-02
ENSG00000129003.15	VPS13C	vacuolar protein sorting 13 homolog C	Cytoplasm	other		2,238	1.18×10^-07
ENSG00000132821.11	VSTM2L	V-set and transmembrane domain containing 2 like	Extracellular Space	other		3,325	8.26×10^-09
ENSG00000199990.1	VTRNA1-1	vault RNA 1-1	Other	other		7,582	1.30×10^-09
ENSG00000202111.1	VTRNA1-2	vault RNA 1-2	Other	other		5,391	1.04×10^-03
ENSG00000146556.14	WASH2P	WASP family homolog 2, pseudogene	Other	other		-1,986	8.61×10^-04
ENSG00000182484.14	WASH6P	WASP family homolog 6, pseudogene	Cytoplasm	other		-2,068	7.86×10^-05
ENSG00000071127.16	WDR1	WD repeat domain 1	Extracellular Space	other		1,28	2.17×10^-04
ENSG00000167716.18	WDR81	WD repeat domain 81	Plasma Membrane	other		-1,098	2.46×10^-02
ENSG00000095397.13	WHRN	whirlin	Plasma Membrane	other		-1,37	1.57×10^-02
ENSG00000188064.9	WNT7B	Wnt family member 7B	Extracellular Space	other		-2,337	2.86×10^-02
ENSG00000124535.15	WRNIP1	WRN helicase interacting protein 1	Nucleus	enzyme		-1,638	1.77×10^-02
ENSG00000047644.17	WWC3	WWC family member 3	Cytoplasm	other		1,229	3.18×10^-02
ENSG00000018408.14	WWTR1	WW domain containing transcription regulator 1	Nucleus	transcription regulator		1,398	1.46×10^-03
ENSG00000101966.12	XIAP	X-linked inhibitor of apoptosis	Cytoplasm	enzyme	XIAP antisense oligonucleoti	1,091	1.24×10^-02
ENSG00000182489.8	XKRX	XK related X-linked	Other	other		1,621	2.07×10^-02
ENSG00000079246.15	XRCC5	X-ray repair cross complementing 5	Nucleus	enzyme		1,862	1.99×10^-07
ENSG00000234825.3	XRCC6P2	X-ray repair cross complementing 6 pseudogene 2	Other	other		2,317	2.70×10^-03
ENSG00000199204.1	Y_RNA		Other	other		3,614	1.65×10^-02
ENSG00000065978.17	YBX1	Y-box binding protein 1	Nucleus	transcription regulator		3,272	6.53×10^-28
ENSG00000060138.12	YBX3	Y-box binding protein 3	Nucleus	transcription regulator		1,479	1.63×10^-07
ENSG00000185728.16	YTHDF3	YTH N6-methyladenosine RNA binding protein 3	Cytoplasm	other		1,027	3.56×10^-02
ENSG00000108953.16	YWHAE	tyrosine 3-monooxygenase/tryptophan 5-monooxygenase activation protein epsilon	Cytoplasm	other		1,663	3.60×10^-06
ENSG00000170027.6	YWHAG	tyrosine 3-monooxygenase/tryptophan 5-monooxygenase activation protein gamma	Cytoplasm	other		1,021	1.47×10^-03
ENSG00000134308.13	YWHAQ	tyrosine 3-monooxygenase/tryptophan 5-monooxygenase activation protein theta	Cytoplasm	other		1,936	4.31×10^-04
ENSG00000177485.6	ZBTB33	zinc finger and BTB domain containing 33	Nucleus	transcription regulator		1,863	7.51×10^-06
ENSG00000119574.12	ZBTB45	zinc finger and BTB domain containing 45	Nucleus	transcription regulator		-2,726	4.86×10^-02
ENSG00000065548.17	ZC3H15	zinc finger CCCH-type containing 15	Cytoplasm	translation regulator		2,256	1.82×10^-04
ENSG00000174460.3	ZCCHC12	zinc finger CCHC-type containing 12	Nucleus	transcription regulator		2,078	2.98×10^-02

ENSG00000139168.7	ZCRB1	zinc finger CCHC-type and RNA binding motif containing 1	Nucleus	other		1,709	4.68×10^-02
ENSG00000160446.18	ZDHHC12	zinc finger DHHC-type palmitoyltransferase 12	Cytoplasm	enzyme		-1,118	1.65×10^-02
ENSG00000104219.12	ZDHHC2	zinc finger DHHC-type palmitoyltransferase 2	Nucleus	enzyme		1,788	3.37×10^-02
ENSG00000148516.21	ZEB1	zinc finger E-box binding homeobox 1	Nucleus	transcription regulator		1,006	3.73×10^-06
ENSG00000196867.7	ZFP28	ZFP28 zinc finger protein	Nucleus	transcription regulator		1,042	3.50×10^-02
ENSG00000181007.8	ZFP82	ZFP82 zinc finger protein	Nucleus	transcription regulator		1,694	1.48×10^-03
ENSG00000179588.8	ZFPM1	zinc finger protein, FOG family member 1	Nucleus	transcription regulator		1,728	6.50×10^-05
ENSG00000188070.9	ZFTA	zinc finger translocation associated	Other	other		-1,245	2.15×10^-02
ENSG00000166432.14	ZMAT1	zinc finger matrin-type 1	Nucleus	other		1,43	1.22×10^-02
ENSG00000100319.12	ZMAT5	zinc finger matrin-type 5	Nucleus	other		-2,451	5.24×10^-03
ENSG00000196247.11	ZNF107	zinc finger protein 107	Nucleus	transcription regulator		1,788	2.45×10^-03
ENSG00000196646.11	ZNF136	zinc finger protein 136	Nucleus	transcription regulator		1,895	7.41×10^-04
ENSG00000167384.10	ZNF180	zinc finger protein 180	Nucleus	transcription regulator		2,459	1.40×10^-02
ENSG00000136870.10	ZNF189	zinc finger protein 189	Nucleus	transcription regulator		2,401	5.19×10^-04
ENSG00000175395.15	ZNF25	zinc finger protein 25	Nucleus	transcription regulator		1,745	3.01×10^-02
ENSG00000056277.15	ZNF280C	zinc finger protein 280C	Nucleus	transcription regulator		2,391	4.07×10^-03
ENSG00000223910.1	ZNF32-AS3	ZNF32 antisense RNA 3	Other	other		2,655	1.54×10^-02
ENSG00000225614.2	ZNF469	zinc finger protein 469	Nucleus	transcription regulator		-1,421	4.13×10^-03
ENSG00000177853.14	ZNF518A	zinc finger protein 518A	Other	other		1,209	2.21×10^-02
ENSG00000118156.12	ZNF541	zinc finger protein 541	Nucleus	transcription regulator		-3,086	1.51×10^-02
ENSG00000188785.11	ZNF548	zinc finger protein 548	Other	other		1,202	2.92×10^-02
ENSG00000102870.5	ZNF629	zinc finger protein 629	Nucleus	transcription regulator		-1,117	8.73×10^-03
ENSG00000167395.10	ZNF646	zinc finger protein 646	Nucleus	transcription regulator		-1,222	2.67×10^-04
ENSG00000179195.15	ZNF664	zinc finger protein 664	Nucleus	transcription regulator		1,247	3.03×10^-03
ENSG00000197472.14	ZNF695	zinc finger protein 695	Nucleus	transcription regulator		2,223	4.07×10^-03
ENSG00000242779.6	ZNF702P	zinc finger protein 702, pseudogene	Nucleus	other		2,038	3.39×10^-02
ENSG00000227124.8	ZNF717	zinc finger protein 717	Nucleus	transcription regulator		1,16	3.75×10^-02
ENSG00000268696.1	ZNF723	zinc finger protein 723	Other	other		2,937	3.55×10^-02
ENSG00000197779.13	ZNF81	zinc finger protein 81	Nucleus	transcription regulator		1,392	1.74×10^-02
ENSG00000152475.6	ZNF837	zinc finger protein 837	Other	other		-3,71	3.53×10^-02
ENSG00000159904.11	ZNF890P	zinc finger protein 890, pseudogene	Other	other		-3,631	4.08×10^-02
ENSG00000213988.9	ZNF90	zinc finger protein 90	Nucleus	transcription regulator		1,134	1.53×10^-02
ENSG00000106400.11	ZNHIT1	zinc finger HIT-type containing 1	Nucleus	other		-1,075	2.30×10^-02
ENSG00000169249.12	ZRSR2	zinc finger CCCH-type, RNA binding motif and serine/arginine rich 2	Nucleus	other		1,346	4.82×10^-02
ENSG00000158691.14	ZSCAN12	zinc finger and SCAN domain containing 12	Nucleus	transcription regulator		1,303	3.96×10^-03
ENSG00000214655.10	ZSWIM8	zinc finger SWIM-type containing 8	Extracellular Space	other		-1,025	5.36×10^-03
ENSG00000198205.6	ZXDA	zinc finger X-linked duplicated A	Other	transcription regulator		1,851	7.39×10^-04

Suppl. Table 3: Deregulated genes in extracellular vesicles secreted from CPCs after LNP treatment

Ensembl ID	Symbol	Entrez Gene Name	Location	Type(s)	logFC	FDR	Drug(s)
ENSG00000124574.14	ABCC10	ATP binding cassette subfamily C member 10	Plasma Membrane	transporter	3,001	3,18×10^-06	
ENSG00000204574.12	ABCF1	ATP binding cassette subfamily F member 1	Cytoplasm	transporter	-1,423	1,23×10^-02	
ENSG00000163995.18	ABLIM2	actin binding LIM protein family member 2	Cytoplasm	other	5,262	1,24×10^-18	
ENSG00000269026.2	AC0030067		Other	other	3,473	1,63×10^-02	
ENSG00000242073.2	AC0060147		Other	other	4,125	2,39×10^-02	
ENSG00000229379.1	AC0060411		Other	other	5,489	3,29×10^-03	
ENSG00000236231.1	AC0170482		Other	other	9,299	2,30×10^-27	
ENSG00000267740.5	AC02459212		Other	other	8,075	2,81×10^-03	
ENSG00000229642.1	AC0271191		Other	other	7,252	8,90×10^-03	
ENSG00000255495.1	AC145124.2		Other	other	3,056	9,41×10^-04	
ENSG00000131584.18	ACAP3	ArfGAP with coiled-coil, ankyrin repeat and PH domains 3	Nucleus	transcription regulator	1,474	2,88×10^-02	
ENSG00000100813.14	ACIN1	apoptotic chromatin condensation inducer 1	Nucleus	enzyme	-1,069	2,76×10^-03	
ENSG00000107796.12	ACTA2	actin alpha 2, smooth muscle	Cytoplasm	other	2,534	5,61×10^-13	
ENSG00000159251.6	ACTC1	actin alpha cardiac muscle 1	Cytoplasm	enzyme	2,978	1,75×10^-24	
ENSG00000178631.7	ACTG1P1	actin gamma 1 pseudogene 1	Other	other	4,284	2,72×10^-02	
ENSG00000077522.12	ACTN2	actinin alpha 2	Nucleus	transcription regulator	2,024	1,50×10^-03	
ENSG00000197859.9	ADAMTSL2	ADAMTS like 2	Extracellular Space	other	6,576	2,73×10^-76	
ENSG00000185736.15	ADARB2	adenosine deaminase RNA specific B2 (inactive)	Nucleus	enzyme	6,293	1,61×10^-08	
ENSG00000162618.12	ADGRL4	adhesion G protein-coupled receptor L4	Plasma Membrane	G-protein coupled receptor	12,641	3,44×10^-31	
ENSG00000130396.20	AFDN	afadin, adherens junction formation factor	Nucleus	other	-1,152	9,89×10^-03	
ENSG00000260994.1	AGGF1P7	angiogenic factor with G-patch and FHA domains 1 pseudogene 7	Other	other	8,994	1,24×10^-02	
ENSG00000162482.4	AKR7A3	aldo-keto reductase family 7 member A3	Cytoplasm	enzyme	5,295	1,13×10^-03	
ENSG00000253710.2	ALG11	ALG11 alpha-1,2-mannosyltransferase	Cytoplasm	enzyme	2,615	2,64×10^-02	
ENSG00000214274.9	ANG	angiogenin	Extracellular Space	enzyme	5,087	5,46×10^-03	
ENSG00000148677.6	ANKRD1	ankyrin repeat domain 1	Cytoplasm	transcription regulator	1,318	5,47×10^-08	
ENSG00000106013.12	ANKRD7	ankyrin repeat domain 7	Nucleus	transcription regulator	7,300	2,79×10^-02	
ENSG00000160746.12	ANO10	anoctamin 10	Plasma Membrane	ion channel	2,174	3,57×10^-02	
ENSG00000136938.8	ANP32B	acidic nuclear phosphoprotein 32 family member B	Nucleus	other	-1,136	3,18×10^-06	
ENSG00000182718.16	ANXA2	annexin A2	Plasma Membrane	other	-1,775	2,88×10^-02	
ENSG00000243478.7	AOX2P	aldehyde oxidase 2, pseudogene	Other	other	11,407	4,67×10^-61	
ENSG00000238220.1	AP00027564		Other	other	8,994	1,24×10^-02	
ENSG00000279586.1	AP0007111		Other	other	4,229	2,70×10^-02	
ENSG00000177879.14	AP3S1	adaptor related protein complex 3 subunit sigma 1	Cytoplasm	transporter	2,486	5,17×10^-07	
ENSG00000084674.13	APOB	apolipoprotein B	Extracellular Space	transporter	2,780	1,44×10^-03	mipomersen
ENSG00000267467.3	APOC4	apolipoprotein C4	Extracellular Space	transporter	7,300	2,79×10^-02	
ENSG00000006740.16	ARHGAP44	Rho GTPase activating protein 44	Cytoplasm	other	2,328	3,88×10^-02	
ENSG00000116584.17	ARHGEF2	Rho/Rac guanine nucleotide exchange factor 2	Cytoplasm	other	-3,596	4,79×10^-02	
ENSG00000116017.10	ARID3A	AT-rich interaction domain 3A	Nucleus	transcription regulator	1,229	1,09×10^-02	
ENSG00000100299.17	ARSA	arylsulfatase A	Cytoplasm	enzyme	7,174	2,12×10^-82	
ENSG00000256167.1	ATF4P4	activating transcription factor 4 pseudogene 4	Other	other	3,933	3,04×10^-02	
ENSG00000144848.10	ATG3	autophagy related 3	Cytoplasm	enzyme	1,630	4,57×10^-02	
ENSG00000101844.17	ATG4A	autophagy related 4A cysteine peptidase	Cytoplasm	peptidase	3,636	1,19×10^-02	
ENSG00000124406.16	ATP8A1	ATPase phospholipid transporting 8A1	Cytoplasm	transporter	2,432	3,06×10^-02	
ENSG00000085224.20	ATRX	ATRX chromatin remodeler	Nucleus	transcription regulator	-1,474	2,39×10^-02	
ENSG00000175711.8	B3GNTL1	UDP-GlcNAc:betaGal beta-1,3-N-acetylglucosaminyltransferase like 1	Other	enzyme	8,698	1,25×10^-11	
ENSG00000099968.17	BCL2L13	BCL2 like 13	Cytoplasm	other	1,783	4,73×10^-02	
ENSG00000185963.13	BICD2	BICD cargo adaptor 2	Cytoplasm	other	1,194	2,61×10^-02	
ENSG00000138696.10	BMPR1B	bone morphogenetic protein receptor type 1B	Plasma Membrane	kinase	4,375	2,88×10^-02	LDN-193189
ENSG00000261236.5	BOP1	BOP1 ribosomal biogenesis factor	Nucleus	other	1,769	6,20×10^-03	
ENSG00000196544.7	BORCS6	BLOC-1 related complex subunit 6	Cytoplasm	other	2,620	1,47×10^-03	
ENSG00000171634.16	BPTF	bromodomain PHD finger transcription factor	Nucleus	transcription regulator	-1,122	1,60×10^-02	
ENSG00000176605.7	C14orf177	chromosome 14 putative open reading frame 177	Other	other	11,178	3,68×10^-17	
ENSG00000235034.6	C19orf81	chromosome 19 open reading frame 81	Other	other	4,196	4,99×10^-02	
ENSG00000160298.17	C21orf58	chromosome 21 open reading frame 58	Other	other	4,201	2,28×10^-67	
ENSG00000063180.8	CA11	carbonic anhydrase 11	Extracellular Space	enzyme	-3,821	3,00×10^-02	
ENSG00000177469.12	CAVIN1	caveolae associated protein 1	Nucleus	transcription regulator	1,361	6,74×10^-03	
ENSG00000160200.17	CBS/CBSL	cystathionine beta-synthase	Cytoplasm	enzyme	-2,750	2,40×10^-02	
ENSG00000167131.16	CCDC103	coiled-coil domain containing 103	Cytoplasm	other	1,795	5,03×10^-07	
ENSG00000168491.9	CCDC110	coiled-coil domain containing 110	Nucleus	other	4,859	1,49×10^-02	
ENSG00000164989.15	CCDC171	coiled-coil domain containing 171	Other	other	1,842	6,00×10^-03	
ENSG00000151838.11	CCDC175	coiled-coil domain containing 175	Other	other	8,397	2,80×10^-36	
ENSG00000105383.14	CD33	CD33 molecule	Plasma Membrane	other	4,161	5,47×10^-03	gemtuzumab ozoga
ENSG00000164045.11	CDC25A	cell division cycle 25A	Nucleus	phosphatase	1,759	2,17×10^-02	
ENSG00000128536.15	CDHR3	cadherin related family member 3	Plasma Membrane	other	3,453	3,18×10^-02	
ENSG00000008128.22	CDK11A	cyclin dependent kinase 11A	Nucleus	kinase	-3,911	2,38×10^-02	
ENSG00000226020.4	CDK2AP2P3	cyclin dependent kinase 2 associated protein 2 pseudogene 3	Other	other	7,300	2,79×10^-02	
ENSG00000124762.13	CDKN1A	cyclin dependent kinase inhibitor 1A	Nucleus	kinase	1,271	2,07×10^-02	
ENSG00000147883.10	CDKN2B	cyclin dependent kinase inhibitor 2B	Nucleus	transcription regulator	1,745	2,79×10^-03	
ENSG00000218739.9	CEBPZOS	CEBPZ opposite strand	Other	other	6,478	1,93×10^-43	
ENSG00000172831.11	CES2	carboxylesterase 2	Cytoplasm	enzyme	2,629	2,91×10^-05	
ENSG00000206530.8	CFAP44	cilia and flagella associated protein 44	Extracellular Space	peptidase	11,337	7,49×10^-101	
ENSG00000170004.16	CHD3	chromodomain helicase DNA binding protein 3	Nucleus	enzyme	-1,906	6,83×10^-03	
ENSG00000129749.3	CHRNA10	cholinergic receptor nicotinic alpha 10 subunit	Plasma Membrane	transmembrane receptor	6,498	8,58×10^-06	succinylcholine, rap
ENSG00000174343.5	CHRNA9	cholinergic receptor nicotinic alpha 9 subunit	Plasma Membrane	transmembrane receptor	6,088	2,35×10^-14	ABT-089, isoflurane
ENSG00000013297.10	CLDN11	claudin 11	Plasma Membrane	other	2,604	4,89×10^-02	
ENSG00000182372.7	CLN8	CLN8 transmembrane ER and ERGIC protein	Cytoplasm	other	2,410	1,44×10^-02	
ENSG00000171603.16	CLSTN1	calsyntenin 1	Plasma Membrane	other	-4,180	1,28×10^-02	
ENSG00000176571.11	CNBD1	cyclic nucleotide binding domain containing 1	Other	other	9,126	3,91×10^-03	
ENSG00000213854.3	CNN2P6	calponin 2 pseudogene 6	Other	other	5,438	2,14×10^-03	
ENSG00000111799.20	COL12A1	collagen type XII alpha 1 chain	Extracellular Space	other	4,409	2,09×10^-24	collagenase
ENSG00000108821.13	COL1A1	collagen type I alpha 1 chain	Extracellular Space	other	1,450	9,63×10^-11	collagenase
ENSG00000188517.14	COL25A1	collagen type XXV alpha 1 chain	Cytoplasm	other	2,852	9,23×10^-06	collagenase
ENSG00000168542.12	COL3A1	collagen type III alpha 1 chain	Extracellular Space	other	2,055	3,67×10^-11	collagenase
ENSG00000206384.10	COL6A6	collagen type VI alpha 6 chain	Extracellular Space	other	3,429	1,37×10^-04	
ENSG00000049089.13	COL9A2	collagen type IX alpha 2 chain	Extracellular Space	other	-3,100	9,79×10^-03	collagenase
ENSG00000110442.11	COMMD9	COMM domain containing 9	Cytoplasm	other	4,324	1,09×10^-05	
ENSG00000115520.8	COQ10B	coenzyme Q10B	Cytoplasm	other	4,171	6,31×10^-19	
ENSG00000240230.5	COX19	cytochrome c oxidase assembly factor COX19	Cytoplasm	other	2,686	1,01×10^-04	
ENSG00000158525.15	CPA5	carboxypeptidase A5	Extracellular Space	peptidase	5,405	5,30×10^-03	
ENSG00000226321.5	CROCC2	ciliary rootlet coiled-coil, rootletin family member 2	Other	other	4,897	6,04×10^-03	
ENSG00000164796.17	CSMD3	CUB and Sushi multiple domains 3	Plasma Membrane	enzyme	2,731	4,78×10^-02	
ENSG00000159176.13	CSRP1	cysteine and glycine rich protein 1	Nucleus	other	1,910	3,82×10^-03	
ENSG00000227519.1	CTA_342B111		Other	other	9,450	1,07×10^-04	
ENSG00000257639.1	CTB_31N192		Other	other	13,033	2,05×10^-24	
ENSG00000243829.1	CTB_33G101		Other	other	6,912	1,22×10^-02	
ENSG00000242858.1	CTC_484M21		Other	other	5,201	2,81×10^-03	
ENSG00000268362.5	CTD_2017D111		Other	other	4,157	3,26×10^-02	
ENSG00000269815.1	CTD_2278I104		Other	other	7,670	8,25×10^-09	
ENSG00000240627.1	CTD_2312P211		Other	other	10,652	6,53×10^-05	
ENSG00000251648.2	CTD_2353N241		Other	other	6,197	4,30×10^-04	
ENSG00000267303.1	CTD_2369P212		Other	other	8,994	1,24×10^-02	
ENSG00000260871.1	CTD_2373J61		Other	other	10,391	3,71×10^-02	
ENSG00000269053.1	CTD_2521M248		Other	other	5,498	4,36×10^-02	
ENSG00000279014.1	CTD_2651B208		Other	other	5,169	5,74×10^-15	
ENSG00000165168.7	CYBB	cytochrome b-245 beta chain	Cytoplasm	enzyme	8,210	5,82×10^-38	
ENSG00000008256.15	CYTH3	cytohesin 3	Cytoplasm	other	2,245	1,83×10^-02	

ENSG00000150401.14	DCUN1D2	defective in cullin neddylation 1 domain containing 2	Other	other	2,612	2,41×10^-02	
ENSG00000279928.1	DDX11L17	DEAD/H-box helicase 11 like 17 (pseudogene)	Other	other	5,344	2,44×10^-02	
ENSG00000100201.18	DDX17	DEAD-box helicase 17	Nucleus	enzyme	-1,585	4,60×10^-02	
ENSG00000108654.11	DDX5	DEAD-box helicase 5	Nucleus	enzyme	-1,124	2,81×10^-02	
ENSG00000131504.15	DIAPH1	diaphanous related formin 1	Plasma Membrane	other	-2,237	4,02×10^-02	
ENSG00000187775.16	DNAH17	dynein axonemal heavy chain 17	Cytoplasm	other	6,282	1,62×10^-10	
ENSG00000039139.9	DNAH5	dynein axonemal heavy chain 5	Cytoplasm	enzyme	7,630	4,00×10^-39	
ENSG00000187726.8	DNAJB13	DnaJ heat shock protein family (Hsp40) member B13	Cytoplasm	other	8,953	5,43×10^-53	
ENSG00000104129.9	DNAJC17	DnaJ heat shock protein family (Hsp40) member C17	Other	other	1,694	3,10×10^-02	
ENSG00000125170.10	DOK4	docking protein 4	Plasma Membrane	other	1,368	1,77×10^-05	
ENSG00000101134.11	DOK5	docking protein 5	Plasma Membrane	other	3,739	2,79×10^-03	
ENSG00000124205.15	EDN3	endothelin 3	Extracellular Space	other	4,666	4,80×10^-02	
ENSG00000232587.1	EEF1A1P3	eukaryotic translation elongation factor 1 alpha 1 pseudogene 3	Other	other	6,058	2,43×10^-05	
ENSG00000173442.11	EHBP1L1	EH domain binding protein 1 like 1	Other	other	2,615	4,57×10^-07	
ENSG00000103966.9	EHD4	EH domain containing 4	Plasma Membrane	enzyme	3,053	1,21×10^-15	
ENSG00000128829.11	EIF2AK4	eukaryotic translation initiation factor 2 alpha kinase 4	Cytoplasm	kinase	2,012	2,45×10^-06	GCN2iA
ENSG00000205609.12	EIF3CL	eukaryotic translation initiation factor 3 subunit C like	Other	other	-4,396	1,09×10^-02	
ENSG00000158417.10	EIF5B	eukaryotic translation initiation factor 5B	Cytoplasm	translation regulator	-1,021	4,02×10^-02	
ENSG00000062598.17	ELMO2	engulfment and cell motility 2	Cytoplasm	other	1,768	3,83×10^-02	
ENSG00000229919.3	ELOCP3	elongin C pseudogene 3	Other	other	9,687	5,34×10^-08	
ENSG00000197774.12	EME2	essential meiotic structure-specific endonuclease subunit 2	Other	other	3,259	4,32×10^-09	
ENSG00000163064.6	EN1	engrailed homeobox 1	Nucleus	transcription regulator	3,468	1,63×10^-03	
ENSG00000120658.12	ENOX1	ecto-NOX disulfide-thiol exchanger 1	Plasma Membrane	enzyme	3,739	5,42×10^-05	
ENSG00000163508.12	EOMES	eomesodermin	Nucleus	transcription regulator	2,299	1,57×10^-04	
ENSG00000116016.13	EPAS1	endothelial PAS domain protein 1	Nucleus	transcription regulator	1,459	2,71×10^-02	PT-2385, belzutifar
ENSG00000142627.12	EPHA2	EPH receptor A2	Plasma Membrane	kinase	-3,758	3,14×10^-02	afatinib/dasatinib, c
ENSG00000196411.9	EPHB4	EPH receptor B4	Plasma Membrane	kinase	-4,099	1,44×10^-02	tesevatinib, NVP-B
ENSG00000151491.12	EPS8	epidermal growth factor receptor pathway substrate 8	Plasma Membrane	peptidase	4,152	1,30×10^-02	
ENSG00000105722.9	ERF	ETS2 repressor factor	Nucleus	transcription regulator	1,695	1,34×10^-02	
ENSG00000120705.12	ETF1	eukaryotic translation termination factor 1	Cytoplasm	translation regulator	1,943	5,44×10^-09	
ENSG00000182197.10	EXT1	exostosin glycosyltransferase 1	Cytoplasm	enzyme	2,418	2,35×10^-12	
ENSG00000112319.17	EYA4	EYA transcriptional coactivator and phosphatase 4	Cytoplasm	phosphatase	7,178	5,26×10^-33	
ENSG00000103089.8	FA2H	fatty acid 2-hydroxylase	Cytoplasm	enzyme	5,823	3,21×10^-16	
ENSG00000169122.11	FAM110B	family with sequence similarity 110 member B	Cytoplasm	other	2,625	4,14×10^-04	
ENSG00000112584.13	FAM120B	family with sequence similarity 120B	Nucleus	other	1,795	1,63×10^-02	
ENSG00000204677.10	FAM153CP	protein FAM153C	Other	other	6,420	1,73×10^-02	
ENSG00000111879.18	FAM184A	family with sequence similarity 184 member A	Extracellular Space	other	-1,611	4,00×10^-02	
ENSG00000146067.15	FAM193B	family with sequence similarity 193 member B	Nucleus	other	-3,586	4,76×10^-02	
ENSG00000185112.5	FAM43A	family with sequence similarity 43 member A	Other	other	1,987	8,99×10^-03	
ENSG00000142530.10	FAM71E1	family with sequence similarity 71 member E1	Other	other	5,804	3,62×10^-10	
ENSG00000130244.12	FAM98C	family with sequence similarity 98 member C	Other	other	2,808	1,62×10^-08	
ENSG00000145982.11	FARS2	phenylalanyl-tRNA synthetase 2, mitochondrial	Cytoplasm	enzyme	2,405	1,27×10^-02	
ENSG00000099364.16	FBXL19	F-box and leucine rich repeat protein 19	Cytoplasm	enzyme	1,836	4,63×10^-07	
ENSG00000167196.13	FBXO22	F-box protein 22	Cytoplasm	enzyme	1,575	2,65×10^-02	
ENSG00000267673.6	FDX2	ferredoxin 2	Cytoplasm	transporter	2,321	5,74×10^-03	
ENSG00000249715.9	FER1L5	fer-1 like family member 5	Other	other	5,167	1,72×10^-06	
ENSG00000022267.16	FHL1	four and a half LIM domains 1	Cytoplasm	other	3,183	3,90×10^-02	
ENSG00000183386.9	FHL3	four and a half LIM domains 3	Plasma Membrane	other	5,142	2,06×10^-08	
ENSG00000176971.3	FIBIN	fin bud initiation factor homolog	Cytoplasm	other	5,737	1,04×10^-14	
ENSG00000109920.12	FNBP4	formin binding protein 4	Nucleus	other	2,520	3,89×10^-09	
ENSG00000065970.8	FOXJ2	forkhead box J2	Nucleus	transcription regulator	12,748	4,24×10^-28	
ENSG00000150893.10	FREM2	FRAS1 related extracellular matrix 2	Extracellular Space	other	-3,723	3,83×10^-02	
ENSG00000146013.10	GFRA3	GDNF family receptor alpha 3	Plasma Membrane	transmembrane receptor	5,500	1,50×10^-02	
ENSG00000104522.15	GFUS	GDP-L-fucose synthase	Plasma Membrane	enzyme	2,561	2,08×10^-08	
ENSG00000165113.12	GKAP1	G kinase anchoring protein 1	Cytoplasm	other	5,274	3,30×10^-43	
ENSG00000120063.9	GNA13	G protein subunit alpha 13	Plasma Membrane	enzyme	1,352	1,53×10^-02	
ENSG00000113384.13	GOLPH3	golgi phosphoprotein 3	Cytoplasm	other	1,942	2,63×10^-04	
ENSG00000062194.15	GPBP1	GC-rich promoter binding protein 1	Nucleus	transcription regulator	-3,591	4,85×10^-02	
ENSG00000185974.6	GRK1	G protein-coupled receptor kinase 1	Plasma Membrane	kinase	8,212	2,46×10^-05	
ENSG00000125388.19	GRK4	G protein-coupled receptor kinase 4	Plasma Membrane	kinase	3,373	3,14×10^-02	
ENSG00000125651.13	GTF2F1	general transcription factor IIF subunit 1	Nucleus	transcription regulator	-1,208	4,73×10^-02	
ENSG00000277053.4	GTF2IP1	general transcription factor IIi pseudogene 1	Other	other	-3,269	2,98×10^-02	
ENSG00000272196.2	H2AC18/H2AC19	H2A clustered histone 18	Nucleus	other	-5,368	2,78×10^-04	
ENSG00000183598.3	H3C13	H3 clustered histone 13	Nucleus	other	2,315	2,18×10^-03	
ENSG00000203852.3	H3C15	H3 clustered histone 15	Nucleus	other	-4,173	1,35×10^-02	
ENSG00000179101.5	H3P47		Other	other	7,860	2,85×10^-06	
ENSG00000084754.10	HADHA	hydroxyacyl-CoA dehydrogenase trifunctional multienzyme complex s	Cytoplasm	enzyme	2,160	1,07×10^-06	
ENSG00000138029.13	HADHB	hydroxyacyl-CoA dehydrogenase trifunctional multienzyme complex s	Cytoplasm	enzyme	2,824	2,10×10^-02	
ENSG00000164107.8	HAND2	heart and neural crest derivatives expressed 2	Nucleus	transcription regulator	1,357	4,48×10^-03	
ENSG00000188536.12	HBA1/HBA2	hemoglobin subunit alpha 2	Extracellular Space	transporter	10,962	8,96×10^-136	iron dextran, meflo
ENSG00000206172.8	HBA1/HBA2	hemoglobin subunit alpha 2	Extracellular Space	transporter	12,125	5,21×10^-16	iron dextran, meflo
ENSG00000196591.11	HDAC2	histone deacetylase 2	Nucleus	transcription regulator	-2,075	1,53×10^-02	theophylline, trame
ENSG00000227183.3	HDGFP1	heparin binding growth factor pseudogene 1	Other	other	4,392	5,15×10^-06	
ENSG00000165338.16	HECTD2	HECT domain E3 ubiquitin protein ligase 2	Cytoplasm	enzyme	2,186	3,23×10^-02	
ENSG00000173706.12	HEG1	heart development protein with EGF like domains 1	Plasma Membrane	other	1,414	9,67×10^-03	
ENSG00000051108.14	HERPUD1	homocysteine inducible ER protein with ubiquitin like domain 1	Cytoplasm	other	1,592	4,33×10^-02	
ENSG00000220557.1	HMGB1P13	high mobility group box 1 pseudogene 13	Other	other	4,669	9,24×10^-03	
ENSG00000164104.11	HMGB2	high mobility group box 2	Nucleus	transcription regulator	-1,073	1,43×10^-03	
ENSG00000237603.1	HMGB3P12	high mobility group box 3 pseudogene 12	Other	other	6,495	1,88×10^-05	
ENSG00000249439.1	HMGN1P14	high mobility group nucleosome binding domain 1 pseudogene 14	Other	other	10,466	2,54×10^-12	
ENSG00000198830.10	HMGN2	high mobility group nucleosomal binding domain 2	Nucleus	other	-1,747	1,02×10^-02	
ENSG00000212769.5	HMGN2P8	high mobility group nucleosomal binding domain 2 pseudogene 8	Other	other	4,494	7,29×10^-03	
ENSG00000170144.18	HNRNPA3	heterogeneous nuclear ribonucleoprotein A3	Nucleus	other	-1,664	1,11×10^-02	
ENSG00000105323.16	HNRNPUL1	heterogeneous nuclear ribonucleoprotein U like 1	Nucleus	other	-1,539	2,21×10^-02	
ENSG00000172987.12	HPSE2	heparanase 2 (inactive)	Plasma Membrane	enzyme	6,329	1,11×10^-05	
ENSG00000165868.12	HSPA12A	heat shock protein family A (Hsp70) member 12A	Cytoplasm	other	2,438	4,57×10^-02	
ENSG00000132622.10	HSPA12B	heat shock protein family A (Hsp70) member 12B	Other	other	3,750	7,62×10^-04	
ENSG00000170606.13	HSPA4	heat shock protein family A (Hsp70) member 4	Cytoplasm	other	-1,461	4,68×10^-02	
ENSG00000232953.1	HSPA8P18	heat shock protein family A (Hsp70) member 8 pseudogene 18	Other	other	5,576	1,50×10^-02	
ENSG00000152778.8	IFIT5	interferon induced protein with tetratricopeptide repeats 5	Plasma Membrane	other	3,648	3,37×10^-02	
ENSG00000159128.14	IFNGR2	interferon gamma receptor 2	Plasma Membrane	transmembrane receptor	2,893	4,86×10^-05	interferon gamma-1
ENSG00000167244.17	IGF2	insulin like growth factor 2	Extracellular Space	growth factor	1,802	3,06×10^-17	MEDI-573, BI 8368
ENSG00000113302.4	IL12B	interleukin 12B	Extracellular Space	cytokine	10,156	3,85×10^-45	ustekinumab, meth
ENSG00000112706.11	IMPG1	interphotoreceptor matrix proteoglycan 1	Extracellular Space	other	6,964	2,61×10^-04	
ENSG00000068383.18	INPP5A	inositol polyphosphate-5-phosphatase A	Plasma Membrane	phosphatase	2,605	2,36×10^-03	
ENSG00000278685.4	IQCA1L	IQ motif containing with AAA domain 1 like	Other	other	4,982	4,31×10^-02	
ENSG00000173226.16	IQCB1	IQ motif containing B1	Extracellular Space	other	1,973	3,16×10^-02	
ENSG00000119669.4	IRF2BPL	interferon regulatory factor 2 binding protein like	Nucleus	enzyme	1,607	3,62×10^-02	
ENSG00000140968.10	IRF8	interferon regulatory factor 8	Nucleus	transcription regulator	3,781	2,39×10^-07	
ENSG00000016082.14	ISL1	ISL LIM homeobox 1	Nucleus	transcription regulator	1,973	2,00×10^-02	
ENSG00000138448.11	ITGAV	integrin subunit alpha V	Plasma Membrane	transmembrane receptor	1,570	4,73×10^-02	abciximab, intetum
ENSG00000123243.14	ITIH5	inter-alpha-trypsin inhibitor heavy chain 5	Plasma Membrane	other	5,833	9,48×10^-12	
ENSG00000143772.9	ITPKB	inositol-trisphosphate 3-kinase B	Cytoplasm	kinase	1,843	3,14×10^-02	GNF362
ENSG00000188385.11	JAKMIP3	Janus kinase and microtubule interacting protein 3	Other	other	3,327	4,14×10^-02	
ENSG00000140044.12	JDP2	Jun dimerization protein 2	Nucleus	transcription regulator	1,517	5,41×10^-03	
ENSG00000161999.11	JMJD8	jumonji domain containing 8	Cytoplasm	other	5,973	3,19×10^-110	
ENSG00000237556.1	KCND3-AS1	KCND3 antisense RNA 1	Other	other	11,596	1,03×10^-17	
ENSG00000180509.11	KCNE1	potassium voltage-gated channel subfamily E regulatory subunit 1	Plasma Membrane	ion channel	3,848	4,92×10^-02	nicorandil, amiodar
ENSG00000276289.4	KCNE1B	potassium voltage-gated channel subfamily E regulatory subunit 1B	Plasma Membrane	ion channel	6,768	5,89×10^-04	
ENSG00000171303.6	KCNK3	potassium two pore domain channel subfamily K member 3	Plasma Membrane	ion channel	5,293	6,00×10^-04	doxapram, amiodar
ENSG00000162687.16	KCNT2	potassium sodium-activated channel subfamily T member 2	Plasma Membrane	ion channel	3,058	2,16×10^-02	

ENSG00000100196.10	KDELR3	KDEL endoplasmic reticulum protein retention receptor 3	Cytoplasm	transporter	4,057	3,38×10^-03	
ENSG00000117245.12	KIF17	kinesin family member 17	Cytoplasm	transporter	3,743	1,34×10^-05	
ENSG00000129250.11	KIF1C	kinesin family member 1C	Cytoplasm	other	-1,056	3,30×10^-02	
ENSG00000126790.11	L3HYPDH	trans-L-3-hydroxyproline dehydratase	Other	enzyme	3,748	8,57×10^-04	
ENSG00000226440.7	LAMA4-AS1	LAMA4 antisense RNA 1	Other	other	4,920	5,61×10^-03	
ENSG00000131981.15	LGALS3	galectin 3	Extracellular Space	other	5,248	5,52×10^-18	GCS-100, GR-MD-
ENSG00000214269.3	LGMNP1	legumain pseudogene 1	Other	other	5,064	4,10×10^-05	
ENSG00000259527.2	LINC00052	long intergenic non-protein coding RNA 52	Other	other	5,688	3,73×10^-03	
ENSG00000184274.3	LINC00315	long intergenic non-protein coding RNA 315	Other	other	6,064	9,33×10^-04	
ENSG00000178977.3	LINC00324	long intergenic non-protein coding RNA 324	Other	other	2,173	1,20×10^-02	
ENSG00000281778.1	LINC00550	long intergenic non-protein coding RNA 550	Other	other	7,146	6,18×10^-05	
ENSG00000233395.1	LINC00841	long intergenic non-protein coding RNA 841	Other	other	3,551	2,48×10^-02	
ENSG00000229017.6	LINC01277	long intergenic non-protein coding RNA 1277	Other	other	5,892	3,37×10^-02	
ENSG00000273415.1	LINC02725		Other	other	11,933	1,66×10^-09	
ENSG00000071282.11	LMCD1	LIM and cysteine rich domains 1	Cytoplasm	transcription regulator	3,256	3,27×10^-08	
ENSG00000160789.19	LMNA	lamin A/C	Nucleus	other	1,321	3,04×10^-04	
ENSG00000266149.1	LOC100192426	uncharacterized LOC100192426	Other	other	11,843	7,15×10^-19	
ENSG00000255284.1	LOC171391	uncharacterized LOC171391	Other	other	4,510	2,06×10^-04	
ENSG00000134324.11	LPIN1	lipin 1	Nucleus	phosphatase	2,229	1,90×10^-08	
ENSG00000250462.8	LRRC37BP1	leucine rich repeat containing 37B pseudogene 1	Other	other	2,126	4,27×10^-02	
ENSG00000125872.7	LRRN4	leucine rich repeat neuronal 4	Plasma Membrane	other	2,176	1,54×10^-03	
ENSG00000108848.15	LUC7L3	LUC7 like 3 pre-mRNA splicing factor	Nucleus	other	-1,410	5,95×10^-05	
ENSG00000150556.16	LYPD6B	LY6/PLAUR domain containing 6B	Other	other	3,653	5,84×10^-05	
ENSG00000133315.10	MACROD1	mono-ADP ribosylhydrolase 1	Cytoplasm	enzyme	2,827	2,89×10^-03	
ENSG00000113648.16	MACROH2A1	macroH2A.1 histone	Nucleus	other	2,476	2,21×10^-44	
ENSG00000198934.4	MAGEE1	MAGE family member E1	Plasma Membrane	other	4,836	1,94×10^-08	
ENSG00000198162.12	MAN1A2	mannosidase alpha class 1A member 2	Cytoplasm	enzyme	1,849	2,03×10^-07	
ENSG00000175130.6	MARCKSL1	MARCKS like 1	Cytoplasm	other	-1,317	1,49×10^-02	
ENSG00000151224.12	MAT1A	methionine adenosyltransferase 1A	Cytoplasm	enzyme	6,427	1,90×10^-08	
ENSG00000112159.11	MDN1	midasin AAA ATPase 1	Nucleus	other	-1,555	5,54×10^-03	
ENSG00000159479.16	MED8	mediator complex subunit 8	Nucleus	other	1,902	4,32×10^-02	
ENSG00000176845.12	METRNL	meteorin like, glial cell differentiation regulator	Cytoplasm	other	2,592	4,97×10^-03	
ENSG00000165171.10	METTL27	methyltransferase like 27	Other	enzyme	4,944	4,19×10^-03	
ENSG00000185432.11	METTL7A	methyltransferase like 7A	Cytoplasm	other	3,119	2,79×10^-02	
ENSG00000197530.12	MIB2	MIB E3 ubiquitin protein ligase 2	Nucleus	transcription regulator	1,470	1,14×10^-02	
ENSG00000158747.13	MICOS10-NBL1/NBL1	NBL1, DAN family BMP antagonist	Nucleus	other	3,734	1,59×10^-04	
ENSG00000207605.2	mir-191	microRNA 191	Cytoplasm	microRNA	5,456	1,93×10^-03	
ENSG00000224141.5	MIR548XHG	MIR548X host gene	Other	other	14,066	9,71×10^-40	
ENSG00000273776.1	MIR6126	microRNA 6126	Cytoplasm	microRNA	4,911	2,22×10^-05	
ENSG00000227195.8	MIR663AHG	MIR663A host gene	Other	other	2,247	1,44×10^-13	
ENSG00000108788.11	MLX	MAX dimerization protein MLX	Nucleus	transcription regulator	3,899	2,87×10^-38	
ENSG00000169446.5	MMGT1	membrane magnesium transporter 1	Cytoplasm	transporter	3,328	9,79×10^-03	
ENSG00000243927.5	MRPS6	mitochondrial ribosomal protein S6	Cytoplasm	other	1,201	4,26×10^-02	
ENSG00000174099.10	MSRB3	methionine sulfoxide reductase B3	Cytoplasm	enzyme	2,929	4,80×10^-02	
ENSG00000198899.2	MT-ATP6	ATP synthase F0 subunit 6	Cytoplasm	transporter	-1,617	1,18×10^-03	
ENSG00000228253.1	MT-ATP8	ATP synthase F0 subunit 8	Cytoplasm	enzyme	-1,371	1,29×10^-02	
ENSG00000198804.2	MT-CO1	cytochrome c oxidase subunit I	Cytoplasm	enzyme	-1,273	8,65×10^-03	naproxen/sumatript
ENSG00000198712.1	MT-CO2	cytochrome c oxidase subunit II	Cytoplasm	enzyme	-1,170	2,94×10^-03	naproxen/sumatript
ENSG00000198938.2	MT-CO3	cytochrome c oxidase subunit III	Cytoplasm	enzyme	-1,182	3,14×10^-02	
ENSG00000198727.2	MT-CYB	cytochrome b	Cytoplasm	enzyme	-1,591	1,02×10^-05	atovaquone, atovac
ENSG00000198888.2	MT-ND1	NADH dehydrogenase subunit 1	Cytoplasm	enzyme	-1,601	8,01×10^-06	
ENSG00000198763.3	MT-ND2	NADH dehydrogenase subunit 2	Cytoplasm	enzyme	-1,651	3,54×10^-04	
ENSG00000198886.2	MT-ND4	NADH dehydrogenase subunit 4	Cytoplasm	enzyme	-1,543	1,67×10^-06	
ENSG00000198786.2	MT-ND5	NADH dehydrogenase subunit 5	Cytoplasm	enzyme	-1,465	2,44×10^-08	
ENSG00000211459.2	MT-RNR1	s-rRNA	Cytoplasm	other	-1,307	3,82×10^-02	
ENSG00000210151.2	MT-TS1	tRNA	Cytoplasm	other	2,721	1,22×10^-06	
ENSG00000248527.1	MTATP6P1	MT-ATP6 pseudogene 1	Other	other	-1,182	2,20×10^-02	
ENSG00000063601.16	MTMR1	myotubularin related protein 1	Cytoplasm	phosphatase	2,571	1,02×10^-02	
ENSG00000102043.15	MTMR8	myotubularin related protein 8	Nucleus	phosphatase	6,989	1,11×10^-04	
ENSG00000223431.1	MTND6P21	MT-ND6 pseudogene 21	Other	other	5,358	1,92×10^-02	
ENSG00000205592.13	MUC19	mucin 19, oligomeric	Cytoplasm	other	-3,988	2,38×10^-02	
ENSG00000162576.16	MXRA8	matrix remodeling associated 8	Cytoplasm	other	1,723	2,87×10^-02	
ENSG00000185697.16	MYBL1	MYB proto-oncogene like 1	Nucleus	transcription regulator	2,166	8,49×10^-09	
ENSG00000144821.9	MYH15	myosin heavy chain 15	Extracellular Space	other	3,674	2,03×10^-03	
ENSG00000197616.11	MYH6	myosin heavy chain 6	Cytoplasm	enzyme	5,034	5,32×10^-77	
ENSG00000198336.9	MYL4	myosin light chain 4	Cytoplasm	other	1,711	4,48×10^-03	
ENSG00000106631.8	MYL7	myosin light chain 7	Cytoplasm	enzyme	2,147	8,76×10^-07	
ENSG00000133454.15	MYO18B	myosin XVIIIB	Cytoplasm	other	3,495	3,33×10^-03	
ENSG00000141052.17	MYOCD	myocardin	Nucleus	transcription regulator	2,579	2,64×10^-06	
ENSG00000244754.8	N4BP2L2	NEDD4 binding protein 2 like 2	Nucleus	transcription regulator	8,926	5,33×10^-61	
ENSG00000177694.14	NAALADL2	N-acetylated alpha-linked acidic dipeptidase like 2	Nucleus	other	7,119	7,18×10^-11	
ENSG00000105402.7	NAPA	NSF attachment protein alpha	Cytoplasm	transporter	4,669	9,48×10^-93	
ENSG00000132780.16	NASP	nuclear autoantigenic sperm protein	Nucleus	other	-1,146	1,39×10^-03	
ENSG00000255358.1	NDUFAF2P2	NDUFAF2 pseudogene 2	Other	other	5,161	3,37×10^-02	
ENSG00000110400.10	NECTIN1	nectin cell adhesion molecule 1	Plasma Membrane	other	5,675	1,39×10^-187	
ENSG00000173848.18	NET1	neuroepithelial cell transforming 1	Nucleus	other	-2,411	2,89×10^-02	
ENSG00000131196.17	NFATC1	nuclear factor of activated T cells 1	Nucleus	transcription regulator	-3,842	2,81×10^-02	
ENSG00000187736.12	NHEJ1	non-homologous end joining factor 1	Nucleus	other	2,630	1,20×10^-02	
ENSG00000116962.14	NID1	nidogen 1	Extracellular Space	other	2,082	1,73×10^-02	
ENSG00000140807.5	NKD1	NKD inhibitor of WNT signaling pathway 1	Other	other	-3,982	1,88×10^-02	
ENSG00000171487.14	NLRP5	NLR family pyrin domain containing 5	Cytoplasm	other	6,149	5,71×10^-04	
ENSG00000158806.13	NPM2	nucleophosmin/nucleoplasmin 2	Nucleus	other	6,234	2,16×10^-11	
ENSG00000175745.11	NR2F1	nuclear receptor subfamily 2 group F member 1	Nucleus	ligand-dependent nuclear receptor	8,451	1,53×10^-11	
ENSG00000198435.3	NRARP	NOTCH regulated ankyrin repeat protein	Nucleus	transcription regulator	6,345	1,33×10^-53	
ENSG00000248008.2	NRAV	negative regulator of antiviral response	Other	other	2,959	8,93×10^-03	
ENSG00000118257.16	NRP2	neuropilin 2	Plasma Membrane	kinase	1,305	2,74×10^-03	
ENSG00000179915.20	NRXN1	neurexin 1	Plasma Membrane	transporter	3,448	8,09×10^-06	
ENSG00000143552.9	NUP210L	nucleoporin 210 like	Other	other	12,382	5,05×10^-69	
ENSG00000226328.6	NUP50-DT	NUP50 divergent transcript	Other	other	5,476	4,44×10^-03	
ENSG00000149635.2	OCSTAMP	osteoclast stimulatory transmembrane protein	Other	other	10,644	8,38×10^-106	
ENSG00000162745.10	OLFML2B	olfactomedin like 2B	Extracellular Space	other	3,624	2,86×10^-03	
ENSG00000276240.1	OR4E1	olfactory receptor family 4 subfamily E member 1	Plasma Membrane	other	5,870	4,32×10^-09	
ENSG00000128694.11	OSGEPL1	O-sialoglycoprotein endopeptidase like 1	Cytoplasm	peptidase	4,457	1,39×10^-02	
ENSG00000172939.8	OXSR1	oxidative stress responsive kinase 1	Nucleus	kinase	1,518	3,69×10^-02	
ENSG00000130669.17	PAK4	p21 (RAC1) activated kinase 4	Cytoplasm	kinase	2,159	1,57×10^-06	FRAX355, PF-3758
ENSG00000129116.17	PALLD	palladin, cytoskeletal associated protein	Plasma Membrane	other	1,676	1,03×10^-04	
ENSG00000111224.13	PARP11	poly(ADP-ribose) polymerase family member 11	Nucleus	enzyme	7,947	7,37×10^-199	
ENSG00000138650.8	PCDH10	protocadherin 10	Plasma Membrane	other	-4,812	1,98×10^-03	
ENSG00000156374.14	PCGF6	polycomb group ring finger 6	Nucleus	transcription regulator	2,318	7,01×10^-03	
ENSG00000099139.13	PCSK5	proprotein convertase subtilisin/kexin type 5	Extracellular Space	peptidase	1,605	1,75×10^-02	
ENSG00000244119.1	PDCL3P4	PDCL3 pseudogene 4	Other	other	5,365	6,43×10^-06	
ENSG00000065989.15	PDE4A	phosphodiesterase 4A	Cytoplasm	enzyme	4,506	3,34×10^-33	caffeine, pentoxifyll
ENSG00000138735.15	PDE5A	phosphodiesterase 5A	Cytoplasm	enzyme	3,311	2,55×10^-09	dyphylline, udenafil
ENSG00000156973.13	PDE6D	phosphodiesterase 6D	Cytoplasm	enzyme	4,791	6,92×10^-37	
ENSG00000170962.12	PDGFD	platelet derived growth factor D	Extracellular Space	growth factor	3,705	8,83×10^-03	
ENSG00000160209.18	PDXK	pyridoxal kinase	Cytoplasm	kinase	1,472	3,17×10^-02	
ENSG00000242265.5	PEG10	paternally expressed 10	Nucleus	other	-1,393	1,21×10^-03	
ENSG00000157911.9	PEX10	peroxisomal biogenesis factor 10	Cytoplasm	other	3,850	1,33×10^-19	
ENSG00000070087.13	PFN2	profilin 2	Cytoplasm	enzyme	1,458	1,02×10^-02	
ENSG00000176732.6	PFN4	profilin family member 4	Other	other	8,994	1,24×10^-02	
ENSG00000112419.14	PHACTR2	phosphatase and actin regulator 2	Other	other	-1,826	4,36×10^-02	

ENSG00000118482.11	PHF3	PHD finger protein 3	Nucleus	other	-4,583	3,75×10^-03	
ENSG00000125207.7	PIWIL1	piwi like RNA-mediated gene silencing 1	Cytoplasm	enzyme	8,202	1,84×10^-47	
ENSG00000166689.14	PLEKHA7	pleckstrin homology domain containing A7	Cytoplasm	other	1,569	4,28×10^-02	
ENSG00000120756.12	PLS1	plastin 1	Plasma Membrane	other	3,176	2,45×10^-02	
ENSG00000182013.17	PNMA8A	PNMA family member 8A	Other	other	2,702	1,60×10^-04	
ENSG00000105258.8	POLR2I	RNA polymerase II subunit I	Nucleus	transcription regulator	1,812	1,30×10^-02	
ENSG00000184486.8	POU3F2	POU class 3 homeobox 2	Nucleus	transcription regulator	4,265	5,18×10^-09	
ENSG00000173281.4	PPP1R3B	protein phosphatase 1 regulatory subunit 3B	Cytoplasm	phosphatase	4,766	1,34×10^-33	
ENSG00000138814.16	PPP3CA	protein phosphatase 3 catalytic subunit alpha	Cytoplasm	phosphatase	1,699	1,79×10^-02	voclosporin, tacroli
ENSG00000171132.13	PRKCE	protein kinase C epsilon	Cytoplasm	kinase	2,455	1,22×10^-02	ingenol mebutate, I
ENSG00000117523.15	PRRC2C	proline rich coiled-coil 2C	Cytoplasm	other	-1,675	5,94×10^-08	
ENSG00000150687.11	PRSS23	serine protease 23	Extracellular Space	peptidase	1,784	1,05×10^-02	
ENSG00000105227.14	PRX	periaxin	Nucleus	other	5,274	1,11×10^-28	
ENSG00000156011.16	PSD3	pleckstrin and Sec7 domain containing 3	Cytoplasm	other	2,930	1,17×10^-23	
ENSG00000174915.11	PTDSS2	phosphatidylserine synthase 2	Cytoplasm	enzyme	2,260	1,10×10^-03	
ENSG00000187514.14	PTMA	prothymosin alpha	Nucleus	other	-1,022	1,42×10^-07	
ENSG00000163629.12	PTPN13	protein tyrosine phosphatase non-receptor type 13	Cytoplasm	phosphatase	-1,494	1,24×10^-02	
ENSG00000196090.12	PTPRT	protein tyrosine phosphatase receptor type T	Plasma Membrane	phosphatase	3,453	3,98×10^-02	
ENSG00000177192.13	PUS1	pseudouridine synthase 1	Nucleus	enzyme	2,665	5,08×10^-10	
ENSG00000143545.8	RAB13	RAB13, member RAS oncogene family	Plasma Membrane	enzyme	-1,804	6,34×10^-03	
ENSG00000132698.13	RAB25	RAB25, member RAS oncogene family	Cytoplasm	enzyme	3,064	4,57×10^-03	
ENSG00000164520.11	RAET1E	retinoic acid early transcript 1E	Plasma Membrane	other	6,216	2,62×10^-11	
ENSG00000112183.14	RBM24	RNA binding motif protein 24	Cytoplasm	other	2,376	1,48×10^-02	
ENSG00000119707.13	RBM25	RNA binding motif protein 25	Nucleus	other	-1,240	1,21×10^-04	
ENSG00000144642.20	RBMS3	RNA binding motif single stranded interacting protein 3	Cytoplasm	other	2,325	4,73×10^-02	
ENSG00000170748.6	RBMXL2	RBMX like 2	Nucleus	other	3,626	6,12×10^-03	
ENSG00000175718.9	RBMXL3	RBMX like 3	Other	other	5,691	4,80×10^-02	
ENSG00000137710.14	RDX	radixin	Cytoplasm	other	-1,991	4,88×10^-03	
ENSG00000076344.15	RGS11	regulator of G protein signaling 11	Plasma Membrane	enzyme	5,130	6,24×10^-10	
ENSG00000158315.10	RHBDL2	rhomboid like 2	Plasma Membrane	peptidase	9,103	2,47×10^-112	
ENSG00000155366.16	RHOC	ras homolog family member C	Plasma Membrane	enzyme	-3,609	4,75×10^-02	
ENSG00000042062.11	RIPOR3	RIPOR family member 3	Other	other	2,735	1,73×10^-02	
ENSG00000200674.1	RN7SKP160		Other	other	5,892	3,37×10^-02	
ENSG00000243738.3	RN7SL181P	RNA, 7SL, cytoplasmic 181, pseudogene	Other	other	4,416	8,65×10^-04	
ENSG00000265753.2	RN7SL444P		Other	other	3,643	4,86×10^-02	
ENSG00000242894.3	RN7SL634P	RNA, 7SL, cytoplasmic 634, pseudogene	Other	other	5,947	8,69×10^-03	
ENSG00000241172.3	RN7SL70P	RNA, 7SL, cytoplasmic 70, pseudogene	Other	other	3,816	4,80×10^-02	
ENSG00000239748.3	RN7SL795P	RNA, 7SL, cytoplasmic 795, pseudogene	Other	other	3,998	3,79×10^-02	
ENSG00000275757.1	RNA5-8SN1	RNA, 5.8S ribosomal N1	Other	other	3,781	6,72×10^-07	
ENSG00000276700.1	RNA5-8SN4	RNA, 5.8S ribosomal N4	Other	other	6,725	1,70×10^-02	
ENSG00000201185.1	RNA5SP202	RNA, 5S ribosomal pseudogene 202	Other	other	2,257	6,97×10^-05	
ENSG00000252261.1	RNA5SP262	RNA, 5S ribosomal pseudogene 262	Other	other	2,385	6,67×10^-06	
ENSG00000200246.1	RNA5SP263		Other	other	4,009	1,28×10^-03	
ENSG00000202474.1	RNA5SP283	RNA, 5S ribosomal pseudogene 283	Other	other	5,127	1,37×10^-02	
ENSG00000199525.1	RNA5SP295	RNA, 5S ribosomal pseudogene 295	Other	other	6,418	1,20×10^-03	
ENSG00000222427.1	RNA5SP338	RNA, 5S ribosomal pseudogene 338	Other	other	4,419	4,63×10^-04	
ENSG00000272435.1	RNA5SP357	RNA, 5S ribosomal pseudogene 357	Other	other	4,130	2,40×10^-02	
ENSG00000222268.1	RNA5SP425	RNA, 5S ribosomal pseudogene 425	Other	other	5,118	4,73×10^-02	
ENSG00000200558.1	RNA5SP429	RNA, 5S ribosomal pseudogene 429	Other	other	3,914	2,22×10^-03	
ENSG00000252680.1	RNA5SP449	RNA, 5S ribosomal pseudogene 449	Other	other	2,771	2,16×10^-11	
ENSG00000251770.1	RNA5SP486	RNA, 5S ribosomal pseudogene 486	Other	other	4,412	1,32×10^-02	
ENSG00000145860.11	RNF145	ring finger protein 145	Other	other	2,200	3,29×10^-04	
ENSG00000185946.15	RNPC3	RNA binding region (RNP1, RRM) containing 3	Nucleus	other	2,382	1,88×10^-02	
ENSG00000206652.1	RNU1-1	RNA, U1 small nuclear 1	Other	other	-9,716	2,89×10^-11	
ENSG00000200340.1	RNU1-105P	RNA, U1 small nuclear 105, pseudogene	Other	other	4,605	6,51×10^-05	
ENSG00000199805.1	RNU1-134P	RNA, U1 small nuclear 134, pseudogene	Other	other	1,408	2,78×10^-02	
ENSG00000212609.1	RNU1-139P		Other	other	4,128	9,79×10^-28	
ENSG00000199629.1	RNU1-14P	RNA, U1 small nuclear 14, pseudogene	Other	other	2,472	1,72×10^-03	
ENSG00000202347.1	RNU1-16P	RNA, U1 small nuclear 16, pseudogene	Other	other	3,995	5,41×10^-41	
ENSG00000207005.1	RNU1-2	RNA, U1 small nuclear 2	Nucleus	other	7,113	1,33×10^-02	
ENSG00000200197.1	RNU1-21P	RNA, U1 small nuclear 21, pseudogene	Other	other	3,800	9,83×10^-06	
ENSG00000206596.1	RNU1-27P	RNA, U1 small nuclear 27, pseudogene	Other	other	4,671	2,92×10^-57	
ENSG00000206588.1	RNU1-28P	RNA, U1 small nuclear 28, pseudogene	Other	other	-8,069	1,69×10^-08	
ENSG00000207513.1	RNU1-3	RNA, U1 small nuclear 3	Other	other	-9,835	1,80×10^-10	
ENSG00000201119.1	RNU1-33P		Other	other	4,861	3,99×10^-33	
ENSG00000206624.1	RNU1-39P	RNA, U1 small nuclear 39, pseudogene	Other	other	2,125	9,10×10^-06	
ENSG00000207389.1	RNU1-4	RNA, U1 small nuclear 4	Nucleus	other	6,542	1,58×10^-36	
ENSG00000253000.1	RNU1-45P		Other	other	3,676	3,82×10^-16	
ENSG00000207154.1	RNU1-46P		Other	other	5,629	2,16×10^-24	
ENSG00000212170.1	RNU1-77P		Other	other	4,413	4,96×10^-13	
ENSG00000270103.3	RNU11		Other	other	2,173	1,42×10^-08	
ENSG00000276027.1	RNU12	RNA, U12 small nuclear	Nucleus	other	4,108	5,60×10^-48	
ENSG00000222985.1	RNU2-14P		Other	other	4,219	6,19×10^-37	
ENSG00000222328.1	RNU2-2P	RNA, U2 small nuclear 2, pseudogene	Nucleus	other	2,217	1,48×10^-29	
ENSG00000252604.1	RNU2-44P		Other	other	4,835	2,01×10^-19	
ENSG00000222426.1	RNU2-50P	RNA, U2 small nuclear 50, pseudogene	Other	other	3,271	1,23×10^-17	
ENSG00000222650.1	RNU2-70P	RNA, U2 small nuclear 70, pseudogene	Other	other	3,061	1,16×10^-02	
ENSG00000199568.1	RNU5A-1	RNA, U5A small nuclear 1	Other	other	2,200	2,03×10^-07	
ENSG00000200972.1	RNU5A-8P	RNA, U5A small nuclear 8, pseudogene	Other	other	2,236	2,32×10^-02	
ENSG00000200169.1	RNU5D-1	RNA, U5D small nuclear 1	Other	other	2,706	8,01×10^-13	
ENSG00000199347.1	RNU5E-1	RNA, U5E small nuclear 1	Other	other	2,997	5,14×10^-37	
ENSG00000202444.1	RNU5E-6P	RNA, U5E small nuclear 6, pseudogene	Other	other	2,403	2,94×10^-13	
ENSG00000207291.1	RNU6-30P	RNA, U6 small nuclear 30, pseudogene	Other	other	6,912	1,22×10^-02	
ENSG00000207349.1	RNVU1-17	RNA, variant U1 small nuclear 17	Other	other	5,396	4,39×10^-02	
ENSG00000206737.1	RNVU1-18	RNA, variant U1 small nuclear 18	Other	other	-8,093	3,53×10^-06	
ENSG00000275538.1	RNVU1-19	RNA, variant U1 small nuclear 19	Other	other	3,042	8,99×10^-03	
ENSG00000277918.1	RNVU1-28		Other	other	1,503	4,86×10^-05	
ENSG00000273768.1	RNVU1-29		Other	other	9,282	8,22×10^-63	
ENSG00000278099.1	RNVU1-2A	RNA, variant U1 small nuclear 2A	Other	other	2,379	9,01×10^-03	
ENSG00000206585.1	RNVU1-7	RNA, variant U1 small nuclear 7	Other	other	2,580	6,11×10^-09	
ENSG00000201098.1	RNY1	RNA, Ro60-associated Y1	Nucleus	other	2,148	5,49×10^-07	
ENSG00000202354.1	RNY3	RNA, Ro60-associated Y3	Other	other	1,578	2,15×10^-02	
ENSG00000185483.11	ROR1	receptor tyrosine kinase like orphan receptor 1	Plasma Membrane	kinase	1,660	1,62×10^-03	cirmtuzumab, VLS-
ENSG00000272980.3	RP11-517H2.6		Other	other	5,474	5,43×10^-06	
ENSG00000260729.1	RP11_106M32		Other	other	4,768	2,47×10^-03	
ENSG00000264643.1	RP11_118G232		Other	other	8,994	1,24×10^-02	
ENSG00000227200.1	RP11_121A143		Other	other	7,800	8,00×10^-06	
ENSG00000253496.2	RP11_13N121		Other	other	7,300	2,70×10^-02	
ENSG00000260483.2	RP11_151H21		Other	other	8,994	1,24×10^-02	
ENSG00000256569.1	RP11_173P155		Other	other	27,099	4,33×10^-10	
ENSG00000261076.1	RP11_179B156		Other	other	5,057	1,43×10^-07	
ENSG00000255439.6	RP11_196G111		Other	other	6,816	2,43×10^-02	
ENSG00000232748.3	RP11_196G116		Other	other	4,593	9,78×10^-03	
ENSG00000261441.1	RP11_217B12		Other	other	3,391	1,80×10^-05	
ENSG00000253288.1	RP11_238K61		Other	other	5,360	1,88×10^-02	
ENSG00000233724.1	RP11_252C242		Other	other	7,300	2,79×10^-02	
ENSG00000278319.1	RP11_266I38		Other	other	8,171	1,10×10^-03	
ENSG00000260310.1	RP11_27M242		Other	other	10,475	1,81×10^-02	
ENSG00000257991.1	RP11_2H83		Other	other	2,285	1,79×10^-04	
ENSG00000273063.1	RP11_334G221		Other	other	5,691	4,80×10^-02	
ENSG00000270696.1	RP11_342K61		Other	other	4,015	4,57×10^-02	

ENSG00000249359.2	RP11_374A41		Other	other	8,745	1,29×10^-06	
ENSG00000254485.5	RP11_380O241		Other	other	4,177	2,09×10^-02	
ENSG00000248755.1	RP11_384D101		Other	other	10,765	1,65×10^-63	
ENSG00000263717.1	RP11_466A196		Other	other	6,912	1,22×10^-02	
ENSG00000278060.1	RP11_466M211		Other	other	12,908	1,92×10^-07	
ENSG00000261889.1	RP11_473M2016		Other	other	3,291	5,52×10^-16	
ENSG00000276131.1	RP11_481J23		Other	other	6,107	7,31×10^-06	
ENSG00000259677.1	RP11_493E31		Other	other	13,795	9,18×10^-17	
ENSG00000237212.1	RP11_569G132		Other	other	5,505	3,91×10^-02	
ENSG00000258413.1	RP11_665C166		Other	other	4,406	4,10×10^-02	
ENSG00000279086.1	RP11_667F141		Other	other	4,154	7,45×10^-06	
ENSG00000254044.5	RP11_681L81		Other	other	9,126	3,91×10^-03	
ENSG00000251588.2	RP11_698E181		Other	other	11,661	6,80×10^-76	
ENSG00000237074.1	RP11_6J212		Other	other	9,874	3,98×10^-02	
ENSG00000255028.5	RP11_708B62		Other	other	11,466	4,03×10^-17	
ENSG00000256469.1	RP11_856F162		Other	other	5,407	2,48×10^-02	
ENSG00000253872.1	RP11_90D111		Other	other	13,578	1,82×10^-18	
ENSG00000281039.1	RP4-777O23.3		Other	other	9,684	9,51×10^-08	
ENSG00000236674.1	RP4_792G43		Other	other	8,723	1,35×10^-30	
ENSG00000236772.1	RP5_1184F45		Other	other	6,554	1,01×10^-05	
ENSG00000231010.1	RP6_109B72		Other	other	6,645	1,07×10^-03	
ENSG00000108298.9	RPL19	ribosomal protein L19	Cytoplasm	other	-1,102	4,86×10^-05	
ENSG00000114391.12	RPL24	ribosomal protein L24	Cytoplasm	other	-1,732	3,61×10^-02	
ENSG00000242595.1	RPL26P26	ribosomal protein L26 pseudogene 26	Other	other	10,149	9,69×10^-11	
ENSG00000162244.10	RPL29	ribosomal protein L29	Cytoplasm	other	-1,774	2,79×10^-02	
ENSG00000136942.14	RPL35	ribosomal protein L35	Cytoplasm	other	-1,730	1,43×10^-05	
ENSG00000122406.12	RPL5	ribosomal protein L5	Cytoplasm	other	-1,895	1,34×10^-03	
ENSG00000228462.1	RPS19P7	ribosomal protein S19 pseudogene 7	Other	other	10,582	2,51×10^-02	
ENSG00000186468.12	RPS23	ribosomal protein S23	Cytoplasm	translation regulator	-1,288	4,36×10^-02	
ENSG00000125844.15	RRBP1	ribosome binding protein 1	Cytoplasm	other	-1,094	3,34×10^-02	
ENSG00000132275.10	RRP8	ribosomal RNA processing 8	Nucleus	enzyme	2,472	2,06×10^-03	
ENSG00000103449.11	SALL1	spalt like transcription factor 1	Nucleus	transcription regulator	-1,967	2,51×10^-02	
ENSG00000155875.13	SAXO1	stabilizer of axonemal microtubules 1	Cytoplasm	other	5,300	1,34×10^-03	
ENSG00000252577.1	SCARNA20	small Cajal body-specific RNA 20	Other	other	2,511	9,01×10^-03	
ENSG00000280466.1	SCARNA4	small Cajal body-specific RNA 4	Other	other	11,300	2,59×10^-16	
ENSG00000281394.1	SCARNA4	small Cajal body-specific RNA 4	Other	other	-3,868	2,70×10^-02	
ENSG00000251733.1	SCARNA8	small Cajal body-specific RNA 8	Other	other	2,328	5,11×10^-04	
ENSG00000169432.14	SCN9A	sodium voltage-gated channel alpha subunit 9	Plasma Membrane	ion channel	3,117	4,16×10^-02	bupivacaine/lidocai
ENSG00000146555.18	SDK1	sidekick cell adhesion molecule 1	Plasma Membrane	other	4,499	2,32×10^-02	
ENSG00000139410.14	SDSL	serine dehydratase like	Cytoplasm	enzyme	3,676	2,51×10^-03	
ENSG00000148396.18	SEC16A	SEC16 homolog A, endoplasmic reticulum export factor	Cytoplasm	other	1,251	4,23×10^-02	
ENSG00000150961.14	SEC24D	SEC24 homolog D, COPII coat complex component	Cytoplasm	transporter	1,791	1,24×10^-02	
ENSG00000075826.16	SEC31B	SEC31 homolog B, COPII coat complex component	Cytoplasm	other	2,594	5,00×10^-03	
ENSG00000007908.15	SELE	selectin E	Plasma Membrane	transmembrane receptor	8,069	2,46×10^-48	uproleselan, GMI-1
ENSG00000248445.5	SEMA6A-AS1	SEMA6A antisense RNA 1	Other	other	5,810	2,66×10^-07	
ENSG00000106366.8	SERPINE1	serpin family E member 1	Extracellular Space	other	2,212	9,31×10^-03	TM5614, drotrecog
ENSG00000099995.18	SF3A1	splicing factor 3a subunit 1	Nucleus	other	-3,823	2,71×10^-02	
ENSG00000087365.14	SF3B2	splicing factor 3b subunit 2	Nucleus	other	-1,152	1,30×10^-02	
ENSG00000104611.11	SH2D4A	SH2 domain containing 4A	Cytoplasm	other	2,434	1,34×10^-02	
ENSG00000251322.7	SHANK3	SH3 and multiple ankyrin repeat domains 3	Plasma Membrane	other	1,889	3,61×10^-03	
ENSG00000164403.14	SHROOM1	shroom family member 1	Other	other	3,955	4,49×10^-03	
ENSG00000127511.9	SIN3B	SIN3 transcription regulator family member B	Nucleus	transcription regulator	-3,790	3,49×10^-02	
ENSG00000163406.10	SLC15A2	solute carrier family 15 member 2	Plasma Membrane	transporter	4,837	2,91×10^-05	
ENSG00000261608.1	SLC25A1P4	solute carrier family 25 member 1 pseudogene 4	Other	other	7,336	8,10×10^-10	
ENSG00000258708.1	SLC25A21-AS1	SLC25A21 antisense RNA 1	Other	other	4,385	2,77×10^-02	
ENSG00000160785.13	SLC25A44	solute carrier family 25 member 44	Cytoplasm	transporter	2,806	1,91×10^-14	
ENSG00000215790.6	SLC35E2A	solute carrier family 35 member E2A	Other	other	4,291	2,60×10^-19	
ENSG00000123643.12	SLC36A1	solute carrier family 36 member 1	Plasma Membrane	transporter	3,139	2,70×10^-02	
ENSG00000138079.13	SLC3A1	solute carrier family 3 member 1	Plasma Membrane	transporter	2,810	4,53×10^-16	
ENSG00000188687.15	SLC4A5	solute carrier family 4 member 5	Plasma Membrane	transporter	6,636	1,64×10^-11	
ENSG00000050438.16	SLC4A8	solute carrier family 4 member 8	Plasma Membrane	transporter	3,091	7,13×10^-11	
ENSG00000164363.9	SLC6A18	solute carrier family 6 member 18	Plasma Membrane	transporter	2,921	1,86×10^-03	
ENSG00000100678.18	SLC8A3	solute carrier family 8 member A3	Plasma Membrane	transporter	3,407	1,03×10^-09	
ENSG00000214329.7	SLC9B1P2	solute carrier family 9 member B1 pseudogene 2	Other	other	10,822	2,65×10^-02	
ENSG00000137776.16	SLTM	SAFB like transcription modulator	Nucleus	other	-1,809	1,34×10^-02	
ENSG00000181625.17	SLX1A/SLX1B	SLX1 homolog B, structure-specific endonuclease subunit	Nucleus	enzyme	5,036	9,73×10^-03	
ENSG00000153147.5	SMARCA5	SWI/SNF related, matrix associated, actin dependent regulator of chro	Nucleus	transcription regulator	-2,100	2,79×10^-02	
ENSG00000173473.10	SMARCC1	SWI/SNF related, matrix associated, actin dependent regulator of chro	Nucleus	transcription regulator	-1,197	7,16×10^-03	
ENSG00000176994.10	SMCR8	SMCR8-C9orf72 complex subunit	Nucleus	other	1,797	4,70×10^-03	
ENSG00000205670.10	SMIM11A	small integral membrane protein 11A	Plasma Membrane	other	7,300	2,79×10^-02	
ENSG00000281398.2	SNHG4	small nucleolar RNA host gene 4	Other	other	6,193	3,80×10^-05	
ENSG00000184602.5	SNN	stannin	Plasma Membrane	other	1,943	3,49×10^-02	
ENSG00000206811.1	SNORA10	small nucleolar RNA, H/ACA box 10	Other	other	1,506	3,14×10^-02	
ENSG00000221716.1	SNORA11	small nucleolar RNA, H/ACA box 11	Other	other	1,997	1,18×10^-03	
ENSG00000221705.1	SNORA11E	small nucleolar RNA, H/ACA box 11E	Other	other	10,203	3,98×10^-02	
ENSG00000280498.1	SNORA16A	small nucleolar RNA, H/ACA box 16A	Other	other	9,814	3,30×10^-02	
ENSG00000276161.1	SNORA17B	small nucleolar RNA, H/ACA box 17B	Other	other	9,126	3,91×10^-03	
ENSG00000206903.1	SNORA24B	small nucleolar RNA, H/ACA box 24B	Other	other	4,595	1,32×10^-04	
ENSG00000206612.1	SNORA2A	small nucleolar RNA, H/ACA box 2A	Other	other	3,135	5,00×10^-03	
ENSG00000207313.1	SNORA2B	small nucleolar RNA, H/ACA box 2B	Other	other	2,889	9,04×10^-03	
ENSG00000221491.2	SNORA2C	small nucleolar RNA, H/ACA box 2C	Other	other	2,467	4,78×10^-03	
ENSG00000253051.1	SNORA31B	small nucleolar RNA, H/ACA box 31B	Other	other	1,314	2,74×10^-03	
ENSG00000263776.1	SNORA4	small nucleolar RNA, H/ACA box 4	Other	other	1,872	5,72×10^-05	
ENSG00000206838.1	SNORA5A	small nucleolar RNA, H/ACA box 5A	Other	other	1,956	1,11×10^-02	
ENSG00000207405.1	SNORA64	small nucleolar RNA, H/ACA box 64	Other	other	2,201	2,89×10^-03	
ENSG00000207523.1	SNORA66	small nucleolar RNA, H/ACA box 66	Other	other	1,307	4,30×10^-04	
ENSG00000200087.1	SNORA73B	small nucleolar RNA, H/ACA box 73B	Other	other	1,382	1,96×10^-06	
ENSG00000206885.1	SNORA75	small nucleolar RNA, H/ACA box 75	Other	other	1,225	2,79×10^-02	
ENSG00000222489.1	SNORA79B	small nucleolar RNA, H/ACA box 79B	Other	other	1,951	2,21×10^-05	
ENSG00000200792.1	SNORA80A	small nucleolar RNA, H/ACA box 80A	Other	other	4,021	8,24×10^-05	
ENSG00000277194.1	SNORD22	small nucleolar RNA, C/D box 22	Other	other	1,242	7,75×10^-04	
ENSG00000263934.4	SNORD3A	small nucleolar RNA, C/D box 3A	Other	other	1,032	2,32×10^-02	
ENSG00000206602.1	SNORD58A	small nucleolar RNA, C/D box 58A	Other	other	3,180	2,57×10^-04	
ENSG00000223224.1	SNORD71	small nucleolar RNA, C/D box 71	Other	other	4,251	5,20×10^-04	
ENSG00000208772.1	SNORD94	small nucleolar RNA, C/D box 94	Other	other	2,818	5,85×10^-03	
ENSG00000101400.5	SNTA1	syntrophin alpha 1	Plasma Membrane	other	3,645	1,03×10^-02	
ENSG00000168807.16	SNTB2	syntrophin beta 2	Plasma Membrane	other	3,893	2,85×10^-27	
ENSG00000172803.17	SNX32	sorting nexin 32	Other	other	4,554	4,06×10^-04	
ENSG00000113140.10	SPARC	secreted protein acidic and cysteine rich	Extracellular Space	other	1,368	1,85×10^-04	
ENSG00000158792.15	SPATA2L	spermatogenesis associated 2 like	Other	other	3,255	4,20×10^-02	
ENSG00000066336.11	SPI1	Spi-1 proto-oncogene	Nucleus	transcription regulator	2,622	1,12×10^-02	
ENSG00000107742.12	SPOCK2	SPARC (osteonectin), cwcv and kazal like domains proteoglycan 2	Extracellular Space	other	3,382	8,61×10^-04	
ENSG00000262655.3	SPON1	spondin 1	Extracellular Space	other	2,058	9,50×10^-03	
ENSG00000118785.13	SPP1	secreted phosphoprotein 1	Extracellular Space	cytokine	1,920	1,33×10^-02	
ENSG00000161011.19	SQSTM1	sequestosome 1	Cytoplasm	transcription regulator	1,326	2,92×10^-03	
ENSG00000153914.15	SREK1	splicing regulatory glutamic acid and lysine rich protein 1	Nucleus	other	-2,132	4,97×10^-03	
ENSG00000116754.13	SRSF11	serine and arginine rich splicing factor 11	Nucleus	other	-1,198	4,53×10^-02	
ENSG00000111728.10	ST8SIA1	ST8 alpha-N-acetyl-neuraminide alpha-2,8-sialyltransferase 1	Cytoplasm	enzyme	2,641	2,84×10^-02	
ENSG00000214530.7	STARD10	StAR related lipid transfer domain containing 10	Cytoplasm	other	2,112	3,98×10^-02	
ENSG00000149043.16	SYT8	synaptotagmin 8	Cytoplasm	transporter	7,112	4,92×10^-07	
ENSG00000147526.19	TACC1	transforming acidic coiled-coil containing protein 1	Nucleus	other	2,148	1,72×10^-04	

ENSG00000176769.9	TCERG1L	transcription elongation regulator 1 like	Other	other	3,305	1,61×10^-03	
ENSG00000125878.5	TCF15	transcription factor 15	Nucleus	transcription regulator	7,322	2,03×10^-61	
ENSG00000182134.15	TDRKH	tudor and KH domain containing	Cytoplasm	other	3,298	3,45×10^-04	
ENSG00000009694.13	TENM1	teneurin transmembrane protein 1	Plasma Membrane	transmembrane receptor	4,060	6,69×10^-06	
ENSG00000112773.15	TENT5A	terminal nucleotidyltransferase 5A	Other	other	2,462	8,12×10^-04	
ENSG00000249311.4	TERF1P3	TERF1 pseudogene 3	Other	other	5,821	2,93×10^-02	
ENSG00000223824.1	TERF1P6	TERF1 pseudogene 6	Other	other	12,540	3,36×10^-09	
ENSG00000135269.17	TES	testin LIM domain protein	Plasma Membrane	other	2,142	4,06×10^-05	
ENSG00000121101.15	TEX14	testis expressed 14, intercellular bridge forming factor	Plasma Membrane	kinase	4,759	1,61×10^-04	
ENSG00000029639.10	TFB1M	transcription factor B1, mitochondrial	Cytoplasm	transcription regulator	2,400	2,38×10^-02	
ENSG00000115112.7	TFCP2L1	transcription factor CP2 like 1	Nucleus	transcription regulator	2,653	2,72×10^-02	
ENSG00000072274.12	TFRC	transferrin receptor	Plasma Membrane	transporter	2,087	8,77×10^-03	CALAA-01, CX-202
ENSG00000120708.16	TGFBI	transforming growth factor beta induced	Extracellular Space	other	2,517	3,59×10^-05	
ENSG00000137801.10	THBS1	thrombospondin 1	Extracellular Space	other	1,250	1,88×10^-02	
ENSG00000064115.10	TM7SF3	transmembrane 7 superfamily member 3	Plasma Membrane	other	3,168	4,22×10^-03	
ENSG00000244187.7	TMEM141	transmembrane protein 141	Other	other	1,638	4,33×10^-02	
ENSG00000164484.11	TMEM200A	transmembrane protein 200A	Other	other	3,224	4,73×10^-02	
ENSG00000072954.6	TMEM38A	transmembrane protein 38A	Cytoplasm	ion channel	8,401	4,30×10^-67	
ENSG00000095209.11	TMEM38B	transmembrane protein 38B	Nucleus	ion channel	3,470	7,70×10^-03	
ENSG00000175348.10	TMEM9B	TMEM9 domain family member B	Other	other	4,242	2,57×10^-04	
ENSG00000120802.13	TMPO	thymopoietin	Nucleus	other	3,163	6,06×10^-77	
ENSG00000168591.15	TMUB2	transmembrane and ubiquitin like domain containing 2	Other	other	2,851	1,40×10^-02	
ENSG00000118194.18	TNNT2	troponin T2, cardiac type	Cytoplasm	other	2,075	8,95×10^-15	
ENSG00000198900.5	TOP1	DNA topoisomerase I	Nucleus	enzyme	-1,777	9,60×10^-03	bevacizumab/irinot
ENSG00000131747.14	TOP2A	DNA topoisomerase II alpha	Nucleus	enzyme	-3,107	4,80×10^-02	bortezomib/doxoru
ENSG00000111669.14	TPI1	triosephosphate isomerase 1	Cytoplasm	enzyme	-1,412	2,21×10^-02	
ENSG00000140416.19	TPM1	tropomyosin 1	Cytoplasm	other	1,001	1,15×10^-04	
ENSG00000076604.14	TRAF4	TNF receptor associated factor 4	Cytoplasm	other	1,457	3,71×10^-02	
ENSG00000196459.13	TRAPPC2	trafficking protein particle complex subunit 2	Cytoplasm	other	3,194	1,73×10^-02	
ENSG00000173334.3	TRIB1	tribbles pseudokinase 1	Cytoplasm	kinase	1,931	1,56×10^-02	
ENSG00000149743.13	TRPT1	tRNA phosphotransferase 1	Other	enzyme	2,415	3,26×10^-02	
ENSG00000170892.10	TSEN34	tRNA splicing endonuclease subunit 34	Nucleus	enzyme	3,347	1,63×10^-15	
ENSG00000176728.7	TTTY14	testis-specific transcript, Y-linked 14	Other	other	4,300	2,48×10^-02	
ENSG00000166402.8	TUB	TUB bipartite transcription factor	Cytoplasm	transcription regulator	1,769	6,05×10^-03	
ENSG00000104804.7	TULP2	TUB like protein 2	Other	enzyme	7,084	1,78×10^-02	
ENSG00000149016.15	TUT1	terminal uridylyl transferase 1, U6 snRNA-specific	Nucleus	enzyme	2,336	3,08×10^-02	
ENSG00000198431.15	TXNRD1	thioredoxin reductase 1	Cytoplasm	enzyme	1,437	2,42×10^-02	arsenic trioxide/ida
ENSG00000278591.1	U2		Other	other	2,479	2,91×10^-05	
ENSG00000272173.1	U47924.31		Other	other	4,120	4,48×10^-03	
ENSG00000108106.13	UBE2S	ubiquitin conjugating enzyme E2 S	Nucleus	enzyme	-4,047	1,63×10^-02	
ENSG00000175564.12	UCP3	uncoupling protein 3	Cytoplasm	transporter	4,862	1,98×10^-11	
ENSG00000151461.19	UPF2	UPF2 regulator of nonsense mediated mRNA decay	Cytoplasm	other	-1,593	2,51×10^-02	
ENSG00000105176.17	URI1	URI1 prefoldin like chaperone	Nucleus	transcription regulator	1,694	5,57×10^-04	
ENSG00000135093.12	USP30	ubiquitin specific peptidase 30	Cytoplasm	peptidase	5,094	8,05×10^-17	
ENSG00000171724.2	VAT1L	vesicle amine transport 1 like	Other	enzyme	2,826	6,64×10^-14	
ENSG00000112715.20	VEGFA	vascular endothelial growth factor A	Extracellular Space	growth factor	12,974	4,65×10^-89	bevacizumab/cape
ENSG00000173511.9	VEGFB	vascular endothelial growth factor B	Extracellular Space	growth factor	7,691	1,01×10^-285	aflibercept, aflibercε
ENSG00000171475.13	WIPF2	WAS/WASL interacting protein family member 2	Cytoplasm	other	1,893	5,43×10^-03	
ENSG00000200291.1	Y_RNA		Other	other	10,206	1,95×10^-02	
ENSG00000083896.12	YTHDC1	YTH domain containing 1	Cytoplasm	other	-1,357	1,88×10^-02	
ENSG00000108953.16	YWHAE	tyrosine 3-monooxygenase/tryptophan 5-monooxygenase activation p	Cytoplasm	other	-1,326	3,23×10^-03	
ENSG00000180011.6	ZADH2	zinc binding alcohol dehydrogenase domain containing 2	Cytoplasm	enzyme	3,208	1,34×10^-02	
ENSG00000146839.18	ZAN	zonadhesin	Plasma Membrane	other	6,167	5,79×10^-04	
ENSG00000198081.10	ZBTB14	zinc finger and BTB domain containing 14	Nucleus	transcription regulator	2,486	2,36×10^-03	
ENSG00000123200.16	ZC3H13	zinc finger CCCH-type containing 13	Extracellular Space	other	-1,344	4,96×10^-04	
ENSG00000206077.10	ZDHHC11B	zinc finger DHHC-type containing 11B	Other	other	4,974	2,86×10^-02	
ENSG00000156599.10	ZDHHC5	zinc finger DHHC-type palmitoyltransferase 5	Nucleus	enzyme	4,168	9,97×10^-05	
ENSG00000169554.16	ZEB2	zinc finger E-box binding homeobox 2	Nucleus	transcription regulator	-3,808	2,98×10^-02	
ENSG00000229043.2	ZFAND2A-DT	ZFAND2A divergent transcript	Other	other	5,716	2,55×10^-19	
ENSG00000169946.13	ZFPM2	zinc finger protein, FOG family member 2	Nucleus	transcription regulator	2,462	4,44×10^-03	
ENSG00000188070.9	ZFTA	zinc finger translocation associated	Other	other	1,819	3,37×10^-03	
ENSG00000166526.16	ZNF3	zinc finger protein 3	Nucleus	transcription regulator	1,636	4,36×10^-02	
ENSG00000215452.3	ZNF663P	zinc finger protein 663, pseudogene	Other	other	4,507	2,65×10^-02	
ENSG00000197372.9	ZNF675	zinc finger protein 675	Nucleus	transcription regulator	2,307	2,61×10^-02	
ENSG00000215343.7	ZNF705B/ZNF705D	zinc finger protein 705B	Other	other	6,523	4,14×10^-05	
ENSG00000213967.10	ZNF726	zinc finger protein 726	Other	other	4,710	1,28×10^-04	
ENSG00000181220.15	ZNF746	zinc finger protein 746	Cytoplasm	transcription regulator	1,740	4,80×10^-02	
ENSG00000133624.13	ZNF767P	zinc finger family member 767, pseudogene	Other	other	1,957	2,19×10^-02	
ENSG00000198146.4	ZNF770	zinc finger protein 770	Other	other	1,982	1,17×10^-02	
ENSG00000183579.15	ZNRF3	zinc and ring finger 3	Plasma Membrane	enzyme	2,133	2,09×10^-02	
ENSG00000074755.14	ZZEF1	zinc finger ZZ-type and EF-hand domain containing 1	Other	other	1,360	2,47×10^-02	

Suppl. Table 4: Top 30 up-regulated genes in HTB-EVs, HUVEC-EVS and CPC-EVs after LNP treatment

HTB EVs

Ensembl ID	Symbol	Entrez Gene Name	Location	Type(s)	Drug(s)	logFC (with shrinkage)	FDR
ENSG00000112715.20	VEGFA	vascular endothelial growth factor A	Extracellular Space	growth factor	bevacizumab/capecitabine, bevacizumab	16,7638939	0
ENSG00000275757.1	RNA5-8SN1	RNA, 5.8S ribosomal N1	Other	other		10,78867104	2,56815E-09
ENSG00000176971.3	FIBIN	fin bud initiation factor homolog	Cytoplasm	other		7,574350765	6,55909E-44
ENSG00000278099.1	RNVU1-2A	RNA, variant U1 small nuclear 2A	Other	other		6,605796274	2,22663E-15
ENSG00000214967.5	NPIPA5 (includes others)	nuclear pore complex interacting protein family member A5	Nucleus	other		5,813595538	3,69606E-07
ENSG00000019991.15	HGF	hepatocyte growth factor	Extracellular Space	growth factor	rilotumumab, SCH 900105, bevacizumab	5,708103623	1,33943E-46
ENSG00000200885.1	RNU1-146P	RNA, U1 small nuclear 146, pseudogene	Other	other		5,6782403	6,49836E-07
ENSG00000199879.1	RNVU1-22		Other	other		5,550062988	1,53873E-13
ENSG00000065970.8	FOXJ2	forkhead box J2	Nucleus	transcription regulator		5,386801881	2,07908E-23
ENSG00000202444.1	RNU5E-6P	RNA, U5E small nuclear 6, pseudogene	Other	other		5,137487387	1,59998E-10
ENSG00000108551.4	RASD1	ras related dexamethasone induced 1	Cytoplasm	enzyme		4,975787293	1,0751E-20
ENSG00000207110.1	RNVU1-32	RNA, variant U1 small nuclear 32	Other	other		4,706741384	4,07645E-07
ENSG00000200340.1	RNU1-105P	RNA, U1 small nuclear 105, pseudogene	Other	other		4,544910827	2,17745E-07
ENSG00000207340.1	RNVU1-1	RNA, variant U1 small nuclear 1	Other	other		4,504878173	2,67928E-05
ENSG00000275538.1	RNVU1-19	RNA, variant U1 small nuclear 19	Other	other		4,43361906	7,75586E-05
ENSG00000273544.1	SNORA44	small nucleolar RNA, H/ACA box 44	Other	other		4,400864658	5,6172E-05
ENSG00000243635.1	RP11_281P111		Other	other		4,255287841	1,60104E-08
ENSG00000258186.2	SLC7A5P2	solute carrier family 7 member 5 pseudogene 2	Other	other		4,109981866	2,61499E-06
ENSG00000222627.1	RNU2-37P	RNA, U2 small nuclear 37, pseudogene	Other	other		4,106063439	3,76798E-06
ENSG00000130522.5	JUND	JunD proto-oncogene, AP-1 transcription factor subunit	Nucleus	transcription regulator		3,94643134	1,13445E-19
ENSG00000212170.1	RNU1-77P		Other	other		3,89262613	6,06624E-09
ENSG00000200087.1	SNORA73B	small nucleolar RNA, H/ACA box 73B	Other	other		3,845409878	9,11862E-06
ENSG00000206908.1	RNU1-136P	RNA, U1 small nuclear 136, pseudogene	Other	other		3,811585309	2,50757E-05
ENSG00000278274.1	SNORA61	small nucleolar RNA, H/ACA box 61	Other	other		3,778741906	1,23235E-05
ENSG00000215606.4	KRT18P35	keratin 18 pseudogene 35	Other	other		3,771943277	4,9453E-05
ENSG00000222810.1	RNU2-68P		Other	other		3,728128631	7,1474E-05
ENSG00000171223.5	JUNB	JunB proto-oncogene, AP-1 transcription factor subunit	Nucleus	transcription regulator		3,690049262	1,08638E-16
ENSG00000202538.1	RNU4-2	RNA, U4 small nuclear 2	Other	other		3,677448138	2,82711E-13
ENSG00000259135.1	RP11_671J114		Other	other		3,670057508	2,36245E-05
ENSG00000260727.1	SLC7A5P1	solute carrier family 7 member 5 pseudogene 1	Other	other		3,588285017	0,000233762

HUVEC EVs

Ensembl ID	Symbol	Entrez Gene Name	Location	Type(s)	Drug(s)	logFC (with shrinkage)	FDR
ENSG00000112715.20	VEGFA	vascular endothelial growth factor A	Extracellular Space	growth factor	bevacizumab/capecitabine, bevacizumab	20,4252527	0
ENSG00000201185.1	RNA5SP202	RNA, 5S ribosomal pseudogene 202	Other	other		10,02558347	8,62529E-18
ENSG00000225840.2	AC010970.2		Other	other		9,07672313	6,0089E-127
ENSG00000200434.1	RNA5-8SP2	RNA, 5.8S ribosomal pseudogene 2	Other	other		8,083228537	3,97516E-28
ENSG00000257991.1	RP11_2H83		Other	other		7,858671997	1,14121E-10
ENSG00000019991.15	HGF	hepatocyte growth factor	Extracellular Space	growth factor	rilotumumab, SCH 900105, bevacizumab	7,63716289	5,684E-145
ENSG00000281181.1	CH507-513H4.3		Other	other		7,509460828	1,6613E-13
ENSG00000252680.1	RNA5SP449	RNA, 5S ribosomal pseudogene 449	Other	other		7,397762354	6,86191E-12
ENSG00000015413.9	DPEP1	dipeptidase 1	Cytoplasm	peptidase	cilastatin	7,297052669	4,66146E-76
ENSG00000199347.1	RNU5E-1	RNA, U5E small nuclear 1	Other	other		7,253111467	2,87713E-26
ENSG00000202386.1	RNA5SP211	RNA, 5S ribosomal pseudogene 211	Other	other		7,161312833	6,39555E-12
ENSG00000281383.1	CH507-513H4.5		Other	other		6,946164915	6,05524E-11
ENSG00000065970.8	FOXJ2	forkhead box J2	Nucleus	transcription regulator		6,840590502	4,1982E-133
ENSG00000199990.1	VTRNA1-1	vault RNA 1-1	Other	other		6,781631446	1,30265E-09
ENSG00000280800.1	CH507-513H4.6		Other	other		6,724771253	3,64827E-15
ENSG00000260182.1	RP11_616M225		Other	other		6,526017785	9,05423E-09
ENSG00000274266.1	SNORA73A	small nucleolar RNA, H/ACA box 73A	Nucleus	other		6,515688769	2,00685E-06
ENSG00000280614.1	CH507-513H4.4		Other	other		6,508337794	1,05665E-14
ENSG00000252261.1	RNA5SP262	RNA, 5S ribosomal pseudogene 262	Other	other		6,218061432	4,83831E-08
ENSG00000251770.1	RNA5SP486	RNA, 5S ribosomal pseudogene 486	Other	other		6,152236673	1,57079E-07
ENSG00000278233.1	RNA5-8SN2	RNA, 5.8S ribosomal N2	Other	other		6,11872747	1,21867E-07
ENSG00000275215.1	RNA5-8SN3	RNA, 5.8S ribosomal N3	Other	other		6,072374018	1,73401E-07
ENSG00000243635.1	RP11_281P111		Other	other		6,057465121	8,1077E-08
ENSG00000276700.1	RNA5-8SN4	RNA, 5.8S ribosomal N4	Other	other		5,923918287	7,04946E-07
ENSG00000202199.1	RNU1-115P		Other	other		5,813625065	4,04873E-26
ENSG00000183305.13	MAGEA2/MAGEA2B	MAGE family member A2	Nucleus	other		5,806013468	6,53823E-16
ENSG00000274917.1	RNA5-8SN5	RNA, 5.8S ribosomal N5	Other	other		5,731989554	2,00685E-06
ENSG00000176971.3	FIBIN	fin bud initiation factor homolog	Cytoplasm	other		5,719205634	2,80637E-77
ENSG00000103495.13	MAZ	MYC associated zinc finger protein	Nucleus	transcription regulator		5,707965086	3,50166E-14
ENSG00000199879.1	RNVU1-22		Other	other		5,707158449	1,9962E-32

CPC EVs

Ensembl ID	Symbol	Entrez Gene Name	Location	Type(s)	Drug(s)	logFC (with shrinkage)	FDR
ENSG00000256569.1	RP11_173P155		Other	other		24,61299343	4,33184E-10
ENSG00000224141.5	MIR548XHG	MIR548X host gene	Other	other		13,45233539	9,70875E-40
ENSG00000112715.20	VEGFA	vascular endothelial growth factor A	Extracellular Space	growth factor	bevacizumab/capecitabine, bevacizumab	12,74905693	4,64543E-89
ENSG00000259677.1	RP11_493E31		Other	other		12,4933458	9,18369E-17
ENSG00000253872.1	RP11_90D111		Other	other		12,43580251	1,81975E-18
ENSG00000257639.1	CTB_31N192		Other	other		12,24095457	2,05451E-24
ENSG00000143552.9	NUP210L	nucleoporin 210 like	Other	other		12,12876803	5,05192E-69
ENSG00000065970.8	FOXJ2	forkhead box J2	Nucleus	transcription regulator		12,09474013	4,23844E-28
ENSG00000162618.12	ADGRL4	adhesion G protein-coupled receptor L4	Plasma Membrane	G-protein coupled receptor		12,06292079	3,43936E-31
ENSG00000251588.2	RP11_698E181		Other	other		11,46615334	6,79589E-76
ENSG00000206530.8	CFAP44	cilia and flagella associated protein 44	Extracellular Space	peptidase		11,20110344	7,4869E-101
ENSG00000243478.7	AOX2P	aldehyde oxidase 2, pseudogene	Other	other		11,18089081	4,67361E-61
ENSG00000206172.8	HBA1/HBA2	hemoglobin subunit alpha 2	Extracellular Space	transporter	iron dextran, mefloquine, donepezil/met	11,16361685	5,20662E-16
ENSG00000266149.1	LOC100192426	uncharacterized LOC100192426	Other	other		11,07060732	7,14722E-19
ENSG00000188536.12	HBA1/HBA2	hemoglobin subunit alpha 2	Extracellular Space	transporter	iron dextran, mefloquine, donepezil/met	10,87043609	8,9646E-136
ENSG00000237556.1	KCND3-AS1	KCND3 antisense RNA 1	Other	other		10,82229497	1,03262E-17
ENSG00000223824.1	TERF1P6	TERF1 pseudogene 6	Other	other		10,80514479	3,35922E-09
ENSG00000255028.5	RP11_708B62		Other	other		10,69031556	4,03476E-17
ENSG00000278060.1	RP11_466M211		Other	other		10,65131221	1,92498E-07
ENSG00000248755.1	RP11_384D101		Other	other		10,58056021	1,65251E-63
ENSG00000149635.2	OCSTAMP	osteoclast stimulatory transmembrane protein	Other	other		10,53633151	8,3787E-106
ENSG00000280466.1	SCARNA4	small Cajal body-specific RNA 4	Other	other		10,52021176	2,59046E-16
ENSG00000176605.7	C14orf177	chromosome 14 putative open reading frame 177	Other	other		10,45584524	3,67805E-17
ENSG00000273415.1	LINC02725		Other	other		10,44913711	1,6619E-09
ENSG00000113302.4	IL12B	interleukin 12B	Extracellular Space	cytokine	ustekinumab, methotrexate/ustekinuma	9,937279869	3,85469E-45
ENSG00000249439.1	HMGN1P14	high mobility group nucleosome binding domain 1 pseudogene 14	Other	other		9,63539843	2,53562E-12
ENSG00000242595.1	RPL26P26	ribosomal protein L26 pseudogene 26	Other	other		9,270156146	9,69255E-11
ENSG00000273768.1	RNVU1-29		Other	other		9,157891591	8,2247E-63
ENSG00000158315.10	RHBDL2	rhomboid like 2	Plasma Membrane	peptidase		9,036847015	2,4724E-112
ENSG00000236231.1	AC0170482		Other	other		9,015197557	2,29584E-27

Suppl. Table 5: Top 30 Down-regulated genes in HTB-EVs, HUVEC-EVS and CPC-EVs after LNP treatment

HTB EVs

Ensembl ID	Symbol	Entrez Gene Name	Location	Type(s)	Drug(s)	logFC (with shrinkage)	FDR
ENSG00000196611.4	MMP1	matrix metallopeptidase 1	Extracellular Space	peptidase	rebimastat, marimastat	-8,308536508	5,04956E-13
ENSG00000137801.10	THBS1	thrombospondin 1	Extracellular Space	other		-5,64032192	2,60802E-20
ENSG00000142798.16	HSPG2	heparan sulfate proteoglycan 2	Extracellular Space	enzyme		-4,832042736	6,02393E-17
ENSG00000249751.3	ECSCR	endothelial cell surface expressed chemotaxis and apoptosis regulator	Other	other		-4,641547271	3,12819E-06
ENSG00000110799.13	VWF	von Willebrand factor	Extracellular Space	other	F8, caplacizumab, turoctocog	-4,609243901	2,82711E-13
ENSG00000150093.18	ITGB1	integrin subunit beta 1	Plasma Membrane	transmembrane receptor	OS2966	-4,089546376	1,64653E-12
ENSG00000125148.6	MT2A	metallothionein 2A	Cytoplasm	other		-4,043096524	1,88313E-12
ENSG00000138722.9	MMRN1	multimerin 1	Extracellular Space	other		-3,717085048	1,19527E-11
ENSG00000115380.18	EFEMP1	EGF containing fibulin extracellular matrix protein 1	Extracellular Space	enzyme		-3,629313884	1,21559E-12
ENSG00000196787.3	H2AC11	H2A clustered histone 11	Nucleus	other		-3,467559656	3,17788E-06
ENSG00000135636.13	DYSF	dysferlin	Plasma Membrane	other		-3,425418625	0,000249541
ENSG00000172889.15	EGFL7	EGF like domain multiple 7	Extracellular Space	other	parsatuzumab	-3,394326141	0,000304111
ENSG00000143870.12	PDIA6	protein disulfide isomerase family A member 6	Cytoplasm	enzyme		-3,364962353	8,35571E-10
ENSG00000104823.8	ECH1	enoyl-CoA hydratase 1	Cytoplasm	enzyme		-3,331832506	0,000377433
ENSG00000111348.8	ARHGDIB	Rho GDP dissociation inhibitor beta	Cytoplasm	enzyme		-3,285671303	1,26147E-05
ENSG00000134871.17	COL4A2	collagen type IV alpha 2 chain	Extracellular Space	other	collagenase	-3,23495724	3,31729E-09
ENSG00000142192.20	APP	amyloid beta precursor protein	Plasma Membrane	other	bapineuzumab, florbetapir F1	-3,224611745	1,21314E-10
ENSG00000106991.13	ENG	endoglin	Plasma Membrane	transmembrane receptor	carotuximab	-3,181751834	7,69146E-06
ENSG00000179776.17	CDH5	cadherin 5	Plasma Membrane	other		-3,147392705	1,68551E-09
ENSG00000100129.17	EIF3L	eukaryotic translation initiation factor 3 subunit L	Cytoplasm	translation regulator		-3,054548959	0,000227985
ENSG00000113758.13	DBN1	drebrin 1	Cytoplasm	other		-3,051880106	3,3819E-11
ENSG00000261371.5	PECAM1	platelet and endothelial cell adhesion molecule 1	Plasma Membrane	other		-3,046442235	1,68551E-09
ENSG00000113140.10	SPARC	secreted protein acidic and cysteine rich	Extracellular Space	other		-2,981638238	5,78791E-10
ENSG00000131236.16	CAP1	cyclase associated actin cytoskeleton regulatory protein 1	Plasma Membrane	other		-2,977234815	1,10641E-05
ENSG00000129250.11	KIF1C	kinesin family member 1C	Cytoplasm	other		-2,943325474	9,32905E-08
ENSG00000135404.11	CD63	CD63 molecule	Plasma Membrane	other		-2,899827241	0,000131651
ENSG00000166825.13	ANPEP	alanyl aminopeptidase, membrane	Plasma Membrane	peptidase	CNGRC peptide-TNF alpha cor	-2,744646402	3,76798E-06
ENSG00000187498.14	COL4A1	collagen type IV alpha 1 chain	Extracellular Space	other	collagenase	-2,735496475	2,51672E-07
ENSG00000163513.17	TGFBR2	transforming growth factor beta receptor 2	Plasma Membrane	kinase	IMC-TR1, SM1-71	-2,72383406	1,51385E-07
ENSG00000128591.15	FLNC	filamin C	Cytoplasm	other		-2,711220139	0,000557576

HUVEC EVs

Ensembl ID	Symbol	Entrez Gene Name	Location	Type(s)	Drug(s)	logFC (with shrinkage)	FDR
ENSG00000276998.1	REXO1L2P	REXO1 like 2, pseudogene	Other	other		-6,142758041	1,18005E-09
ENSG00000225560.6	FAM197Y3 (includes others)	family with sequence similarity 197 Y-linked member 9	Other	other		-4,398635022	7,23261E-05
ENSG00000276077.4	LOC389831 (includes others)	uncharacterized LOC389831	Other	other		-4,089728061	0,000169772
ENSG00000280179.1	CH507_42P116		Other	other		-3,616025395	0,000546418
ENSG00000183791.4	ELOA3P (includes others)	elongin A3, pseudogene	Nucleus	other		-3,253075988	1,74896E-07
ENSG00000153976.2	HS3ST3A1	heparan sulfate-glucosamine 3-sulfotransferase 3A1	Cytoplasm	enzyme		-3,169302722	0,00131364
ENSG00000217874.2	OR4F7P	olfactory receptor family 4 subfamily F member 7 pseudogene	Other	other		-2,999755378	0,001563709
ENSG00000225154.2	TUBAP9	tubulin alpha pseudogene 9	Other	other		-2,971560697	0,001610584
ENSG00000233974.3	RP11_823P93		Other	other		-2,824459221	7,51093E-10
ENSG00000239732.3	TLR9	toll like receptor 9	Plasma Membrane	transmembrane receptor	cavrotolimod, MGN1703, ISS-	-2,793024321	0,002234268
ENSG00000233392.5	UICLM	up-regulated in colorectal cancer liver metastasis	Other	other		-2,578405324	0,00038082
ENSG00000262497.1	FAM187B2P	family with sequence similarity 187, member B pseudogene	Other	other		-2,460298772	0,003559976
ENSG00000274225.1	KB_68A71		Other	other		-2,383321275	0,004353547
ENSG00000160161.9	CILP2	cartilage intermediate layer protein 2	Extracellular Space	other		-2,376284023	0,004573157
ENSG00000271623.1	RP11_435I105		Other	other		-2,36470426	0,004066822
ENSG00000196796.5	NPIPB10P	nuclear pore complex interacting protein family, member B10, pseudogene	Other	other		-2,26579266	0,005190157
ENSG00000189357.8	SPATA31D1 (includes others)	SPATA31 subfamily D member 1	Other	other		-2,152363022	0,002989367
ENSG00000198754.5	OXCT2	3-oxoacid CoA-transferase 2	Cytoplasm	enzyme		-2,140375962	0,006571911
ENSG00000060491.16	OGFR	opioid growth factor receptor	Plasma Membrane	other	enkephalin, methionine	-2,066875031	0,000496139
ENSG00000281348.1	CTD-2574D22.6		Other	other		-1,963929158	0,00873464
ENSG00000071655.17	MBD3	methyl-CpG binding domain protein 3	Nucleus	other		-1,919399031	4,87445E-09
ENSG00000273046.1	HOXC5	homeobox C5	Nucleus	transcription regulator		-1,86105017	0,003659532
ENSG00000167633.16	KIR3DL1	killer cell immunoglobulin like receptor, three Ig domains and long cytoplasmic tail 1	Plasma Membrane	transmembrane receptor		-1,812905698	0,002621101
ENSG00000105429.12	MEGF8	multiple EGF like domains 8	Extracellular Space	other		-1,789057918	6,50162E-05
ENSG00000130383.7	FUT5	fucosyltransferase 5	Cytoplasm	enzyme		-1,771640921	0,00984336
ENSG00000275249.1	AC171558.3		Other	other		-1,74721071	0,011706827
ENSG00000221890.2	NPTXR	neuronal pentraxin receptor	Plasma Membrane	transmembrane receptor		-1,724943995	0,000366895
ENSG00000236081.1	ELFN1-AS1	ELFN1 antisense RNA 1	Other	other		-1,716011176	0,001253463
ENSG00000182484.14	WASH6P	WASP family homolog 6, pseudogene	Cytoplasm	other		-1,675738705	7,85952E-05
ENSG00000188086.12	PRSS45P	serine protease 45, pseudogene	Other	peptidase		-1,654302132	0,009351969

CPC EVs

Ensembl ID	Symbol	Entrez Gene Name	Location	Type(s)	Drug(s)	logFC (with shrinkage)	FDR
ENSG00000207513.1	RNU1-3	RNA, U1 small nuclear 3	Other	other		-9,000853731	1,80044E-10
ENSG00000206652.1	RNU1-1	RNA, U1 small nuclear 1	Other	other		-8,962137757	2,89312E-11
ENSG00000206588.1	RNU1-28P	RNA, U1 small nuclear 28, pseudogene	Other	other		-7,39621453	1,69076E-08
ENSG00000206737.1	RNVU1-18	RNA, variant U1 small nuclear 18	Other	other		-7,12519946	3,52752E-06
ENSG00000272196.2	H2AC18/H2AC19	H2A clustered histone 18	Nucleus	other		-4,330422253	0,000278141
ENSG00000138650.8	PCDH10	protocadherin 10	Plasma Membrane	other		-3,379935774	0,001975867
ENSG00000118482.11	PHF3	PHD finger protein 3	Nucleus	other		-2,994263377	0,00374634
ENSG00000205609.12	EIF3CL	eukaryotic translation initiation factor 3 subunit C like	Other	other		-2,433068411	0,010854623
ENSG00000171603.16	CLSTN1	calsyntenin 1	Plasma Membrane	other		-2,260991266	0,012813572
ENSG00000203852.3	H3C15	H3 clustered histone 15	Nucleus	other		-2,228967832	0,01349518
ENSG00000196411.9	EPHB4	EPH receptor B4	Plasma Membrane	kinase	tesevatinib, NVP-BHG712, JI 1	-2,172891067	0,01443703
ENSG00000108106.13	UBE2S	ubiquitin conjugating enzyme E2 S	Nucleus	enzyme		-2,1009886	0,016323224
ENSG00000140807.5	NKD1	NKD inhibitor of WNT signaling pathway 1	Other	other		-2,018593929	0,018763642
ENSG00000205592.13	MUC19	mucin 19, oligomeric	Cytoplasm	other		-1,913734366	0,023847242
ENSG00000049089.13	COL9A2	collagen type IX alpha 2 chain	Extracellular Space	other	collagenase	-1,897639103	0,009786585
ENSG00000008128.22	CDK11A	cyclin dependent kinase 11A	Nucleus	kinase		-1,893851922	0,023797458
ENSG00000281394.1	SCARNA4	small Cajal body-specific RNA 4	Other	other		-1,81893001	0,026984297
ENSG00000099995.18	SF3A1	splicing factor 3a subunit 1	Nucleus	other		-1,804753869	0,027071898
ENSG00000131196.17	NFATC1	nuclear factor of activated T cells 1	Nucleus	transcription regulator		-1,786433017	0,028088861
ENSG00000169554.16	ZEB2	zinc finger E-box binding homeobox 2	Nucleus	transcription regulator		-1,751477028	0,02984471
ENSG00000063180.8	CA11	carbonic anhydrase 11	Extracellular Space	enzyme		-1,751230643	0,030049914
ENSG00000142627.12	EPHA2	EPH receptor A2	Plasma Membrane	kinase	afatinib/dasatinib, dasatinib,	-1,716582069	0,03138832
ENSG00000127511.9	SIN3B	SIN3 transcription regulator family member B	Nucleus	transcription regulator		-1,678247406	0,034901617
ENSG00000150893.10	FREM2	FRAS1 related extracellular matrix 2	Extracellular Space	other		-1,625443466	0,038257778
ENSG00000277053.4	GTF2IP1	general transcription factor IIi pseudogene 1	Other	other		-1,619931945	0,02984471
ENSG00000160200.17	CBS/CBSL	cystathionine beta-synthase	Cytoplasm	enzyme		-1,540744143	0,024045871
ENSG00000153914.15	SREK1	splicing regulatory glutamic acid and lysine rich protein 1	Nucleus	other		-1,515823575	0,004971201
ENSG00000117523.15	PRRC2C	proline rich coiled-coil 2C	Cytoplasm	other		-1,515570455	5,93683E-08
ENSG00000155366.16	RHOC	ras homolog family member C	Plasma Membrane	enzyme		-1,511414953	0,047515949
ENSG00000146067.15	FAM193B	family with sequence similarity 193 member B	Nucleus	other		-1,506872338	0,047571695

Suppl. Table 6: Overlapped deregulated genes in HTB-EVs, HUVEC-EVS and CPC-EVs after LNP treatment

						Red/green visualisation		
**Ensembl ID**	**Symbol**	**HTB logFC**	**HUVEC logFC**	**CPC logFC**		**HTB logFC**	**HUVEC logFC**	**CPC logFC**
ENSG00000065970.8	FOXJ2	5,640392697	6,891193173	12,74790982		▲5,64	▲6,89	▲12,75
ENSG00000084754.10	HADHA	-1,84361252	2,839936215	2,159776463		▼-1,84	▲2,84	▲2,16
ENSG00000085224.20	ATRX	-1,496320877	1,525948531	-1,474349028		▼-1,5	▲1,53	▼-1,47
ENSG00000108298.9	RPL19	-1,886198387	2,070892798	-1,102364801		▼-1,89	▲2,07	▼-1,1
ENSG00000108654.11	DDX5	-1,440046676	1,661324282	-1,124209334		▼-1,44	▲1,66	▼-1,12
ENSG00000108953.16	YWHAE	-1,868590959	1,663484755	-1,325834991		▼-1,87	▲1,66	▼-1,33
ENSG00000112715.20	VEGFA	16,77311714	20,43038205	12,97428558		▲16,77	▲20,43	▲12,97
ENSG00000113140.10	SPARC	-3,314994891	1,421910055	1,368243408		▼-3,31	▲1,42	▲1,37
ENSG00000113302.4	IL12B	1,816751114	1,51022065	10,1559889		▲1,82	▲1,51	▲10,16
ENSG00000114391.12	RPL24	-1,535527998	1,4417107	-1,73170255		▼-1,54	▲1,44	▼-1,73
ENSG00000122406.12	RPL5	-2,453163458	3,707219957	-1,895458761		▼-2,45	▲3,71	▼-1,9
ENSG00000136938.8	ANP32B	-2,134090596	3,004576406	-1,135981423		▼-2,13	▲3,	▼-1,14
ENSG00000137710.14	RDX	-1,750820607	1,442965171	-1,991185238		▼-1,75	▲1,44	▼-1,99
ENSG00000137776.16	SLTM	-1,172114175	1,110300892	-1,808585576		▼-1,17	▲1,11	▼-1,81
ENSG00000137801.10	THBS1	-5,965848377	1,775251257	1,249897598		▼-5,97	▲1,78	▲1,25
ENSG00000143545.8	RAB13	-3,07831215	1,849019836	-1,804206529		▼-3,08	▲1,85	▼-1,8
ENSG00000143552.9	NUP210L	3,802783381	2,188258031	12,3820262		▲3,8	▲2,19	▲12,38
ENSG00000162244.10	RPL29	-2,055167647	1,594202326	-1,774480367		▼-2,06	▲1,59	▼-1,77
ENSG00000162618.12	ADGRL4	-1,632557661	2,07316928	12,64114467		▼-1,63	▲2,07	▲12,64
ENSG00000167131.16	CCDC103	4,461717966	1,414961088	1,794832294		▲4,46	▲1,41	▲1,79
ENSG00000170144.18	HNRNPA3	-2,221890605	1,280137888	-1,664464575		▼-2,22	▲1,28	▼-1,66
ENSG00000173511.9	VEGFB	1,56551197	2,843356332	7,691474499		▲1,57	▲2,84	▲7,69
ENSG00000173848.18	NET1	-2,754449822	1,07654907	-2,410993799		▼-2,75	▲1,08	▼-2,41
ENSG00000175130.6	MARCKSL1	-2,566822346	1,29112043	-1,31652		▼-2,57	▲1,29	▼-1,32
ENSG00000175711.8	B3GNTL1	1,187204441	1,094076567	8,697769917		▲1,19	▲1,09	▲8,7
ENSG00000176971.3	FIBIN	7,791973725	5,775322204	5,737277602		▲7,79	▲5,78	▲5,74
ENSG00000182718.16	ANXA2	-2,280735476	1,523581401	-1,775066337		▼-2,28	▲1,52	▼-1,78
ENSG00000186468.12	RPS23	-2,24615957	2,452948535	-1,288042037		▼-2,25	▲2,45	▼-1,29
ENSG00000187514.14	PTMA	-1,735273444	1,769508439	-1,022087993		▼-1,74	▲1,77	▼-1,02
ENSG00000198900.5	TOP1	-1,275976606	1,603745081	-1,7765037		▼-1,28	▲1,6	▼-1,78
ENSG00000199347.1	RNU5E-1	4,553414314	7,561169605	2,997396791		▲4,55	▲7,56	▲3,
ENSG00000199568.1	RNU5A-1	4,294845375	5,664913385	2,199903918		▲4,29	▲5,66	▲2,2
ENSG00000199629.1	RNU1-14P	4,596809167	5,130843853	2,471853112		▲4,6	▲5,13	▲2,47
ENSG00000199805.1	RNU1-134P	3,740640233	4,442164191	1,407887008		▲3,74	▲4,44	▲1,41
ENSG00000200087.1	SNORA73B	4,768101053	3,447026474	1,382076765		▲4,77	▲3,45	▲1,38
ENSG00000200197.1	RNU1-21P	4,531502276	5,215518365	3,799559031		▲4,53	▲5,22	▲3,8
ENSG00000200340.1	RNU1-105P	5,284886089	4,40729103	4,605100005		▲5,28	▲4,41	▲4,61
ENSG00000202347.1	RNU1-16P	4,204701504	2,209750547	3,994794984		▲4,2	▲2,21	▲3,99
ENSG00000206530.8	CFAP44	2,531779724	2,226208839	11,33661181		▲2,53	▲2,23	▲11,34
ENSG00000206596.1	RNU1-27P	2,125330026	2,996840774	4,671383565		▲2,13	▲3,	▲4,67
ENSG00000206624.1	RNU1-39P	3,924874668	3,504450103	2,125352471		▲3,92	▲3,5	▲2,13
ENSG00000212609.1	RNU1-139P	3,890941632	5,222944434	4,127578503		▲3,89	▲5,22	▲4,13
ENSG00000222328.1	RNU2-2P	2,443165548	3,905667535	2,216528585		▲2,44	▲3,91	▲2,22
ENSG00000222426.1	RNU2-50P	4,593932437	4,763775961	3,271299601		▲4,59	▲4,76	▲3,27
ENSG00000244754.8	N4BP2L2	1,895431746	3,189464046	8,926198699		▲1,9	▲3,19	▲8,93
ENSG00000248527.1	MTATP6P1	-1,200272461	-1,096351046	-1,182320789		▼-1,2	▼-1,1	▼-1,18
ENSG00000270103.3	RNU11	3,959277863	2,025405545	2,173000549		▲3,96	▲2,03	▲2,17
ENSG00000273768.1	RNVU1-29	2,115969848	3,07665443	9,281915367		▲2,12	▲3,08	▲9,28
ENSG00000275538.1	RNVU1-19	6,358317231	4,302804769	3,042133501		▲6,36	▲4,3	▲3,04
ENSG00000275757.1	RNA5-8SN1	11,43176857	4,886540808	3,781339587		▲11,43	▲4,89	▲3,78
ENSG00000276027.1	RNU12	4,192157488	4,828585445	4,108012842		▲4,19	▲4,83	▲4,11
ENSG00000277918.1	RNVU1-28	3,271339421	1,606673972	1,502952209		▲3,27	▲1,61	▲1,5
ENSG00000278099.1	RNVU1-2A	7,181929453	5,210702519	2,379372713		▲7,18	▲5,21	▲2,38
ENSG00000280466.1	SCARNA4	3,406569284	4,754728299	11,29999233		▲3,41	▲4,75	▲11,3

**Suppl. Table 7:** Expression of pro-angiogenic genes in HTB-EVs after LNP treatment			
**Ensembl ID**	**GeneSymbol**	**Averaged normalised untreated HTB-EVs counts**	**Averaged normalised LNP treated HTB-EVs counts**
ENSG00000127837	AAMP	5,208153652	4,37464576
ENSG00000144476	ACKR3	4,39980351	3,955564839
ENSG00000184009	ACTG1	9,110587351	7,777063986
ENSG00000139567	ACVRL1	5,095742614	4,236487479
ENSG00000143537	ADAM15	5,002593745	4,865439721
ENSG00000151651	ADAM8	3,599063981	3,68594612
ENSG00000020181	ADGRA2	4,384179408	3,906009147
ENSG00000205336	ADGRG1	5,110230184	4,629902776
ENSG00000128165	ADM2	3,84802959	4,096679215
ENSG00000164252	AGGF1	4,796340089	4,957526906
ENSG00000164022	AIMP1	4,998347002	4,347578256
ENSG00000126016	AMOT	5,074952552	4,858561238
ENSG00000166025	AMOTL1	5,733327156	4,82474294
ENSG00000114019	AMOTL2	4,987548554	4,494228663
ENSG00000214274	ANG	3,330662718	2,712240272
ENSG00000154188	ANGPT1	4,597820632	4,529589804
ENSG00000091879	ANGPT2	5,345824976	4,372035102
ENSG00000101280	ANGPT4	4,120223622	4,648060272
ENSG00000132855	ANGPTL3	3,667386408	3,817906069
ENSG00000167772	ANGPTL4	3,774818706	4,169920629
ENSG00000130812	ANGPTL6	3,114354685	3,327125468
ENSG00000166825	ANPEP	5,743147051	3,679244238
ENSG00000182718	ANXA2	8,383187805	6,448160177
ENSG00000248329	APELA	3,419845172	3,557141762
ENSG00000171388	APLN	5,318103436	4,0927003
ENSG00000134817	APLNR	4,079377745	3,704419099
ENSG00000189058	APOD	3,625006361	3,793867558
ENSG00000178878	APOLD1	5,041912292	5,389397993
ENSG00000128805	ARHGAP22	4,268059778	4,647275633
ENSG00000138639	ARHGAP24	4,959865071	4,807249166
ENSG00000110955	ATP5F1B	5,854141385	4,518448518
ENSG00000130770	ATP5IF1	5,759010034	5,009183319
ENSG00000141376	BCAS3	5,328127518	4,939731683
ENSG00000125378	BMP4	4,095289918	3,85938017
ENSG00000172270	BSG	5,231986904	3,990391903
ENSG00000106392	C1GALT1	4,792759491	4,882643182
ENSG00000064989	CALCRL	5,243346892	4,741346619
ENSG00000122786	CALD1	6,998012071	6,262876325
ENSG00000064012	CASP8	4,994189355	5,367162674
ENSG00000105974	CAV1	5,926226232	5,167484966
ENSG00000183287	CCBE1	4,480365209	4,461012501
ENSG00000100147	CCDC134	3,501734297	2,712240272
ENSG00000108691	CCL2	4,253102259	3,695041304
ENSG00000108700	CCL8	3,114235069	3,253316095
ENSG00000118523	CCN2	6,949359895	5,191485081
ENSG00000136999	CCN3	3,198749706	3,783020391
ENSG00000117281	CD160	5,076083627	5,404683368

ENSG00000079385	CEACAM1	4,60695718	4,645863434
ENSG00000135048	CEMIP2	5,554751051	5,37453209
ENSG00000185043	CIB1	3,782860876	3,367218028
ENSG00000169504	CLIC4	5,864068892	4,561920476
ENSG00000204291	COL15A1	4,266411336	4,507665811
ENSG00000182871	COL18A1	5,383030229	3,997509987
ENSG00000169436	COL22A1	3,589171249	3,288753729
ENSG00000187498	COL4A1	6,375683173	4,336924412
ENSG00000134871	COL4A2	6,732083652	4,284417588
ENSG00000144810	COL8A1	5,503381318	4,775620846
ENSG00000171812	COL8A2	3,665600683	3,608810699
ENSG00000173546	CSPG4	4,135455445	3,878029818
ENSG00000189377	CXCL17	3,154113729	3,429499691
ENSG00000169429	CXCL8	4,510521623	6,40623103
ENSG00000186810	CXCR3	3,651378233	3,657392653
ENSG00000138061	CYP1B1	5,270894166	4,958267612
ENSG00000136848	DAB2IP	4,552527118	4,494313587
ENSG00000128917	DLL4	4,662217792	4,962633211
ENSG00000143369	ECM1	4,718559992	4,338084607
ENSG00000249751	ECSCR	5,744640731	2,930014235
ENSG00000127129	EDN2	2,856285109	3,089465284
ENSG00000151617	EDNRA	3,756293504	3,905885156
ENSG00000169242	EFNA1	3,651140522	4,321209946
ENSG00000125266	EFNB2	4,809688819	4,13359894
ENSG00000138798	EGF	4,07501022	4,058357975
ENSG00000172889	EGFL7	4,867774047	2,871691322
ENSG00000172071	EIF2AK3	4,355773884	4,246896629
ENSG00000111145	ELK3	5,585659113	4,176368
ENSG00000161671	EMC10	5,871488586	5,883034768
ENSG00000164035	EMCN	4,62002186	4,224059501
ENSG00000106991	ENG	5,538151995	3,429499691
ENSG00000138792	ENPEP	4,453390969	4,674254397
ENSG00000116016	EPAS1	6,03905549	5,423968739
ENSG00000182585	EPGN	3,390746027	3,480733288
ENSG00000146904	EPHA1	3,670974845	4,080845383
ENSG00000142627	EPHA2	4,42919785	4,09537032
ENSG00000154928	EPHB1	4,030280654	4,620342567
ENSG00000133216	EPHB2	5,686212714	4,359424549
ENSG00000182580	EPHB3	3,533542786	3,949003549
ENSG00000196411	EPHB4	5,099249658	4,247931632
ENSG00000164307	ERAP1	4,893806337	4,702471372
ENSG00000124882	EREG	4,087225217	4,440009345
ENSG00000164283	ESM1	5,189125175	3,644452117
ENSG00000078098	FAP	3,932098934	4,311752165
ENSG00000113578	FGF1	4,642721124	4,461651083
ENSG00000070193	FGF10	3,797015959	4,297613996
ENSG00000156427	FGF18	3,467066598	3,452053013
ENSG00000138685	FGF2	5,102165551	5,383923281
ENSG00000111241	FGF6	2,81443146	2,940133234
ENSG00000102678	FGF9	4,284444656	4,298381191
ENSG00000066468	FGFR2	3,909714699	4,570764641
ENSG00000196924	FLNA	7,842248115	7,102642557

ENSG00000102755	FLT1	5,750032014	5,019579753
ENSG00000037280	FLT4	4,895493707	4,696223906
ENSG00000161791	FMNL3	6,224073854	6,189898749
ENSG00000115414	FN1	9,197526437	7,155767391
ENSG00000054598	FOXC1	4,737488976	5,979748735
ENSG00000163251	FZD5	4,815889936	5,544600001
ENSG00000177283	FZD8	4,208970737	4,67959478
ENSG00000109458	GAB1	4,74026752	4,623101985
ENSG00000263761	GDF2	3,829527025	3,69041385
ENSG00000265107	GJA5	4,806966149	4,769153667
ENSG00000135821	GLUL	5,997737869	5,277395999
ENSG00000120063	GNA13	5,075942485	4,780419978
ENSG00000166923	GREM1	6,012696237	6,101724491
ENSG00000113196	HAND1	3,986319077	3,922961243
ENSG00000164107	HAND2	3,706178195	4,000487354
ENSG00000164683	HEY1	4,113998749	3,724128112
ENSG00000100644	HIF1A	5,586585527	4,722293938
ENSG00000124440	HIF3A	4,562337226	4,549661589
ENSG00000100292	HMOX1	4,224861904	3,941571619
ENSG00000105997	HOXA3	5,054728463	3,950870299
ENSG00000122592	HOXA7	4,161778088	3,684083901
ENSG00000159184	HOXB13	4,552544557	4,81950129
ENSG00000120093	HOXB3	4,629241644	4,461248169
ENSG00000113905	HRG	4,149892634	4,954943549
ENSG00000136720	HS6ST1	4,435909187	4,493412984
ENSG00000142798	HSPG2	8,720348061	4,661014952
ENSG00000109854	HTATIP2	4,789596391	4,538993979
ENSG00000125968	ID1	4,275831495	5,319240711
ENSG00000150782	IL18	4,72738118	4,965393979
ENSG00000161638	ITGA5	6,745293344	4,753606259
ENSG00000138448	ITGAV	5,047682534	4,60191409
ENSG00000119185	ITGB1BP1	4,536066473	4,835684343
ENSG00000101384	JAG1	4,913844628	5,113846688
ENSG00000166086	JAM3	5,126995924	4,42464576
ENSG00000177606	JUN	5,466324731	7,346001654
ENSG00000128052	KDR	5,578980142	3,75724718
ENSG00000102554	KLF5	4,115646398	4,220036254
ENSG00000001631	KRIT1	4,513482241	4,32217644
ENSG00000174697	LEP	4,44331949	4,384429545
ENSG00000116678	LEPR	4,901412365	5,113809044
ENSG00000112062	MAPK14	4,988256275	5,054814169
ENSG00000076706	MCAM	6,410507649	4,296748312
ENSG00000125686	MED1	5,713050361	5,356278137
ENSG00000143995	MEIS1	5,531448442	5,820836419
ENSG00000106511	MEOX2	4,374205476	3,996935839
ENSG00000140545	MFGE8	4,76312009	4,639372635
ENSG00000169330	MINAR1	4,568740344	4,434863355
ENSG00000157227	MMP14	5,373923062	3,948912379
ENSG00000123342	MMP19	4,873364113	4,489547954
ENSG00000087245	MMP2	5,614234712	3,380917737
ENSG00000173269	MMRN2	5,470742798	4,507718623
ENSG00000074842	MYDGF	4,211254116	2,712240272

ENSG00000100345	MYH9	8,738235509	7,466032864
ENSG00000164134	NAA15	5,15428099	4,758431085
ENSG00000115053	NCL	10,75176375	9,275568135
ENSG00000173376	NDNF	4,146632909	3,417736662
ENSG00000131669	NINJ1	4,020735367	4,307754099
ENSG00000164867	NOS3	4,669983368	4,37325032
ENSG00000148400	NOTCH1	5,659661756	4,117422784
ENSG00000007952	NOX1	3,02051965	2,867380794
ENSG00000255346	NOX5	5,581820808	5,819718415
ENSG00000091129	NRCAM	5,14415028	4,401024837
ENSG00000099250	NRP1	6,515335798	5,048910806
ENSG00000118257	NRP2	6,235786424	5,793630324
ENSG00000179915	NRXN1	5,607663155	5,419948372
ENSG00000021645	NRXN3	6,020132283	5,839187055
ENSG00000153989	NUS1	5,146150487	5,021268986
ENSG00000154124	OTULIN	5,280971548	5,645178785
ENSG00000125850	OVOL2	3,414527381	3,503585039
ENSG00000125779	PANK2	5,129818854	4,980485946
ENSG00000197702	PARVA	5,471376417	5,035228214
ENSG00000114209	PDCD10	4,565015491	4,216136996
ENSG00000249915	PDCD6	5,654795121	5,258004036
ENSG00000115539	PDCL3	4,954674005	3,925676571
ENSG00000152270	PDE3B	4,724574838	4,959106477
ENSG00000197461	PDGFA	4,40392896	4,023118901
ENSG00000113721	PDGFRB	4,402880717	4,209528528
ENSG00000119630	PGF	4,794354248	4,359804066
ENSG00000121879	PIK3CA	4,811060148	4,561685686
ENSG00000105851	PIK3CG	4,739620016	4,229633642
ENSG00000276231	PIK3R6	3,435829769	3,428667604
ENSG00000160199	PKNOX1	4,844455732	5,108588337
ENSG00000161714	PLCD3	4,692218831	4,7706832
ENSG00000161381	PLXDC1	4,357753497	4,633561872
ENSG00000004399	PLXND1	6,382269284	4,532646497
ENSG00000101346	POFUT1	5,301484157	4,534319608
ENSG00000154229	PRKCA	5,638584667	5,246759179
ENSG00000184304	PRKD1	3,585386716	3,615404926
ENSG00000105287	PRKD2	4,608659199	3,618417471
ENSG00000183943	PRKX	4,691764639	4,475151314
ENSG00000143125	PROK1	3,420295865	3,48226599
ENSG00000163421	PROK2	3,344054708	3,556948799
ENSG00000171862	PTEN	6,168476987	5,944879869
ENSG00000073756	PTGS2	4,426620933	4,272041146
ENSG00000169398	PTK2	6,193889214	5,318296358
ENSG00000120899	PTK2B	4,255655131	3,846532027
ENSG00000127329	PTPRB	6,00861093	5,519423594
ENSG00000132329	RAMP1	3,015747971	2,712240272
ENSG00000131477	RAMP2	3,436179889	2,867380794
ENSG00000079337	RAPGEF3	5,627675367	5,860169002
ENSG00000105538	RASIP1	4,404700638	4,054247665
ENSG00000168214	RBPJ	4,902091014	4,605220679
ENSG00000143878	RHOB	4,963388962	6,019946259
ENSG00000126785	RHOJ	5,534519611	4,686928702

ENSG00000173821	RNF213	7,006290901	6,720374663
ENSG00000154133	ROBO4	5,639989831	4,372140699
ENSG00000069667	RORA	5,826603667	6,415581899
ENSG00000146374	RSPO3	3,696349754	3,912566738
ENSG00000143556	S100A7	3,472742144	2,712240272
ENSG00000170989	S1PR1	5,006642728	3,590488876
ENSG00000130066	SAT1	5,974730979	6,516152665
ENSG00000171951	SCG2	4,651596328	4,475771903
ENSG00000170381	SEMA3E	4,922412102	5,680382528
ENSG00000196189	SEMA4A	4,995570484	4,80156512
ENSG00000106366	SERPINE1	7,082861753	4,115744779
ENSG00000181555	SETD2	6,125426237	5,776962148
ENSG00000027869	SH2D2A	3,892098842	4,511870988
ENSG00000107338	SHB	3,758533457	3,607358419
ENSG00000160691	SHC1	5,613059491	4,90585335
ENSG00000164690	SHH	4,471583382	4,641286627
ENSG00000096717	SIRT1	4,681835459	4,781417713
ENSG00000140199	SLC12A6	5,260156677	4,677371097
ENSG00000164736	SOX17	4,090595891	2,867380794
ENSG00000203883	SOX18	4,072292841	3,407015314
ENSG00000135250	SRPK2	4,909473022	4,328844837
ENSG00000102359	SRPX2	4,235445569	4,392005071
ENSG00000136011	STAB2	3,382539311	3,833512564
ENSG00000165025	SYK	4,625506231	4,556415704
ENSG00000162367	TAL1	4,578316592	4,393742932
ENSG00000184058	TBX1	3,33384873	3,322814941
ENSG00000121075	TBX4	4,347291805	4,568877724
ENSG00000120156	TEK	4,814767485	3,3812896
ENSG00000163235	TGFA	4,712232088	4,623290628
ENSG00000120708	TGFBI	6,133304379	5,808976559
ENSG00000106799	TGFBR1	5,121617267	5,237491601
ENSG00000005108	THSD7A	5,608621791	5,440677306
ENSG00000154096	THY1	4,587891222	4,127422505
ENSG00000066056	TIE1	5,294584239	4,155063519
ENSG00000166292	TMEM100	4,063628506	4,365760578
ENSG00000185215	TNFAIP2	5,833136041	7,039933342
ENSG00000006327	TNFRSF12A	4,573034446	4,269912543
ENSG00000239697	TNFSF12	3,646204214	3,015872977
ENSG00000106025	TSPAN12	3,807374189	3,517640514
ENSG00000025708	TYMP	3,590342572	4,004815771
ENSG00000153560	UBP1	5,779362671	4,994441945
ENSG00000107731	UNC5B	4,473745034	4,413248505
ENSG00000071246	VASH1	5,964694168	5,120608439
ENSG00000160293	VAV2	4,035040294	4,046373712
ENSG00000134215	VAV3	4,43773251	4,14184381
ENSG00000112715	VEGFA	9,11381039	25,89951115
ENSG00000150630	VEGFC	3,659420857	2,930014235
ENSG00000165197	VEGFD	3,412426789	3,519300148
ENSG00000136451	VEZF1	4,865205353	4,74548949
ENSG00000140105	WARS1	5,412651763	5,249639675
ENSG00000158195	WASF2	5,709307814	4,800343233
ENSG00000154764	WNT7A	4,272908016	4,407898151

ENSG00000100219	XBP1	5,05634012	4,801166756
ENSG00000164924	YWHAZ	6,926289867	5,390556769
ENSG00000163874	ZC3H12A	4,088170922	4,088024805
ENSG00000131845	ZNF304	4,105429589	4,176368

**Suppl. Table 8:** Expression of pro-angiogenic genes in HUVEC-EVs after LNP treatment			
**Ensembl ID**	**GeneSymbol**	**Average normalised untreated HUVEC-EVs counts**	**Average normalised LNP treated HUVEC-EVs counts**
ENSG00000127837	AAMP	6,208132779	5,843478382
ENSG00000144476	ACKR3	5,792312838	5,481567125
ENSG00000184009	ACTG1	6,303042456	7,680975946
ENSG00000139567	ACVRL1	6,082497928	6,231589924
ENSG00000143537	ADAM15	6,053670743	6,025340113
ENSG00000151651	ADAM8	5,688441241	5,627837533
ENSG00000020181	ADGRA2	6,062454371	6,078070726
ENSG00000205336	ADGRG1	6,192547948	5,986666637
ENSG00000128165	ADM2	6,128883986	5,764555599
ENSG00000164252	AGGF1	6,155178881	6,109187965
ENSG00000164022	AIMP1	5,971464842	5,989499237
ENSG00000126016	AMOT	6,413282381	6,420302166
ENSG00000166025	AMOTL1	6,365796739	6,686897763
ENSG00000114019	AMOTL2	6,096516126	5,923422111
ENSG00000214274	ANG	5,643359001	5,342016942
ENSG00000154188	ANGPT1	6,064312706	6,006563833
ENSG00000091879	ANGPT2	6,205630334	6,284450168
ENSG00000101280	ANGPT4	6,119620606	6,051841509
ENSG00000132855	ANGPTL3	5,511716555	5,590137752
ENSG00000167772	ANGPTL4	6,181281149	5,818722118
ENSG00000130812	ANGPTL6	5,605964146	5,710146291
ENSG00000166825	ANPEP	5,85892306	6,096699625
ENSG00000182718	ANXA2	6,503133781	7,268769496
ENSG00000248329	APELA	5,740844407	5,829436343
ENSG00000171388	APLN	5,729047476	5,75245842
ENSG00000134817	APLNR	6,150784811	5,888354899
ENSG00000189058	APOD	5,757294584	5,565183153
ENSG00000178878	APOLD1	6,375592103	6,312703168
ENSG00000128805	ARHGAP22	6,011247276	6,225438765
ENSG00000138639	ARHGAP24	6,107492722	6,140486368
ENSG00000110955	ATP5F1B	5,652891347	5,793526876
ENSG00000130770	ATP5IF1	6,090059053	6,361520392
ENSG00000141376	BCAS3	6,756025853	6,489217864
ENSG00000125378	BMP4	6,022194198	5,93279426
ENSG00000172270	BSG	5,843491809	5,686318244
ENSG00000106392	C1GALT1	6,03130727	6,311471518
ENSG00000064989	CALCRL	5,729409445	5,85999148
ENSG00000122786	CALD1	7,027641142	7,108164198
ENSG00000064012	CASP8	6,270354555	6,284821895
ENSG00000105974	CAV1	6,038427359	6,006865731
ENSG00000183287	CCBE1	6,31065768	6,094711208
ENSG00000100147	CCDC134	5,680384949	5,407425211
ENSG00000108691	CCL2	5,984578611	5,993148989
ENSG00000108700	CCL8	5,411449861	5,787918341
ENSG00000118523	CCN2	6,067086468	6,079790253
ENSG00000136999	CCN3	5,553108957	5,583814882
ENSG00000117281	CD160	6,227243722	6,12656658

ENSG00000079385	CEACAM1	6,498196846	6,197787792
ENSG00000135048	CEMIP2	6,373628073	6,445481513
ENSG00000185043	CIB1	5,434186794	5,456687722
ENSG00000169504	CLIC4	5,916314312	6,485441332
ENSG00000204291	COL15A1	5,877178402	5,831068455
ENSG00000182871	COL18A1	6,131951504	6,279648953
ENSG00000169436	COL22A1	5,989986812	5,992735102
ENSG00000187498	COL4A1	6,217291209	6,614253172
ENSG00000134871	COL4A2	6,160283168	6,646123354
ENSG00000144810	COL8A1	6,137073602	5,963938549
ENSG00000171812	COL8A2	5,997493099	5,820223111
ENSG00000173546	CSPG4	6,15034065	5,882114071
ENSG00000189377	CXCL17	5,87816693	5,837792582
ENSG00000169429	CXCL8	5,421288937	5,480677673
ENSG00000186810	CXCR3	5,765255487	5,505163287
ENSG00000138061	CYP1B1	5,811331727	5,868178157
ENSG00000136848	DAB2IP	6,096530873	6,176580596
ENSG00000128917	DLL4	6,312007605	6,330700627
ENSG00000143369	ECM1	6,071347776	5,925026987
ENSG00000249751	ECSCR	5,467827219	5,750587487
ENSG00000127129	EDN2	5,583612799	5,723073172
ENSG00000151617	EDNRA	5,726378327	5,522894352
ENSG00000169242	EFNA1	5,823109326	5,992614939
ENSG00000125266	EFNB2	6,287123581	6,292784196
ENSG00000138798	EGF	5,860433922	5,875326495
ENSG00000172889	EGFL7	5,701013777	5,638327121
ENSG00000172071	EIF2AK3	5,864982871	5,79580164
ENSG00000111145	ELK3	5,867283286	6,146234354
ENSG00000161671	EMC10	7,809143308	6,971696993
ENSG00000164035	EMCN	5,941620097	6,118405593
ENSG00000106991	ENG	5,743082534	5,975241338
ENSG00000138792	ENPEP	6,316122241	6,199874469
ENSG00000116016	EPAS1	6,196285423	6,400811515
ENSG00000182585	EPGN	5,354197821	5,570852627
ENSG00000146904	EPHA1	5,943541963	5,868974859
ENSG00000142627	EPHA2	5,696036791	5,7893922
ENSG00000154928	EPHB1	6,528123206	6,281514156
ENSG00000133216	EPHB2	6,67281716	6,567888428
ENSG00000182580	EPHB3	5,906021286	5,672008372
ENSG00000196411	EPHB4	6,086036501	5,815352161
ENSG00000164307	ERAP1	6,0211456	6,064283275
ENSG00000124882	EREG	5,889554538	5,837613996
ENSG00000164283	ESM1	5,698230028	5,899146771
ENSG00000078098	FAP	5,978562869	6,028078978
ENSG00000113578	FGF1	6,127970633	5,90273289
ENSG00000070193	FGF10	5,91674635	5,803378151
ENSG00000156427	FGF18	5,792477548	5,661581004
ENSG00000138685	FGF2	6,100611967	6,111301827
ENSG00000111241	FGF6	5,572315042	5,424253238
ENSG00000102678	FGF9	6,096525994	6,31123585
ENSG00000066468	FGFR2	6,214182813	6,046328343
ENSG00000196924	FLNA	6,027070345	6,537315935
ENSG00000102755	FLT1	6,284721157	6,440862124
ENSG00000037280	FLT4	6,229406035	5,951513727

ENSG00000161791	FMNL3	7,117792547	7,232788637
ENSG00000115414	FN1	6,892591077	7,57812111
ENSG00000054598	FOXC1	6,183222635	6,060824133
ENSG00000163251	FZD5	6,255311589	7,047041041
ENSG00000177283	FZD8	5,968242735	5,804595272
ENSG00000109458	GAB1	6,095658799	6,338312597
ENSG00000263761	GDF2	5,805963779	5,758035993
ENSG00000265107	GJA5	6,10142891	6,039394818
ENSG00000135821	GLUL	6,583566408	6,482840833
ENSG00000120063	GNA13	6,098081293	6,135860536
ENSG00000166923	GREM1	6,845392883	6,90643308
ENSG00000113196	HAND1	6,104794014	5,934984757
ENSG00000164107	HAND2	6,004326835	5,689541338
ENSG00000164683	HEY1	5,872703444	5,908738677
ENSG00000100644	HIF1A	5,931648201	6,113496481
ENSG00000124440	HIF3A	6,666372982	6,451231797
ENSG00000100292	HMOX1	5,54739671	5,565183153
ENSG00000105997	HOXA3	6,164214066	6,067491167
ENSG00000122592	HOXA7	5,971198425	5,890665545
ENSG00000159184	HOXB13	6,296954717	6,130949251
ENSG00000120093	HOXB3	6,184420001	6,128097172
ENSG00000113905	HRG	6,476830893	6,300610653
ENSG00000136720	HS6ST1	6,088587991	5,954559123
ENSG00000142798	HSPG2	6,522407638	8,156643023
ENSG00000109854	HTATIP2	6,178371844	6,047977012
ENSG00000125968	ID1	5,66561466	5,527799427
ENSG00000150782	IL18	6,431828047	6,20625318
ENSG00000161638	ITGA5	6,483726827	6,457736961
ENSG00000138448	ITGAV	5,77725715	5,855600473
ENSG00000119185	ITGB1BP1	6,000701493	5,957489295
ENSG00000101384	JAG1	6,206615578	6,30118283
ENSG00000166086	JAM3	6,016889144	5,910162053
ENSG00000177606	JUN	6,027174679	6,031334467
ENSG00000128052	KDR	5,948431869	6,122759845
ENSG00000102554	KLF5	6,15254675	5,786525656
ENSG00000001631	KRIT1	5,975632701	5,986363515
ENSG00000174697	LEP	6,021011797	5,823527186
ENSG00000116678	LEPR	6,043945086	6,168605277
ENSG00000112062	MAPK14	6,30546779	6,344004955
ENSG00000076706	MCAM	6,215947238	6,293482335
ENSG00000125686	MED1	6,495420643	6,520084709
ENSG00000143995	MEIS1	6,68028619	6,780730383
ENSG00000106511	MEOX2	5,939716976	5,852044178
ENSG00000140545	MFGE8	6,26991684	6,129283679
ENSG00000169330	MINAR1	6,1962013	6,04908241
ENSG00000157227	MMP14	6,02440106	6,298659231
ENSG00000123342	MMP19	6,126453402	6,106292586
ENSG00000087245	MMP2	5,77164024	6,204349794
ENSG00000173269	MMRN2	6,109756989	6,085137492
ENSG00000074842	MYDGF	5,382427517	5,53527297
ENSG00000100345	MYH9	6,830753642	7,700840332
ENSG00000164134	NAA15	6,126548953	6,33036341
ENSG00000115053	NCL	6,779922234	7,860896151
ENSG00000173376	NDNF	6,004896791	6,041685297

ENSG00000131669	NINJ1	5,691848004	5,826131493
ENSG00000164867	NOS3	5,907639731	5,997024569
ENSG00000148400	NOTCH1	6,081388212	6,135101196
ENSG00000007952	NOX1	5,421815401	5,363051708
ENSG00000255346	NOX5	6,879413396	6,673523781
ENSG00000091129	NRCAM	5,794401657	5,815077197
ENSG00000099250	NRP1	6,57097619	6,682628768
ENSG00000118257	NRP2	6,752958686	6,919663825
ENSG00000179915	NRXN1	6,770635205	6,8943585
ENSG00000021645	NRXN3	7,182835854	7,022661823
ENSG00000153989	NUS1	6,116298786	6,155465736
ENSG00000154124	OTULIN	6,492972264	6,495774781
ENSG00000125850	OVOL2	5,81164895	5,34063721
ENSG00000125779	PANK2	6,105030788	6,125404454
ENSG00000197702	PARVA	6,898190598	6,487641433
ENSG00000114209	PDCD10	5,803682026	5,63013438
ENSG00000249915	PDCD6	6,502034459	6,530913624
ENSG00000115539	PDCL3	5,865905031	6,039817425
ENSG00000152270	PDE3B	6,332442617	6,299731783
ENSG00000197461	PDGFA	5,792082461	5,850974784
ENSG00000113721	PDGFRB	6,142290259	5,643758759
ENSG00000119630	PGF	6,143979923	6,129416469
ENSG00000121879	PIK3CA	6,055338784	6,274965382
ENSG00000105851	PIK3CG	6,075569761	6,090194459
ENSG00000276231	PIK3R6	5,654338329	5,653183583
ENSG00000160199	PKNOX1	6,391472987	6,459298325
ENSG00000161714	PLCD3	6,171843348	5,929647459
ENSG00000161381	PLXDC1	6,150324852	6,186920876
ENSG00000004399	PLXND1	6,332276547	6,513371981
ENSG00000101346	POFUT1	6,200386568	6,100493553
ENSG00000154229	PRKCA	6,58282412	6,240725122
ENSG00000184304	PRKD1	5,759918116	5,719385276
ENSG00000105287	PRKD2	6,2677068	5,887872003
ENSG00000183943	PRKX	5,870934116	5,979741944
ENSG00000143125	PROK1	5,635088826	5,598831834
ENSG00000163421	PROK2	5,553057057	5,631403713
ENSG00000171862	PTEN	6,333654173	6,369442573
ENSG00000073756	PTGS2	5,698766164	5,818038379
ENSG00000169398	PTK2	6,443640669	6,586123531
ENSG00000120899	PTK2B	6,243306407	5,996646227
ENSG00000127329	PTPRB	6,608179717	6,679187728
ENSG00000132329	RAMP1	5,481274113	5,31448642
ENSG00000131477	RAMP2	5,709994732	5,48384752
ENSG00000079337	RAPGEF3	7,161868262	6,750614195
ENSG00000105538	RASIP1	5,982371413	5,649626783
ENSG00000168214	RBPJ	6,153757968	6,251094932
ENSG00000143878	RHOB	5,739825092	6,057729186
ENSG00000126785	RHOJ	6,045633874	6,566145382
ENSG00000173821	RNF213	7,098996366	7,042026012
ENSG00000154133	ROBO4	6,421287713	6,516259699
ENSG00000069667	RORA	6,767055328	6,806798583
ENSG00000146374	RSPO3	5,786657769	5,867537024
ENSG00000143556	S100A7	5,336864926	5,247698419
ENSG00000170989	S1PR1	6,045098051	6,228023211

ENSG00000130066	SAT1	5,835784543	6,721368666
ENSG00000171951	SCG2	5,969042225	5,984171386
ENSG00000170381	SEMA3E	6,399996603	6,373254469
ENSG00000196189	SEMA4A	6,552982061	6,070662266
ENSG00000106366	SERPINE1	6,021838245	6,239307676
ENSG00000181555	SETD2	6,658298506	6,799642342
ENSG00000027869	SH2D2A	5,983077257	5,851694313
ENSG00000107338	SHB	5,578783716	5,507392574
ENSG00000160691	SHC1	5,805555336	5,934847119
ENSG00000164690	SHH	6,166372825	5,99950939
ENSG00000096717	SIRT1	5,985489148	6,194823503
ENSG00000140199	SLC12A6	6,4602833	6,266016823
ENSG00000164736	SOX17	5,624054236	5,853015254
ENSG00000203883	SOX18	5,616170384	5,770169005
ENSG00000135250	SRPK2	5,732951744	5,970281025
ENSG00000102359	SRPX2	5,842849856	5,808306924
ENSG00000136011	STAB2	5,855310272	5,755844897
ENSG00000165025	SYK	6,172520077	6,212827454
ENSG00000162367	TAL1	6,011508585	5,957489295
ENSG00000184058	TBX1	5,861266354	6,204676637
ENSG00000121075	TBX4	6,057619852	5,974943438
ENSG00000120156	TEK	5,935523112	5,882932712
ENSG00000163235	TGFA	6,258375965	6,125256519
ENSG00000120708	TGFBI	6,754567087	6,456402497
ENSG00000106799	TGFBR1	6,03320733	6,259145919
ENSG00000005108	THSD7A	6,521037323	6,560936008
ENSG00000154096	THY1	6,443962984	6,339822615
ENSG00000066056	TIE1	6,242648258	6,185265031
ENSG00000166292	TMEM100	5,837548592	5,792606816
ENSG00000185215	TNFAIP2	6,05747071	5,768511874
ENSG00000006327	TNFRSF12A	5,612239603	5,424253238
ENSG00000239697	TNFSF12	5,726404969	5,363051708
ENSG00000106025	TSPAN12	5,600485093	5,714891592
ENSG00000025708	TYMP	5,758490271	5,673916374
ENSG00000153560	UBP1	6,500680567	6,338429586
ENSG00000107731	UNC5B	6,106035672	6,032918832
ENSG00000071246	VASH1	6,617600438	6,797133772
ENSG00000160293	VAV2	5,848459851	5,760347829
ENSG00000134215	VAV3	6,002396625	6,103792445
ENSG00000112715	VEGFA	7,117943814	26,62693261
ENSG00000150630	VEGFC	5,49237854	5,638194158
ENSG00000165197	VEGFD	5,530413055	5,754804133
ENSG00000136451	VEZF1	5,981652617	6,121491755
ENSG00000140105	WARS1	6,066463664	6,164004544
ENSG00000158195	WASF2	6,374053931	6,249262735
ENSG00000154764	WNT7A	6,175784646	6,079100624
ENSG00000100219	XBP1	5,875624572	5,782197938
ENSG00000164924	YWHAZ	6,29195382	6,676680473
ENSG00000163874	ZC3H12A	5,93037097	5,614579017
ENSG00000131845	ZNF304	5,822534498	5,977660468

**Suppl. Table 9:** Expression of pro-angiogenic genes in CPC-EVs after LNP treatment			
**Ensembl ID**	**GeneSymbol**	**Average normalised untretaed CPC-EVs counts**	**Average normalised LNP treated CPC-EVs counts**
ENSG00000112715	VEGFA	16,65490909	29,92193444
ENSG00000100345	MYH9	9,902387693	10,0470353
ENSG00000115053	NCL	10,87605174	9,921589838
ENSG00000115414	FN1	9,649530949	9,897562572
ENSG00000122786	CALD1	9,520380566	9,658639603
ENSG00000184009	ACTG1	9,819391796	9,427009677
ENSG00000196924	FLNA	8,828689606	8,929491858
ENSG00000182871	COL18A1	8,013662779	7,669014945
ENSG00000164924	YWHAZ	7,404976837	7,052245855
ENSG00000134871	COL4A2	7,234348909	6,974386324
ENSG00000177606	JUN	7,086769061	6,858267298
ENSG00000173821	RNF213	6,419910756	6,762176259
ENSG00000118257	NRP2	5,784791526	6,744109438
ENSG00000130770	ATP5IF1	6,746141228	6,547233609
ENSG00000164107	HAND2	5,563344809	6,531080591
ENSG00000142798	HSPG2	6,855346594	6,453802939
ENSG00000172270	BSG	6,822258991	6,372200341
ENSG00000100644	HIF1A	5,975798432	6,3435874
ENSG00000120063	GNA13	5,400705119	6,251985399
ENSG00000166025	AMOTL1	7,183577961	6,187487114
ENSG00000187498	COL4A1	6,910542424	6,125677588
ENSG00000143878	RHOB	6,283694904	6,102086776
ENSG00000120708	TGFBI	4,700552862	6,090288197
ENSG00000181555	SETD2	6,382708506	6,08464068
ENSG00000114019	AMOTL2	6,533994622	5,959549511
ENSG00000116016	EPAS1	5,099113222	5,948452168
ENSG00000113196	HAND1	6,250099622	5,948426663
ENSG00000087245	MMP2	6,349228395	5,909658702
ENSG00000101384	JAG1	5,106143446	5,877337298
ENSG00000161638	ITGA5	5,534605002	5,787241293
ENSG00000125968	ID1	6,646685921	5,750263032
ENSG00000054598	FOXC1	5,138251877	5,69671831
ENSG00000159184	HOXB13	4,122877653	5,675113845
ENSG00000107731	UNC5B	5,134158092	5,657948782
ENSG00000169398	PTK2	6,315454655	5,627386523
ENSG00000004399	PLXND1	6,015192453	5,581433016
ENSG00000171862	PTEN	5,398465639	5,580642447
ENSG00000135250	SRPK2	5,650055822	5,558590736
ENSG00000138448	ITGAV	4,891843154	5,483206021
ENSG00000179915	NRXN1	4,303776316	5,41516363
ENSG00000148400	NOTCH1	5,432388821	5,408804104
ENSG00000182718	ANXA2	6,859450515	5,382178683
ENSG00000169504	CLIC4	5,587505202	5,366682911
ENSG00000126016	AMOT	5,868095112	5,28403363
ENSG00000177283	FZD8	4,957229839	5,213194375
ENSG00000125686	MED1	5,21373679	5,207067355

ENSG00000157227	MMP14	5,718734377	5,191940149
ENSG00000160691	SHC1	5,416225795	5,191940149
ENSG00000163251	FZD5	4,81379976	5,136805706
ENSG00000106366	SERPINE1	4,48717077	5,12903933
ENSG00000127837	AAMP	5,530510777	5,123897759
ENSG00000158195	WASF2	5,514762975	5,086413552
ENSG00000135821	GLUL	6,194595691	5,024169451
ENSG00000164736	SOX17	6,038505062	5,005630928
ENSG00000110955	ATP5F1B	5,698922712	4,948786431
ENSG00000140545	MFGE8	5,036892183	4,937131505
ENSG00000140105	WARS1	5,435842967	4,857945972
ENSG00000184304	PRKD1	4,708366605	4,831771832
ENSG00000161671	EMC10	5,444090155	4,831271379
ENSG00000153989	NUS1	4,686514004	4,826129807
ENSG00000154096	THY1	4,88968838	4,826129807
ENSG00000100219	XBP1	6,099985799	4,826129807
ENSG00000118523	CCN2	5,563123241	4,819869195
ENSG00000143995	MEIS1	5,099708414	4,789258282
ENSG00000146374	RSPO3	4,742506347	4,714365056
ENSG00000164683	HEY1	4,45724513	4,656528617
ENSG00000130066	SAT1	5,663268627	4,656528617
ENSG00000111145	ELK3	4,803865999	4,651387045
ENSG00000164134	NAA15	5,362166015	4,639363554
ENSG00000125779	PANK2	4,816478395	4,639363554
ENSG00000102554	KLF5	4,972378735	4,597350457
ENSG00000143537	ADAM15	4,100030689	4,543857627
ENSG00000164022	AIMP1	4,884797424	4,543857627
ENSG00000125378	BMP4	4,7324051	4,543857627
ENSG00000133216	EPHB2	5,249969278	4,543857627
ENSG00000161791	FMNL3	5,262170155	4,543857627
ENSG00000113721	PDGFRB	5,017440931	4,534372446
ENSG00000131845	ZNF304	4,219496605	4,534372446
ENSG00000076706	MCAM	4,614151501	4,432092876
ENSG00000099250	NRP1	5,597353968	4,407741462
ENSG00000138792	ENPEP	4,189029926	4,369114866
ENSG00000001631	KRIT1	4,712528396	4,369114866
ENSG00000161714	PLCD3	4,820645228	4,369114866
ENSG00000069667	RORA	4,582941043	4,311439073
ENSG00000125266	EFNB2	5,00472681	4,265905433
ENSG00000182580	EPHB3	4,885606099	4,178953524
ENSG00000114209	PDCD10	4,663769858	4,178953524
ENSG00000006327	TNFRSF12A	4,523889285	4,178953524
ENSG00000071246	VASH1	4,884059507	4,178953524
ENSG00000109458	GAB1	4,759603609	4,173811952
ENSG00000119185	ITGB1BP1	4,367258953	4,173811952
ENSG00000119630	PGF	4,523513594	4,173811952
ENSG00000154229	PRKCA	4,458709637	4,173811952
ENSG00000096717	SIRT1	5,237287429	4,173811952
ENSG00000141376	BCAS3	4,616358396	4,09881045
ENSG00000135048	CEMIP2	5,137714843	4,09881045
ENSG00000136848	DAB2IP	6,140833697	4,09881045

ENSG00000074842	MYDGF	4,370287472	4,09881045
ENSG00000197702	PARVA	5,274740096	4,09881045
ENSG00000249915	PDCD6	4,812916126	4,09881045
ENSG00000106799	TGFBR1	4,808605318	4,09881045
ENSG00000214274	ANG	3,653844524	4,056797353
ENSG00000138685	FGF2	4,339663594	4,056797353
ENSG00000101346	POFUT1	4,341354839	4,056797353
ENSG00000168214	RBPJ	4,707389597	4,056797353
ENSG00000153560	UBP1	5,631173316	4,056797353
ENSG00000134817	APLNR	4,970551601	3,987045699
ENSG00000117281	CD160	3,632211856	3,987045699
ENSG00000169436	COL22A1	4,125184948	3,987045699
ENSG00000091129	NRCAM	4,749069902	3,987045699
ENSG00000105287	PRKD2	4,960754879	3,987045699
ENSG00000196189	SEMA4A	4,237277254	3,987045699
ENSG00000140199	SLC12A6	4,265704857	3,987045699
ENSG00000134215	VAV3	4,056606289	3,987045699
ENSG00000154764	WNT7A	3,971133006	3,987045699
ENSG00000139567	ACVRL1	3,941805574	3,924067688
ENSG00000205336	ADGRG1	4,024108797	3,924067688
ENSG00000128805	ARHGAP22	4,242061754	3,924067688
ENSG00000105974	CAV1	4,14790135	3,924067688
ENSG00000172889	EGFL7	4,16060225	3,924067688
ENSG00000105997	HOXA3	3,978819978	3,924067688
ENSG00000128052	KDR	5,263650337	3,924067688
ENSG00000154124	OTULIN	4,563615315	3,924067688
ENSG00000197461	PDGFA	4,280412313	3,924067688
ENSG00000170989	S1PR1	4,022263579	3,924067688
ENSG00000121075	TBX4	4,260417624	3,924067688
ENSG00000185215	TNFAIP2	5,069978622	3,924067688
ENSG00000150630	VEGFC	3,708179883	3,924067688
ENSG00000079385	CEACAM1	3,863264457	3,891539773
ENSG00000128917	DLL4	4,786073125	3,891539773
ENSG00000066468	FGFR2	5,803664783	3,891539773
ENSG00000100292	HMOX1	4,479437023	3,891539773
ENSG00000136720	HS6ST1	5,224382037	3,891539773
ENSG00000166086	JAM3	4,621956955	3,891539773
ENSG00000112062	MAPK14	4,569497475	3,891539773
ENSG00000105851	PIK3CG	3,725579974	3,891539773
ENSG00000163874	ZC3H12A	4,340353345	3,891539773
ENSG00000144476	ACKR3	4,22213944	3,446492595
ENSG00000151651	ADAM8	4,041909992	3,446492595
ENSG00000020181	ADGRA2	4,687685837	3,446492595
ENSG00000128165	ADM2	4,305734657	3,446492595
ENSG00000164252	AGGF1	4,809121257	3,446492595
ENSG00000154188	ANGPT1	3,707674927	3,446492595
ENSG00000091879	ANGPT2	4,212236012	3,446492595
ENSG00000101280	ANGPT4	3,741784533	3,446492595
ENSG00000167772	ANGPTL4	4,08479737	3,446492595
ENSG00000130812	ANGPTL6	3,642725584	3,446492595
ENSG00000166825	ANPEP	4,224612612	3,446492595

ENSG00000248329	APELA	4,496583823	3,446492595
ENSG00000171388	APLN	3,735789924	3,446492595
ENSG00000189058	APOD	3,566645273	3,446492595
ENSG00000178878	APOLD1	3,893605313	3,446492595
ENSG00000138639	ARHGAP24	5,431990256	3,446492595
ENSG00000106392	C1GALT1	4,255898238	3,446492595
ENSG00000064989	CALCRL	3,675935411	3,446492595
ENSG00000064012	CASP8	4,056845685	3,446492595
ENSG00000183287	CCBE1	4,468683224	3,446492595
ENSG00000100147	CCDC134	4,044379762	3,446492595
ENSG00000108691	CCL2	3,929676772	3,446492595
ENSG00000136999	CCN3	3,595863125	3,446492595
ENSG00000185043	CIB1	4,247457519	3,446492595
ENSG00000204291	COL15A1	3,897978742	3,446492595
ENSG00000144810	COL8A1	3,759397082	3,446492595
ENSG00000171812	COL8A2	3,80138094	3,446492595
ENSG00000173546	CSPG4	4,188819269	3,446492595
ENSG00000189377	CXCL17	3,589943892	3,446492595
ENSG00000169429	CXCL8	3,802556649	3,446492595
ENSG00000186810	CXCR3	3,596890349	3,446492595
ENSG00000138061	CYP1B1	4,207218927	3,446492595
ENSG00000143369	ECM1	3,875297038	3,446492595
ENSG00000249751	ECSCR	3,916103937	3,446492595
ENSG00000127129	EDN2	3,557773478	3,446492595
ENSG00000151617	EDNRA	3,991603202	3,446492595
ENSG00000169242	EFNA1	4,307682051	3,446492595
ENSG00000138798	EGF	3,912395335	3,446492595
ENSG00000172071	EIF2AK3	4,555112137	3,446492595
ENSG00000164035	EMCN	3,681768617	3,446492595
ENSG00000106991	ENG	3,651226781	3,446492595
ENSG00000182585	EPGN	3,492007264	3,446492595
ENSG00000146904	EPHA1	4,225527419	3,446492595
ENSG00000142627	EPHA2	5,631689739	3,446492595
ENSG00000154928	EPHB1	4,442502387	3,446492595
ENSG00000196411	EPHB4	5,855368616	3,446492595
ENSG00000164307	ERAP1	3,972816645	3,446492595
ENSG00000124882	EREG	3,653804311	3,446492595
ENSG00000164283	ESM1	3,623643954	3,446492595
ENSG00000078098	FAP	3,624626672	3,446492595
ENSG00000113578	FGF1	3,886475592	3,446492595
ENSG00000070193	FGF10	4,343739657	3,446492595
ENSG00000156427	FGF18	3,819576689	3,446492595
ENSG00000111241	FGF6	3,636683964	3,446492595
ENSG00000102678	FGF9	3,953519627	3,446492595
ENSG00000102755	FLT1	4,668415701	3,446492595
ENSG00000037280	FLT4	4,142372907	3,446492595
ENSG00000263761	GDF2	3,683270301	3,446492595
ENSG00000265107	GJA5	3,894352461	3,446492595
ENSG00000166923	GREM1	4,47883666	3,446492595
ENSG00000124440	HIF3A	4,439684779	3,446492595
ENSG00000122592	HOXA7	3,657184104	3,446492595

ENSG00000120093	HOXB3	4,379646681	3,446492595
ENSG00000113905	HRG	3,850244432	3,446492595
ENSG00000109854	HTATIP2	4,065748318	3,446492595
ENSG00000150782	IL18	3,643578329	3,446492595
ENSG00000174697	LEP	3,839009616	3,446492595
ENSG00000116678	LEPR	3,95750587	3,446492595
ENSG00000106511	MEOX2	3,886839398	3,446492595
ENSG00000169330	MINAR1	4,354205361	3,446492595
ENSG00000123342	MMP19	3,843608261	3,446492595
ENSG00000173269	MMRN2	3,95832008	3,446492595
ENSG00000173376	NDNF	4,147707607	3,446492595
ENSG00000131669	NINJ1	4,303624615	3,446492595
ENSG00000164867	NOS3	3,942415647	3,446492595
ENSG00000007952	NOX1	3,492007264	3,446492595
ENSG00000255346	NOX5	4,050583645	3,446492595
ENSG00000021645	NRXN3	4,746744747	3,446492595
ENSG00000125850	OVOL2	4,342878596	3,446492595
ENSG00000115539	PDCL3	5,228257352	3,446492595
ENSG00000152270	PDE3B	4,671520851	3,446492595
ENSG00000121879	PIK3CA	4,330762195	3,446492595
ENSG00000276231	PIK3R6	3,566645273	3,446492595
ENSG00000160199	PKNOX1	4,397274768	3,446492595
ENSG00000161381	PLXDC1	3,959705095	3,446492595
ENSG00000183943	PRKX	4,493414294	3,446492595
ENSG00000163421	PROK2	3,627025775	3,446492595
ENSG00000073756	PTGS2	3,706606513	3,446492595
ENSG00000120899	PTK2B	4,412324376	3,446492595
ENSG00000127329	PTPRB	3,874647991	3,446492595
ENSG00000132329	RAMP1	3,705844547	3,446492595
ENSG00000131477	RAMP2	3,691215045	3,446492595
ENSG00000079337	RAPGEF3	4,540510666	3,446492595
ENSG00000105538	RASIP1	3,814385014	3,446492595
ENSG00000126785	RHOJ	3,797173372	3,446492595
ENSG00000154133	ROBO4	3,758651644	3,446492595
ENSG00000171951	SCG2	3,726131735	3,446492595
ENSG00000170381	SEMA3E	4,353667768	3,446492595
ENSG00000027869	SH2D2A	3,672076913	3,446492595
ENSG00000107338	SHB	5,362671653	3,446492595
ENSG00000164690	SHH	3,947765408	3,446492595
ENSG00000203883	SOX18	4,040677943	3,446492595
ENSG00000102359	SRPX2	3,682430834	3,446492595
ENSG00000136011	STAB2	3,598571592	3,446492595
ENSG00000165025	SYK	3,823132208	3,446492595
ENSG00000162367	TAL1	3,907637452	3,446492595
ENSG00000184058	TBX1	4,036022885	3,446492595
ENSG00000120156	TEK	4,083957525	3,446492595
ENSG00000163235	TGFA	3,860905868	3,446492595
ENSG00000005108	THSD7A	4,42194914	3,446492595
ENSG00000066056	TIE1	3,800713867	3,446492595
ENSG00000166292	TMEM100	3,762617312	3,446492595
ENSG00000239697	TNFSF12	3,86816607	3,446492595

ENSG00000106025	TSPAN12	4,332066131	3,446492595
ENSG00000025708	TYMP	3,940635989	3,446492595
ENSG00000160293	VAV2	5,368462061	3,446492595
ENSG00000165197	VEGFD	3,510836078	3,446492595
ENSG00000136451	VEZF1	4,748220552	3,446492595

Suppl Table S10: Top 5 canonical pathways identified in the transcriptome of HTB-EVs, HUVEC-EVs, and CPC-EVs after treatment of their parent cells with LNP-VEGFA mRNA

HTB-EVs

Ingenuity Canonical Pathways	-log(p-value)	Ratio	Molecules	
EIF2 Signaling	4,87×10^01	3,62×10^-01	ACTB,DDIT3,EIF2S2,EIF2S3,EIF3A,EIF3C,EIF3D,EIF3E,EIF3H,EIF3L,EIF4A1,EIF4G1,EIF4G2,EIF4G3,EIF5B,FAU,HNRNPA1,MYC,PABPC1,PIK3C2A,PPP1CA,PPP1R15A,RALA,RASD1,RPL10A,RPL11,RPL12,RPL13A,RPL14,RPL15,RPL17,RPL18,RPL18A,RPL19,RPL21,RPL22,RPL23,RPL23A,RPL24,RPL26,RPL27,RPL29,RPL3,RPL32,RPL34,RPL35A,RPL36A,RPL36AL,RPL37,RPL39,RPL4,RPL5,RPL6,RPL7,RPL7A,RPL8,RPL9,RPLP0,RPLP1,RPLP2,RPS10,RPS11,RPS12,RPS15A,RPS16,RPS17,RPS18,RPS20,RPS23,RPS24,RPS26,RPS27A,RPS3,RPS3A,RPS4X,RPS5,RPS6,RPS8,RPSA,UBA52,VEGFA	
mTOR Signaling	1,6×10^01	2,08×10^-01	CDC42,EIF3A,EIF3C,EIF3D,EIF3E,EIF3H,EIF3L,EIF4A1,EIF4B,EIF4G1,EIF4G2,EIF4G3,FAU,FKBP1A,HIF1A,PIK3C2A,RAC1,RALA,RASD1,RHOA,RHOB,RHOJ,RPS10,RPS11,RPS12,RPS15A,RPS16,RPS17,RPS18,RPS20,RPS23,RPS24,RPS26,RPS27A,RPS3,RPS3A,RPS4X,RPS5,RPS6,RPS6KA2,RPS8,RPSA,VEGFA,VEGFB	
Regulation of eIF4 and p70S6K Signaling	1,5×10^01	2,18×10^-01	EIF2S2,EIF2S3,EIF3A,EIF3C,EIF3D,EIF3E,EIF3H,EIF3L,EIF4A1,EIF4G1,EIF4G2,EIF4G3,FAU,ITGA5,ITGA6,ITGB1,PABPC1,PIK3C2A,RALA,RASD1,RPS10,RPS11,RPS12,RPS15A,RPS16,RPS17,RPS18,RPS20,RPS23,RPS24,RPS26,RPS27A,RPS3,RPS3A,RPS4X,RPS5,RPS6,RPS8,RPSA	
Coronavirus Pathogenesis Pathway	1,38×10^01	1,97×10^-01	CASP3,CXCL8,DDIT3,E2F4,EEF1A1,FAU,FOS,HDAC4,HIF1A,IL6,JAK1,JUN,KPNB1,NFKBIA,NPM1,PA2G4,RAB7A,RPS10,RPS11,RPS12,RPS15A,RPS16,RPS17,RPS18,RPS20,RPS23,RPS24,RPS26,RPS27A,RPS3,RPS3A,RPS4X,RPS5,RPS6,RPS8,RPSA,SERPINE1,TBK1,TGFB1,TGFBR2	
Mitochondrial Dysfunction	1,34×10^01	2,11×10^-01	ACO1,APP,ATP5F1A,ATP5F1B,ATP5F1C,ATP5MG,ATP5PB,ATP5PO,CASP3,COX4I1,COX5A,COX6B2,COX6C,COX7C,CPT1A,CYB5R3,GPX4,GSR,MT-CO1,MT-CO3, NDUFA6, NDUFAB1, NDUFB10, NDUFS4, NDUFS5, PARK7, PDHA1, SOD2, UQCR10, UQCR11, UQCRC1, UQCRH, UQCRQ, VDAC1, VDAC2, VDAC3	
All Canonical Pathways associated with cardiac				
Ingenuity Canonical Pathways	-log(p-value)	Ratio	Molecules	Also contains by other EVs
Role of NFAT in Cardiac Hypertrophy	9,59×10^-01	6,76×10^-02	CALM1,CSNK1A1,GNAI2,GNB1,GNG11,HDAC4,IL6,IL6ST,ITPR2,PDIA3,PIK3C2A,RALA,RASD1,TGFB1,TGFBR2	HUVEC-EVs & CPC-EVs
Cardiac Hypertrophy Signaling (Enhanced)	9,07×10^-01	5,9×10^-02	APEX1,CALM1,CXCL8,DIAPH1,GNAI2,GNB1,GNG11,HDAC4,HSPB1,IL12B,IL18RAP,IL1RL1,IL6,IL6ST,ITGA5,ITGA6,ITGB1,ITPR2,JUN,MYC,PDE6D,PDIA3,PIK3C2A,PKN1,PRKG1,PTK2,RALA,RASD1,RHOA,RPS6,TGFB1,TGFBR2	HUVEC-EVs & CPC-EVs
Apelin Cardiac Fibroblast Signaling Pathway	2,4×10^00	2,17×10^-01	ANGPT2,CCN2,IL6,SERPINE1,TGFB1	CPC-EVs
Cardiac Hypertrophy Signaling	1,88×10^00	8,08×10^-02	CALM1,CDC42,GNAI2,GNB1,GNG11,HSPB1,IL6,JUN,MYL12A,MYL12B,MYL6,PDIA3,PIK3C2A,RAC1,RALA,RASD1,RHOA,RHOB,RHOJ,TGFB1,TGFBR2	HUVEC-EVs & CPC-EVs
Cardiac β-adrenergic Signaling	-0×10^00	3,93×10^-02	AKAP12,APEX1,GNAI2,GNB1,GNG11,PDE6D,PPP1CA	HUVEC-EVs & CPC-EVs

HUVEC-EVs

Ingenuity Canonical Pathways	-log(p-value)	Ratio	Molecules	
EIF2 Signaling	6,43×10^01	4,06×10^-01	ACTB,ATF4,EIF1,EIF2S2,EIF2S3,EIF3A,EIF3D,EIF3J,EIF3L,EIF4A1,EIF4G2,FAU,HNRNPA1,MT-RNR1,MT-RNR2,NKX6-2,PABPC1,PIK3CB,PIK3R5,PPP1R15A,RALA,RALB,RNA5-8SN5,RPL10,RPL11,RPL12,RPL13A,RPL14,RPL15,RPL17,RPL18A,RPL19,RPL21,RPL22,RPL23,RPL23A,RPL24,RPL26,RPL27,RPL27A,RPL28,RPL29,RPL3,RPL31,RPL32,RPL34,RPL35,RPL35A,RPL36,RPL36A,RPL36AL,RPL38,RPL39,RPL4,RPL5,RPL6,RPL7,RPL7A,RPL8,RPL9,RPLP0,RPLP1,RPLP2,RPS10,RPS11,RPS12,RPS13,RPS15,RPS15A,RPS17,RPS18,RPS19,RPS2,RPS20,RPS23,RPS24,RPS25,RPS26,RPS27,RPS27A,RPS27L,RPS3,RPS3A,RPS4X,RPS5,RPS6,RPS8,RPS9,RPSA,VEGFA,XIAP	
mTOR Signaling	2,37×10^01	2,41×10^-01	EIF3A,EIF3D,EIF3J,EIF3L,EIF4A1,EIF4B,EIF4G2,FAU,FKBP1A,MT-RNR1,MT-RNR2,PIK3CB,PIK3R5,PPP2CA,PRKD3,RALA,RALB,RHOA,RHOB,RHOBTB2,RHOJ,RND3,RPS10,RPS11,RPS12,RPS13,RPS15,RPS15A,RPS17,RPS18,RPS19,RPS2,RPS20,RPS23,RPS24,RPS25,RPS26,RPS27,RPS27A,RPS27L,RPS3,RPS3A,RPS4X,RPS5,RPS6,RPS6KA6,RPS8,RPS9,RPSA,VEGFA,VEGFB	
Regulation of eIF4 and p70S6K Signaling	2,36×10^01	2,63×10^-01	EIF1,EIF2S2,EIF2S3,EIF3A,EIF3D,EIF3J,EIF3L,EIF4A1,EIF4G2,FAU,ITGA6,ITGB1,ITGB2,MT-RNR1,MT-RNR2,PABPC1,PIK3CB,PIK3R5,PPP2CA,RALA,RALB,RPS10,RPS11,RPS12,RPS13,RPS15,RPS15A,RPS17,RPS18,RPS19,RPS2,RPS20,RPS23,RPS24,RPS25,RPS26,RPS27,RPS27A,RPS27L,RPS3,RPS3A,RPS4X,RPS5,RPS6,RPS8,RPS9,RPSA	
Coronavirus Pathogenesis Pathway	1,7×10^01	2,07×10^-01	ATF4,BCL2L11,EEF1A1,FAU,HDAC7,JAK1,MAPK8,MT-RNR1,MT-RNR2,NPM1,RAB7A,RBL2,RN7SL1,RN7SL2,RPS10,RPS11,RPS12,RPS13,RPS15,RPS15A,RPS17,RPS18,RPS19,RPS2,RPS20,RPS23,RPS24,RPS25,RPS26,RPS27,RPS27A,RPS27L,RPS3,RPS3A,RPS4X,RPS5,RPS6,RPS8,RPS9,RPSA,STING1,TNPO1	
Germ Cell-Sertoli Cell Junction Signaling	9,06×10^00	1,64×10^-01	ACTB,ACTG1,ACTN4,CFL1,CLINT1,CTNNB1,ITGA6,ITGB1,MAP3K1,MAP3K10,MAP3K14,MAP3K7,MAPK8,MYO7A,PIK3CB,PIK3R5,RAB8B,RALA,RALB,RHOA,RHOB,RHOBTB2,RHOJ,RND3,TUBB,TUBB2A,TUBB4A,TUBB6	
All Canonical Pathways associated with cardiac				
Ingenuity Canonical Pathways	-log(p-value)	Ratio	Molecules	Also contains by other EVs
Cardiac Hypertrophy Signaling	6,12×10^00	1,15×10^-01	ADCY4,ATF2,CACNB3,CACNG4,CACNG7,CALM1 (includes others),GNG11,GNG2,HSPB1,MAP3K1,MAP3K10,MAP3K14,MAP3K7,MAPK8,MEF2A,MYL12A,MYL12B,MYL6,MYL9,NKX2-5,PIK3CB,PIK3R5,PRKAR2B,RALA,RALB,RHOA,RHOB,RHOBTB2,RHOJ,RND3	HTB-EVs & CPC-EVs
Cardiac β-adrenergic Signaling	4,61×10^-01	5,06×10^-02	ADCY4,AKAP12,CACNB3,CACNG4,CACNG7,GNG11,GNG2,PPP2CA,PRKAR2B	HTB-EVs & CPC-EVs
Embryonic Stem Cell Differentiation into Cardiac Lineages	4,52×10^-01	1×10^-01	NKX2-5	CPC-EVs
Role of NFAT in Cardiac Hypertrophy	2,87×10^00	9,01×10^-02	ADCY4,CACNB3,CACNG4,CACNG7,CALM1 (includes others),GNG11,GNG2,HDAC7,IL6ST,MAP3K1,MAP3K7,MAPK8,MEF2A,NKX2-5,PIK3CB,PIK3R5,PRKAR2B,PRKD3,RALA,RALB	HTB-EVs & CPC-EVs
Cardiac Hypertrophy Signaling (Enhanced)	1,8×10^00	6,27×10^-02	ADCY4,ATF2,CACNB3,CACNG4,CACNG7,CALM1 (includes others),CTNNB1,FGFR4,FZD5,GNG11,GNG2,HDAC7,HSPB1,IL12B,IL6ST,ITGA6,ITGB1,ITGB2,MAP3K1,MAP3K10,MAP3K14,MAP3K7,MAPK8,MEF2A,NKX2-5,PIK3CB,PIK3R5,PRKAR2B,PRKD3,RALA,RALB,RHOA,RPS6,WNT7B	HTB-EVs & CPC-EVs

CPC-EVs

Ingenuity Canonical Pathways	-log(p-value)	Ratio	Molecules	
Hepatic Fibrosis / Hepatic Stellate Cell Activation	6,92×10^00	8,76×10^-02	ACTA2,COL12A1,COL1A1,COL25A1,COL3A1,COL6A6,COL9A2,IFNGR2,IGF2,MYH6,MYL4,MYL7,MYO18B,PDGFD,SERPINE1,VEGFA,VEGFB	
Oxidative Phosphorylation	4,42×10^00	9,01×10^-02	ENOX1,MT-ATP6,MT-CO1,MT-CO2,MT-CO3,MT-CYB,MT-ND1,MT-ND2,MT-ND4,MT-ND5	
Calcium Signaling	4,27×10^00	6,42×10^-02	ACTA2,ACTC1,CHRNA10,CHRNA9,HDAC2,MYH6,MYL4,MYL7,MYO18B,NFATC1,PPP3CA,SLC8A3,TNNT2,TPM1	
Tight Junction Signaling	3,94×10^00	6,74×10^-02	ACTA2,ACTC1,AFDN,ARHGEF2,CLDN11,MYH6,MYL4,MYL7,MYO18B,NAPA,NECTIN1,RAB13	
tRNA Splicing	3,74×10^00	1,3×10^-01	PDE4A,PDE5A,PDE6D,TRPT1,TSEN34,TULP2	
Ingenuity Canonical Pathways	-log(p-value)	Ratio	Molecules	Also contains by other EVs
Cardiac β-adrenergic Signaling	9,59×10^-01	3,37×10^-02	GNA13,PDE4A,PDE5A,PDE6D,SLC8A3,TULP2	HTB-EVs & HUVEC-EVs
Embryonic Stem Cell Differentiation into Cardiac Lineages	7,72×10^-01	1×10^-01	ISL1	HUVEC-EVs
Cardiac Hypertrophy Signaling	4,66×10^-01	2,31×10^-02	GNA13,HAND2,MYL4,MYL7,PPP3CA,RHOC	HTB-EVs & HUVEC-EVs
Apelin Cardiac Fibroblast Signaling Pathway	4,6×10^-01	4,35×10^-02	SERPINE1	HTB-EVs
Cardiac Hypertrophy Signaling (Enhanced)	1,12×10^00	2,77×10^-02	CYBB,DIAPH1,GNA13,HAND2,HDAC2,IL12B,ITGAV,MYOCD,NFATC1,PDE4A,PDE5A,PDE6D,PPP3CA,PRKCE,TULP2	HTB-EVs & HUVEC-EVs
Role of NFAT in Cardiac Hypertrophy	-0×10^00	1,8×10^-02	HDAC2,PPP3CA,PRKCE,SLC8A3	HTB-EVs & HUVEC-EVs
